# Nicotinamide Riboside and Metformin Ameliorate Mitophagy Defect in Induced Pluripotent Stem Cell-Derived Astrocytes With *POLG* Mutations

**DOI:** 10.3389/fcell.2021.737304

**Published:** 2021-09-24

**Authors:** Anbin Chen, Cecilie Katrin Kristiansen, Yu Hong, Atefeh Kianian, Evandro Fei Fang, Gareth John Sullivan, Jian Wang, Xingang Li, Laurence A. Bindoff, Kristina Xiao Liang

**Affiliations:** ^1^Department of Neurosurgery, Qilu Hospital and Institute of Brain and Brain-Inspired Science, Cheeloo College of Medicine, Shandong University, Jinan, China; ^2^Shandong Key Laboratory of Brain Function Remodeling, Jinan, China; ^3^Department of Clinical Medicine (K1), University of Bergen, Bergen, Norway; ^4^Neuro-SysMed, Center of Excellence for Clinical Research in Neurological Diseases, Department of Neurology, Haukeland University Hospital, Bergen, Norway; ^5^Department of Clinical Molecular Biology, Akershus University Hospital, University of Oslo, Oslo, Norway; ^6^The Norwegian Centre on Healthy Ageing, Oslo, Norway; ^7^Department of Molecular Medicine, Institute of Basic Medical Sciences, University of Oslo, Oslo, Norway; ^8^Institute of Immunology, Oslo University Hospital, Oslo, Norway; ^9^Hybrid Technology Hub – Centre of Excellence, Institute of Basic Medical Sciences, University of Oslo, Oslo, Norway; ^10^Department of Pediatric Research, Oslo University Hospital, Oslo, Norway; ^11^Department of Biomedicine, University of Bergen, Bergen, Norway

**Keywords:** mitophagy, astrocytes, POLG, IPSC (induced pluripotent stem cells), nicotinamide riboside (NR), metformin, mitochondria

## Abstract

Mitophagy specifically recognizes and removes damaged or superfluous mitochondria to maintain mitochondrial homeostasis and proper neuronal function. Defective mitophagy and the resulting accumulation of damaged mitochondria occur in several neurodegenerative diseases. Previously, we showed mitochondrial dysfunction in astrocytes with *POLG* mutations, and here, we examined how *POLG* mutations affect mitophagy in astrocytes and how this can be ameliorated pharmacologically. Using induced pluripotent stem cell (iPSC)-derived astrocytes carrying *POLG* mutations, we found downregulation of mitophagy/autophagy-related genes using RNA sequencing-based KEGG metabolic pathway analysis. We confirmed a deficit in mitochondrial autophagosome formation under exogenous stress conditions and downregulation of the mitophagy receptor p62, reduced lipidation of LC3B-II, and decreased expression of lysosome protein lysosomal-associated membrane protein 2A (LAMP2A). These changes were regulated by the PINK1/Parkin pathway and AKT/mTOR/AMPK/ULK1 signaling pathways. Importantly, we found that double treatment with nicotinamide riboside (NR) and metformin rescued mitophagy defects and mitochondrial dysfunction in POLG-mutant astrocytes. Our findings reveal that impaired mitophagy is involved in the observed mitochondrial dysfunction caused by *POLG* mutations in astrocytes, potentially contributing to the phenotype in POLG-related diseases. This study also demonstrates the therapeutic potential of NR and metformin in these incurable mitochondrial diseases.

## Graphical Abstract

Hypothetical mechanisms of mitophagy dysfunction in POLG-mutant astrocytes.

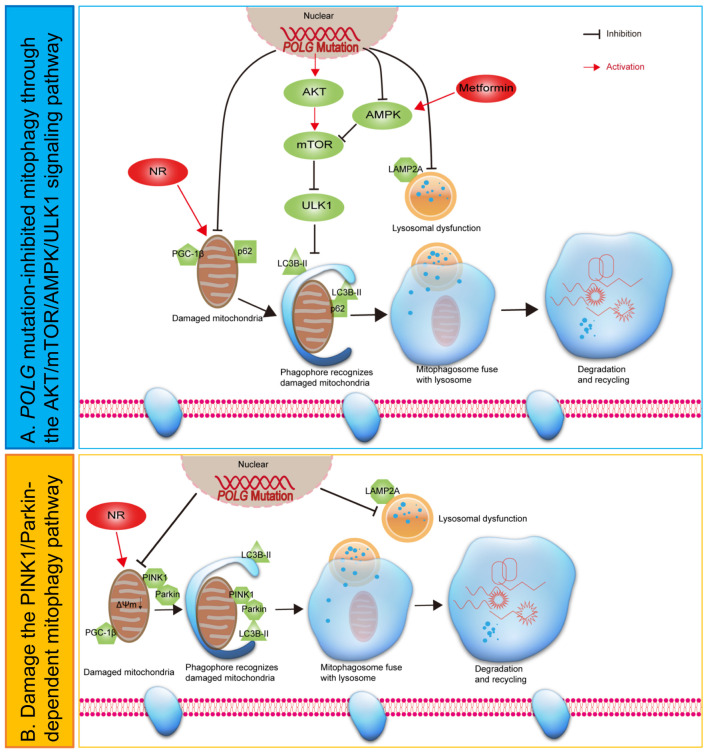

## Introduction

Mitochondrial DNA (mtDNA) polymerase γ (pol γ) replicates the mitochondrial genome and the holoenzyme consists of a catalytic subunit (encoded by *POLG*) and a dimeric form of its accessory subunit (encoded by *POLG*2) (Graziewicz et al., 2006). Mutations in *POLG* are associated with a wide range of mitochondrial diseases that form a continuum from catastrophic early-onset hepato-cerebral degeneration to late-onset progressive external ophthalmoplegia (Hikmat et al., 2020).

Mitochondria are fundamental, subcellular organelles with multiple functions including ATP generation and participating in the regulation of cell metabolism, calcium (Ca2^+^) signaling, redox state, neurotransmission, and plasticity (Lin and Beal, 2006; Mattson et al., 2008; Sheng and Cai, 2012; Fang et al., 2016; Kerr et al., 2017). Mutations in *POLG* lead to neuronal loss conditioned by abnormal mtDNA homeostasis comprising both depletion and a time-dependent increase in mtDNA damage, along with loss of complex I (Tzoulis et al., 2014). We have demonstrated that these findings could be replicated using induced pluripotent stem cell (iPSC)-derived neural stem cells (NSCs) in which we also found evidence of increased reactive oxygen species (ROS) production and cellular senescence (Liang et al., 2020b). In iPSC-derived neurons, we also showed mitochondrial dysfunction and mtDNA depletion in dopaminergic neurons from POLG patients (Liang et al., 2021).

Mitochondria are dynamic organelles, and mitochondrial fusion and fission are essential processes for maintaining homeostasis *via* the segregation of damaged mitochondria (Burté et al., 2015). Fusion and fission are also essential for mitophagy, the selective autophagy pathway that recognizes and degrades damaged or superfluous mitochondria (Youle and Narendra, 2011; Fivenson et al., 2017; Lautrup et al., 2019). Accumulating empirical evidence suggests that mitophagy impairment contributes to neurodegenerative diseases such as Alzheimer’s disease (AD) and Parkinson’s disease (PD) (Kerr et al., 2017; Fang et al., 2019b). Several mitophagy pathways are recognized including the PINK1/Parkin pathway (Vives-Bauza et al., 2010; Deas et al., 2011) and the energy-sensitive PI3K/AKT/mTOR/ULK1 axis (Egan et al., 2011; Hung et al., 2021). The kinase mammalian target of rapamycin (mTOR) is a major negative regulator of mitophagy/autophagy (Heras-Sandoval et al., 2014), and AMP-activated protein kinase (AMPK) is recognized as an important mitophagy activator, acting through a pleiotropic mechanism involving mTOR inhibition and direct phosphorylation/activation of Unc-51-like kinase 1 (ULK1) (Kim et al., 2011; Hung et al., 2021). The mTOR is a downstream target of both phosphatidylinositol 3 kinase (PI3K), and AKT and is activated by receptors of both neurotrophic and growth factors, promoting cell growth, differentiation, and survival (Manning and Toker, 2017).

Astrocytes are the major glial cell type in the central nervous system and serve multiple functions including synaptic signaling, neurotransmitter synthesis and recycling, as well as nutrient uptake and regulation of local blood flow (Haydon and Carmignoto, 2006). Many of these processes depend on local metabolism and/or energy utilization. Astrocytes respond to increases in neuronal activity and metabolic demand by upregulating glycolysis, but also possess a significant capacity for mitochondrial energy metabolism (Jackson and Robinson, 2018). Growing evidence also links astrocyte dysfunction with neurodegenerative disease (Acosta et al., 2017; Liddelow et al., 2017; Richetin et al., 2020; Sonninen et al., 2020). While astrocytic mitophagy/autophagy plays a fundamental role in maintaining brain function and health, disease-driven astrocytic mitophagy/autophagy impairment can potentiate inflammation, oxidative stress, and neural death (Maldonado et al., 2018; Wang and Xu, 2020).

A previous study in mice links mitochondrial dysfunction in astrocytes with POLG-related disease (Ignatenko et al., 2018). In an earlier study, we showed that POLG-mutant astrocytes exhibited mitochondrial dysfunction including loss of mitochondrial membrane potential, loss of ATP production, loss of complex I, disturbed NAD^+^/NADH metabolism, and mtDNA depletion (Liang et al., 2020a). However, whether and how *POLG* mutations affect mitophagy in these cells remains unclear. In this study, we aimed to identify how mitochondrial dysfunction driven by *POLG* mutations impacts mitophagy. We used astrocytes derived from iPSCs of two POLG patients, one homozygous for c.2243G > C; p.W748S and one compound heterozygous for c.1399G > A/c.2243G > C; p.A467T/W748S mutations. We identified a defect in mitophagy with lowered lysosomal activity and abnormal regulation of multiple pathways including PINK1/Parkin and AKT/mTOR/AMPK/ULK1. More importantly, treatment with nicotinamide riboside (NR) and metformin rescued the impaired mitophagy *via* upregulating the SIRT1/AMPK pathway and downregulating the mTOR pathway, which further improved mitochondrial function. Our results show, for the first time, that *POLG* mutations induce mitophagy impairment and that this defect is treatable.

## Materials and Methods

### Ethics Approval

The project was approved by the Western Norway Committee for Ethics in Health Research (REK nr. 2012/919).

### Generation of Induced Pluripotent Stem Cells, Neural Stem Cells, and Astrocyte Differentiation

The fibroblasts from one homozygous c.2243G > C, p.W748S/W748S (WS5A) and one compound heterozygous c.1399G > A/c.2243G > C, p.A467T/W748S (CP2A) patient were collected by punch biopsy. Detroit 551 fibroblasts (ATCC^®^ CCL 110^TM^) and AG05836B fibroblasts (RRID: CVCL_2B58) were used as control lines. The generation and maintenance of iPSCs and their derived NSCs were described previously (Liang et al., 2020a,b, 2021). Astrocytes were differentiated from NSCs according to the protocol described previously (Liang et al., 2020a). In addition, commercial human normal astrocytes (HNA) (Lonza) was applied as a control. Details of the samples used in this study are described in Supplementary Table 1.

### Nicotinamide Riboside and Metformin Treatment

Nicotinamide riboside was provided by Evandro Fei Fang (University of Oslo, Norway). The cells were seeded in six-well plates and treated with metformin (Sigma-Aldrich, St. Louis, MO, United States) and/or NR for 72 h. The medium was changed daily.

### Immunofluorescence Staining

Cells were fixed with 4% (v/v) paraformaldehyde (PFA) and blocked using blocking buffer containing 10% (v/v) normal goat serum (Sigma-Aldrich) with 0.3% (v/v) Triton^TM^ X-100 (Sigma-Aldrich). The cells were then incubated with primary antibody solution overnight at 4°C and further stained with secondary antibody solution (1:800 in blocking buffer) for 1 h at room temperature (RT). The coverslips were mounted onto cover slides using ProLong^TM^ diamond antifade mounting medium with DAPI (Invitrogen, Carlsbad, CA, United States). The detailed information of the antibodies used is provided in Supplementary Tables 2, 3.

### Live Cell Staining for Mitochondrial Morphology

Cells were seeded on μ-Slide 4 Well (Ibidi) and cultured at 37°C in a humidified incubator overnight. The cells were stained with 500 nM MitoTracker Green (MTG) (Invitrogen) for 45 min at 37°C. Images were captured using the Leica TCS SP8 confocal microscope (Leica Microsystems).

### Mitochondrial Volume and Membrane Potential Measurement

Cells were stained with 150 nM MitoTracker Deep Red (MTDR) (Invitrogen) and 150 nM MTG (Invitrogen) for 45 min. Cells treated with 100 μM FCCP (Abcam, Cambridge, MA, United States) were used as negative control. Stained cells were detached with TrypLE^TM^ express (Invitrogen) and analyzed on a FACS BD Accuri^TM^ C6 flow cytometer (BD Biosciences, San Jose, CA, United States). The data analysis was performed using the Accuri^TM^ C6 software (BD Biosciences).

### Mitophagy Detection Assay

Astrocytes were seeded on μ-Slide 4 Well and cultured at 37°C overnight. The cells were then treated with 10 μg/mL FCCP for 24 h and were co-stained with mitophagy dye and lysosome dye using a Mitophagy Detection Kit (Dojindo) according to the instruction. MTDR was used to label the mitochondria. Images were captured using the Leica TCS SP8 confocal microscope (Leica Microsystems, Wetzlar, Germany). Quantitative measurements of immunofluorescence images were performed with Image J software (Image J 1.52a; Wayne Rasband National Institutes of Health, United States^1^). More than five areas were randomly chosen for quantitative evaluations.

### Mitochondrial Complex I Enzyme Activity Assay

We used the Complex I Enzyme Activity Microplate Assay Kit (Abcam: ab109721) to measure complex I activity. All steps were performed according to the manufacturer’s protocol. Cells were seeded in six-well plates at a density of 200,000 cells per well before the initiation of the experiment. Six hours after seeding, cells were treated with different concentrations of metformin for 72 h and then harvested. Extracted samples were loaded into a 96-well microplate and incubated for 3 h before washing three times with buffer. Assay solution was added, and the reaction was measured using a microplate reader (INFINITE F50) with a wavelength of 450 nm for 90 min. Activity is expressed as the change in absorbance per minute (mOD/min) per 130 μg of cell lysate.

### Western Blotting

Extraction of protein was performed using 1× RIPA extraction buffer (Thermo Fisher Scientific, Carlsbad, CA, United States) supplemented with protease inhibitor cocktail and phosphatase inhibitor cocktail (Thermo Fisher Scientific). Protein concentration was determined using Pierce^TM^ BCA Protein Assay Kit (Thermo Fisher Scientific). The cell protein was loaded into NuPAGE^TM^ 4–12% Bis-Tris Protein Gels (Invitrogen) and resolved in the PVDF membrane (BioRad, Hercules, CA, United States) using the Trans-Blot^®^ Turbo^TM^ transfer system (BioRad). Membranes were blocked with 5% non-fat dry milk or 5% bovine serum albumin (BSA) in TBST for 1 h at RT. Membranes were then incubated overnight at 4°C with primary antibodies. After washing in TBST three times, membranes were incubated with donkey anti-mouse antibody or swine anti-rabbit antibody conjugated to horseradish peroxidase secondary for 1 h at RT. Super Signal West Pico Chemiluminescent Substrate Kit (Thermo Fisher Scientific) was used as enzyme-substrate according to the manufacturer’s recommendations. The membranes were visualized by ChemiDoc imaging systems (BioRad). Further details of the used antibodies are provided in Supplementary Tables 2, 3.

### RNA Sequencing

Total RNA was extracted using QIAGEN RNeasy Kit (QIAGEN, Hilden, Germany). Library preparation was conducted at BGI following the guide of the standard protocol. Library preparation (BGISEQ-500RS High-throughput sequencing kit, PE50, V3.0, MGI Tech Co., Ltd., Shenzhen, China), hybridization, and sequencing were performed according to the manufacturer’s procedure from BGI (BGI-Shenzhen, China). The sequencing was performed at BGI-Shenzhen using the BGISEQ-500 system. The sequencing data were filtered to remove very low-quality reads using SOAP nuke (v1.5.2) software. The processed FASTQ files were mapped to the human transcriptome and genome using HISAT2 (v2.0.4). The genome version was GRCh38, with annotations from Bowtie2 (v2.2.5). The expression level of the gene was calculated by RSEM (v1.2.12). Differential expression was done with DEseq2 (v1.4.5) package. To take an insight into the change of phenotype, KEGG^2^ enrichment analysis of annotated differentially expressed genes (DEGs) were performed by Phyper^3^ based on hypergeometric test. Significant DEGs were defined as ones with at least 0.3 fragments per kilobase million (FPKM) level of expression in at least one of the conditions and a *q*-value less than 0.05 by Bonferroni test.

### Statistical Analyses

All data are expressed as mean ± SEM for the number of samples (*n* ≥ 3). Statistical analyses were performed with GraphPad Prism 8.0.2 software (GraphPad Software) using the Mann–Whitney *U* test. Significance is denoted for *p*-values of less than 0.05.

## Results

### Generation and Characterization of Patient-Specific Astrocytes Derived From Induced Pluripotent Stem Cells Carrying *POLG* Mutations

**FIGURE 1:**
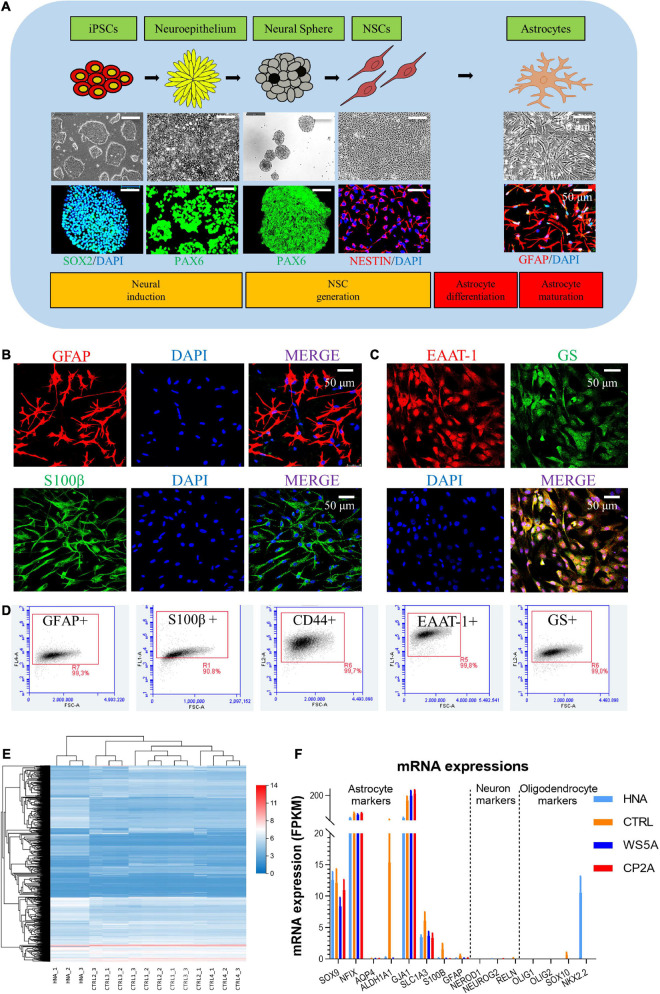
Generation and characterization of patient-specific astrocytes carrying *POLG* mutations. **(A)** Schematic of dual differentiation and seeding paradigm for astrocytes (upper panel), representative phase-contrast images (middle panel) and immunostaining for specific stages during neural induction from iPSCs to astrocytes (lower panel). **(B,C)** Representative phase-contrast images and confocal images of immunostaining for GFAP (red), S100β (green), EAAT-1 (red), and GS (green). Nuclei are stained with DAPI (blue). Scale bar is 50 μm. **(D)** Flow cytometric analysis for percentage of the positive cells expressing GFAP, S100β, CD44, EAAT-1, and GS. **(E)** Clustering heat map [log (value + 1)] for DEGs in HNA and control iPSC-derived astrocytes. **(F)** mRNA expression of astrocyte, neuron, and oligodendrocyte-related genes in HNA and all iPSC-derived astrocytes. The different markers are listed on the *x*-axis, and mRNA expression is on the *y*-axis. Results are expressed as fragments per kilobase million (FPKM). Data information: The data in **B–D** represent one clone from AG05836 control. The data in **E** represent HNA, four different control astrocytes with three clones from Detroit 551 control and one clone from AG05836 control. The data in **F** represent HNA, four different control astrocytes with three clones from Detroit 551 control and one clone from AG05836 control, three clones from WS5A and two clones from CP2A.

To generate astrocytes, we generated iPSCs showing positive expression of SOX2, OCT4, and NANOG (Supplementary Figure 1) and then differentiated these into NSCs as described previously (Liang et al., 2020a,b, 2021). Two patient lines were used, one homozygous for c.2243G > C; p.W748S (WS5A) and the other a compound heterozygous for c.1399G > A/c.2243G > C; p.A467T/W748S (CP2A). We employed two control iPSC lines generated from normal human fibroblast lines, Detroit 551 and AG05836B for wild-type controls. In addition, one commercial human primary astrocyte line, HNA, was used as a positive astrocyte lineage control. We differentiated iPSC-derived NSCs to astrocytes as previously described (Liang et al., 2020a,b, 2021). The astrocytes from all patients and controls (Supplementary Figure 4) showed typical stellate morphology (Figure 1A). To minimize the intra-clonal phenotypic variability, multiple clones from each individual patient were differentiated and included in further studies.

Next, we characterized the iPSC-derived astrocytes using immunostaining against key astrocyte markers. We found robust expression of glial fibrillary acidic protein (GFAP) and S100 calcium-binding protein β (S100β) (Figure 1B and Supplementary Figure 2), and the functional markers excitatory amino acid transporter 1 (EAAT-1, also known as GLAST-1) and glutamine synthetase (GS) (Figure 1C and Supplementary Figure 3). Astrocytic identity and purity were confirmed using flow cytometry against a panel of markers GFAP, S100β, CD44, EAAT-1, and GS (Supplementary Figures 5–9). We found that over 99% of cells were positive for all these markers (Figure 1D). Additionally, we performed transcriptomic analysis to characterize the specific cell type and the level of astrocyte maturation from each individual clone. RNA sequencing (RNA-seq) analysis was performed on three clones from WS5A, two clones from CP2A, one clone from the commercial HNA control, and four control astrocyte clones including one clone from AG05836 and three clones from Detroit 551-derived astrocytes.

Unsupervised hierarchical clustering showed that replicates from the individual clones in the control group clustered together, also clustering closely with HNA (Figure 1E). We found significant homogeneity with a cluster of astrocytic markers *SOX9*, *NFIX*, *AQP4*, *ALDH1A1*, *GJA1*, *SLC1A3*, *S100B*, and *GFAP* in all the samples, but no expression of neuronal markers *NEROD1*, *NEUROG2*, and *RELN* and oligodendrocyte markers *OLIG1*, *OLIG2*, *SOX10*, and *NKX2.2* (Figure 1F).

Collectively, these data suggest that our protocol produces nearly pure cultures of differentiated astrocytes that exhibit similar transcriptional profiles to primary astrocytes.

### Transcriptomic Analysis Shows Downregulated Mitophagy/Autophagy Genes in POLG-Mutant Astrocytes

**FIGURE 2:**
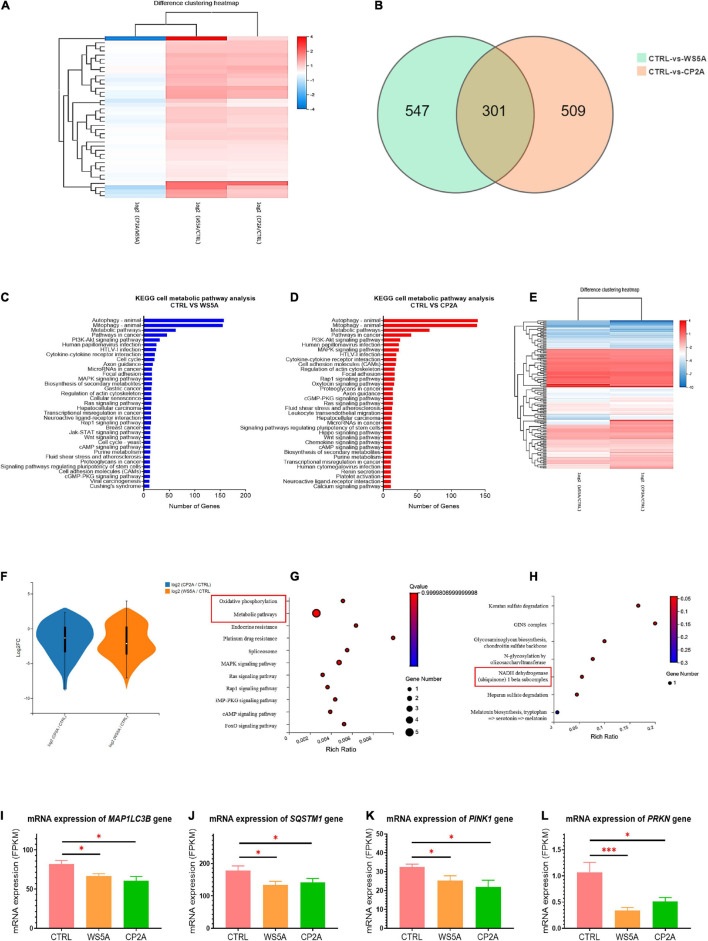
Transcriptomic analysis shows downregulation of mitophagy/autophagy in POLG-mutant astrocytes. **(A)** Clustering heap map [log (value + 1)] for DEGs in WS5A, CP2A, and control astrocytes. **(B)** Venn diagram of the DEGs in two compared groups of WS5A astrocytes vs. controls and CP2A astrocytes vs. controls. **(C,D)** KEGG metabolic pathway analysis for DEGs in two compared groups of WS5A astrocytes vs. controls **(C)** and CP2A astrocytes vs. controls **(D)**. **(E)** Unsupervised clustering of DEGs in mitophagy/autophagy pathways in WS5A astrocytes vs. controls and CP2A astrocytes vs. controls. **(F)** Differential analysis of mitophagy-related genes in patient astrocytes compared to controls. **(G)** KEGG pathway enrichment analysis of the mitophagy-related genes in patient astrocytes compared to controls. **(H)** KEGG module enrichment analysis of the mitophagy-related genes in patient astrocytes compared to controls. **(I–L)** RNA-seq analysis for the mRNA expression of mitophagy-related genes *MAP1LC3B* **(I)**, *SQS1M1* **(J)**, *PINK1* **(K)**, and *PRKN* **(L)** in WS5A, CP2A, and control astrocytes. Results are expressed as FPKM. Data information: The data in **I–L** represent four different control astrocytes, including three clones from Detroit 551 control and one clone from AG05836 control, three clones from WS5A and two clones from CP2A. Data are presented as mean ± SEM for the number of samples (*n* ≥ 3 per clone). Mann–Whitney *U* test was used for the data presented in **I–L**. Significance is denoted for *p*-values of less than 0.05. *p < 0.05; ***p < 0.001.

We explored the DEGs as well as their related signaling and metabolic pathways by comparing the transcriptomic profile of patient (WS5A and CP2A) versus (vs.) control astrocytes. Using unsupervised clustering, we identified a clear clustering of the patient astrocytes (WS5A and CP2A) vs. controls (Figure 2A). The Venn diagram showed 547 genes were significantly differentially expressed in the WS5A lines and 509 in the CP2A lines compared to controls (statistical significance cutoff was *p* = 0.05, Figure 2B).

KEGG metabolic pathway analysis revealed that the greatest enrichment was for genes involved in the mitophagy/autophagy pathways in both patient astrocyte lines (WS5A and CP2A) compared to controls (Figures 2C,D). This was also significant for genes involved in PI3K/AKT signaling pathway (Figures 2C,D). Unsupervised clustering of DEGs in the mitophagy/autophagy pathways demonstrated a similar pattern in WS5A cells vs. controls and CP2A cells vs. controls (Figure 2E). Differential analysis showed the downregulation of mitophagy genes in patient astrocytes compared to controls (Figure 2F). In addition, KEGG pathway enrichment analyses showed that there was also an associated dysregulation of both oxidative phosphorylation (Figure 2G) and other metabolic pathways, especially in NADH dehydrogenase-linked pathways (Figure 2H) in patient astrocytes. We also demonstrated significantly lower expressions of the mitophagy/autophagy-related genes *MAP1LC3B* (Figure 2I), *SQSTM1* (Figure 2J), *PINK1* (Figure 2K), and *PRKN* (Figure 2L) in both WS5A and CP2A lines compared to controls. This evidence suggests linkage between mitophagy changes and POLG-induced mitochondrial dysfunction in these astrocytes.

**FIGURE 3:**
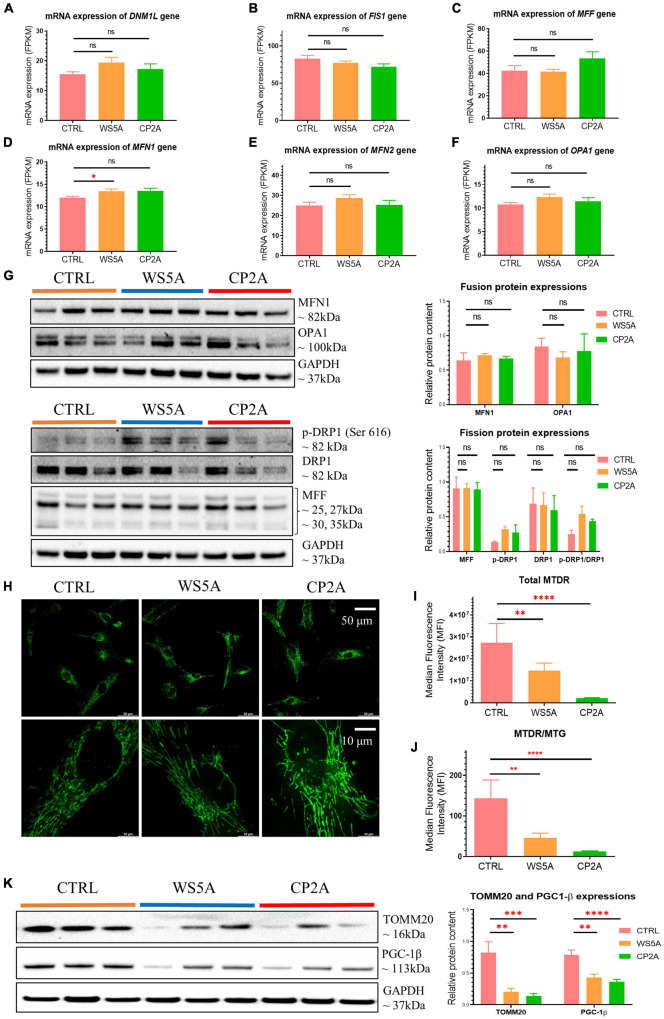
POLG-mutant astrocytes exhibit loss of mitochondrial volume and impaired mitochondrial function. **(A–F)** RNA-seq analysis for the mRNA expression of fusion/fission-related genes *DNM1L* **(A)**, *FIS1* **(B)**, *MFF* **(C)**, *MFN1* **(D)**, *MFN2* **(E)**, and *OPA1* **(F)** in WS5A, CP2A, and control astrocytes. Results are expressed as FPKM. **(G)** Representative images of western blotting for MFN1, OPA1, p-DRP1 (Ser 616), DRP1, MFF, and GAPDH, and quantitative measurements of protein levels of MFN1, OPA1, p-DRP1 (Ser 616), DRP1, and MFF in control and POLG-mutant astrocytes. **(H)** Representative images of live cell staining for MTG. The upper panel scale bar is 50 μm; the lower panel is taken from the upper panel and the scale bar is 10 μm. **(I,J)** Flow cytometric measurements of total MTDR level **(I)** and specific MTDR level normalized with MTG **(J)** in WS5A, CP2A, and control astrocytes. **(K)** Representative images of western blotting for TOMM20, PGC-1β, GAPDH, and quantitative measurements of protein levels of TOMM20 and PGC-1β in control and POLG-mutant astrocytes. Data information: The data in **A–F** represent four different control astrocytes, including three clones from Detroit 551 control and one clone from AG05836 control, three clones from WS5A and two clones from CP2A. The data in **G,K** represent three different control astrocytes, including two clones from Detroit 551 control and one clone from AG05836 control, three clones from WS5A and three clones from CP2A. The data in **H** represent one clone from AG05836 control, one clone from WS5A and one clone from CP2A. The data in **I,J** represent three different control astrocytes, including two clones from Detroit 551 control and one clone from AG05836 control, three clones from WS5A and three clones from CP2A. Data are presented as mean ± SEM for the number of samples (*n* ≥ 3 per clone). Mann–Whitney *U* test was used for the data presented in **A–K**. Significance is denoted for *p*-values of less than 0.05. *p < 0.05; **p < 0.01; ***p < 0.001; ****p < 0.0001; ns, not significant.

Given that mitochondrial dynamics also plays a role in mitochondrial quality control, we assessed transcript levels of mitochondrial fusion/fission-related genes. Expression levels of *DNM1L*, *FIS1*, *MFF*, *MFN2*, and *OPA1* (Figures 3A–C,E,F) were similar in both patient-derived and control astrocytes, while expression of *MFN1* was higher than control in WS5A patient astrocytes (Figure 3D). When we measured protein levels using western blotting, however, we found that levels of fusion proteins mitofusin-1 (MFN1) and optic atrophy (OPA1), as well as fission proteins mitochondrial fission factor (MFF) and dynamin-related protein 1 (DRP1), were unaffected in patient astrocytes (Figure 3G).

These results indicate that *POLG* mutations lead to changes in astrocytes that appear to target the mitophagy/autophagy pathways but not the mitochondrial fission/fusion pathways.

### POLG-Mutant Astrocytes Exhibit Loss of Mitochondrial Volume and Impaired Mitochondrial Function

Next, we asked whether astrocytes generated from POLG patients exhibited changes in mitochondrial volume and function. We investigated mitochondrial morphology using fluorescence microscopy, and biogenesis with western blotting analysis, mitochondrial volume and mitochondrial membrane potential with flow cytometry. We assessed mitochondrial morphology using live cell staining with MTG and found a similar mitochondrial morphology in both patients and controls (Figure 3H).

We then investigated mitochondrial membrane potential. To do this, we used flow cytometry and the combination of the mitochondrial potential-dependent dye MTDR to measure membrane potential and a mitochondrial potential-independent dye MTG to assess mitochondrial volume. We found significantly lower total MTDR (Figure 3I) and specific MTDR when normalized to mitochondrial mass MTG (Figure 3J) in both patient-derived astrocytes (WS5A and CP2A) as compared to controls.

Next, we quantified mitochondrial proteins by western blotting and observed decreased levels of TOMM20 in both patient lines (WS5A and CP2A) compared to controls (Figure 3K). To investigate if the decrease reflected downregulated mitochondrial biogenesis, we assessed PGC-1β, a positive regulator of mitochondrial biogenesis and respiration. We observed a significant decrease in PGC-1β in both patient-derived astrocytes (WS5A and CP2A) vs. controls (Figure 3K).

These findings demonstrate that *POLG* mutations in astrocytes are associated with loss of mitochondrial volume, decreased mitochondrial membrane potential, and biogenesis.

### POLG-Mutant Astrocytes Show Impaired Mitophagy Regulation *via* PINK1/Parkin Signaling Pathway

**FIGURE 4:**
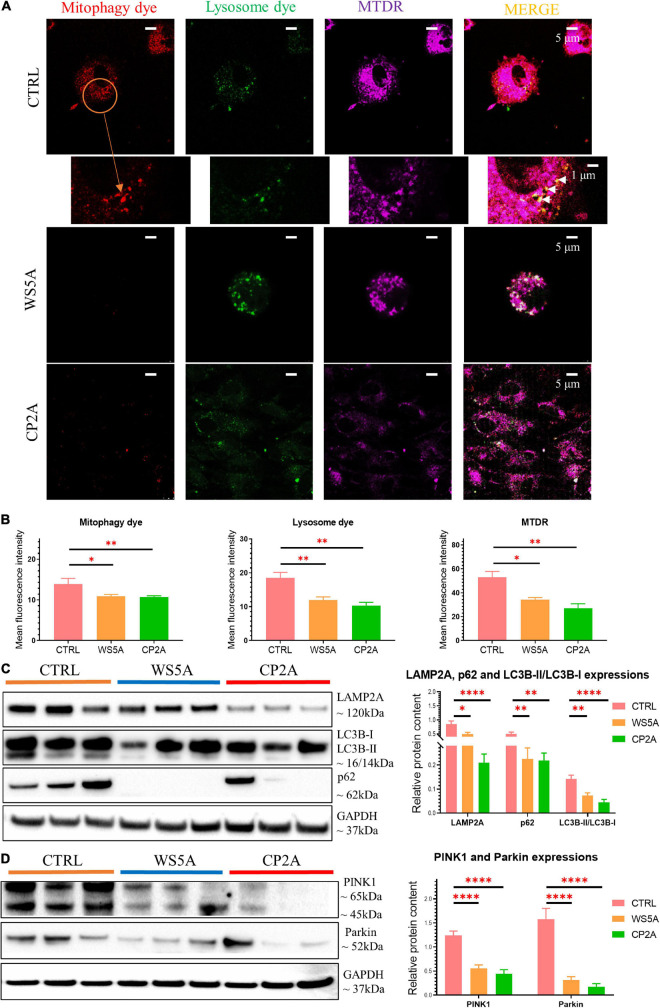
POLG-mutant astrocytes show impaired mitophagy regulation *via* PINK1/Parkin signaling pathway. **(A)** Representative confocal images of immunostaining for mitophagy dye (red), lysosome dye (green), MTDR (purple), and MERGE (yellow) in WS5A, CP2A, and control astrocytes. Scale bar is 5 or 1 μm. White arrows denote mitophagy formation. The cells were treated with FCCP for 24 h before staining. **(B)** Quantification of the immunofluorescence levels of mitophagy dye, lysosome dye, and MTDR in WS5A, CP2A, and control astrocytes. **(C)** Representative images of western blotting for LAMP2A, p62, LC3B-I, LC3B-II, and quantitative measurements of protein levels in WS5A, CP2A, and control astrocytes. **(D)** Representative images of western blotting for PINK1, Parkin and quantitative measurements of protein levels of PINK1, Parkin in WS5A, CP2A, and control astrocytes. Data information: the data in **A** represent one clone from Detroit 551 control, one clone from WS5A, and one clone from CP2A. The data in **B**, 40,000 astrocytes per sample were seeded and four different control astrocytes, including three clones from Detroit 551 control and one clone from AG05836 control, two clones from WS5A, and two clones from CP2A were observed with three technical replicates. The data in **C,D** represent three different control astrocytes, including two clones from Detroit 551 control and one clone from AG05836 control, three clones from WS5A and three clones from CP2A. Data are presented as mean ± SEM for the number of samples (*n* ≥ 3 per clone). Mann–Whitney *U* test was used for the data presented in **B–D**. Significance is calculated by comparison to controls and is denoted for *p*-values of less than 0.05. *p < 0.05; **p < 0.01; ****p < 0.0001.

Evidence from RNA-seq analysis suggested downregulation of mitophagy-related genes in patient iPSC-derived astrocytes. We investigated this further by treating astrocytes with carbonyl cyanide 4 – (trifluoromethoxy) phenylhydrazone (FCCP) – to induce mitophagy and then evaluated mitophagosome formation using a fluorescent mitophagy dye, together with a lysosome dye and the mitochondrial dye MTDR. We observed decreased red fluorescent puncta representing mitophagosome formation and green fluorescent intensity for measuring lysosomes in both patient and control astrocytes (Figure 4A). Quantification of the mean fluorescence intensity in more than five random areas showed that this decrease was significant for both mitophagy (Figure 4B) and lysosome formation (Figure 4B) in patient astrocytes. Consistent with the flow cytometric measurements (Figures 3I,J), MTDR staining demonstrated a significantly lower expression level in patient astrocytes as compared to controls (Figure 4B). These data suggest that astrocytes with *POLG* mutations lead to impaired mitophagy and decreased lysosome formation.

Next, we examined mitophagy-related proteins including the autophagosome marker microtubule-associated protein 1 light chain 3β (LC3B), autophagy receptor p62, and lysosomal marker lysosomal-associated membrane protein 2A (LAMP2A) using western blotting. We found a decreased level of LC3B-II with a significantly decreased ratio of LC3B-II/LC3B-I in both WS5A and CP2A astrocytes compared to controls, indicating reduced mitophagy in POLG-mutant astrocytes (Figure 4C). We also identified the decreased protein expression of p62 (Figure 4C) and downregulation of LAMP2A (Figure 4C) in POLG-mutant astrocytes vs. control cells.

Loss mitochondrial membrane potential leads to activation of PINK1, which stimulates the recruitment of Parkin to the mitochondrial outer membrane of damaged mitochondria and activates Parkin’s ubiquitin-ligase activity. Activated mitochondrial Parkin leads to the ubiquitination of mitochondrial proteins and subsequent mitophagy. We investigated PINK1/Parkin pathway using western blotting and found downregulation of PINK1 and Parkin in patient astrocytes as compared to controls (Figure 4D), suggesting that *POLG* mutations may impair mitochondrial priming of mitophagy in astrocytes *via* PINK1/Parkin axis.

These findings suggest that *POLG* mutations can lead to impaired mitophagy and decreased lysosome formation *via* PINK1/Parkin-dependent pathway.

### POLG-Mutant Astrocytes Demonstrate Mitophagy Impairment *via* Modulating AKT/mTOR/AMPK/ULK1 Signaling Axis

**FIGURE 5:**
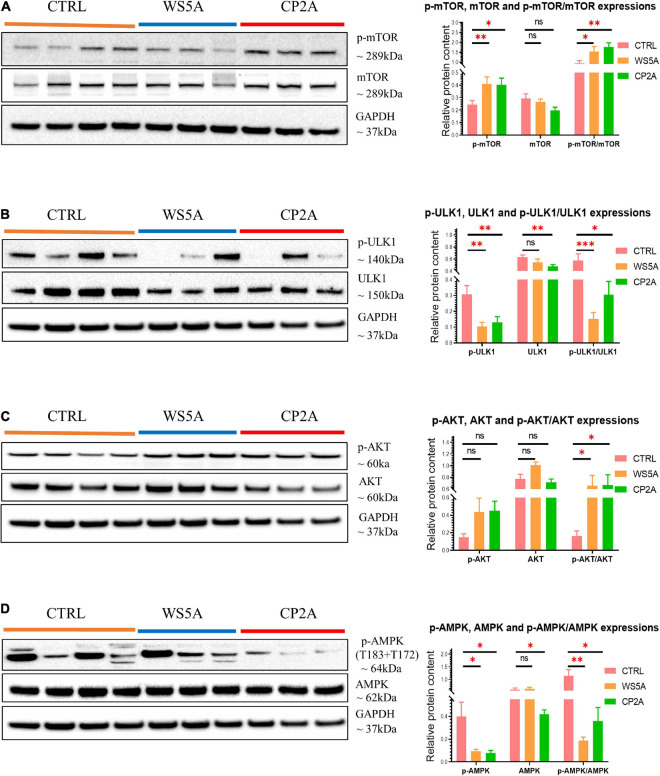
POLG-mutant astrocytes demonstrate mitophagy impairment *via* modulating AKT/mTOR/AMPK/ULK1 signaling axis. **(A)** Representative images of western blotting for p-mTOR, mTOR, GAPDH, and quantitative measurements of protein levels in WS5A, CP2A, and control astrocytes. **(B)** Representative images of western blotting for p-ULK1, ULK1, GAPDH, and quantitative measurements of protein levels in WS5A, CP2A, and control astrocytes. **(C)** Representative images of western blotting for p-AKT, AKT, GAPDH, and quantitative measurements of protein levels of in WS5A, CP2A, and control astrocytes. **(D)** Representative images of western blotting for p-AMPK (T183 + T172), AMPK, GAPDH, and quantitative measurements of protein levels in WS5A, CP2A, and control astrocytes. Data information: the loading controls from **D** are re-used from **B,C**. The data in **A–D** represent four different control astrocytes, including three clones from Detroit 551 control and one clone from AG05836 control, three clones from WS5A and three clones from CP2A. Data are presented as mean ± SEM for the number of samples (*n* ≥ 3 per clone). Mann–Whitney *U* test was used for the data presented in **A–D**. Significance is denoted for *p*-values of less than 0.05. *p < 0.05; **p < 0.01; ***p < 0.001; ns, not significant.

The mTOR signaling pathway plays a pivotal role in regulating metabolism (Lazarou et al., 2015; Perluigi et al., 2015). Abnormal activation of the AKT/mTOR/AMPK signaling pathway inhibits autophagy, and defects affecting this pathway are associated with human neurodegenerative disease (Saxton and Sabatini, 2017). Therefore, we asked if abnormal AKT/mTOR/AMPK signaling was driving the mitophagy impairment in POLG-mutant astrocytes. To address this question, we used western blotting to analyze p-mTOR, mTOR, p-AKT, AKT, p-ULK1, ULK1, p-AMPK (T183 + T172), and AMPK protein levels. We found a significantly increased ratio of p-mTOR/mTOR in both patient astrocytes compared to controls, indicating abnormal activation of the mTOR signaling pathway (Figure 5A). We also demonstrated a significantly increased ratio of p-AKT/AKT (Figure 5C) and a decrease in the ratio of p-ULK1/ULK1 (Figure 5B) and p-AMPK (T183 + T172)/AMPK (Figure 5D).

Taken together, these data indicate that *POLG* mutations induced astrocytic mitophagy failure by regulating the AKT/mTOR/AMPK/ULK1 signaling pathway.

### Nicotinamide Riboside and Metformin Treatment Rescue Mitophagy Defect by Modulated SIRT1/AMPK/mTOR Pathway in POLG-Mutant Astrocytes

**FIGURE 6:**
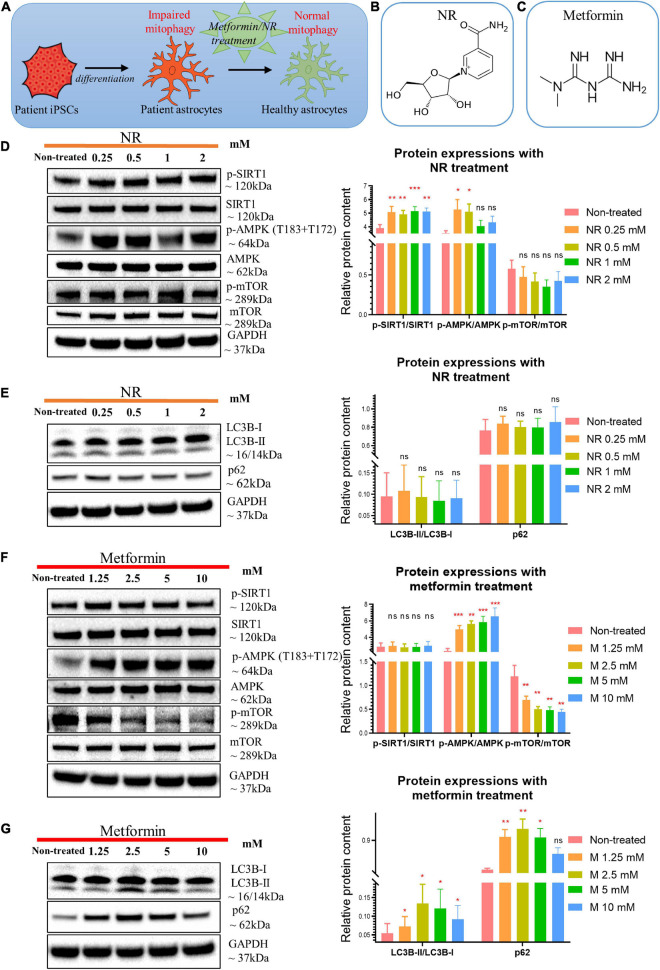
Nicotinamide riboside and metformin treatment modulated SIRT1/AMPK/mTOR pathway in POLG-mutant astrocytes. **(A)** Schematic diagram of compound treatment for POLG-mutant astrocytes. **(B,C)** Chemical structure of NR **(B)** and metformin **(C)**. **(D)** Representative images and quantification of western blotting for p-SIRT1, SIRT-1, p-AMPK (T183 + T172), AMPK, p-mTOR, mTOR, and GAPDH in the POLG WS5A astrocytes treated with a serial concentration of NR alone for 72 h. **(E)** Representative images and quantification of the western blotting for LC3B-I, LC3B-II, p62, and GAPDH in the POLG WS5A astrocytes treated with a serial concentration of NR alone for 72 h. **(F)** Representative images and quantification of western blotting for p-SIRT1, SIRT1, p-AMPK (T183 + T172), AMPK, p-mTOR, mTOR, and GAPDH in the POLG WS5A astrocytes treated with a serial concentration of metformin alone for 72 h. **(G)** Representative images and quantification of the western blotting for LC3B-I, LC3B-II, p62, and GAPDH in the POLG WS5A astrocytes treated with a serial concentration of metformin alone for 72 h. Data information: the data in **D–G** represent one clone from WS5A. Mann–Whitney *U* test was used for the data presented in **D–G**. Significance is calculated by comparison to non-treated cells and is denoted for *p*-values of less than 0.05. *p < 0.05; **p < 0.01; ***p < 0.001; ns, not significant.

Next, we asked whether the mitophagy defect could be rescued (Figure 6A). The NAD^+^ precursor NR (Figure 6B) potentially activates mitophagy/autophagy through the SIRT1 or mTOR pathways (Cea et al., 2012; Fang et al., 2017; Vannini et al., 2019; Zheng et al., 2019). We therefore treated WS5A astrocytes for 72 h with varying concentrations of NR: 0, 0.25, 0.5, 1, and 2 mM. Cell viability was maintained under all treatment conditions (Supplementary Figure 10). Using western blotting, we found that cells treated with NR alone showed upregulated phosphorylation of SIRT1/SIRT1 (p-SIRT1/SIRT1) ratio at all drug concentrations. We also identified an increased p-AMPK (T183 + T172)/AMPK ratio, but only in cells treated with 0.25 and 0.5 mM NR (Figure 6D). No significant change in the mTOR pathway (Figure 6D) or the mitophagy-related proteins LC3B-II/LC3B-I and p62 (Figure 6E) were identified.

The AMPK activator metformin (Figure 6C) improves mitophagy (Bhansali et al., 2020; Bharath et al., 2020; Kodali et al., 2021) and mitochondrial function (Bharath et al., 2020) *via* AMPK pathway activation. Therefore, we treated WS5A astrocytes for 72 h with a series of metformin concentrations: 0, 1.25, 2.5, 5, and 10 mM. We observed that cell viability was maintained under all treatment conditions (Supplementary Figure 11). We found upregulated AMPK signaling with increased phosphorylation of AMPK at T183 + T172/AMPK ratio at all concentrations (Figure 6F). Metformin-treated cells showed a dose-dependent decrease in phosphorylated mTOR relative to total mTOR (Figure 6F) and an increased LC3B-II/LC3B-I ratio, which peaked in cells treated with 2.5 mM, and increased p62 at all concentrations except 10 mM (Figure 6G).

**FIGURE 7:**
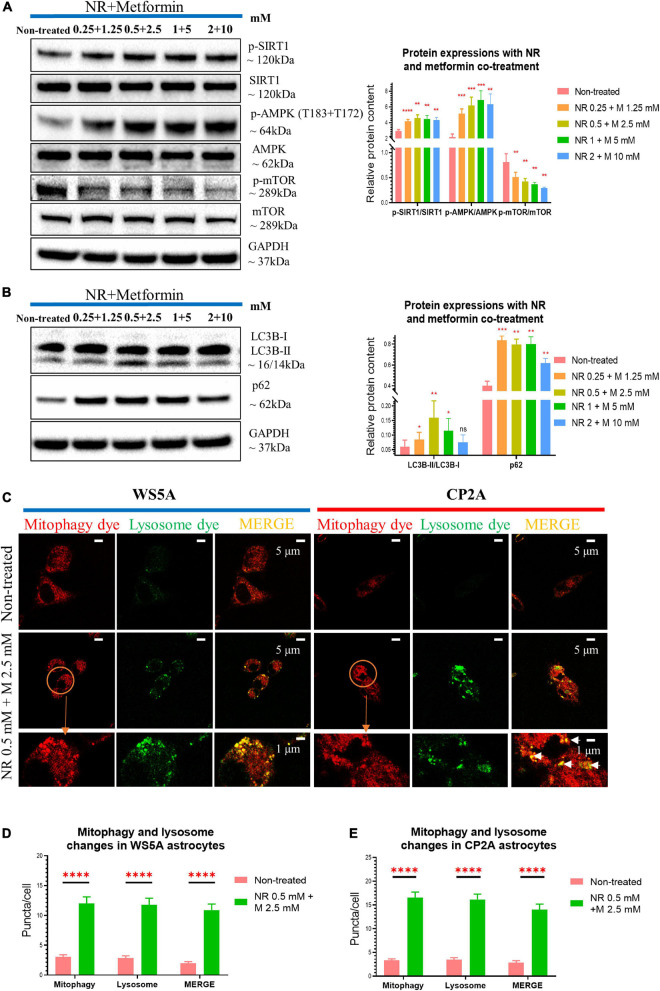
Nicotinamide riboside and metformin co-treatment rescue mitophagy defect in POLG-mutant astrocytes. **(A)** Representative images and quantification of western blotting for p-SIRT1, SIRT1, p-AMPK (T183 + T172), AMPK, p-mTOR, mTOR, GAPDH, and in the POLG WS5A astrocytes treated with a serial concentration of NR and metformin co-treatment for 72 h. **(B)** Representative images and quantification of the western blotting for LC3B-I, LC3B-II, p62, and GAPDH in the POLG WS5A astrocytes treated with a serial concentration of NR and metformin co-treatment for 72 h. **(C)** Representative confocal images of immunostaining for mitophagy dye (red), lysosome dye (green), and MERGE (yellow) in WS5A and CP2A astrocytes with and without NR and metformin dual treatment. Scale bar is 5 or 1 μm. White arrows denote mitophagy formation. **(D,E)** Quantification of the puncta numbers/cell of mitophagy dye, lysosome dye, and MERGE in WS5A **(D)** and CP2A astrocytes **(E)** with and without NR and metformin co-treatment. Data information: the data in **A,B** represent one clone from WS5A. The data in **C** represent one clone from WS5A with and without co-treatment, one clone from CP2A with and without co-treatment. The data in **D,E**, 40,000 astrocytes per sample were seeded and two clones from WS5A with and without co-treatment and two clones from CP2A with and without co-treatment were observed with three technical replicates. Mann–Whitney *U* test was used for the data presented in **A,B,D,E**. Significance is calculated by comparison to non-treated cells and is denoted for *p*-values of less than 0.05. *p < 0.05; **p < 0.01; ***p < 0.001; ****p < 0.0001; ns, not significant.

We then examined whether combining NR and metformin gave additional benefit. Cells were treated with the following concentrations: 0 mM, NR 0.25 mM + metformin 1.25 mM, NR 0.5 mM + metformin 2.5 mM, NR 1 mM + metformin 5 mM, and NR 2 mM + metformin 10 mM. Cell growth was maintained under all treatment conditions (Supplementary Figure 12). Combined treatment gave increased levels of p-SIRT1/SIRT1 ratio and p-AMPK (T183 + T172)/AMPK ratio, but decreased level of p-mTOR/mTOR ratio at all concentrations (Figure 7A). Dual treatment increased LC3B-II/LC3B-I ratio, showing a peak in the cells treated with 0.5 mM NR and 2.5 mM metformin, while 10 mM did not reach significance (Figure 7B). The level of p62 increased at all concentrations (Figure 7B).

To confirm whether the dual treatment could rescue the mitophagy, we treated both WS5A and CP2A astrocytes and performed the mitophagy detection assay described above. When incubated with FCCP for 24 h, we observed increased mitophagy (higher red fluorescent signal) and lysosomal formation (elevated green, fluorescent puncta) in the co-treated patient cells as compared to controls (Figure 7C). Quantification of the fluorescent signals showed significantly increased levels for both mitophagy and lysosomal formation (Figures 7D,E) in both patient iPSC-derived astrocytes after the treatment.

Collectively, our data suggest NR alone and metformin alone modulate mitophagy in POLG-mutant astrocytes in different manners, while dual treatment of NR and metformin provides a quantifiable improvement of the mitophagy impairment *via* regulating SIRT1/AMPK/mTOR signaling pathway in POLG-mutant astrocytes.

### Nicotinamide Riboside and Metformin Treatment Improve Mitochondrial Function in POLG-Mutant Astrocytes

**FIGURE 8:**
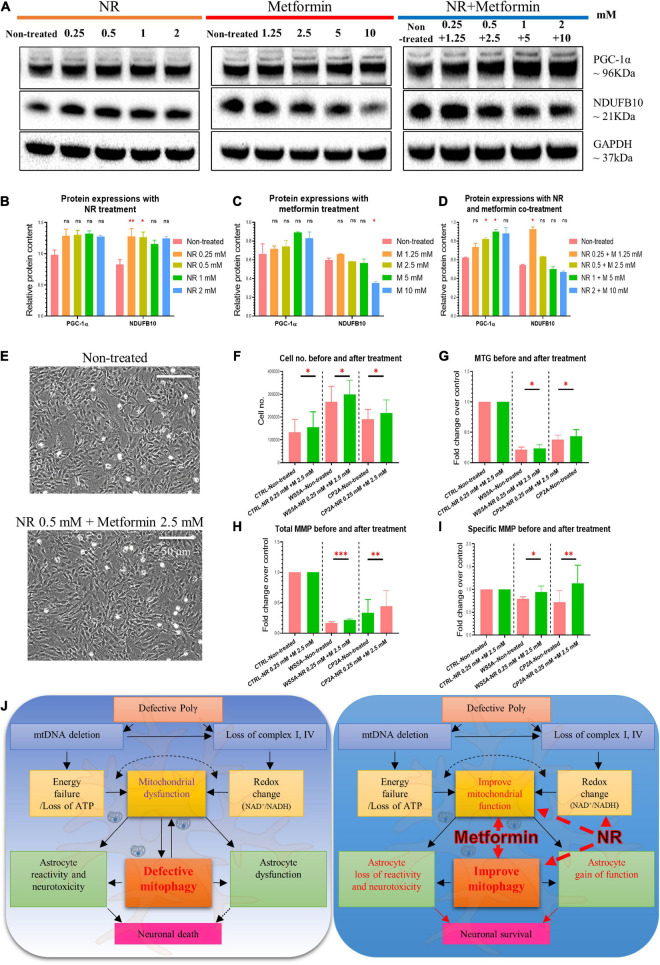
Nicotinamide riboside and metformin treatment improve mitochondrial function in POLG-mutant astrocytes. **(A)** Representative images of the western blotting for NDUFB10, PGC-1α, and GAPDH in the POLG WS5A astrocytes treated with a serial concentration of NR alone, metformin alone, and co-treatment for 72 h. **(B–D)** Protein quantifications of the western blotting in **A** for NDUFB10 and PGC-1α in the POLG WS5A astrocytes treated with a serial concentration of NR alone **(B)**, metformin alone **(C)**, and co-treatment **(D)** for 72 h. **(E)** Representative phase-contrast images of WS5A astrocytes before and after the dual treatment of NR 0.5 mM and metformin 2.5 mM. Scale bar is 50 μm. **(F)** Cell number counting of WS5A astrocytes before and after the dual treatment of NR 0.5 mM and metformin 2.5 mM. **(G–I)** Flow cytometric analysis for MTG **(G)** and MMP at total level measured by TMRE **(H)** and specific MMP level calculated by total TMRE/MTG **(I)** of WS5A astrocytes before and after the dual treatment of NR 0.5 mM and metformin 2.5 mM. MTG, MMP, and specific MMP were normalized with non-treated cells. **(J)** Summary of the possible disease mechanisms in astrocyte defects and neuronal loss in POLG-related disorders. Data information: the data in **A–I** represent one clone from WS5A. Mann–Whitney *U* test was used for the data presented in **B–D** and **F–I**. Significance is calculated by comparison to non-treated cells and is denoted for *p*-values of less than 0.05. *p < 0.05; **p < 0.01; ***p < 0.001; ns, not significant.

Metformin is a known inhibitor of respiratory chain complex I, but this has been shown to be concentration dependent (Owen et al., 2000). There is convincing evidence that inhibition of complex I occurs only at higher metformin concentrations, while at lower concentrations, it improves mitochondrial respiratory chain function (Wang et al., 2019). To check the effect of our treatment, we treated cells with NR, metformin, or both using the following concentrations: 0 mM, NR 0.25 mM + metformin 1.25 mM, NR 0.5 mM + metformin 2.5 mM, NR 1 mM + metformin 5 mM, and NR 2 mM + metformin 10 mM and measured the complex I subunit NDUFB10 expression using western blotting. We observed increased complex I expression with metformin alone or co-treatment when this drug was used at lower concentrations (Figures 8A,C,D). In addition, we found increased complex I enzyme activity in cells treated with metformin 1.25 mM, but not with other concentrations (Supplementary Figure 13). To investigate whether NR and metformin treatment could improve the mitochondrial biogenesis in POLG-mutant astrocytes, we then examined PGC-1α expression using western blotting and found that PGC-1α also tended to increase in patient astrocytes under all treatment conditions, but only at the concentration of the NR 0.5 mM + metformin 2.5 mM and NR 1 mM + metformin 5 mM with significant statistical significance (Figures 8A–D).

Next, we checked the effect of treatment on cell growth, mitochondrial mass, and membrane potential. We treated astrocytes with 0.5 mM NR and 2.5 mM metformin, the concentrations demonstrating the most significant effect as defined in earlier assays. We found that combined treatment did lead to an increase in cell number (Figures 8E,F), mitochondrial mass (Figure 8G), and mitochondrial membrane potential; both total (Figure 8H) and specific levels normalized to mass (Figure 8I) although the increase was small.

Taken together, these data indicate that NR and metformin treatment improved the mitochondrial biogenesis and functions in POLG-mutant astrocytes, suggesting the mitophagy enhancers as potential treatments for POLG-related disorders.

## Discussion

*POLG* mutations impair the replication of mtDNA and cause a range of mitochondrial diseases many of which affect the brain (Hikmat et al., 2020). We showed it was possible to replicate the cellular phenotypes of mitochondrial dysfunction and abnormal NAD^+^ metabolism in neural cells (Liang et al., 2020b, 2021) and confirmed these changes in iPSC-derived astrocytes (Liang et al., 2020a). Guided by unsupervised transcriptomic analysis, which indicated a major dysregulation of mitophagy/autophagy, we found that *POLG* mutations were clearly associated with impaired mitophagy in astrocytes. This impairment occurs *via* multiple mechanisms including (a) downregulation of the autophagic/mitophagic protein p62 and reduced lipidation of LC3B-II, (b) impaired autophagosome–lysosome fusion, (c) downregulation of the PINK1/Parkin pathway, and (d) dysregulation of AKT/mTOR/AMPK/ULK1 pathway. Further and most notably, we demonstrate that NR and metformin treatment improved the impaired mitophagy machinery and appeared to restore the mitochondrial defect. This opens new possibilities for the treatment of these disorders by using mitophagy-enhancing reagents.

In our study, we chose healthy, age-matched control iPSCs and patient-specific iPSCs then differentiated them into astrocytes using a modified technique (Liang et al., 2020a,b, 2021). We are fully aware that the current state of the art for iPSC studies is to use gene-corrected isogenic controls; however, the presence of compound mutations, such as those used in this study (CP2A mutation), makes the generation of individual controls impracticable. At the same time, it has been stated that the high efficiency of genome cutting and repair makes the introduction of heterozygous alleles by the standard CRISPR/Cas9 technique near impossible (EauClaire and Webb, 2019; Liang et al., 2020a,b, 2021).

We performed RNA-seq analysis to confirm the cellular identity and screen for changes in cellular pathways affected by the presence of *POLG* mutations in astrocytes (Liang et al., 2020a). We found that both patient and control astrocytes showed similar astrocytic lineage profiles. Pathway analysis showed clear dysregulation of genes involved in mitophagy/autophagy, with both patient lines being downregulated. This was confirmed by assessing the individual genes involved in mitophagy/autophagy. We identified multiple downregulated genes in several mitophagy/autophagy-related pathways including the PI3K/AKT signaling pathway and cAMP signaling pathway as well as metabolic pathways. While mitochondrial fusion and fission are important activities for the removal of defective organelles *via* mitophagy, we observed similar transcriptomic profiles in patient and control astrocytes. Our data therefore suggest that while *POLG* mutations lead to the downregulation of mitophagy/autophagy-related genes, this appears to be independent of mitochondrial fission/fusion.

To investigate how dysregulated mitophagy might impact POLG affected mitochondria, we looked at changes in mitochondrial function including mitochondrial volume, membrane potential, and biogenesis. We found that POLG-mutant astrocytes had reduced mitochondrial volume, lower membrane potential, and decreased mitochondrial biogenesis factor PGC-1β. This suggests that POLG not only disrupts mitophagy regulation but might also impair mitochondrial biogenesis. Thus, our study suggests that mitochondrial biogenesis and mitophagy are coordinated to regulate mitochondrial homeostasis and maintain cellular energy levels in tissues such as the brain.

Consistent with the transcriptomic findings, we observed that mitophagy activity was diminished under FCCP-induced conditions in both patient cell lines. When we measured the LC3 conversion (LC3B-I–LC3B-II) by western blot analysis, the steady-state levels of LC3B-II were significantly lower in both patient lines. There was also a lower expression of p62. Autophagic adapter protein p62 is the substrate of Parkin and is required for Parkin-induced mitophagy (Song et al., 2016). Thus, aberrant p62 could influence the balance of mitophagy and further disturb mitochondrial quality control. Accumulation of misfolded proteins is known to induce aberrant p62 expression, and p62 is involved in multiple pathways associated with neurodegeneration (Homma et al., 2014; Ma et al., 2019). Abnormal expression of p62 can induce mitophagy and autophagy dysfunction, which can further accelerate misfolded protein aggregation. Based on our study, the data suggest that p62 is associated with POLG-related diseases and impaired Parkin-mediated mitophagy. To dissect what was driving the impairment of mitophagy, we examined the molecular signaling pathways involved. We showed that *POLG* mutations impaired mitochondrial priming of mitophagy *via* the PINK1/Parkin pathway. In mammalian cells, lower mitochondrial membrane potential leads to accumulation of PINK1 on the outer mitochondrial membrane (OMM) and subsequent recruitment of the E3 ubiquitin ligase Parkin. Parkin-mediated ubiquitination of OMM proteins serves as a general signal recognized by autophagy adaptor proteins, such as p62, which further target LC3B and ubiquitin chains, translocating these dysfunctional mitochondria into phagophores. We previously demonstrated the downregulation of the PINK1/Parkin pathway and showed that *POLG* mutations drive a lower level of ATP in astrocytes (Liang et al., 2020a). Given that ATP is a key regulator of PINK1-mediated mitophagy by controlling PINK1 translation levels (Lee et al., 2015), we suggest that POLG-induced ATP depletion downregulates the translation of PINK1 and therefore the recruitment of Parkin in response to mitochondrial depolarization.

We also detected reduced lysosome formation in POLG-mutant astrocytes following FCCP treatment and a reduction in the protein level of lysosome-related membrane protein LAMP2A, indicating damage or dysfunction of lysosomes. Damaged mitochondria are degraded by the autophagy-lysosome pathway (Plotegher and Duchen, 2017), and studies in PARK2-PD fibroblasts suggest that lysosomal function may be influenced by mitochondrial quality control (Guerra et al., 2019). Our data suggest that mutations in POLG induce mitochondrial dysfunction that leads to lysosomal impairment and impairment of mitophagosome-lysosome fusion. Lysosomal impairment, in turn, can trigger mitophagy failure and functional mitochondrial defects in astrocytes. In addition, LAMP2A mediates lysosome fusion and is essential for the final stage of mitophagy. A recent study showed the LAMP2A is required for the clearance of mitochondria but is dispensable for PINK1/Parkin-mediated mitophagy (Liu et al., 2020). We thus conclude that *POLG* mutations may impair the mitophagy machinery in astrocytes in both a PINK1/Parkin-dependent and independent manner, which includes impairment of autophagy-lysosome pathway.

Our previous studies showed that POLG-mutant astrocytes generated less ATP and significantly higher levels of lactate than controls indicating a shift from OXPHOS to glycolysis (Liang et al., 2020a). Given that AMPK is a key metabolic sensor/regulator and essential to balance glycolysis and mitochondrial metabolism in order to control cellular stress and survival (Kishton et al., 2016), we speculated that this metabolic shift could inhibit the AMPK signaling pathway. This, in turn, would modulate mTOR signaling *via* its upstream activator AKT and the downstream ULK1 signaling leading to downregulation of the mitophagy machinery. To investigate how the AMPK, mTOR, and PI3K/AKT pathway might impact POLG-affected mitophagy, we investigated changes in phosphorylated AMPK and mTOR, as well as the upstream PI3K/AKT proteins. We found, as expected, that POLG-mutant astrocytes showed suppressed AMPK and activated mTOR *via* upstream PI3K/AKT proteins. Similar to our findings, studies have demonstrated that autophagy in PD dopaminergic cells was regulated by the AMPK/mTOR signaling pathway, suggesting that AMPK/mTOR-mediated autophagy may also be a potential therapeutic target for the treatment of PD patients (Ng et al., 2012). Together, our findings support the suggestion that changes in the AMPK/mTOR signaling pathway contribute to the POLG-induced mitophagy dysfunction.

Previously, we reported that POLG-mutant astrocytes exhibited abnormal NAD^+^ metabolism *via* SIRT1-dependent regulation (Liang et al., 2020a). SIRT1 is a highly conserved NAD^+^-dependent protein deacetylase that has emerged as a critical mitophagy regulator (Fang et al., 2014). SIRT1 is also required for AMPK activation (Price et al., 2012) and can induce mitophagy activity *via* regulating AMPK/mTOR/ULK signaling (Mei et al., 2020). Recent work has suggested that supplementation with the NAD^+^ precursor NR can potentially restore NAD^+^ metabolic profiles and improve mitophagy in a ULK1-dependent manner (Fang et al., 2019a). We therefore treated our patient astrocytes with NR to increase SIRT1 activity and investigated the effects on AMPK/mTOR-dependent mitophagy. Despite finding that SIRT1 phosphorylation was increased, NR treatment alone failed to alter AMPK/mTOR-related proteins or rectify the mitophagy defects in POLG-mutant astrocytes.

Studies have also suggested that metformin might be beneficial by maintaining SIRT1 activity during the aging process when NAD^+^ levels decline (Bhansali et al., 2020). Moreover, previous studies showed that metformin upregulated mitophagy markers and enhanced mitophagic flux through the AMPK pathway (Bhansali et al., 2020). Our studies showed that metformin activated AMPK and mTOR, but did not increase SIRT1 activity. Metformin stimulated mitophagy in POLG-mutant astrocytes as evidenced by increased LC3B conversion and p62 expression. Thus, given that metformin and NR appeared to activate different parts of the mitophagy process, we asked whether dual treatment would ameliorate the mitophagy defects in POLG-mutant astrocytes. Our results suggest that this is the case: not only does dual treatment significantly elevate LC3B-II/LC3B-I ratio and p62 level, but it also improves mitophagy and lysosome formation in POLG-mutant astrocytes.

Metformin is known to inhibit complex I, but this is concentration dependent: at higher concentrations it inhibits, while at lower concentrations it appears to improve mitochondrial respiratory chain function (Wang et al., 2019). In our study, we showed an increased complex I expression with NR and lower concentration metformin co-treatment. Mitochondrial biogenesis and mitochondrial functional properties were also increased in patient astrocytes following exposure to the co-treatment. In an earlier study (Liang et al., 2020b), we showed that *POLG* mutations lead to mtDNA depletion and loss of complexes I and IV in astrocytes. These changes also led to ATP depletion and abnormal NAD^+^ metabolism. Our current studies demonstrate that POLG-mutant astrocytes also have impaired mitophagy (Figure 8J). It is possible to hypothesize, therefore, that the neuronal loss associated with *POLG* mutations might occur *via* two mechanisms; first, loss of normal mitochondrial function and disrupted mitophagy impairs normal astrocytic support function, and second, the mitochondrial defects cause astrocytes to become reactive and neurotoxic. Our work suggests that treatment of NR and metformin potentially improves both the mitochondrial functional deficit and mitophagy offering a way of rescuing the disease phenotype in patients with *POLG* mutations.

## Conclusion

Overall, these results identify that *POLG* mutations lead to accumulation of damaged mitochondria which can further impart the mitophagy machinery by regulating the PINK1/Parkin and AKT/mTOR/AMPK/ULK1 signaling pathway in astrocytes. Further and most notably, we reveal that NR and metformin rescued mitophagy defect in the POLG-mutant astrocytes, providing new possibilities for the treatment of these disorders by using mitophagy-enhancing reagents.

## Data Availability Statement

The datasets presented in this study can be found in online repositories. The names of the repository/repositories and accession number(s) can be found in the article/Supplementary Material.

## Ethics Statement

The project was approved by the Western Norway Committee for Ethics in Health Research (REK nr. 2012/919). The patients/participants provided their written informed consent to participate in this study.

## Author Contributions

KL and LB contributed to the conceptualization. AC and KL contributed to the methodology. AC, KL, CK, YH, and AK contributed to the investigation. AC, KL, and LB contributed to writing the original draft. AC, KL, CK, YH, AK, EF, GS, JW, XL, and LB contributed to the writing – review and editing. LB, GS, EF, and AC contributed to the funding acquisition. KL, GS, and LB contributed to the resources. KL, LB, and XL contributed to the supervision. All authors agreed to the authorships.

## Conflict of Interest

EF has a CRADA arrangement with ChromaDex and is a consultant to Aladdin Healthcare Technologies and the Vancouver Dementia Prevention Centre. The remaining authors declare that the research was conducted in the absence of any commercial or financial relationships that could be construed as a potential conflict of interest.

## Publisher’s Note

All claims expressed in this article are solely those of the authors and do not necessarily represent those of their affiliated organizations, or those of the publisher, the editors and the reviewers. Any product that may be evaluated in this article, or claim that may be made by its manufacturer, is not guaranteed or endorsed by the publisher.

## Footnotes

1
https://imagej.nih.gov/ij

2
https://www.kegg.jp

3
https://en.wikipedia.org/wiki/Hypergeometric_distribution
