# Supplementary material for: Nicotinamide Riboside and Metformin Ameliorate Mitophagy Defect in Induced Pluripotent Stem Cell-Derived Astrocytes With POLG Mutations
Source: Front Cell Dev Biol. 2021 Sep 24;9:737304. doi: 10.3389/fcell.2021.737304 (PMC8497894; doi:10.3389/fcell.2021.737304)
Supplement: Supplementary file 1 [file Data_Sheet_1.pdf]

Table S1. The list of the sample's information in this study.

Subject	Source	Mutation	Age	Gender
**HNA**	Lonza (CC-2565)	No	Fetal	Female
**ESC 1 astrocyte**	hESC line 360	No	Embro (6 days)	Female
**ESC 2 astrocyte**	H1	No	Embro (6 days)	Male
**Control 1 astrocyte**	Detroit 551	No	Fetal	Female
**Control 2 astrocyte**	AG05836B	No	44 years	Female
**POLG patient 1 astrocyte**	WS5A patient	Homozygous for c.2243G>C; p.W748S	44 years	Female
**POLG patient 2 astrocyte**	CP2A patient	Compound heterozygous for c.1399G>A/c.2243G>C; p.A467T/W748S	49 years	Male

Table S2. Primary antibodies used in this study.

Antibodies	Host	Company	Cat. no.	Application
		**Primary antibodies**		
**GFAP**	Chicken	Abcam	ab4674	ICC/FC
**DCX**	Rabbit	Thermo Fisher Scientific	PA5-17428	ICC
**S100β**	Rabbit	Abcam	ab196442	ICC/FC
**EAAT-1**	Rabbit	Abcam	ab416	ICC/FC
**CD44**	Rabbit	Abcam	ab189524	ICC/FC
**SOX2**	Rabbit	Abcam	ab97959	ICC
**NESTIN**	Mouse	Abcam	ab22035	ICC
**NANOG**	Rabbit	Abcam	ab80892	ICC
**PAX6**	Rabbit	Abcam	ab5790	ICC
**OCT4**	Rabbit	Abcam	ab19857	ICC
**GS**	Mouse	Abcam	ab64613	ICC/FC
**p-AMPK (T183 + T172)**	Rabbit	Abcam	ab133448	WB
**AMPK**	Rabbit	Cell Signaling Technology	2532S	WB
**TOMM20**	Mouse	Abcam	ab56783	ICC/WB
**MFN1**	Rabbit	Cell Signaling Technology	14739	WB
**OPA1**	Rabbit	Cell Signaling Technology	80471	WB
**p-DRP1 (Ser616)**	Rabbit	Cell Signaling Technology	4494	WB
**DRP1**	Rabbit	Cell Signaling Technology	8570	WB
**MFF**	Rabbit	Cell Signaling Technology	84580	WB
**PGC-1α**	Mouse	Abcam	ab77210	WB
**NDUFB10**	Mouse	Abcam	ab196019	WB
**p62**	Rabbit	Abcam	ab109012	WB
**LC3B**	Rabbit	Abcam	ab51520	WB
**LAMP2A**	Rabbit	Abcam	ab125068	WB
**mTOR**	Rabbit	Cell Signaling Technology	2983T	WB
**p-mTOR**	Rabbit	Cell Signaling Technology	2971S	WB
**p-AKT**	Rabbit	Cell Signaling Technology	4060T	WB
**AKT**	Rabbit	Cell Signaling Technology	9272S	WB
**p-ULK1**	Rabbit	Cell Signaling Technology	6888S	WB
**ULK1**	Rabbit	Cell Signaling Technology	8054p-T	WB
**p-SIRT1**	Rabbit	Cell Signaling Technology	2314S	WB
**SIRT1**	Rabbit	Cell Signaling Technology	9475T	WB
**PGC-1β**	Rabbit	Abcam	ab176328	WB
**PINK1**	Rabbit	Cell Signaling Technology	6946T	WB
**Parkin**	Mouse	Cell Signaling Technology	4211T	WB
**GAPDH**	Rabbit	Abcam	ab9485	WB

ICC: Immunofluorimetric cytochemistry staining; FC: Flow cytometry; WB: Western blotting

Table S3. Secondary antibodies used in this study.

Antibodies	Host	Company	Cat. no.	Application
		**Secondary antibodies**		
**Alexa Flour® 488 Goat anti-Rabbit**	Rabbit	Thermo Fisher Scientific	A11008	ICC/FC
**Alexa Flour®594 Goat anti-Mouse**	Mouse	Thermo Fisher Scientific	A11005	ICC/FC
**Alexa Flour®594 Goat anti-Chicken**	Chicken	Thermo Fisher Scientific	A11042	ICC/FC
**Swine anti-Rabbit Immunoglobulins/HRP**	Rabbit	Dako	P0217	WB
**Goat anti-Mouse IgG (H+L) Secondary Antibody, HRP**	Mouse	Thermo Fisher Scientific	62-6520	WB

ICC: Immunofluorimetric cytochemistry staining; FC: Flow cytometry; WB: Western blotting, HRP: Horseradish peroxidase secondary

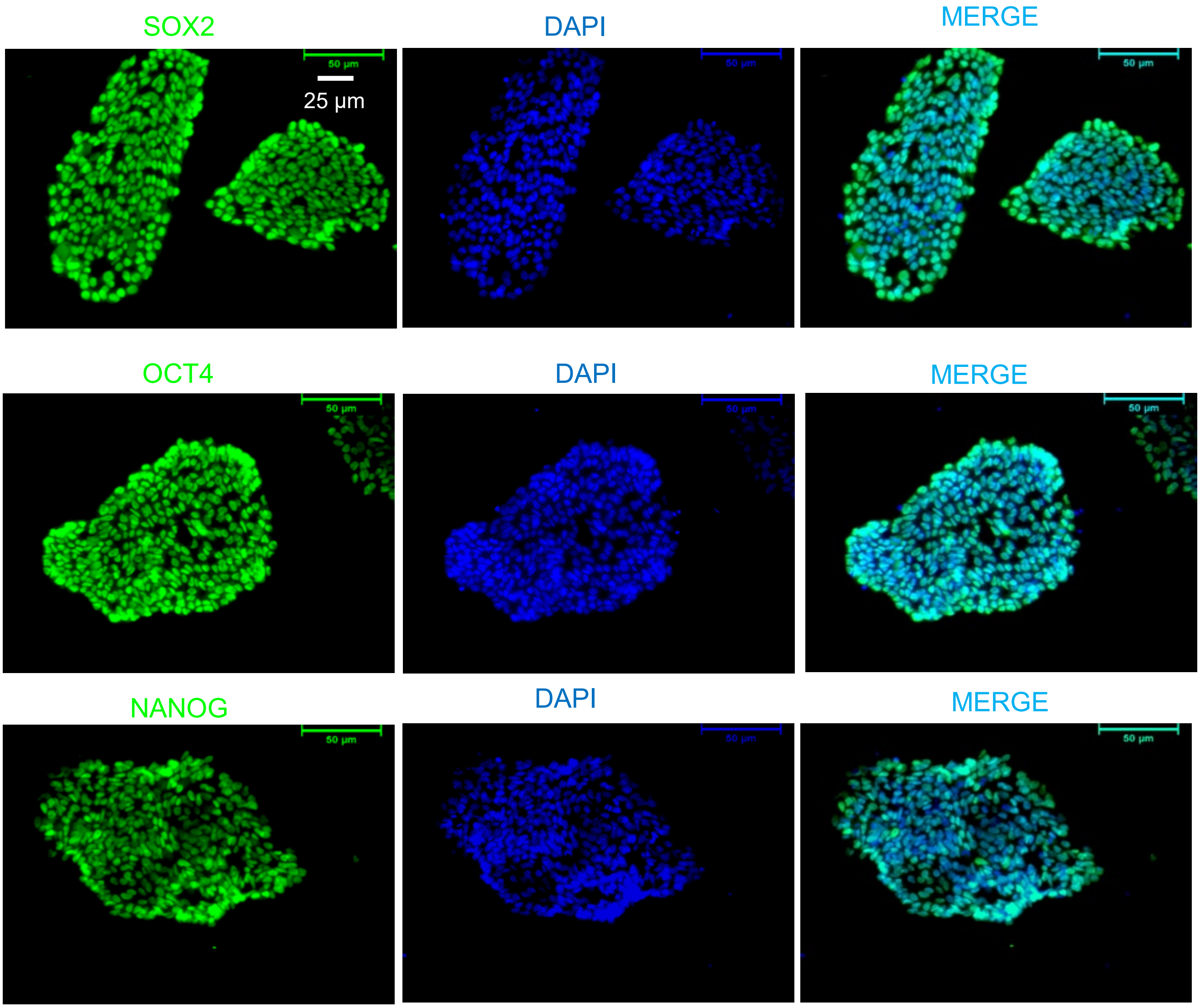

Figure S1: Representative confocal images by immunofluorescence staining of pluripotent markers SOX2, OCT4 and NANOG (green) in iPSC clones. Nuclei are stained with DAPI (blue). Scale bar is 25 µm.

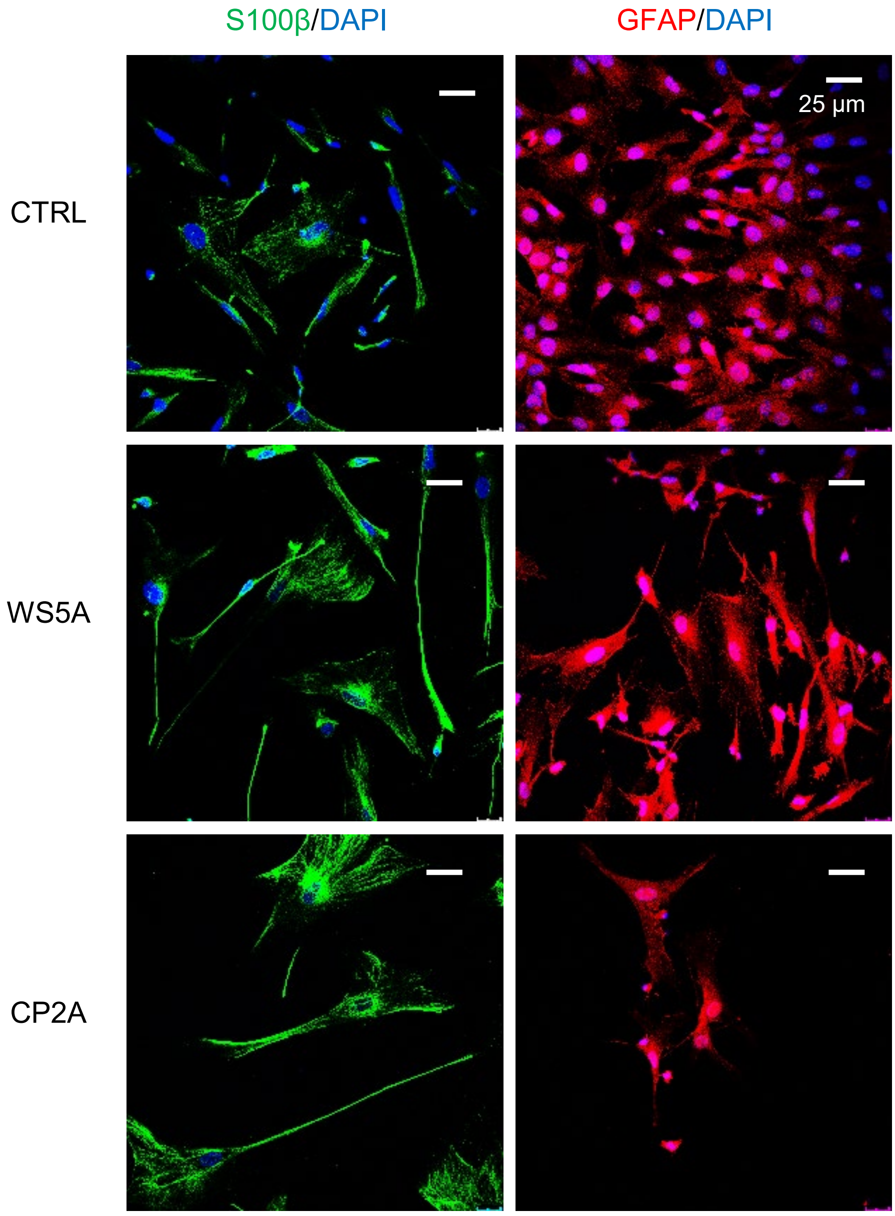

Figure S2: Representative confocal images of immunostaining for GFAP and S100β in iPSC-derived astrocytes from control and patient lines. Nuclei are stained with DAPI (blue). Scale bar is 25 µm.

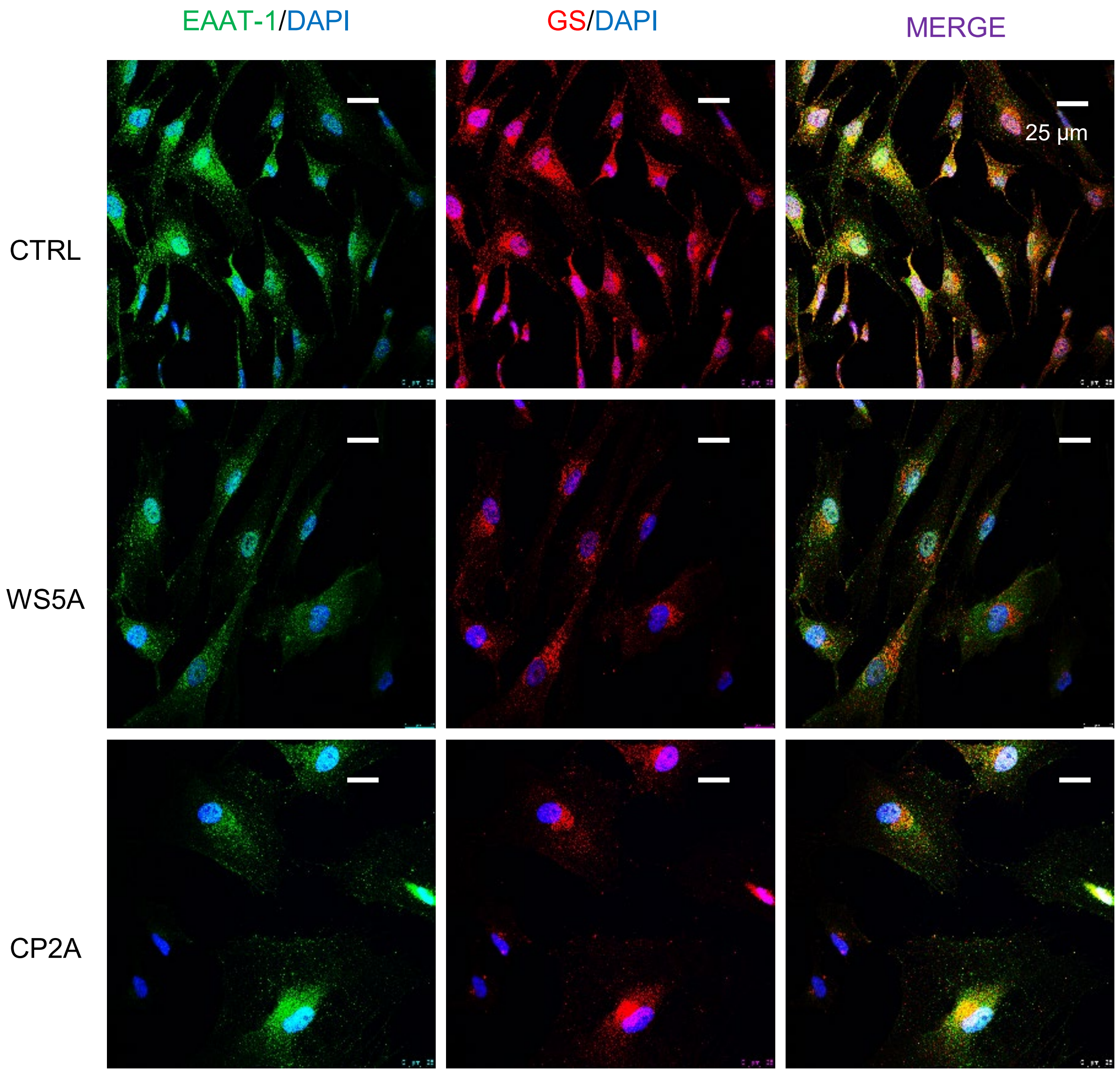

Figure S3: Representative confocal images of immunostaining for EAAT-1 and GS in iPSC-derived astrocytes from control and patient lines. Nuclei are stained with DAPI (blue). Scale bar is 25 µm.

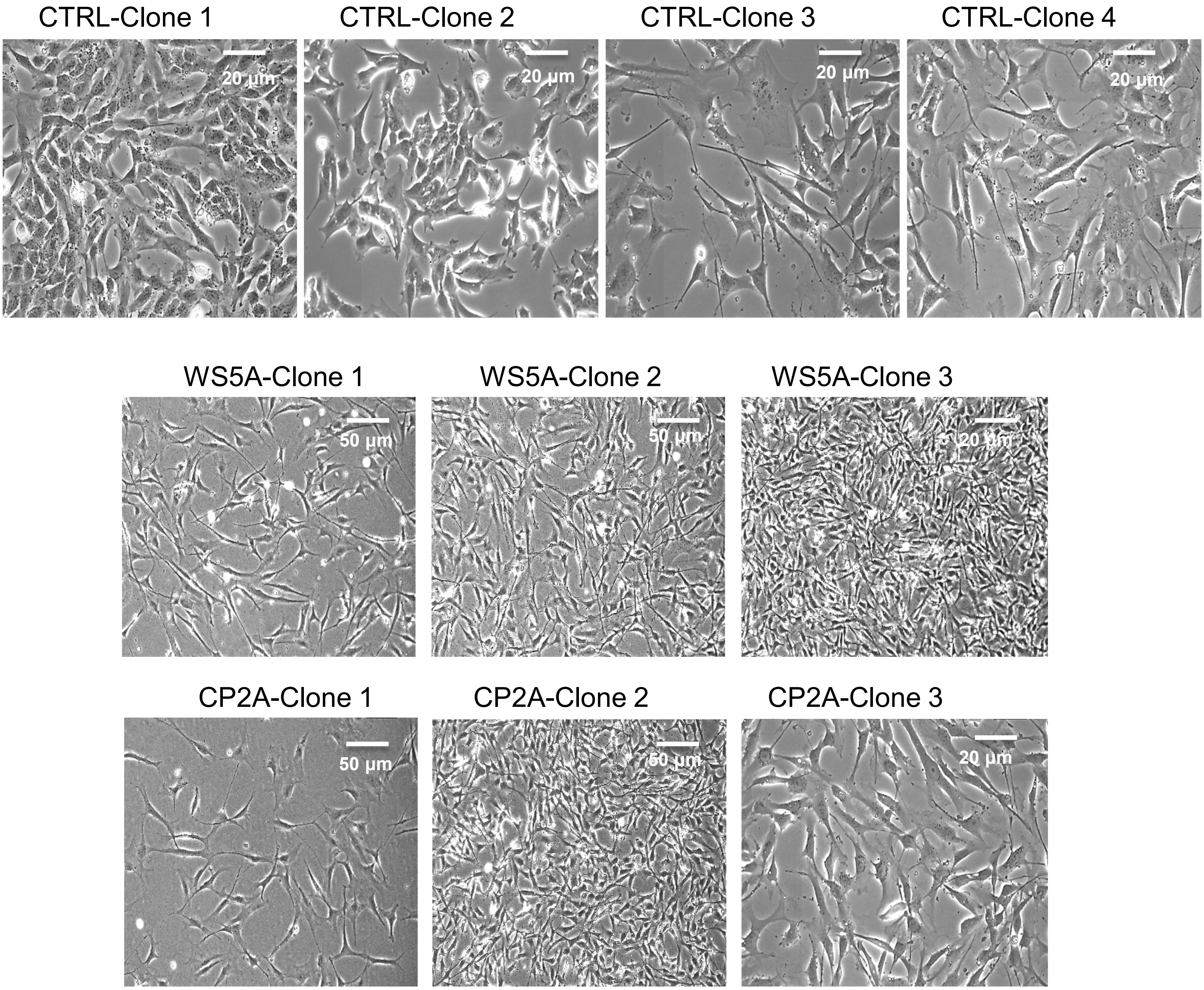

Figure S4. Phase-contrast images of iPSC-derived astrocytes. Scale bar is 50 µm or 20 µm.

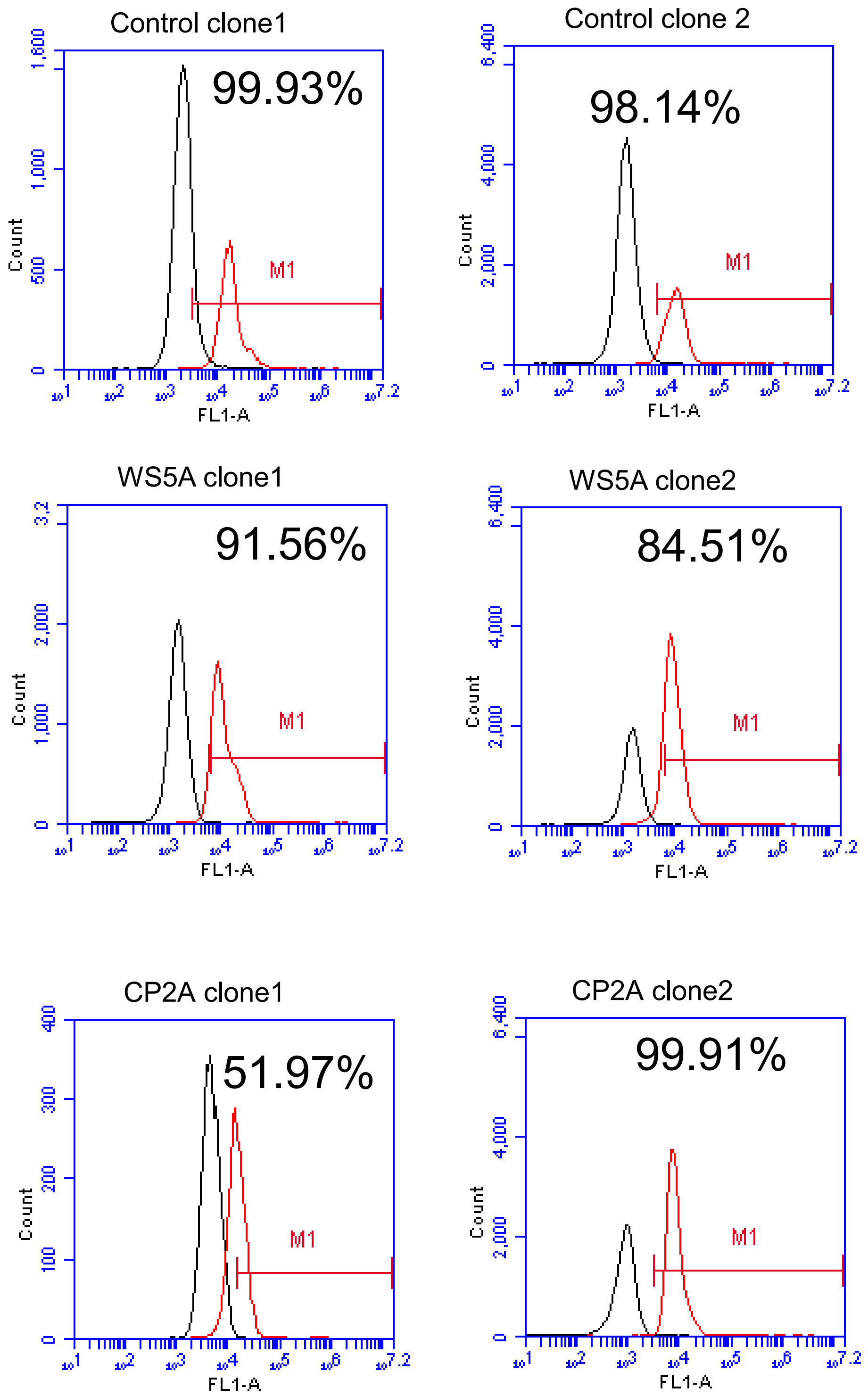

Figure S5. The percentages of positive cells stained with GFAP using flow cytometry.

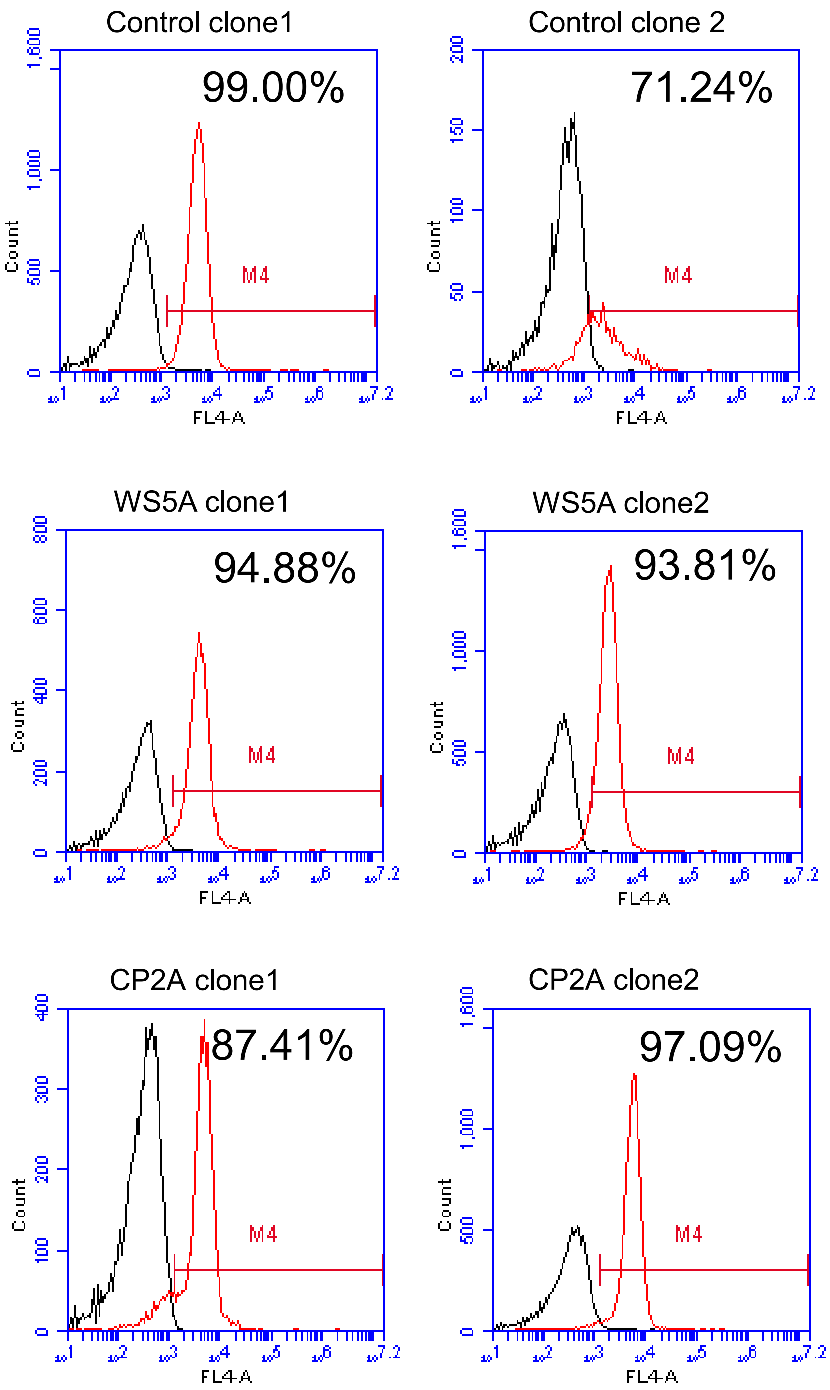

Figure S6. The percentages of positive cells stained with S100β using flow cytometry.

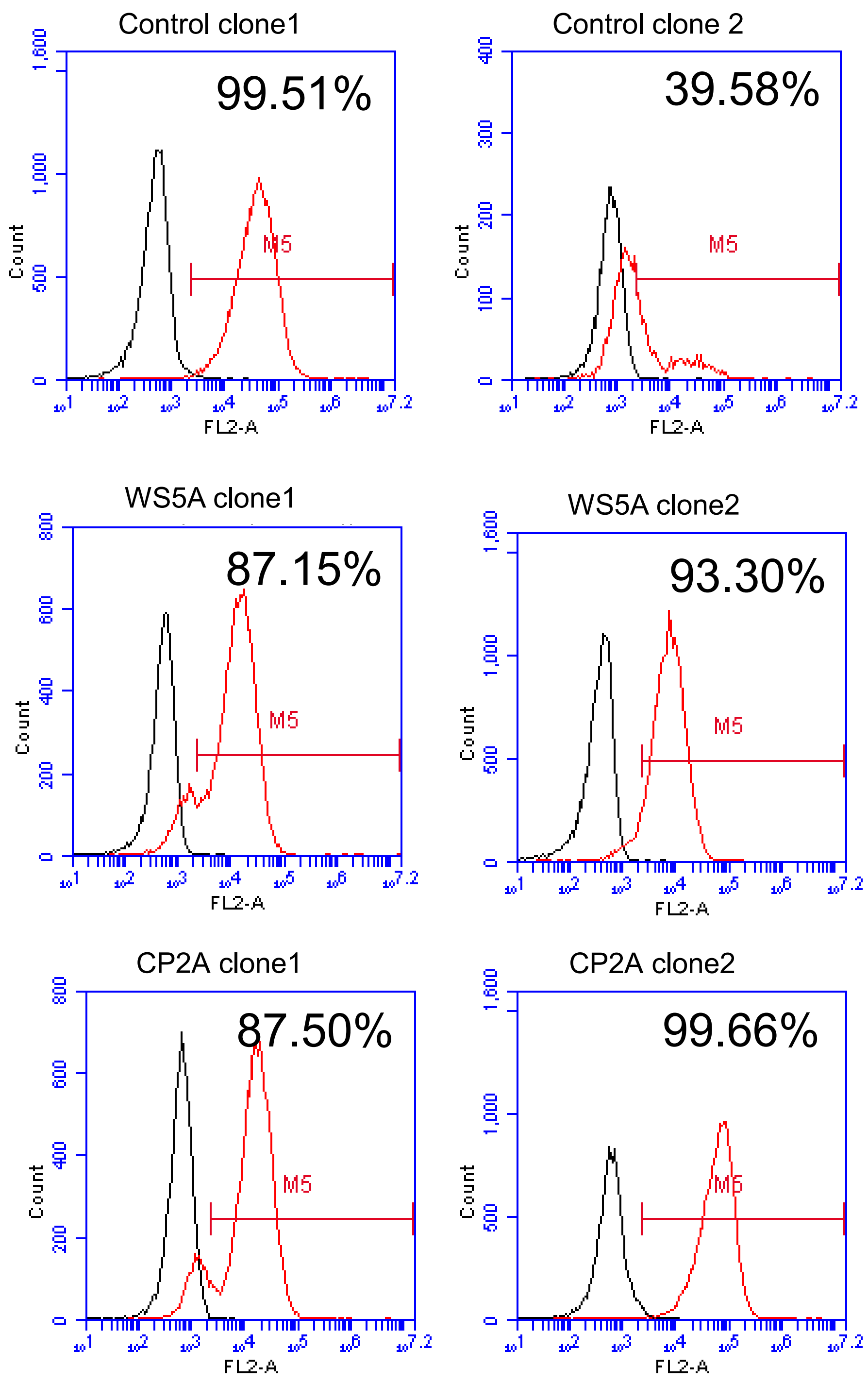

Figure S7. The percentages of positive cells staining with CD44 using flow cytometry.

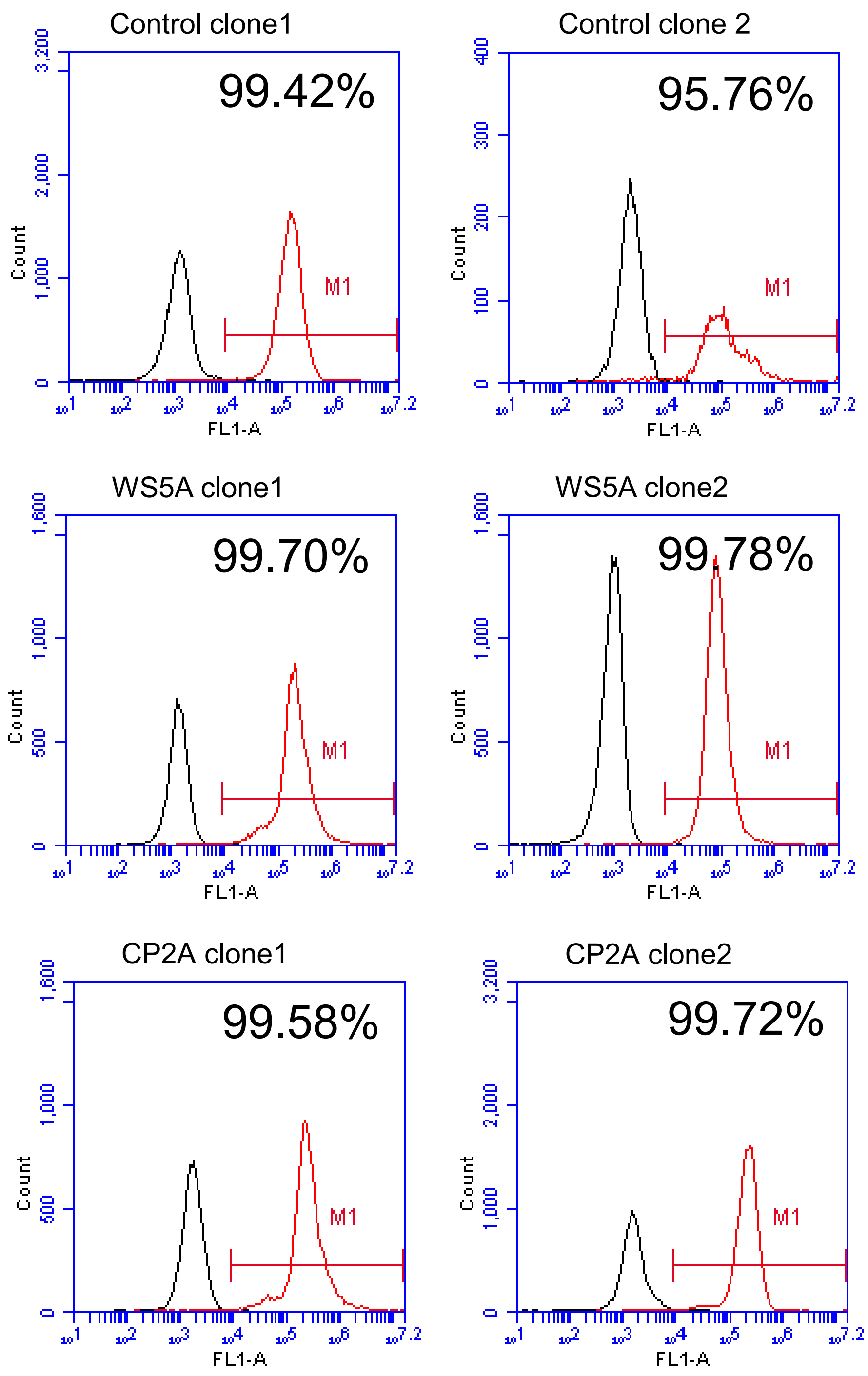

Figure S8. The percentages of positive cells staining with EAAT-1 using flow cytometry.

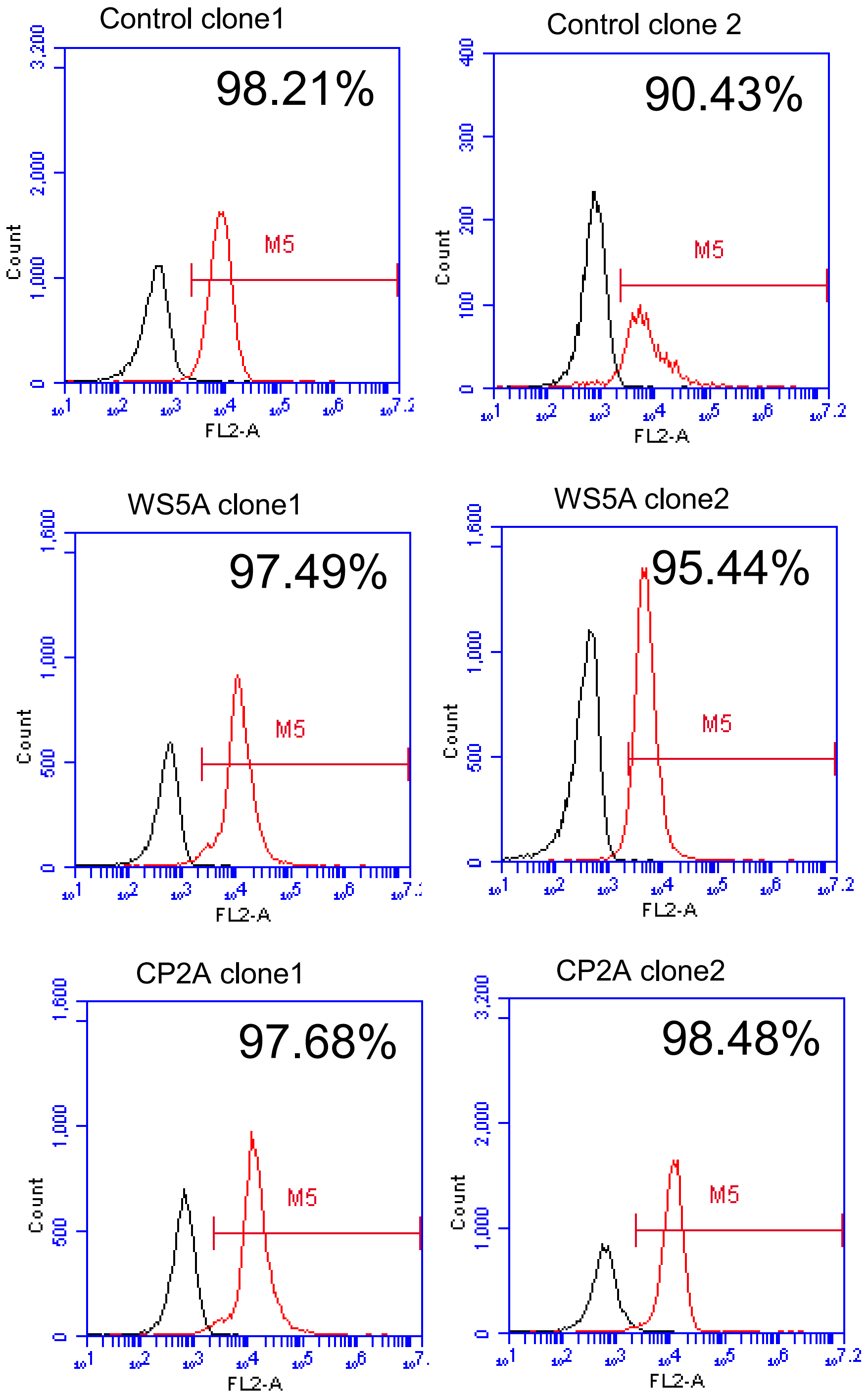

Figure S9. The percentages of positive cells staining with GS using flow cytometry.

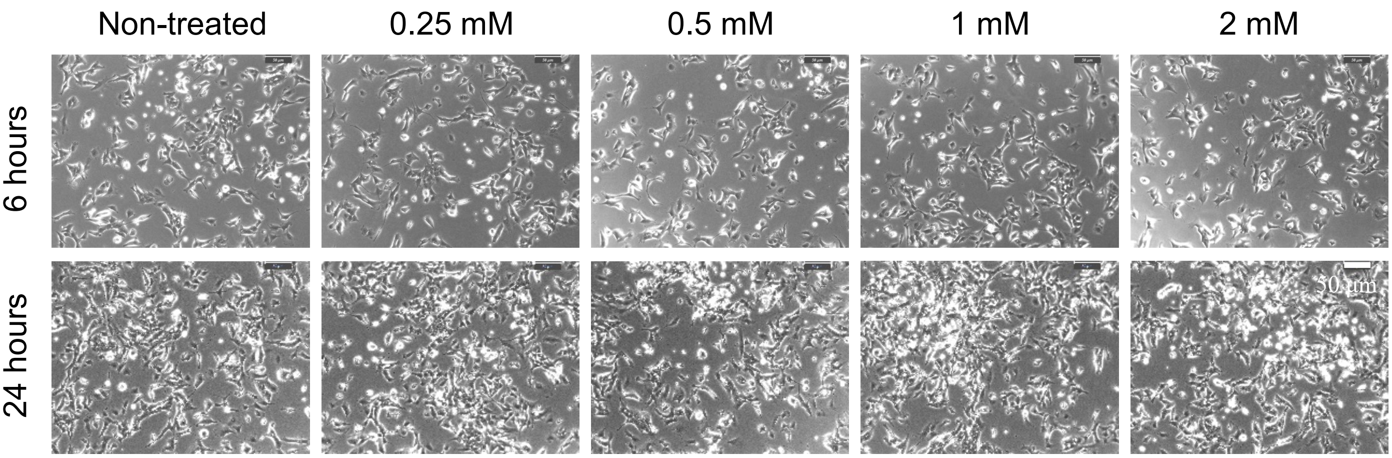

Figure S10. POLG astrocytes were treated with different concentrations of NR (0 mM, 0.25 mM, 0.5 mM, 1 mM, 2 mM). Scale bar is 50 um.

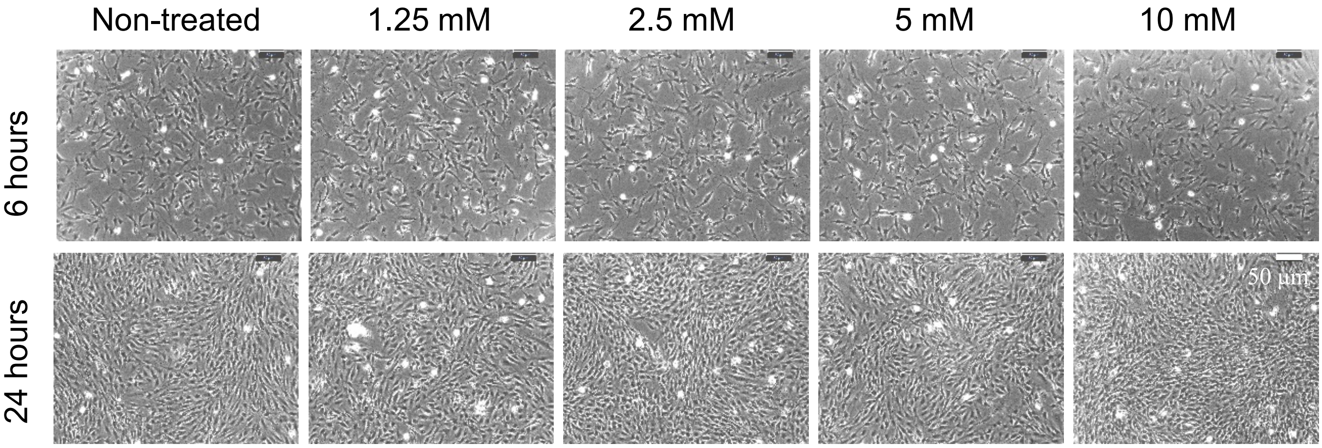

Figure S11. POLG astrocytes were treated different concentrations of metformin (0 mM, 1.25 m M, 2.5 mM, 5 mM, 10 mM). Scale bar is 50 um.

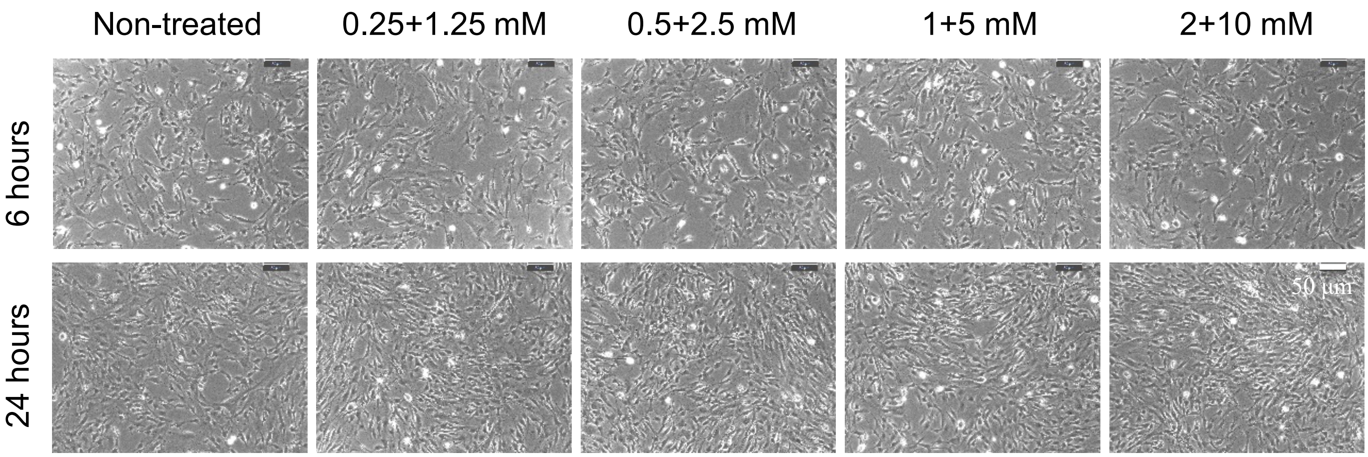

Figure S12. POLG astrocytes were treated with different concentrations of combining NR and m etformin (0 mM, NR 0.25 mM + metformin 1.25 mM, NR 0.5 mM + metformin 2.5 mM, NR 1 m M + metformin 5 mM, NR 2 mM + metformin 10 mM). Scale bar is 50 um.

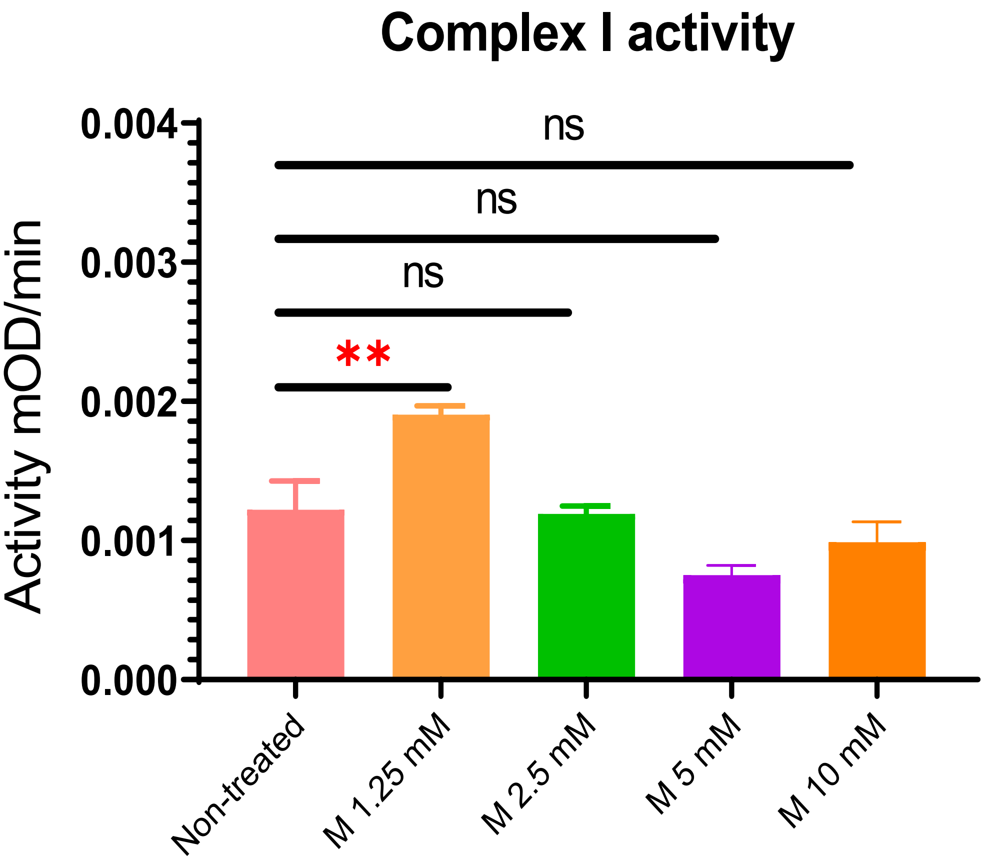

Figure S13. Measurement of the complex I enzyme activity in POLG (WS5A) astrocytes treated with different concentrations of metformin (Non-treated, metformin 1.25 mM, metformin 2.5 mM, metformin 5 mM, metformin 10 mM)).

Data information: The data in the figure represent 1 clone from WS5A. Mann-Whitney U test w as used. Significance is calculated by compared to non-treated cells and is denoted for P value s of less than 0.05. ** P<0.01; ns: not significant.
