# Supplementary material for: Nicotinamide Riboside and Metformin Ameliorate Mitophagy Defect in Induced Pluripotent Stem Cell-Derived Astrocytes With POLG Mutations
Source: Front Cell Dev Biol. 2021 Sep 24;9:737304. doi: 10.3389/fcell.2021.737304 (PMC8497894; doi:10.3389/fcell.2021.737304)
Supplement: Supplementary file 2 [file Data_Sheet_2.pdf]

Figure 1A

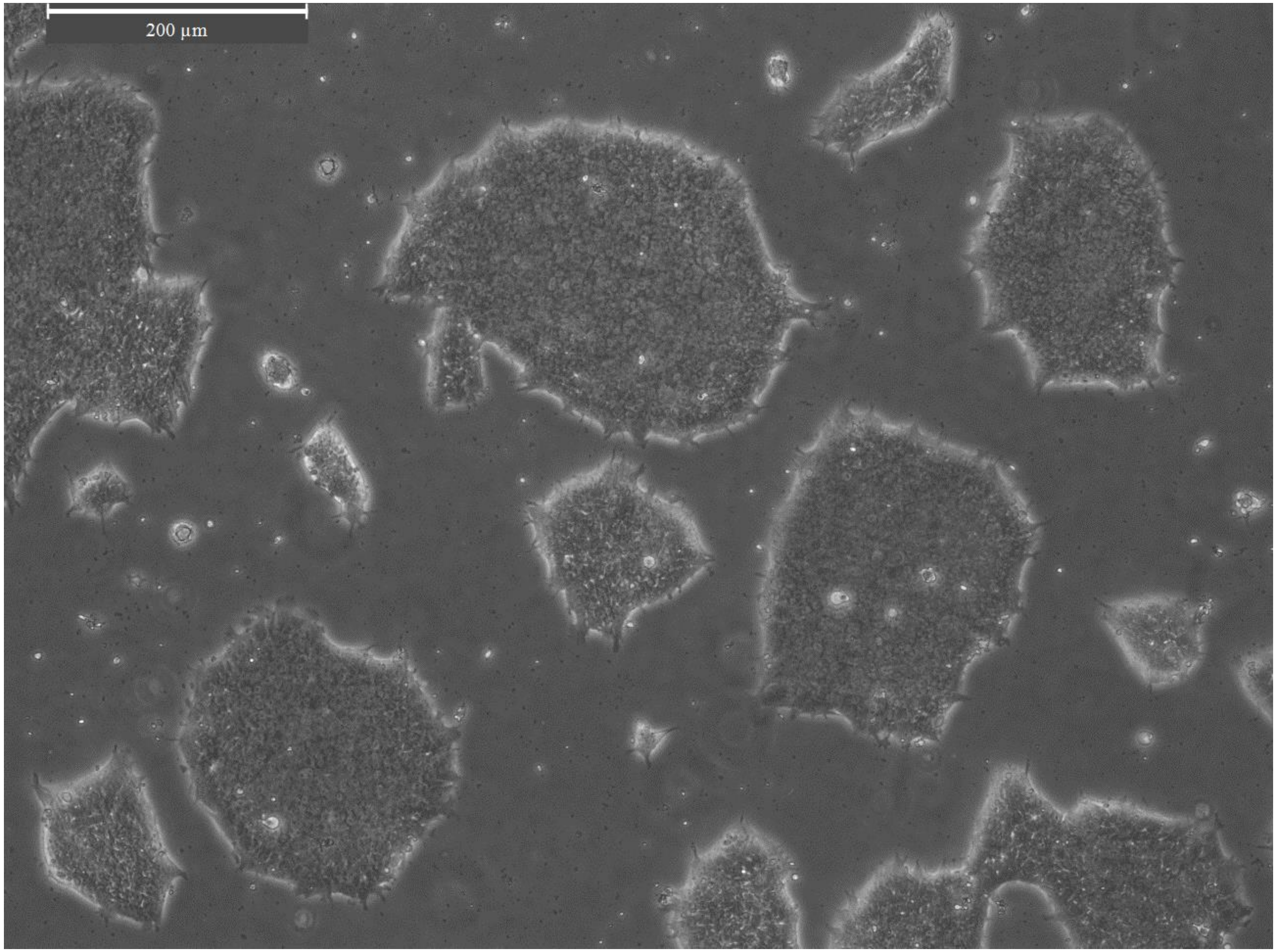

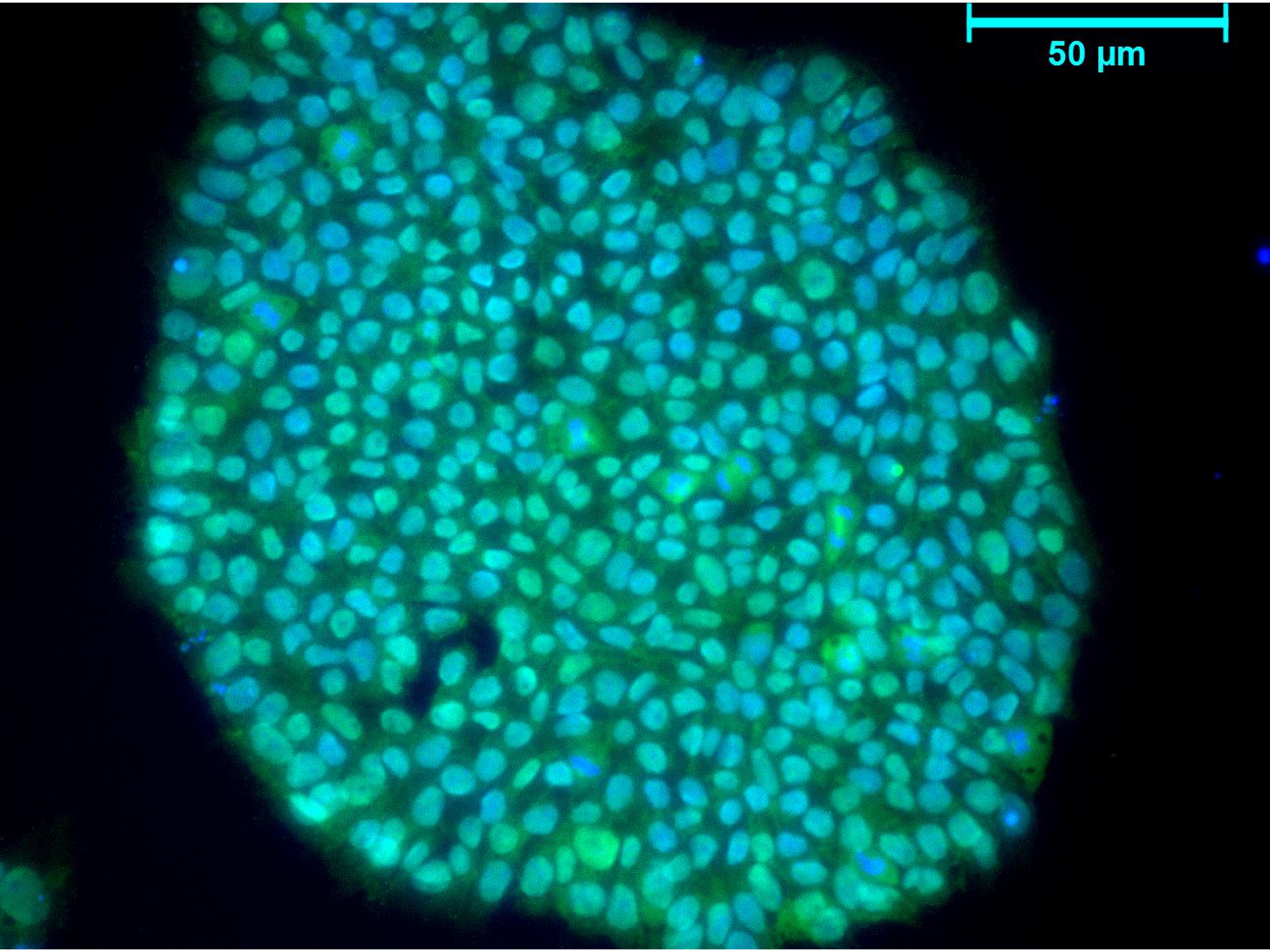
50 µm

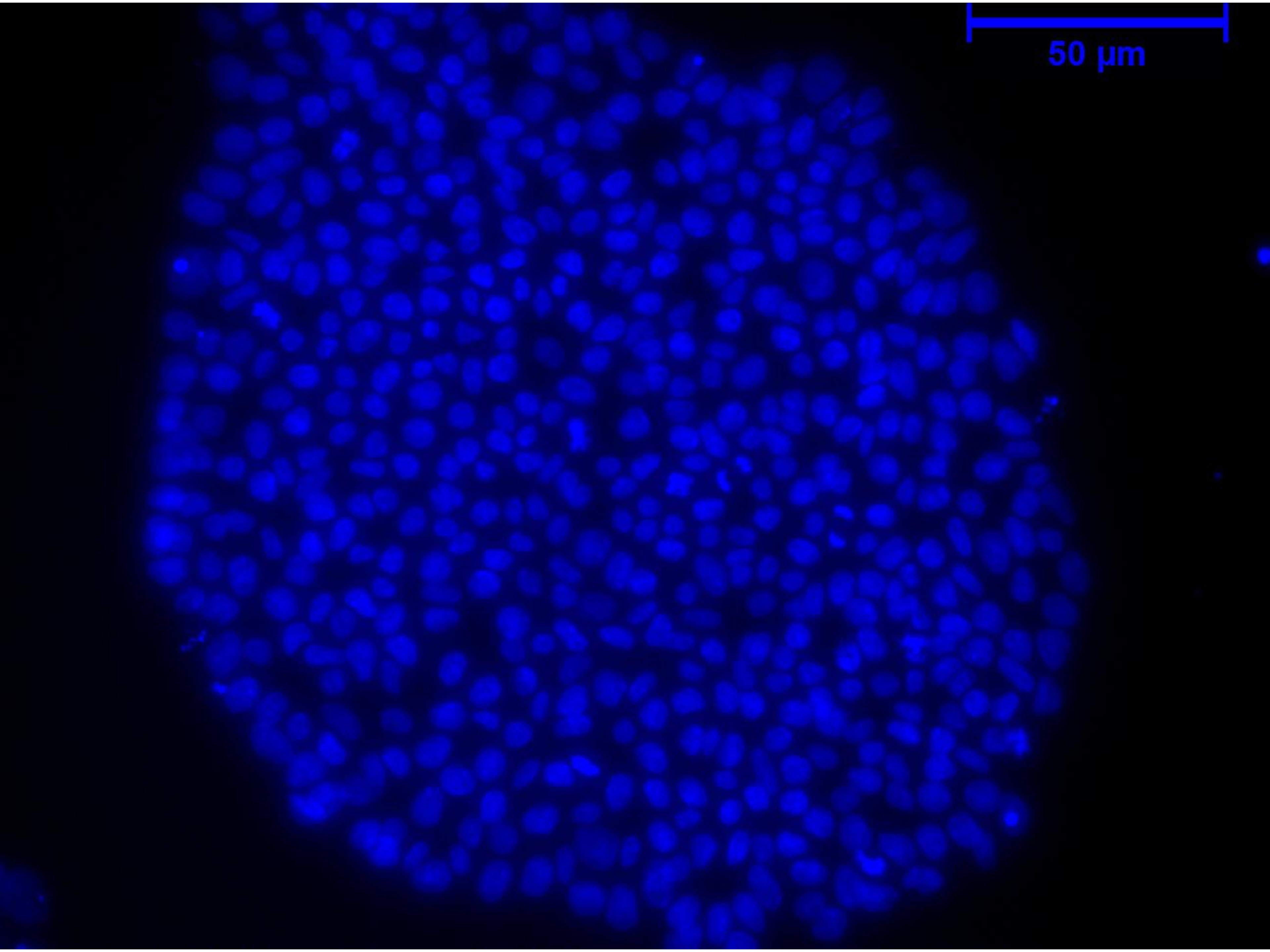
50 µm

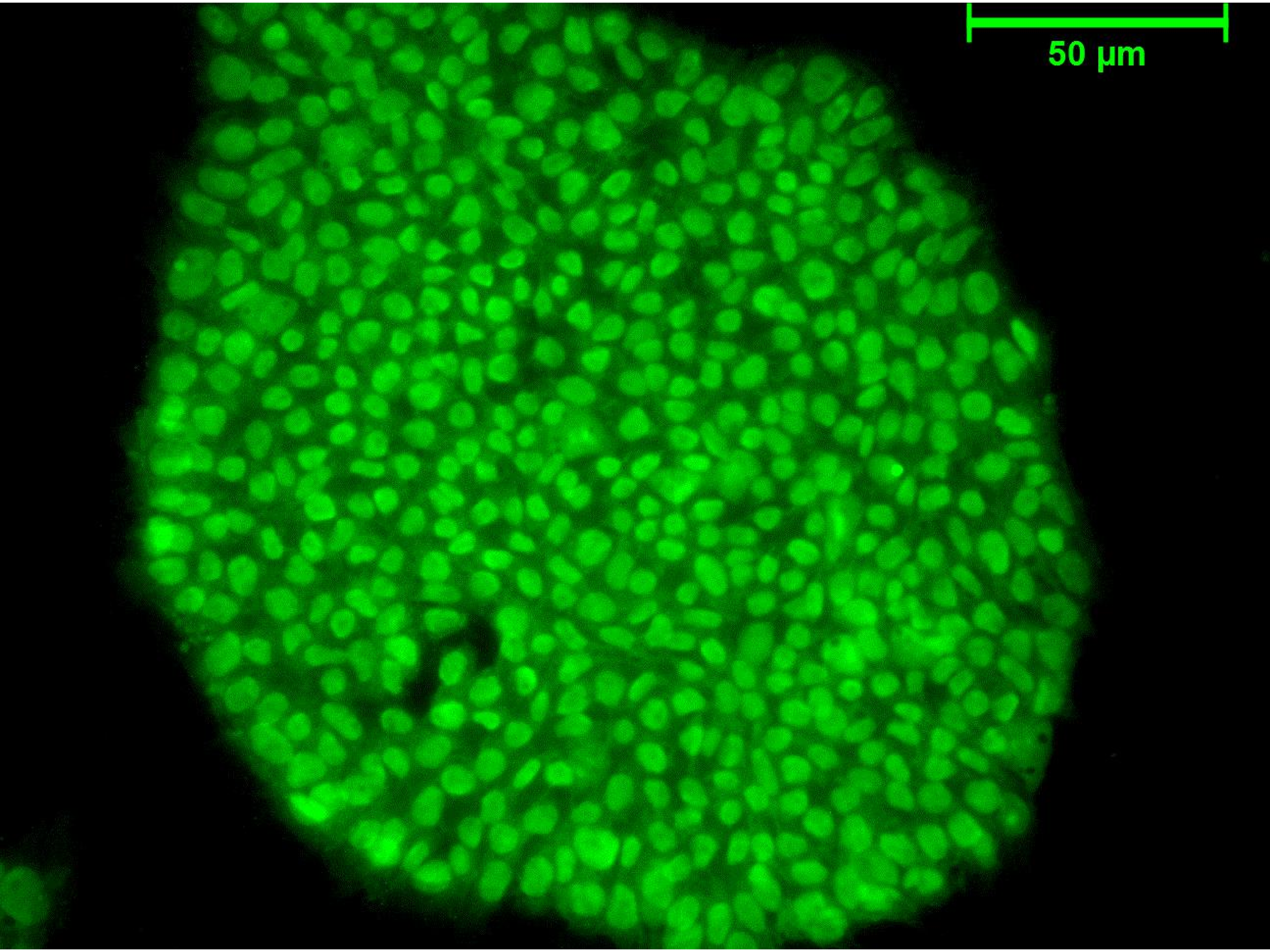
50 µm

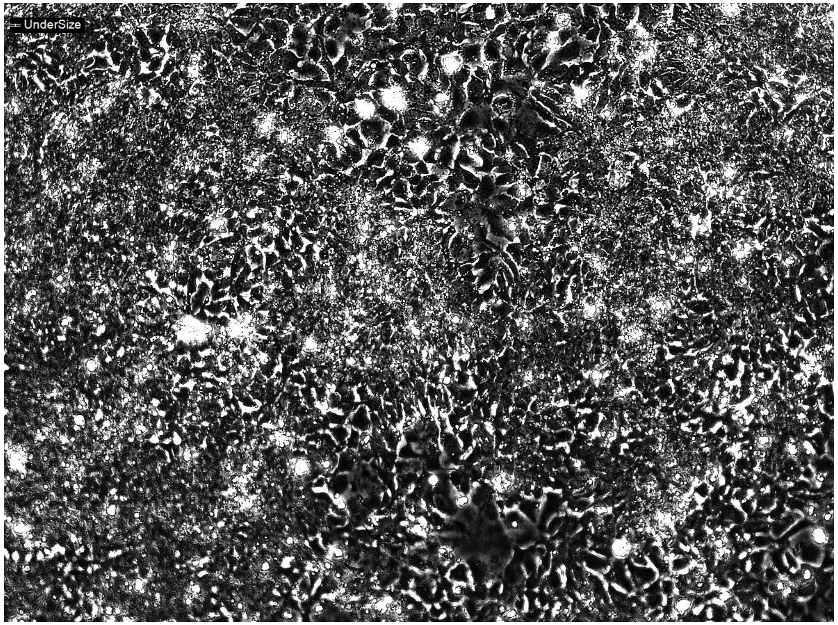
UnderSize

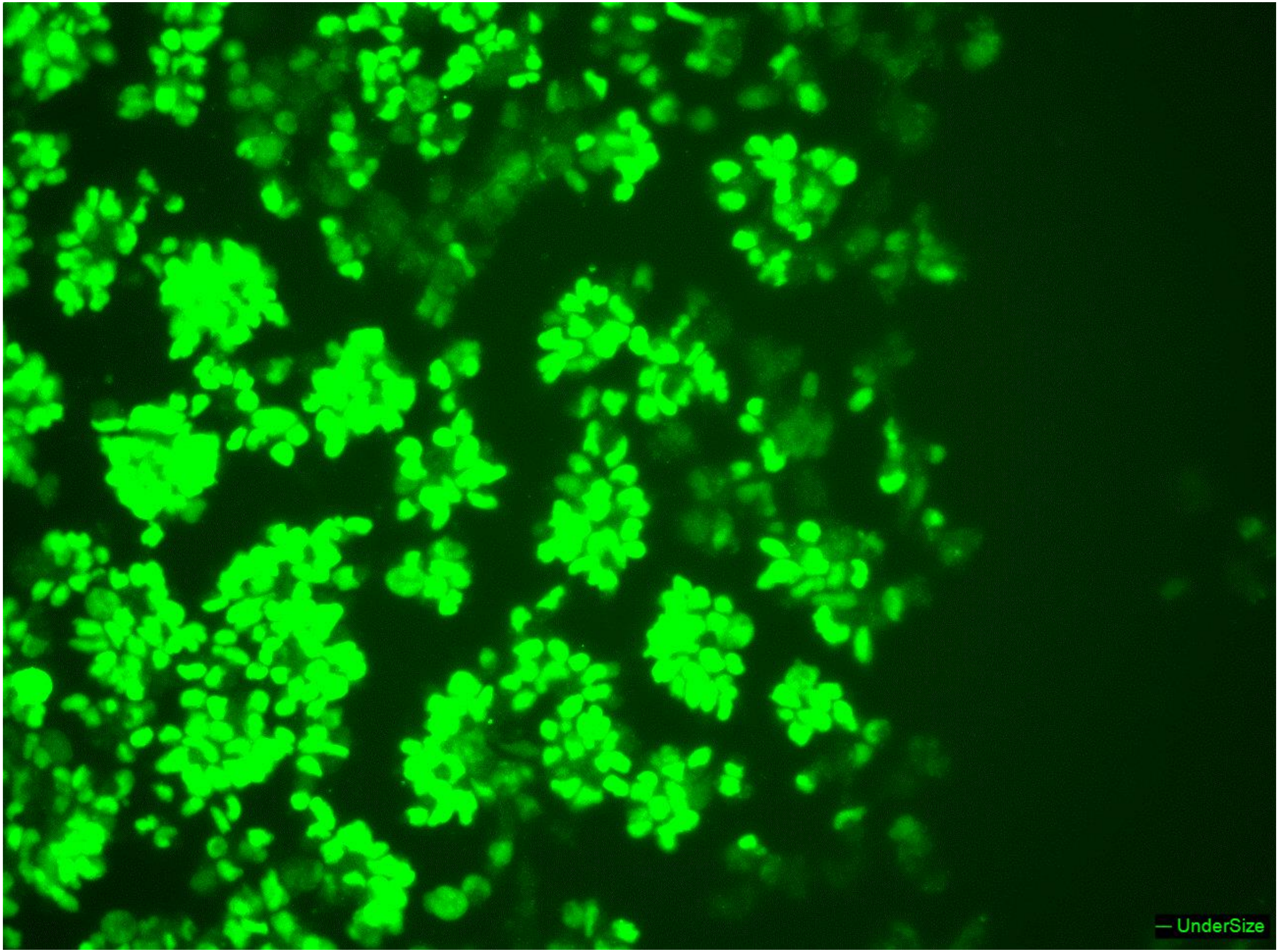
UnderSize

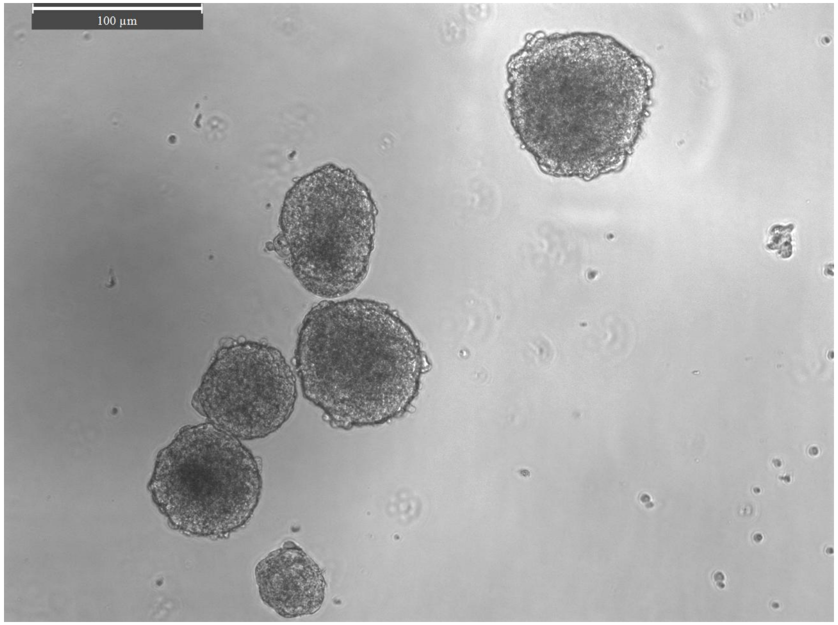
100 µm

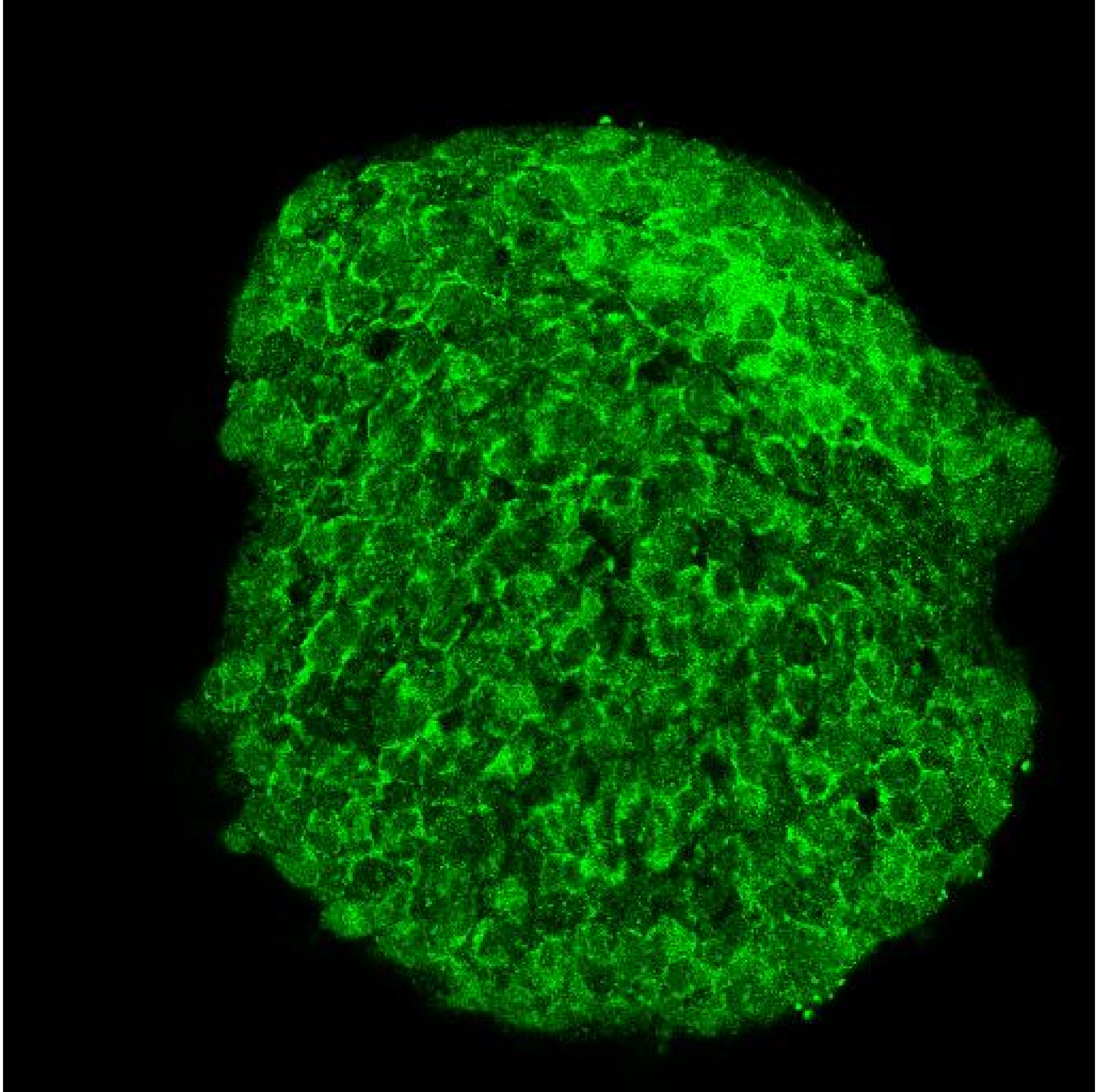

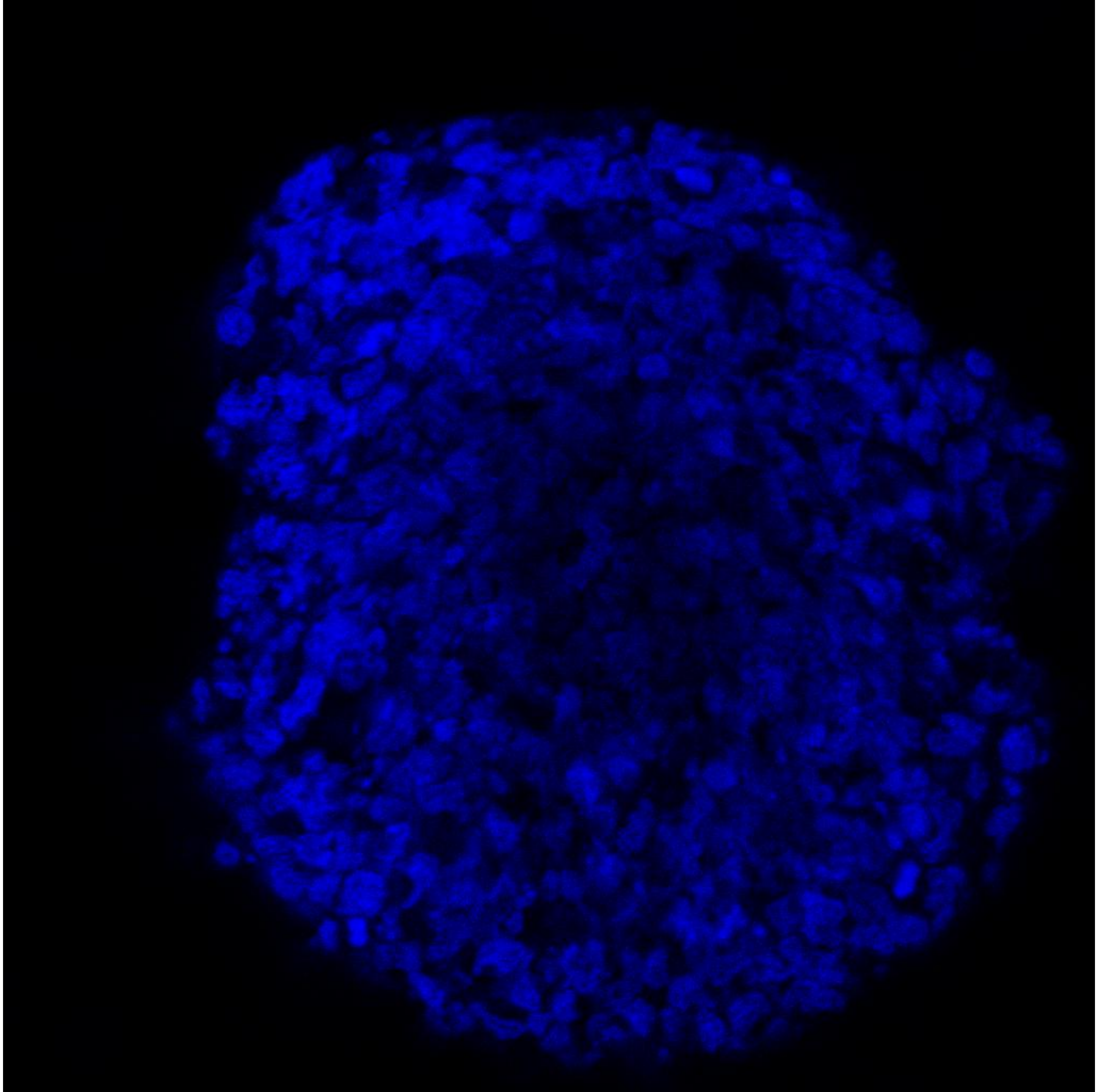

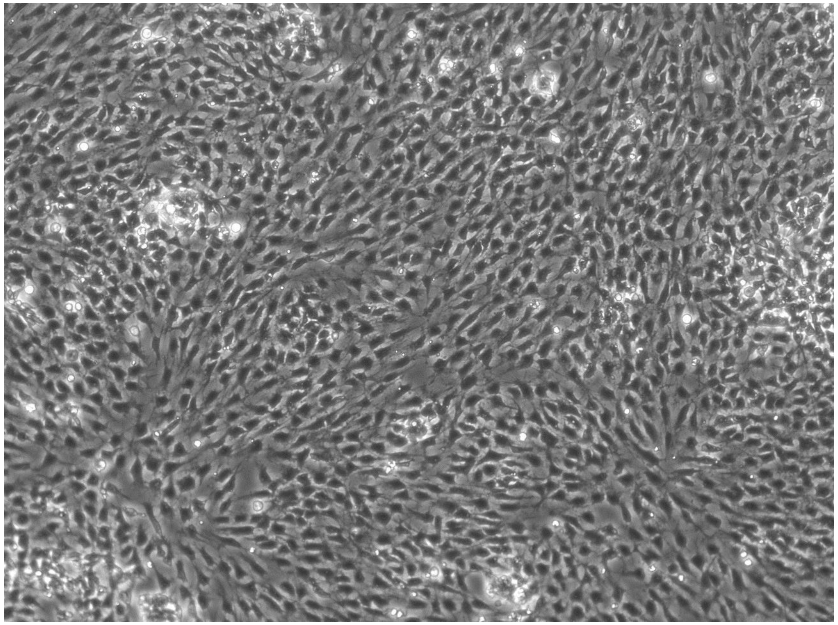

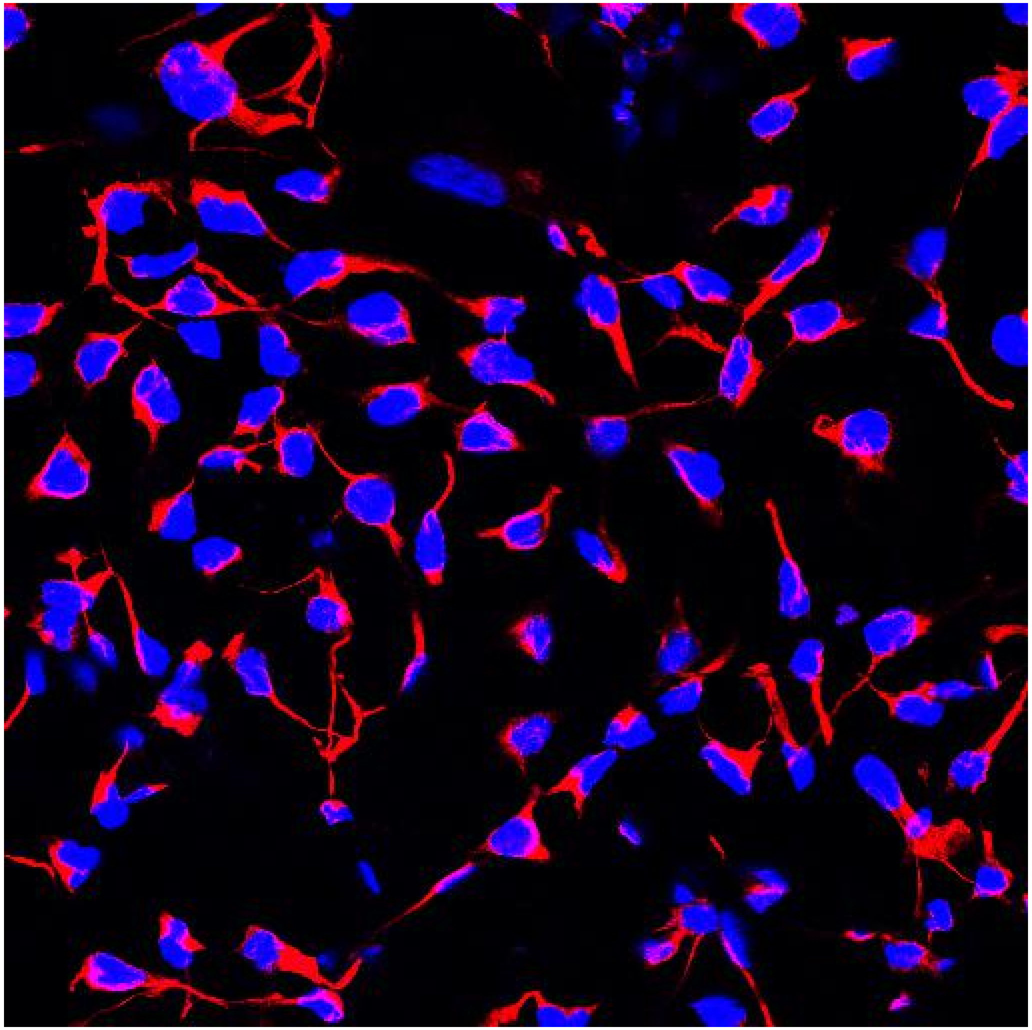

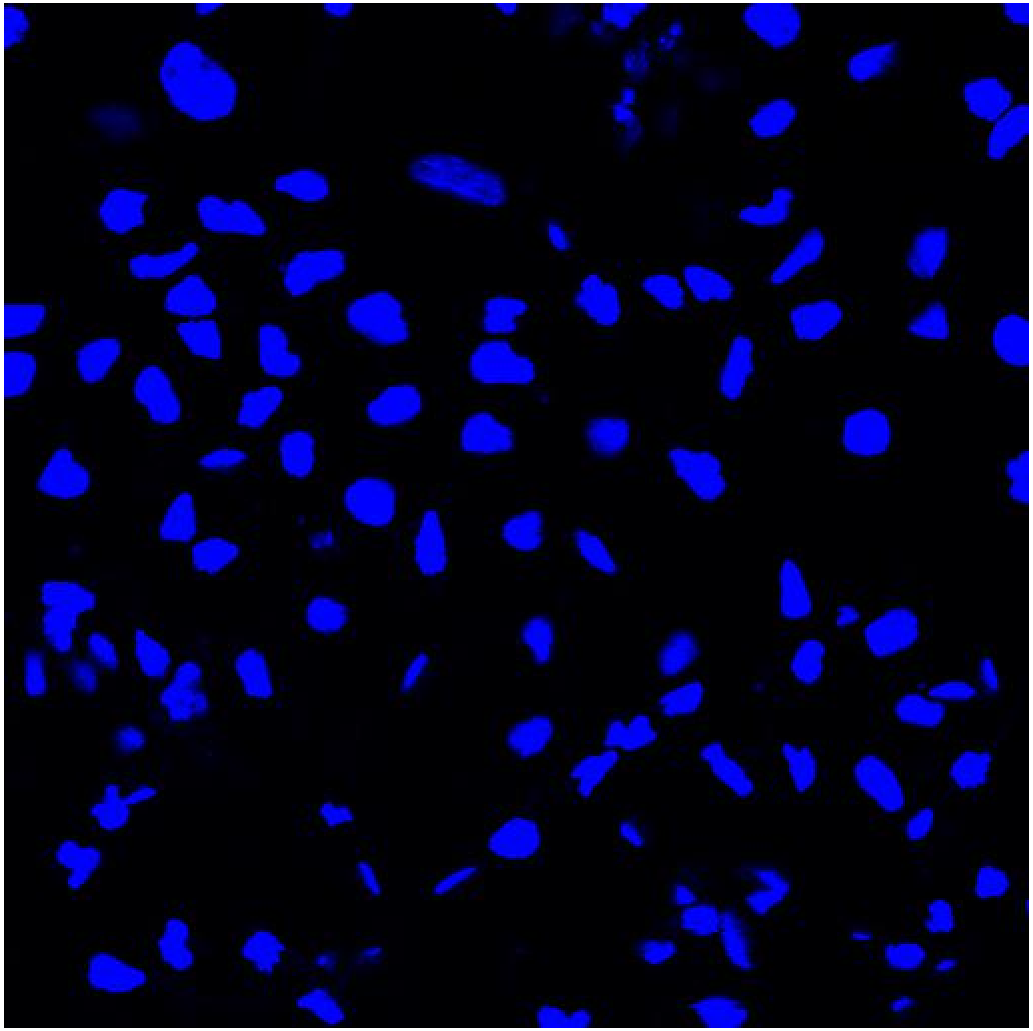

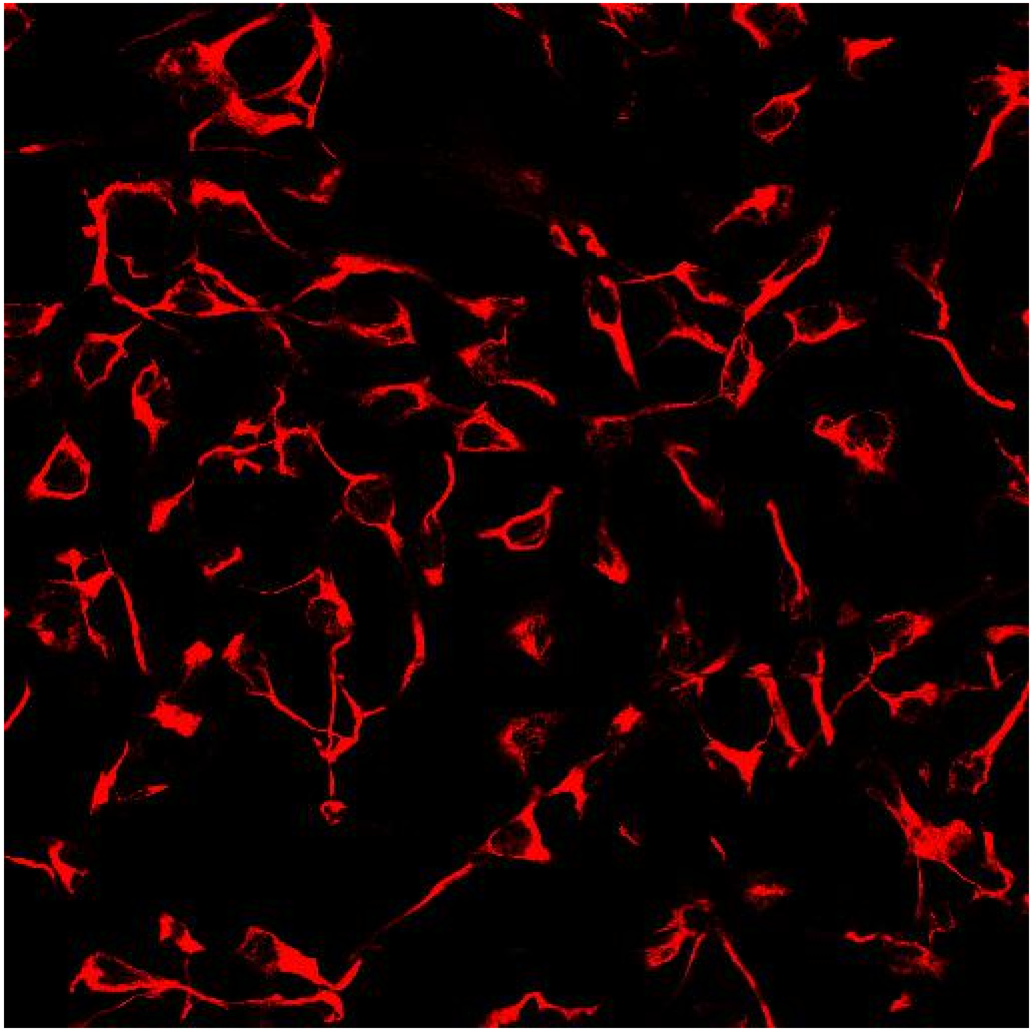

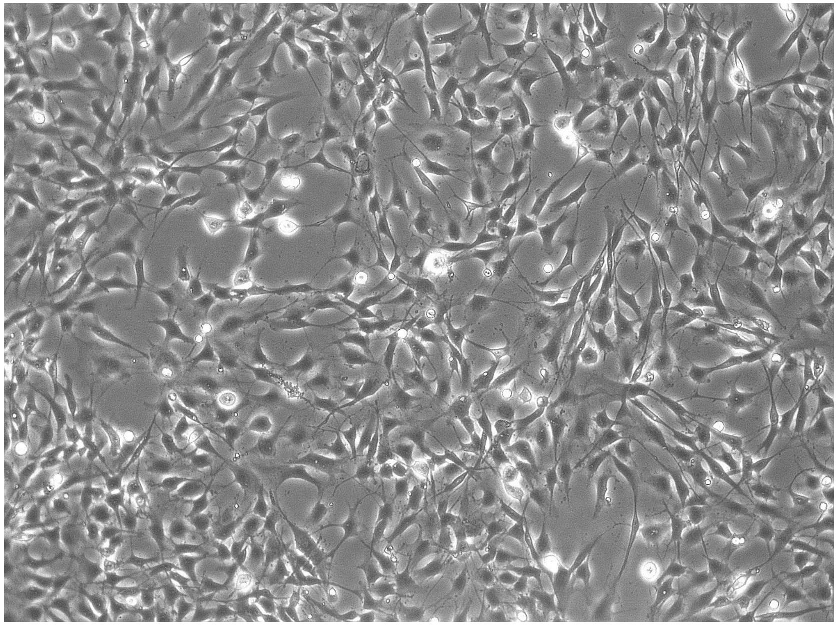

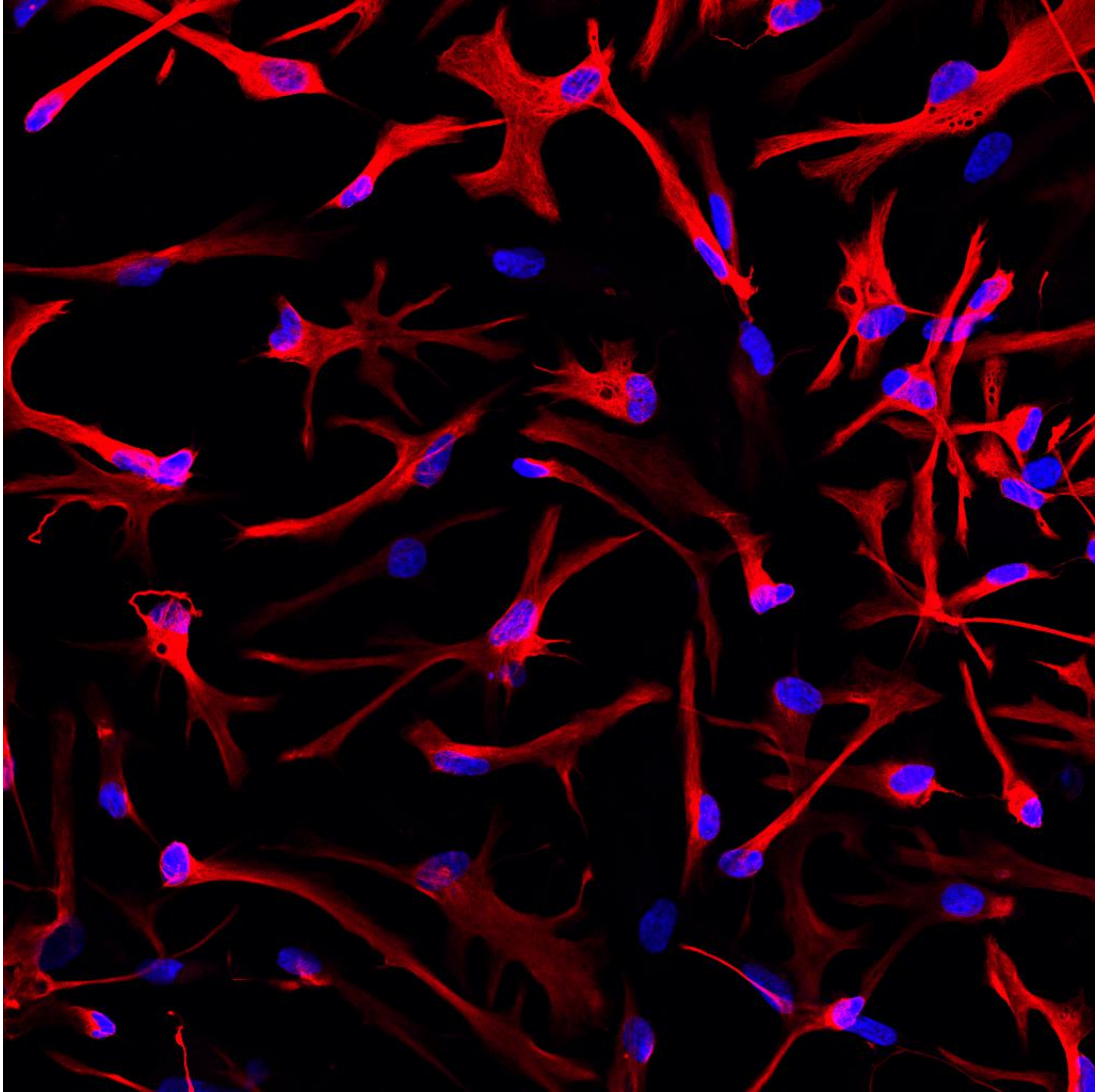

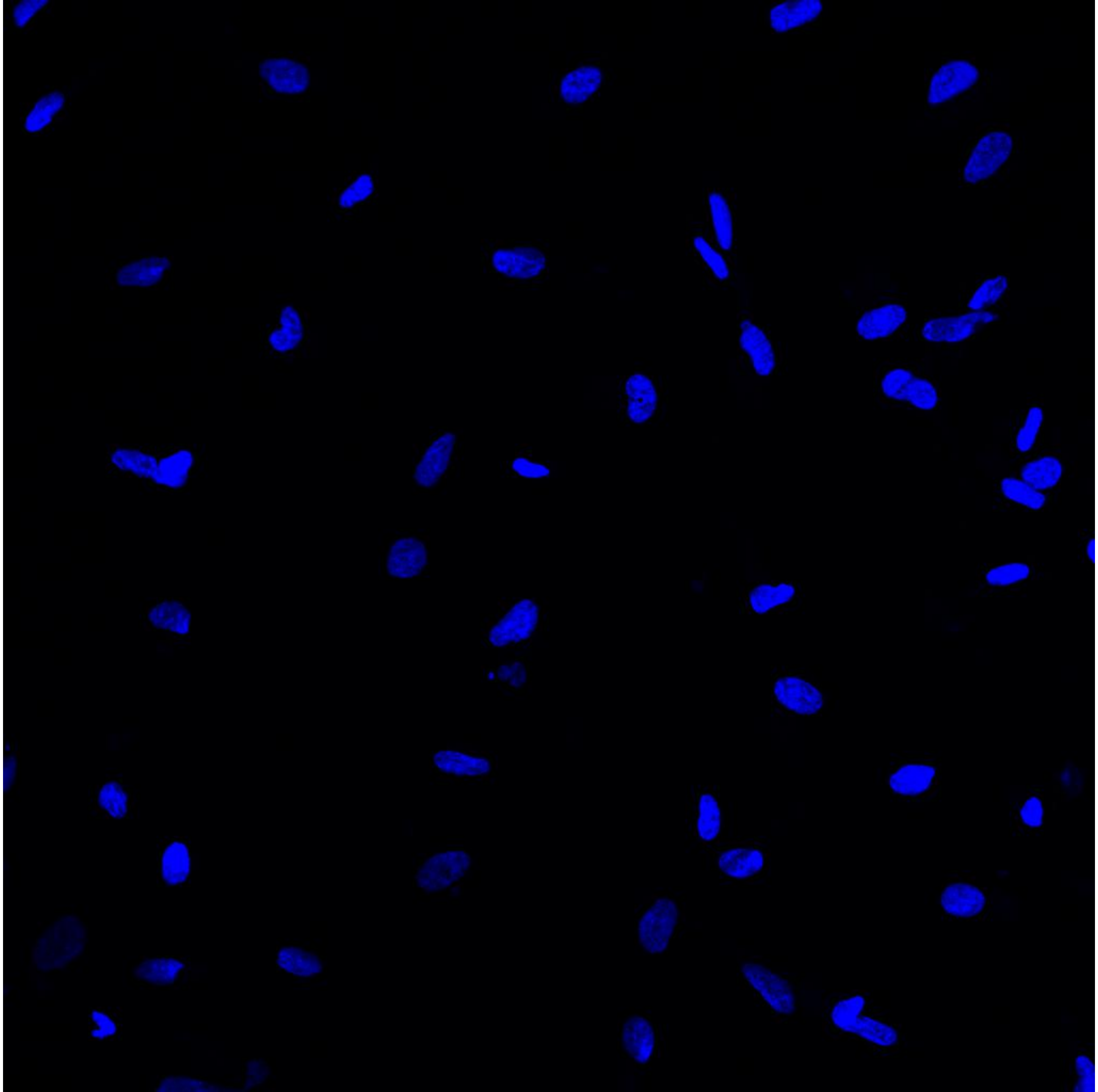

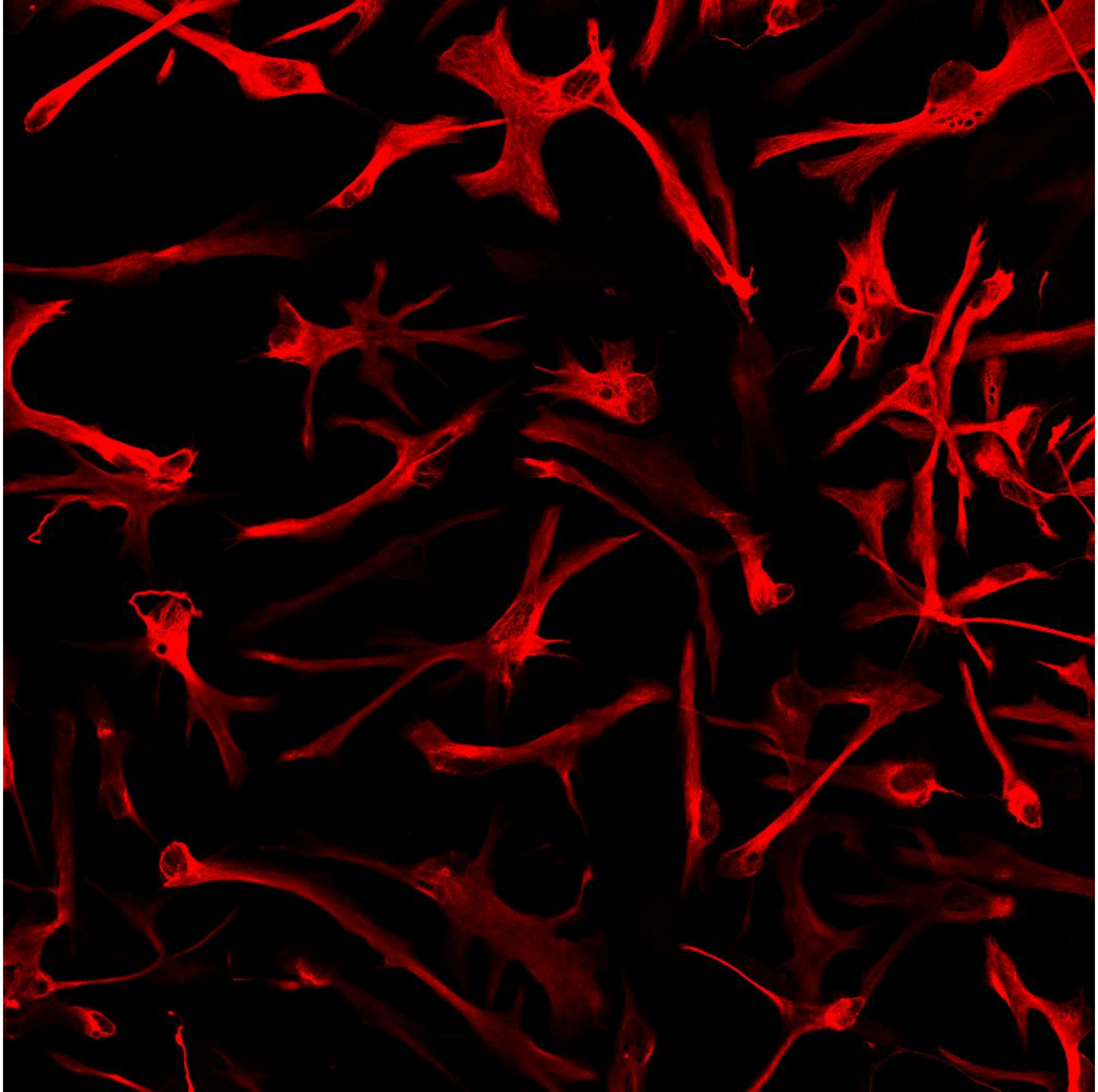

Figure 1B+1C
CTRL

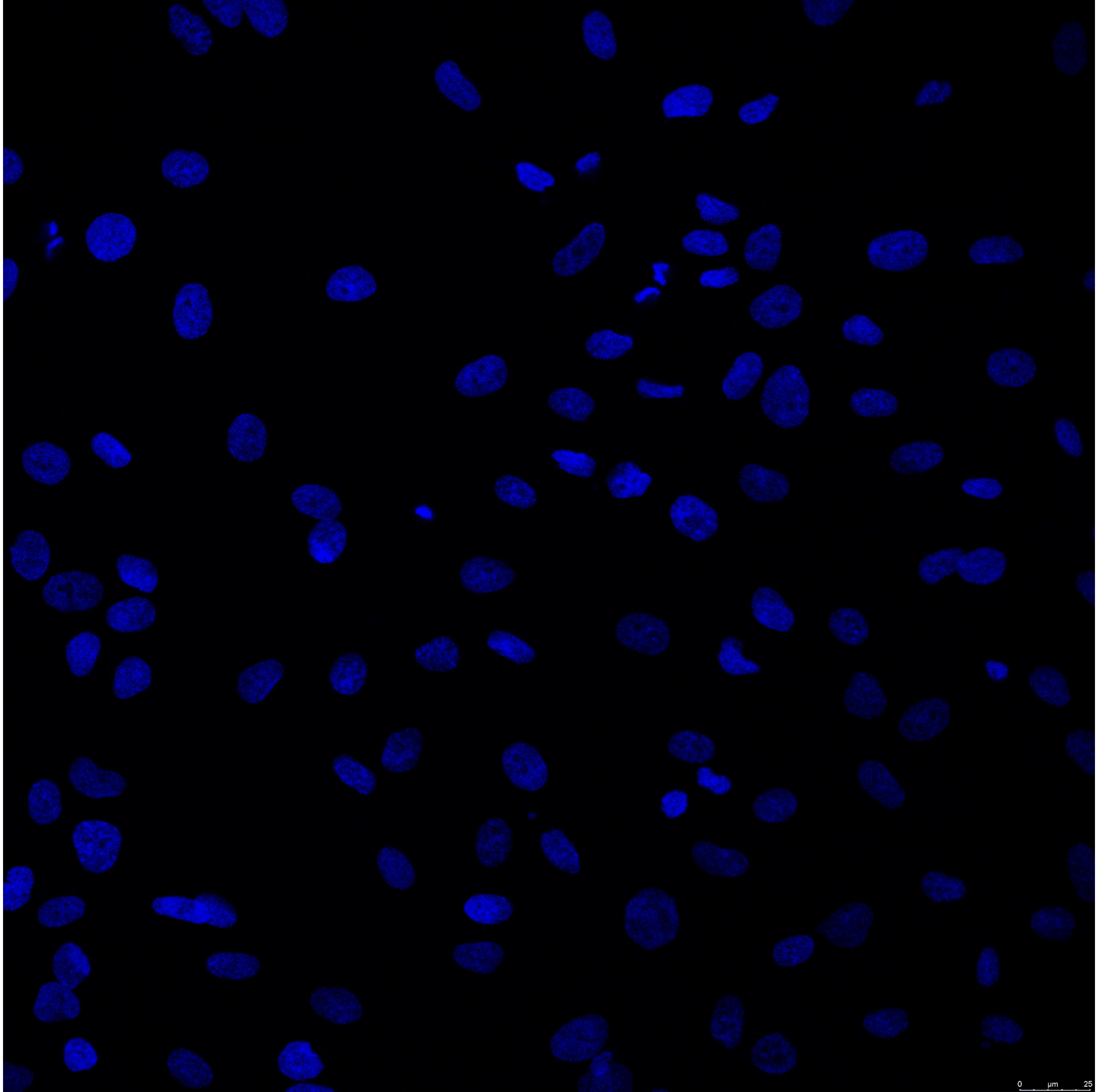

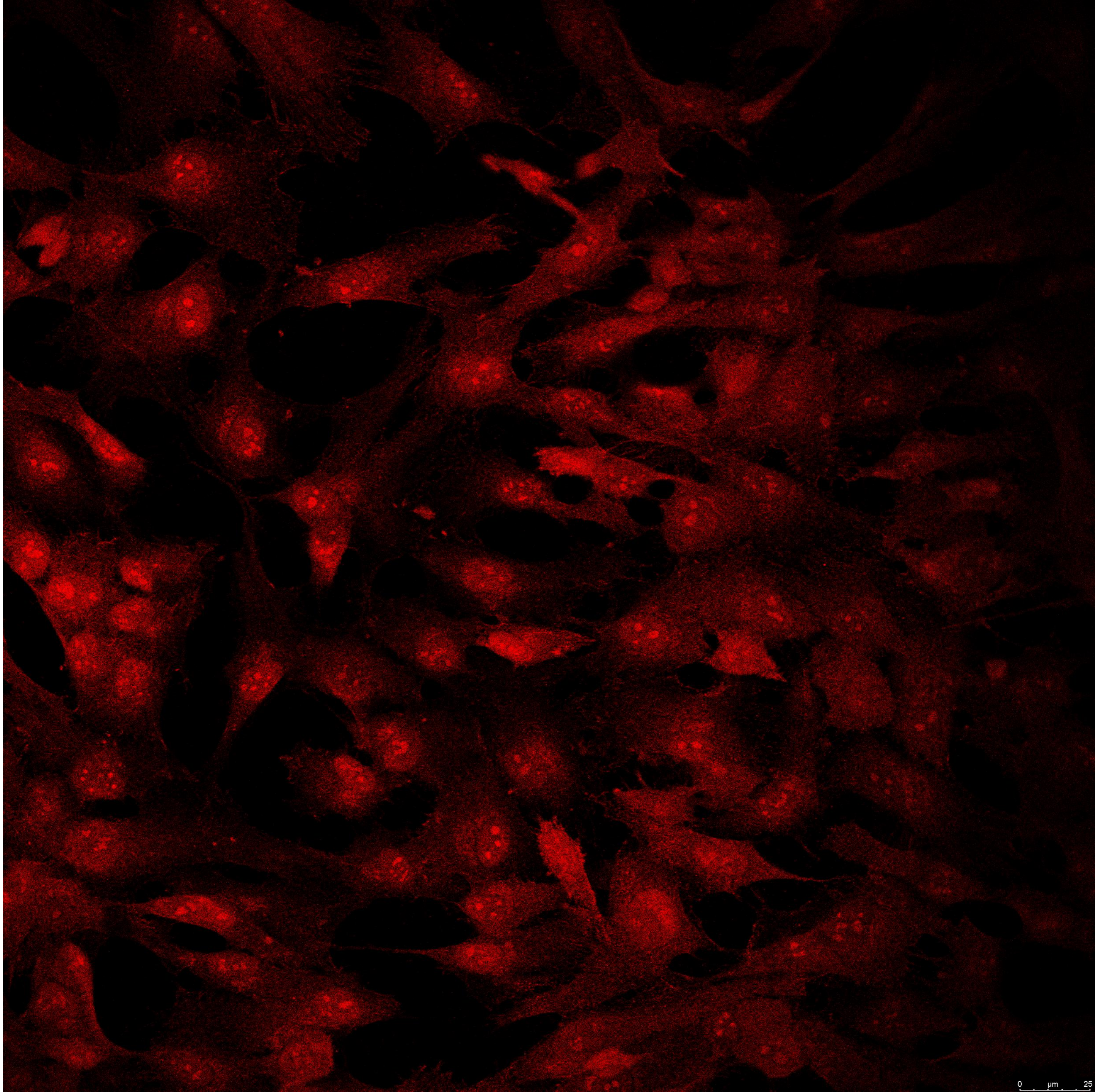
0
µm
25

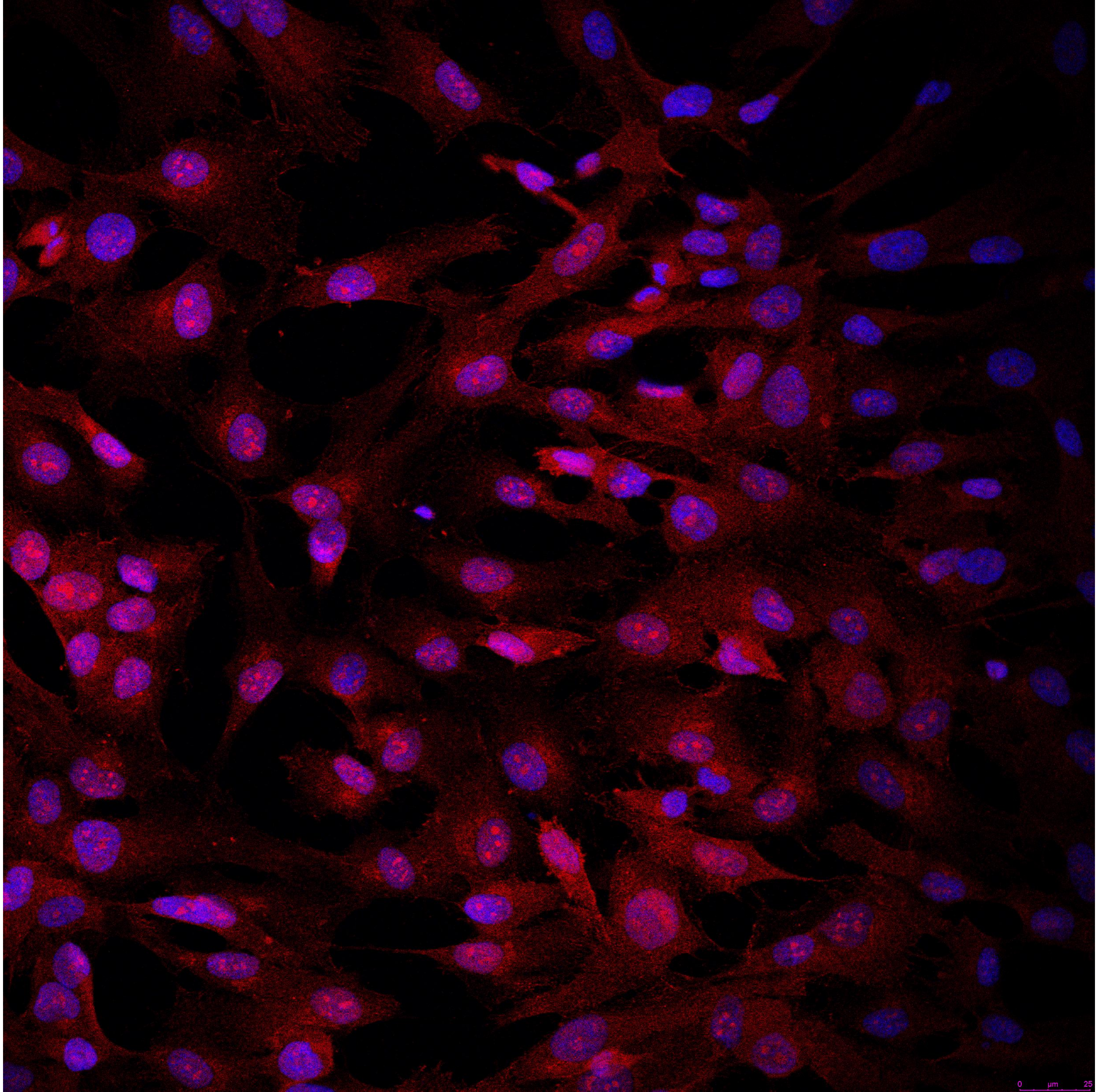
0 µm 25

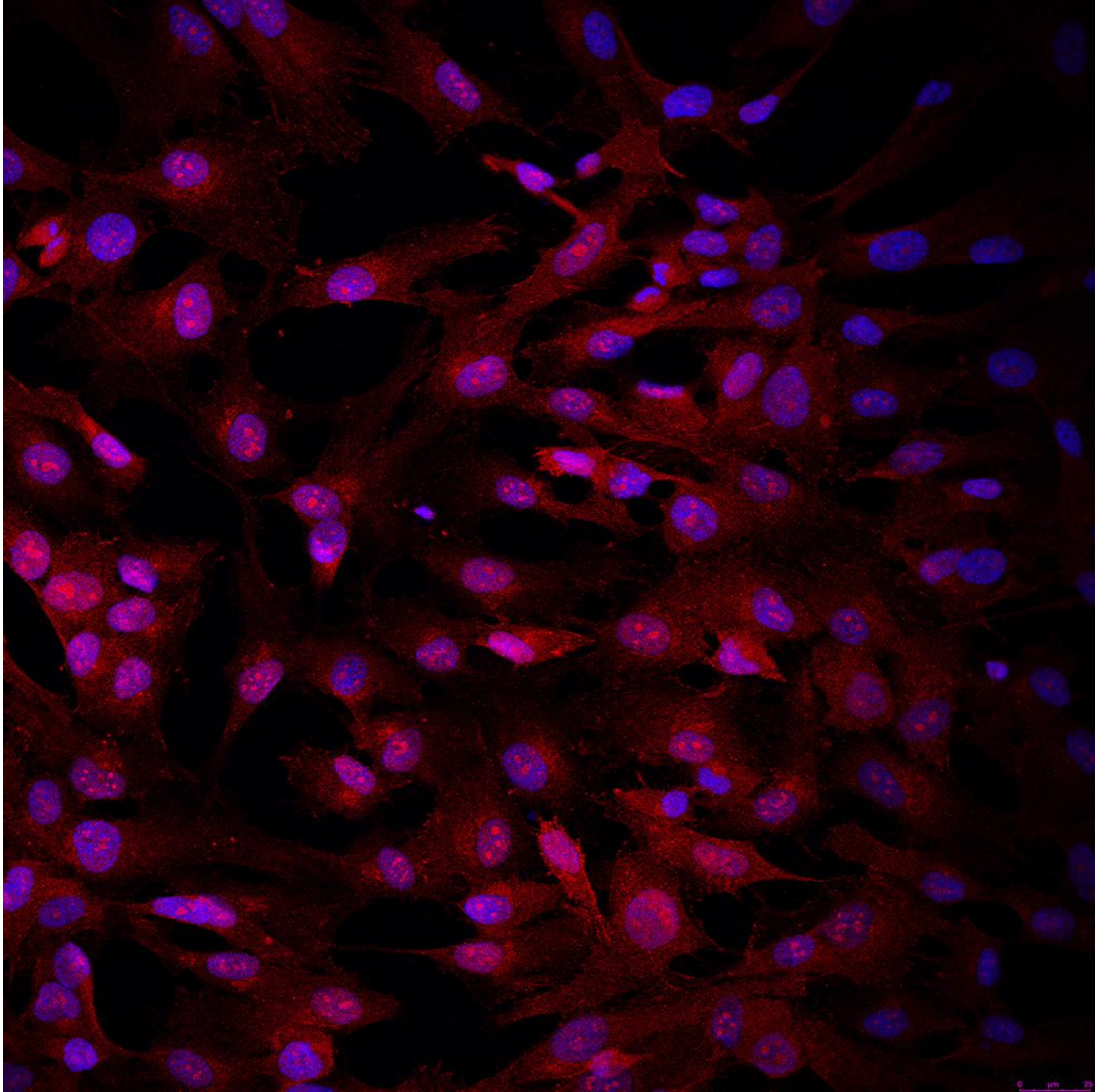
0
µm
25

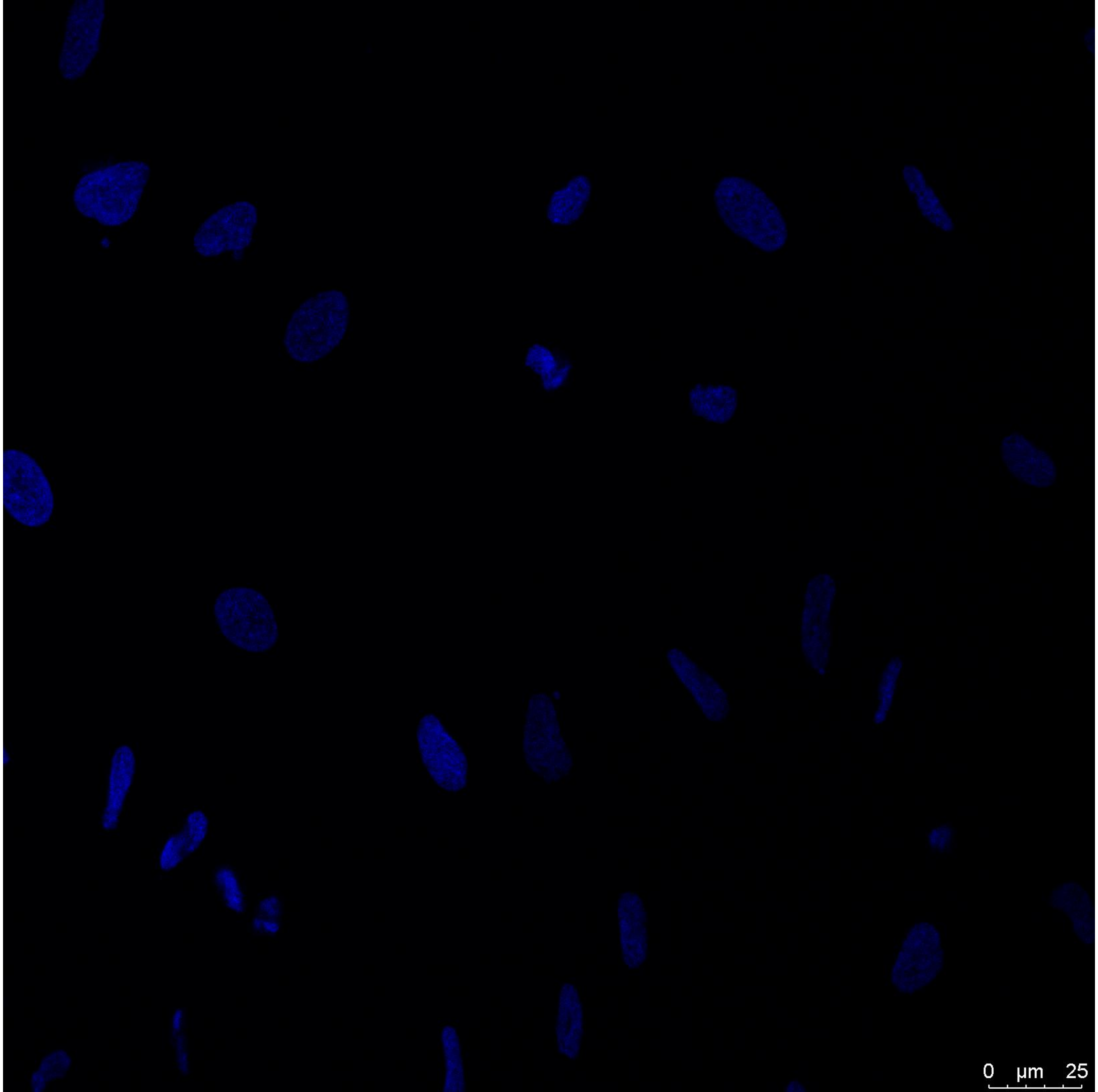
0 µm 25

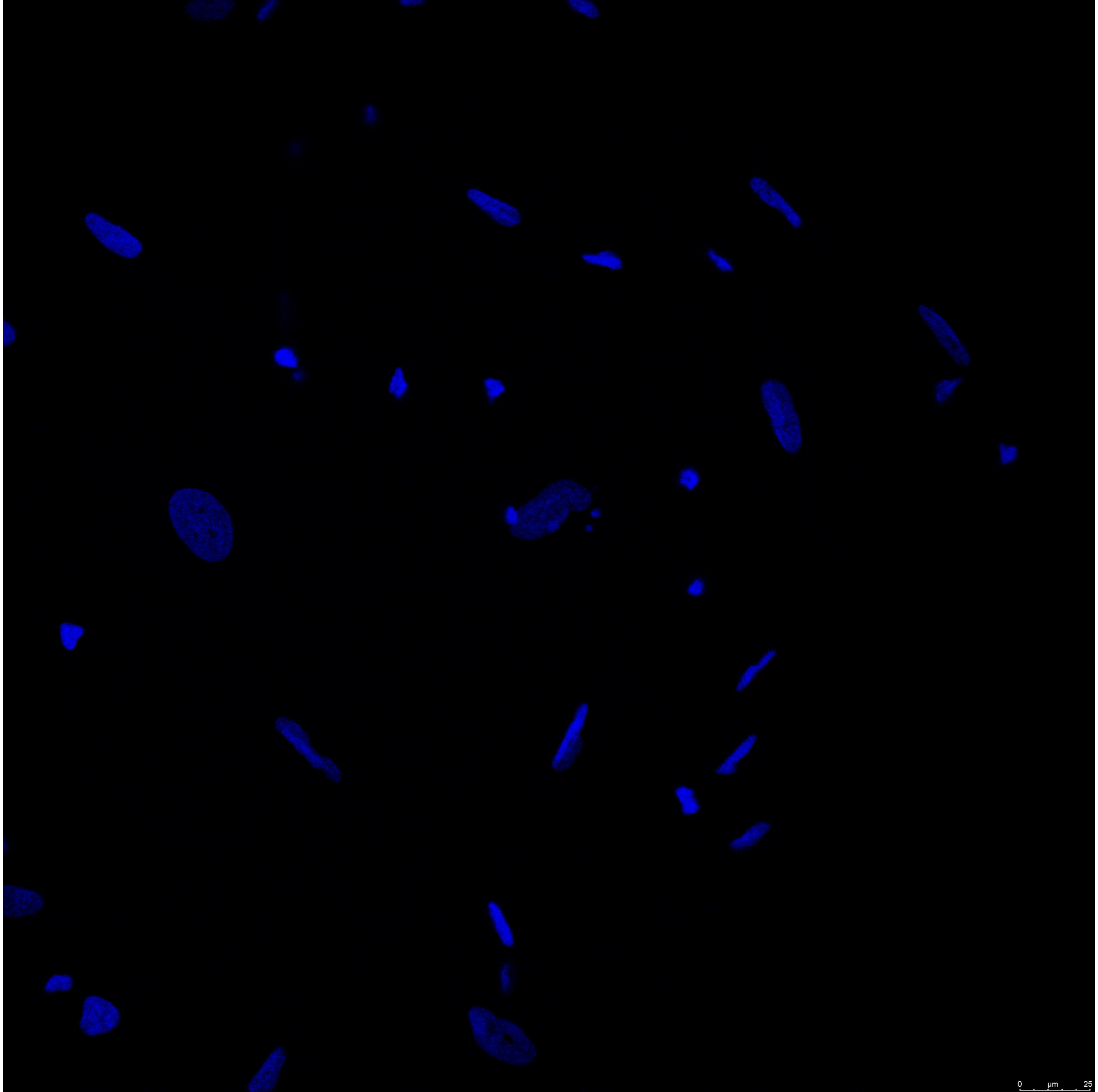
0 µm 25

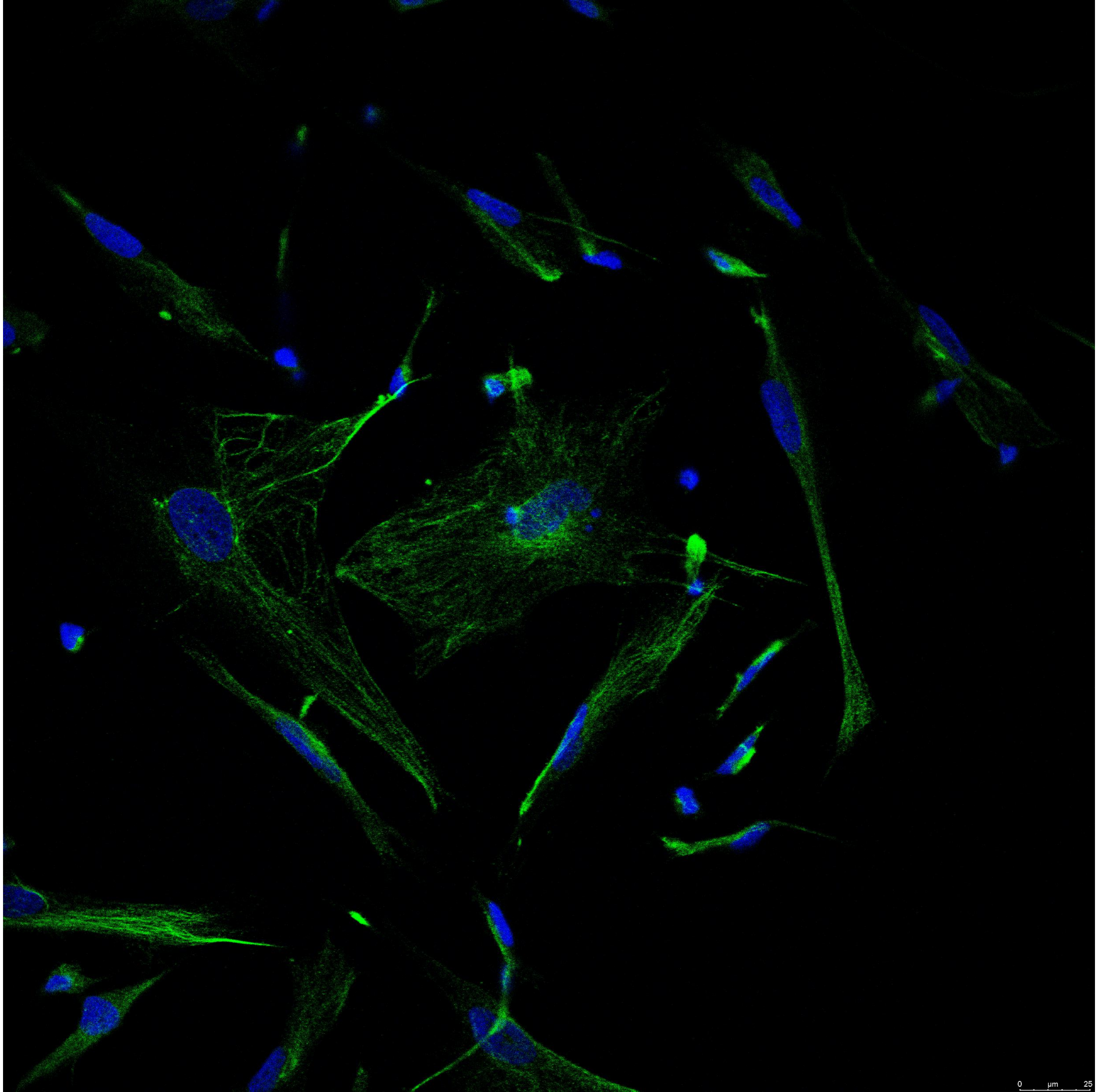
0 µm 25

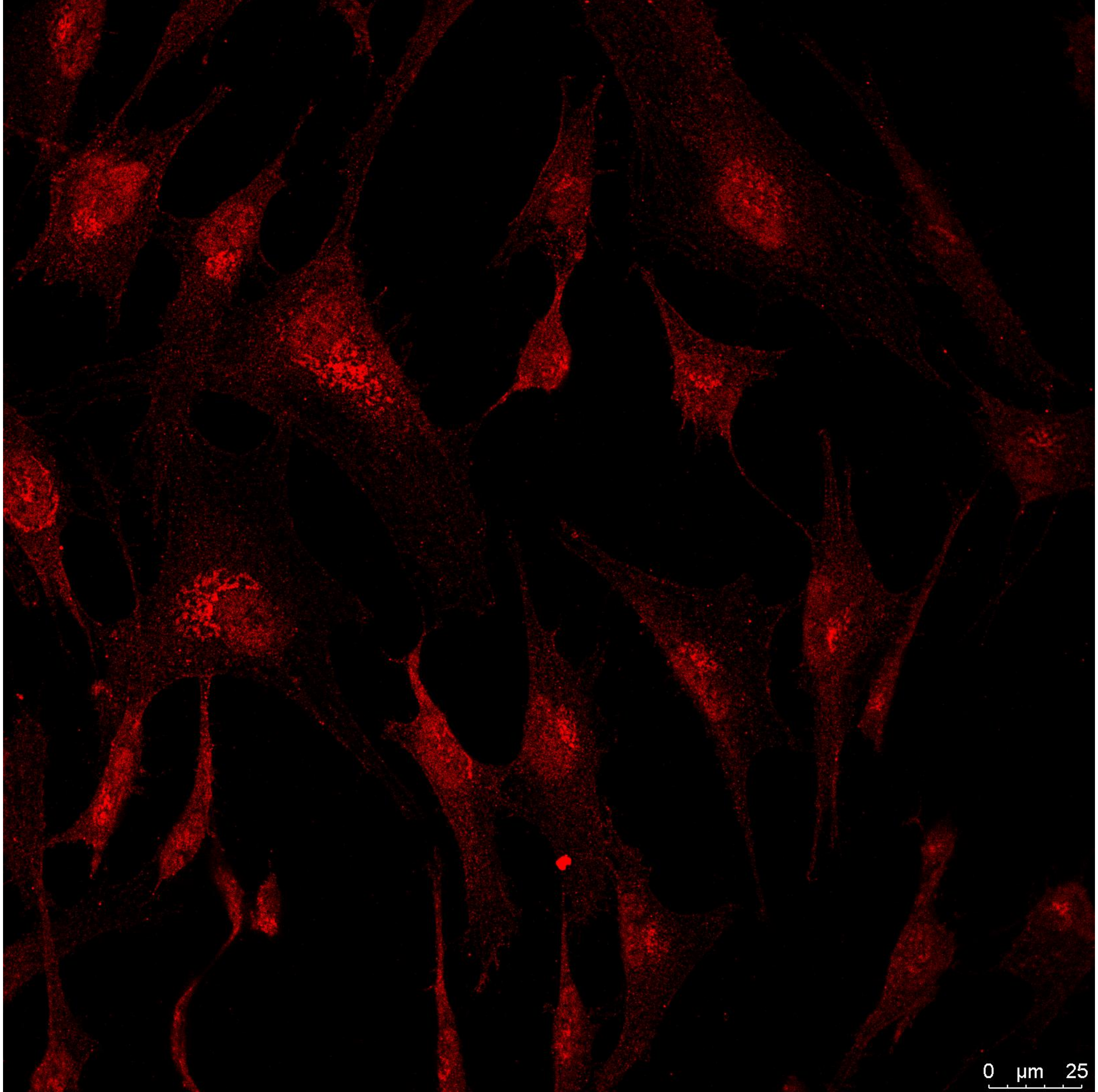
0 µm 25

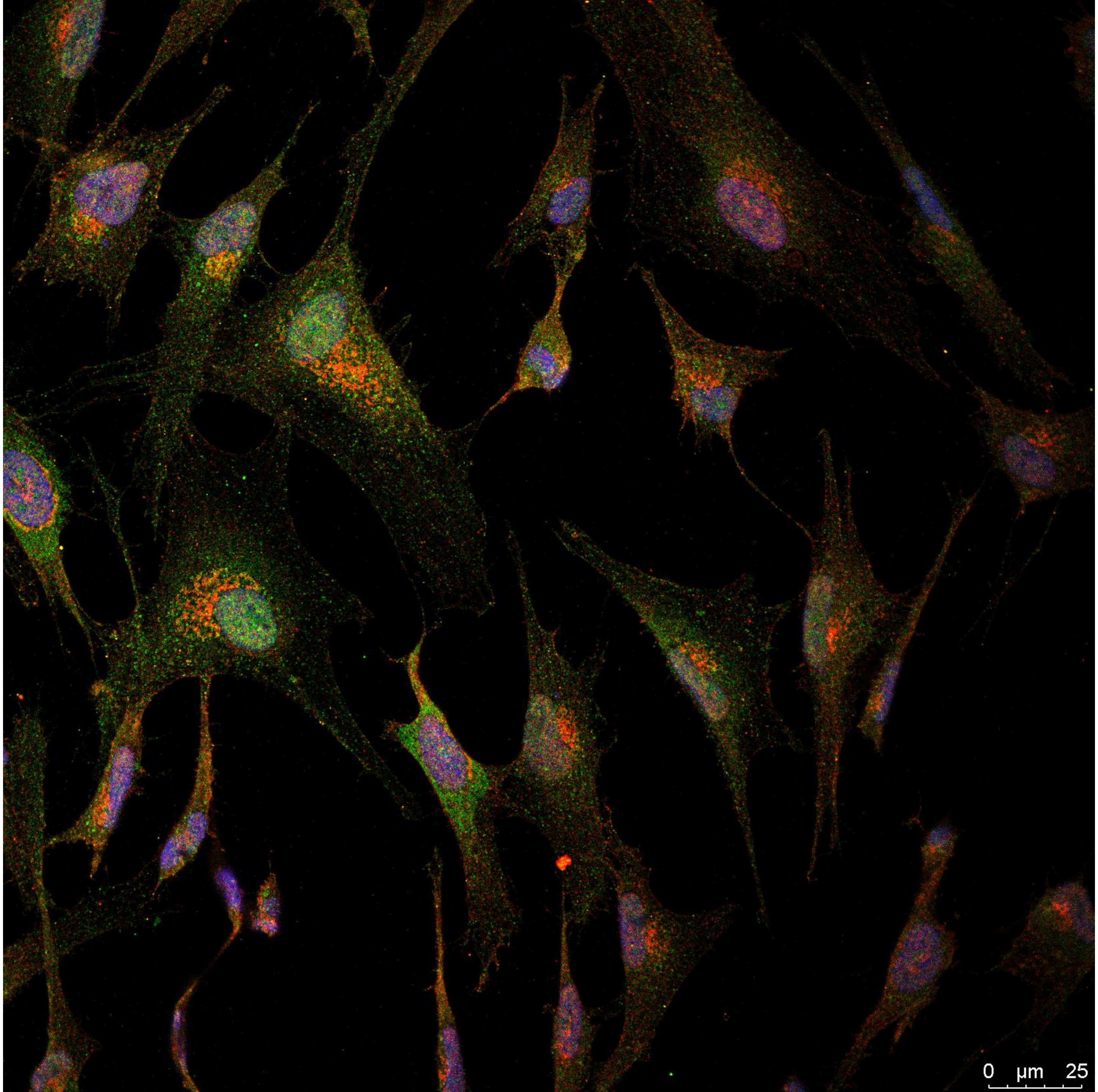
0 µm 25

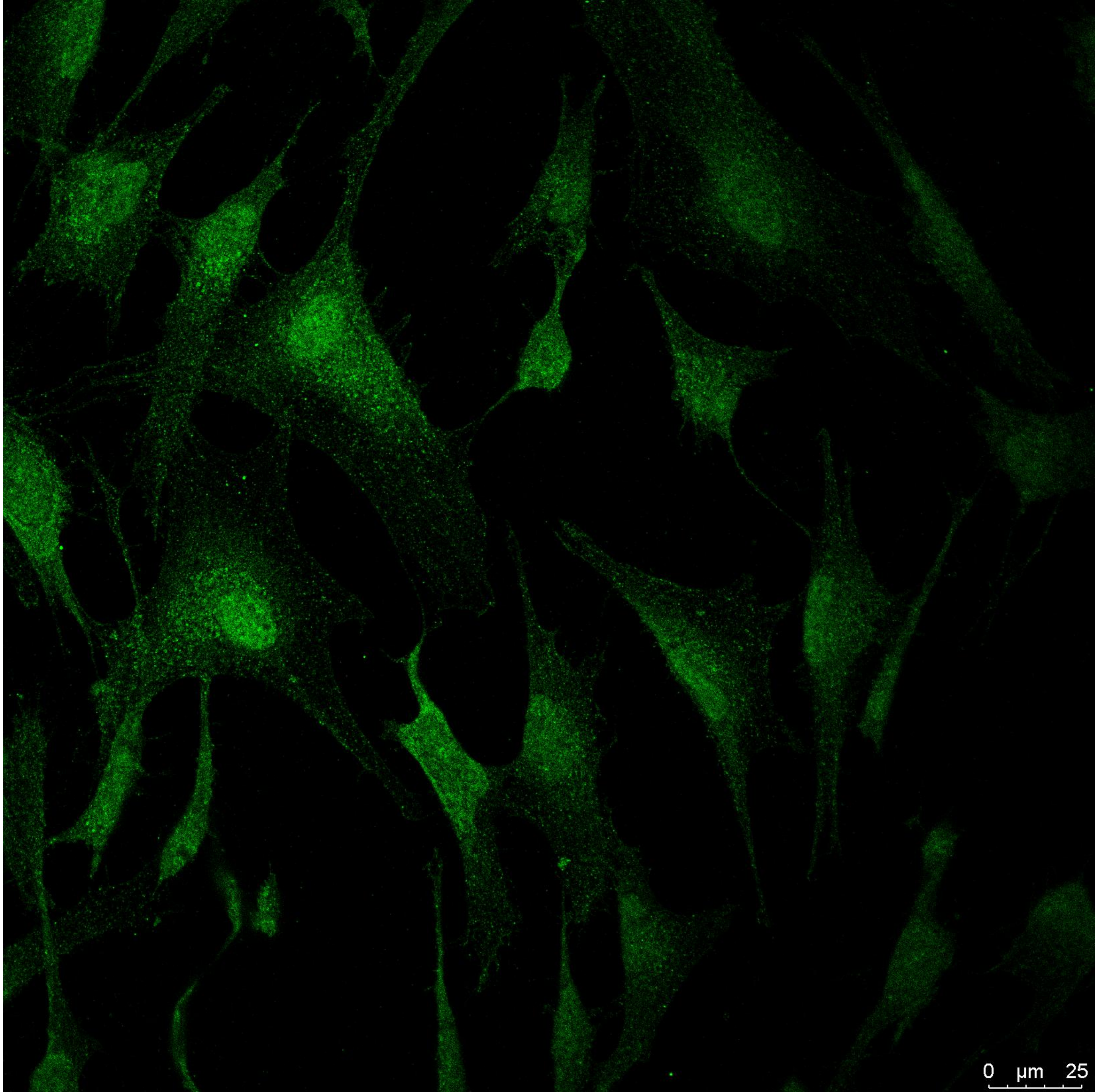
0 µm 25

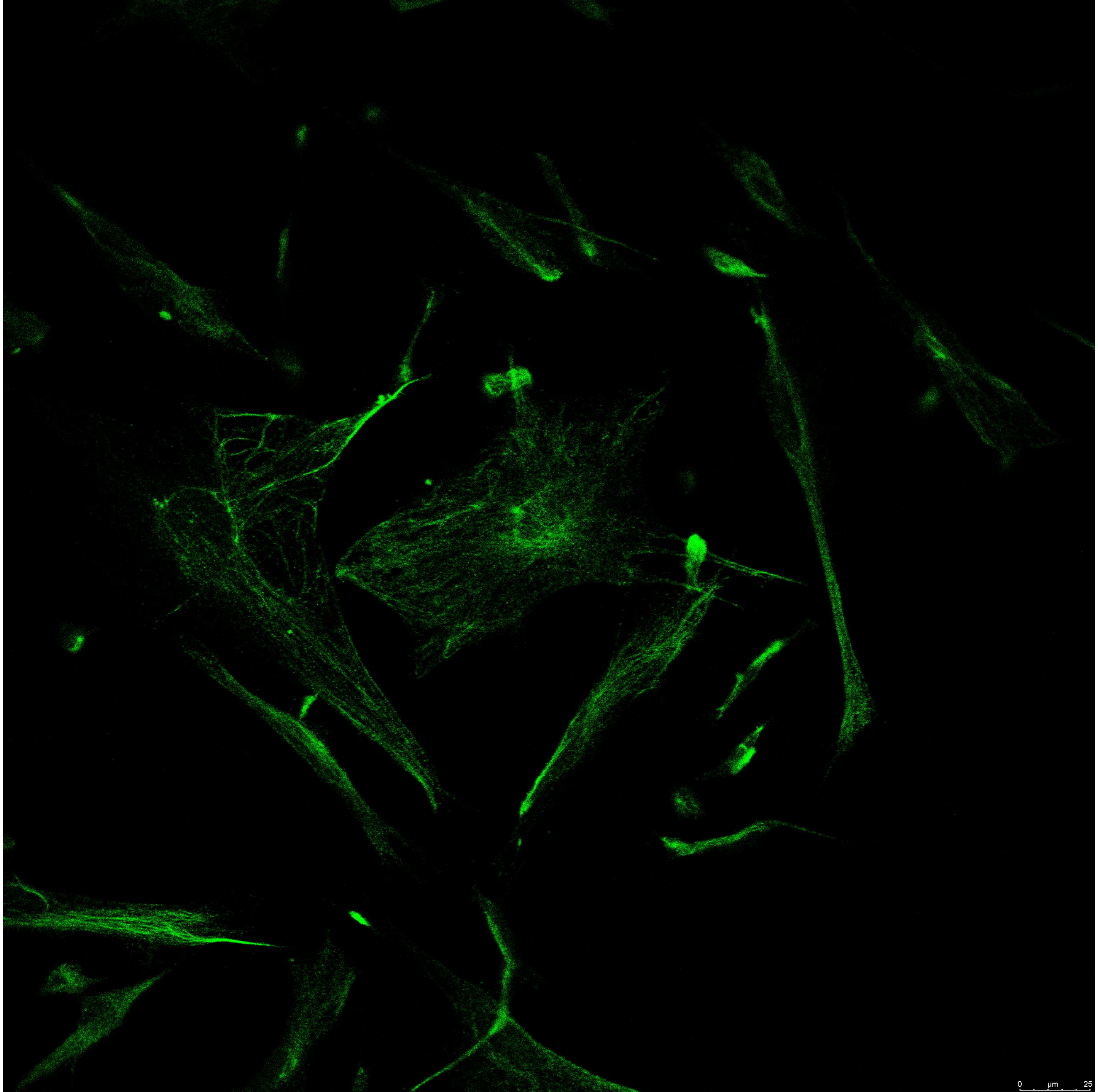
0 µm 25

Figure 1B+1C
WS5A

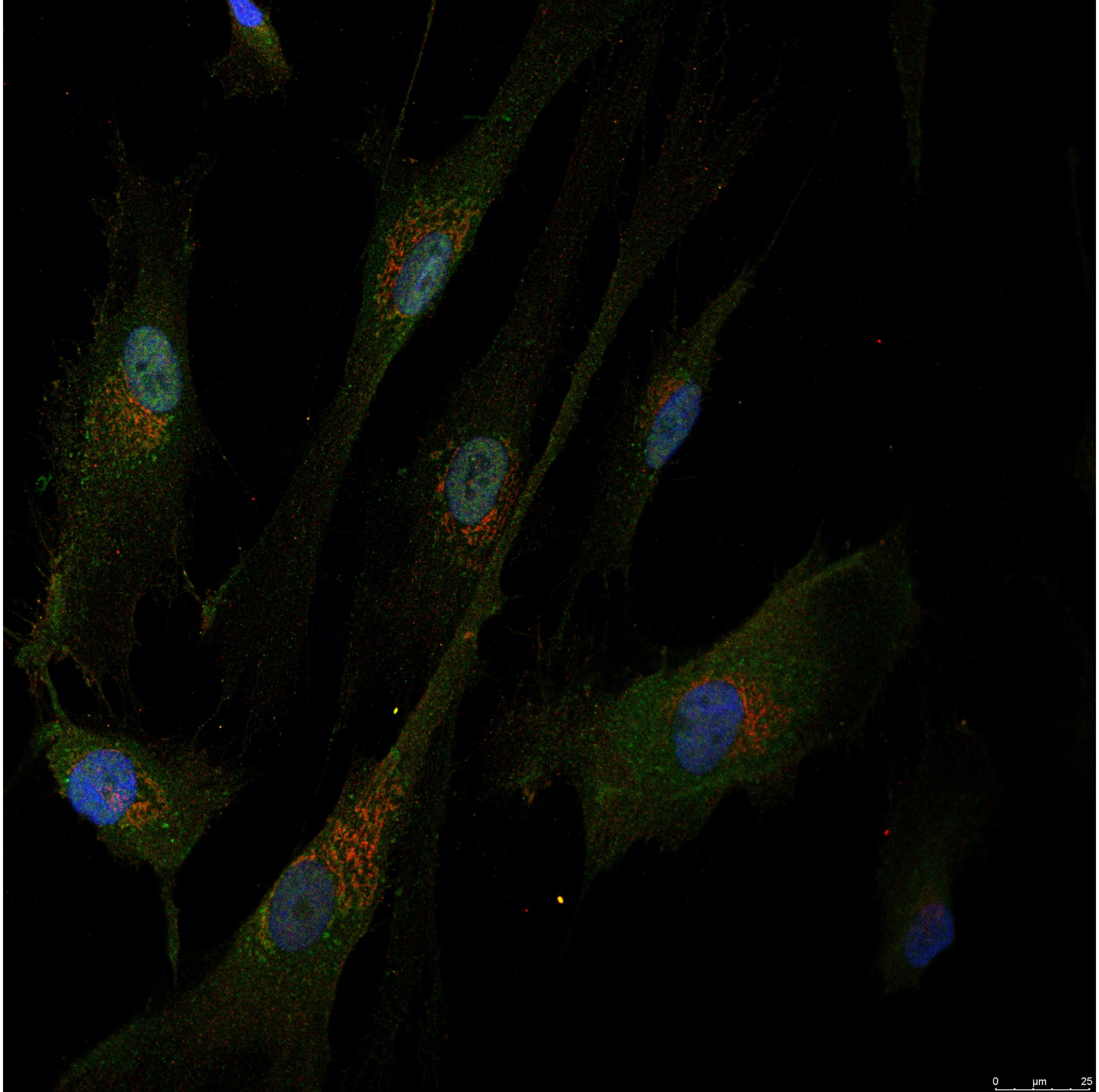

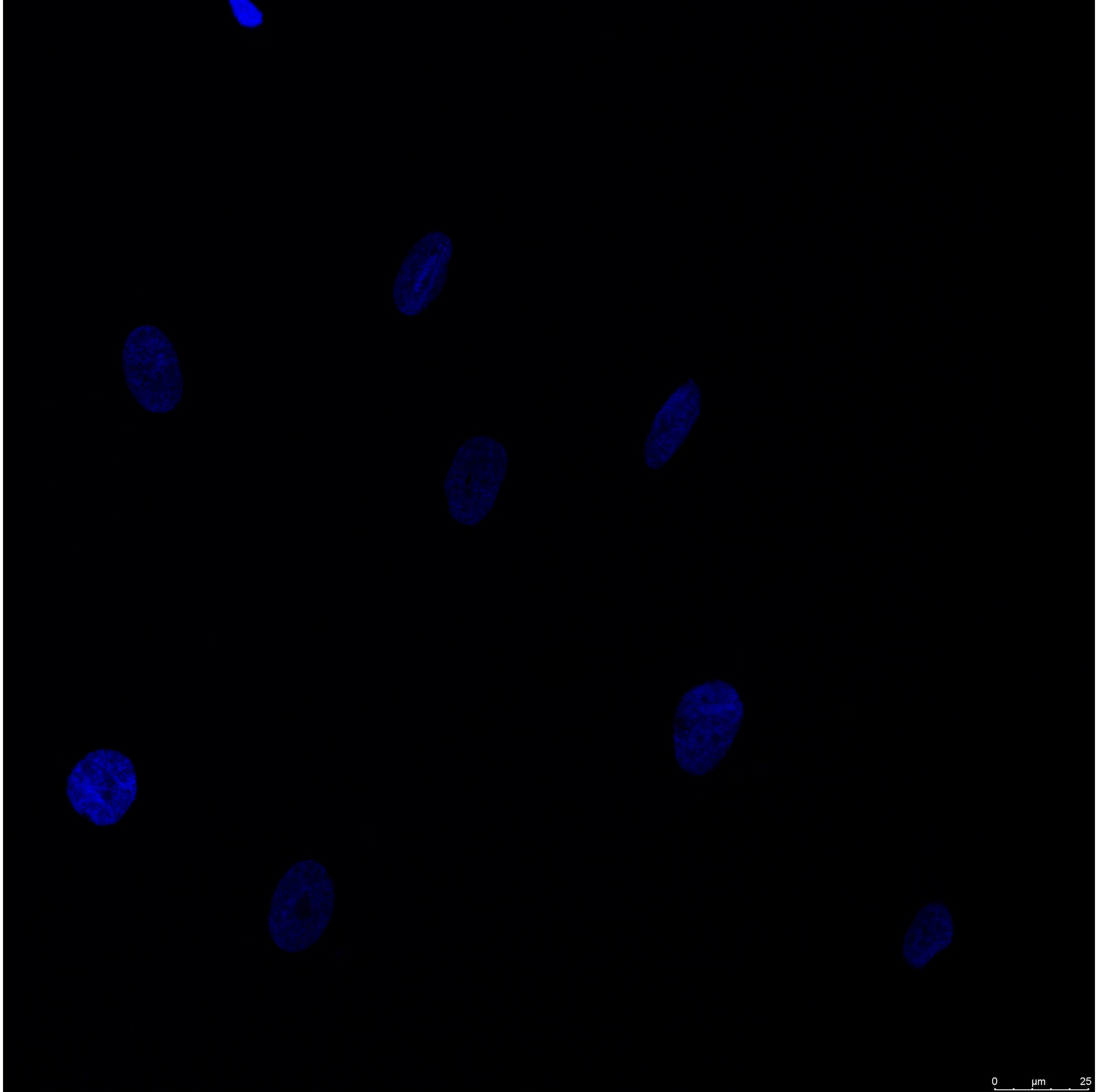
0 μm 25

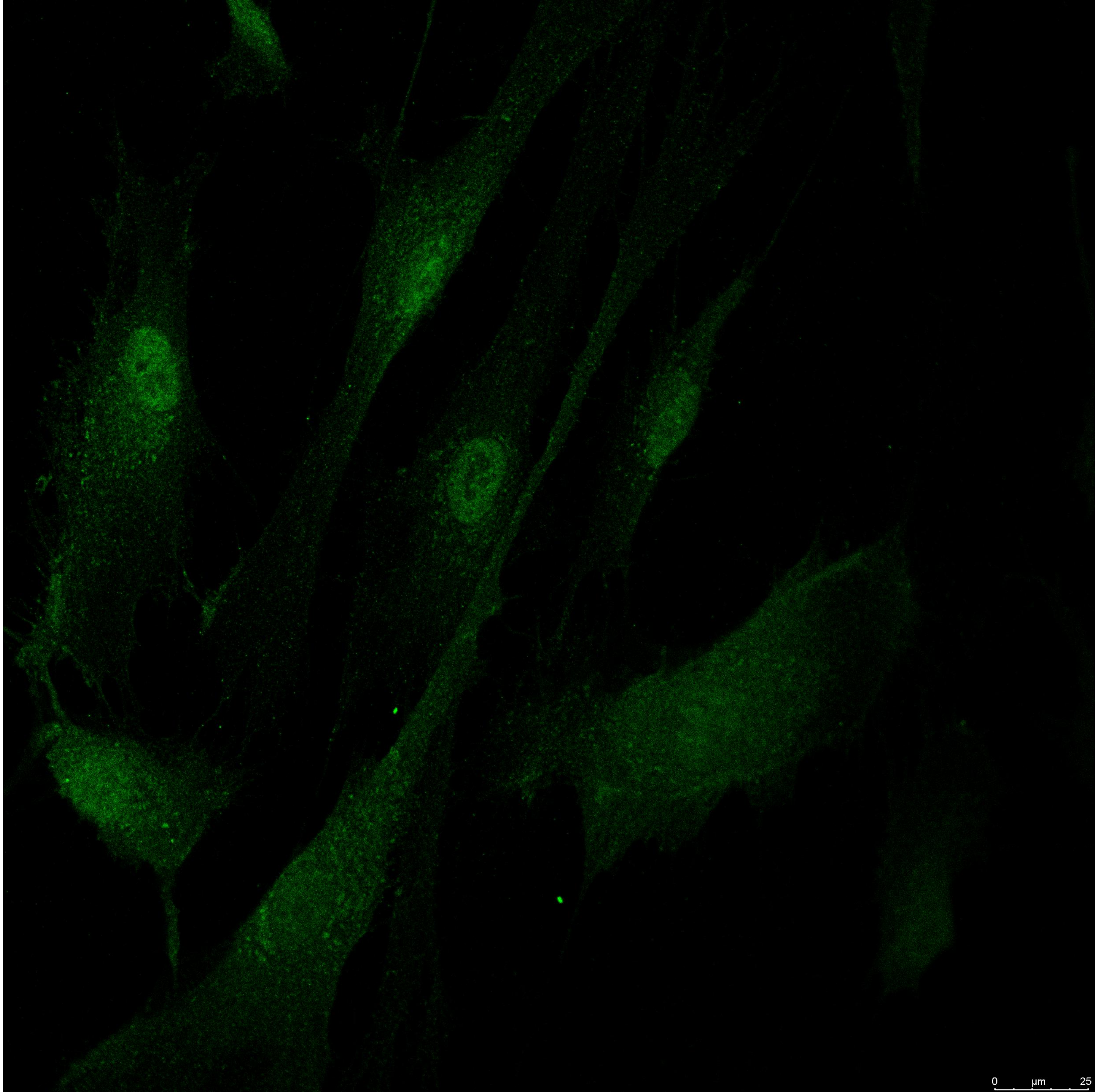
0 µm 25

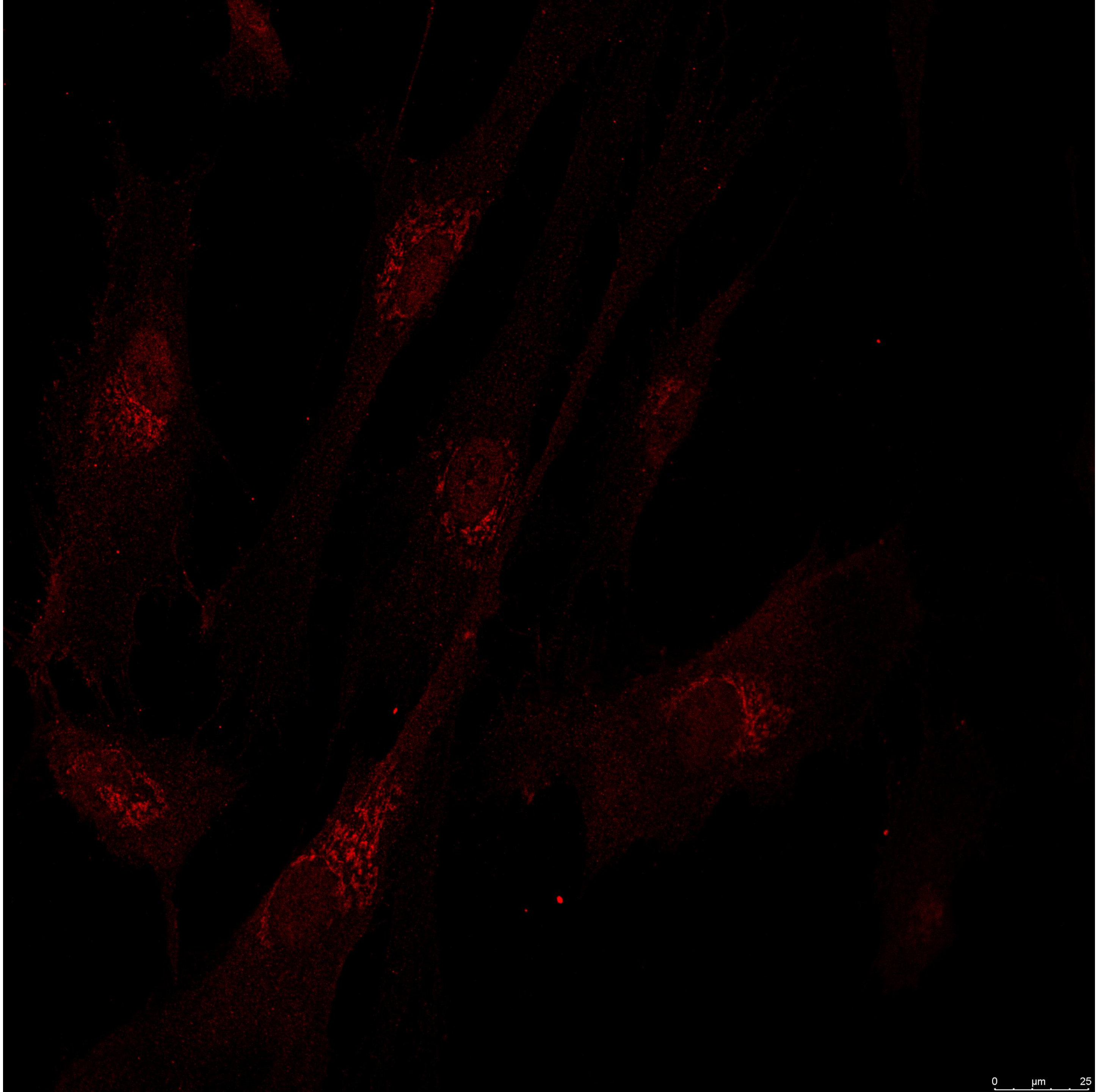
0 µm 25

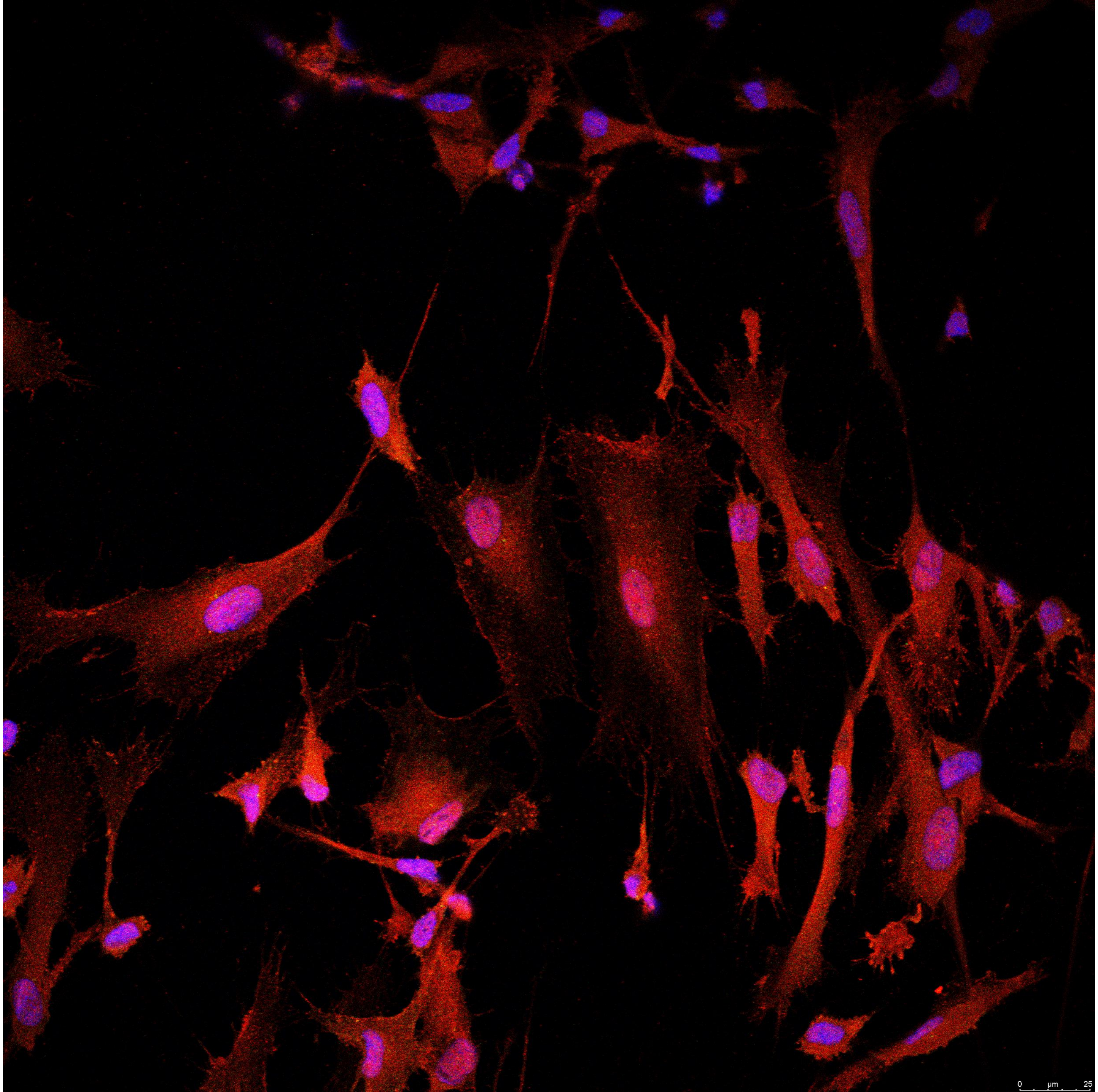
0
µm
25

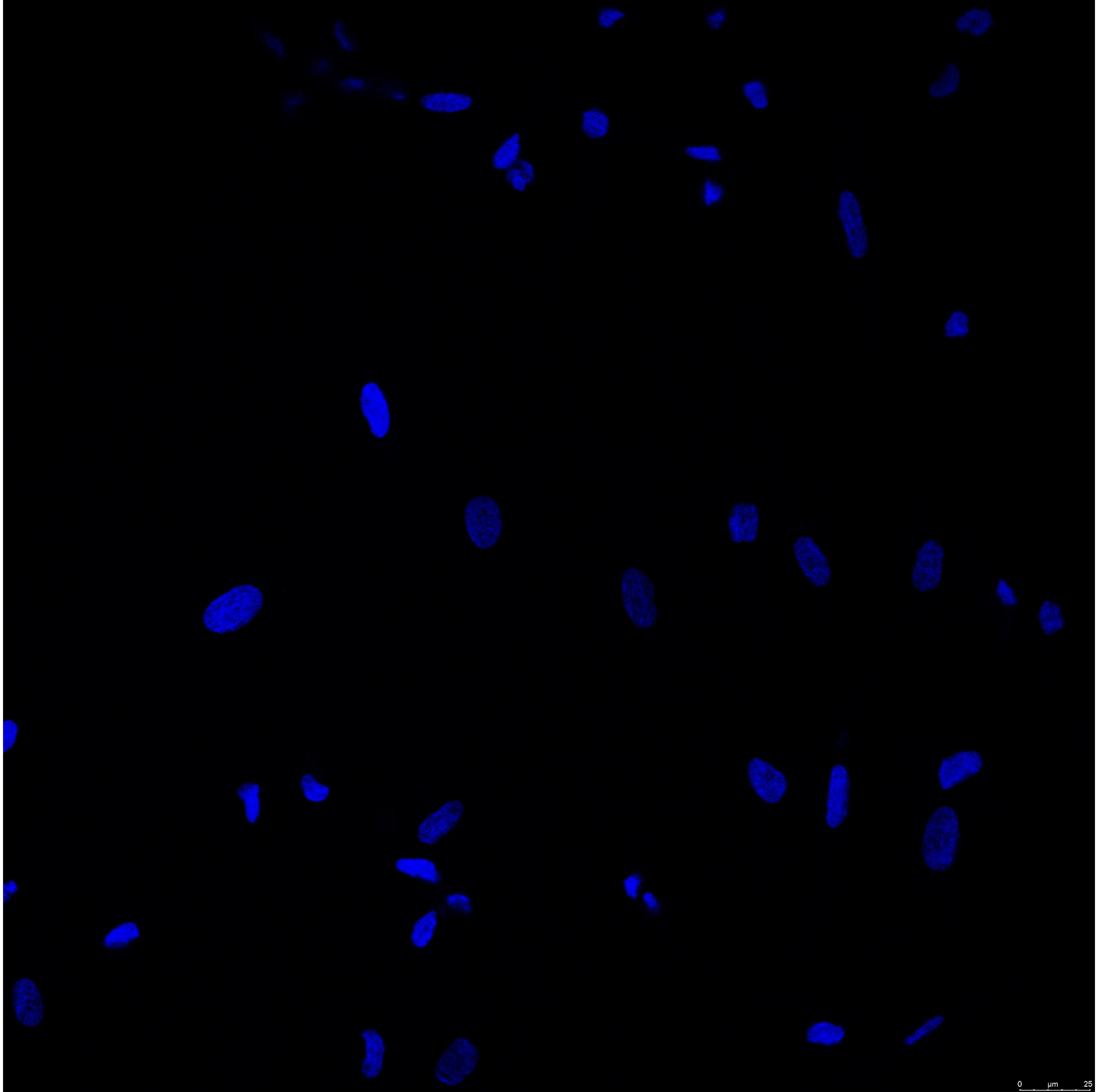
0
µm
25

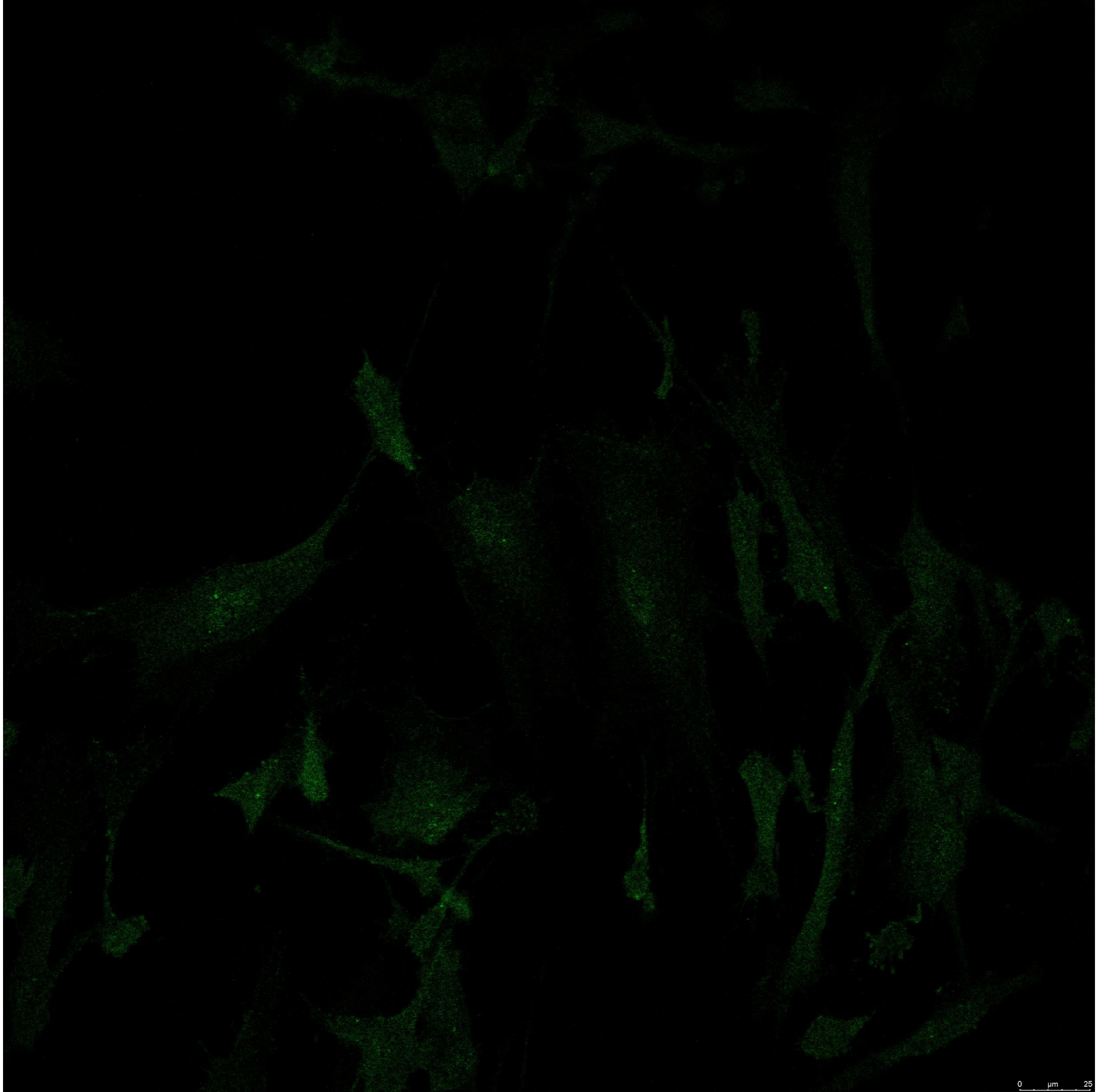
0 µm 25

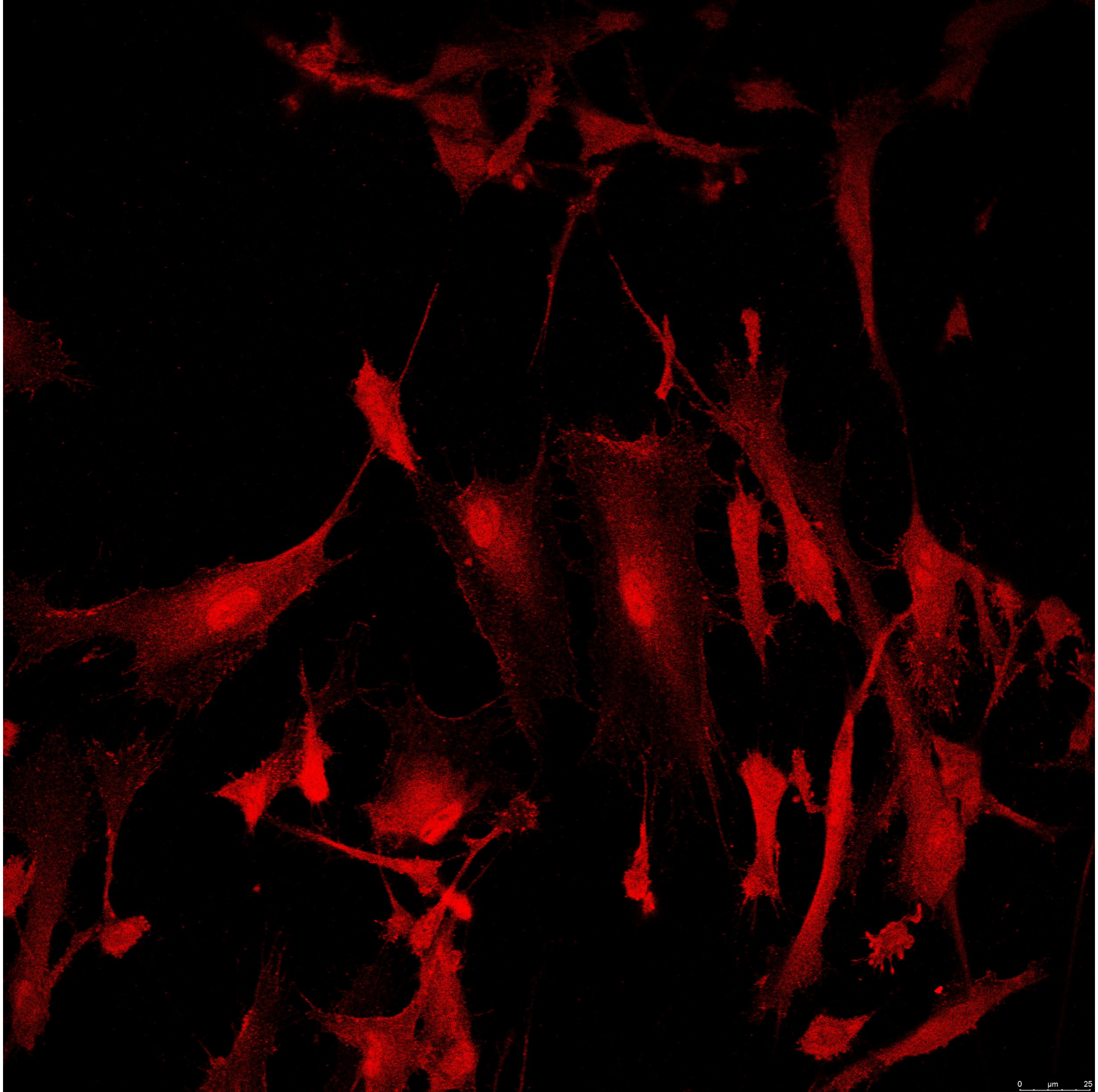
0 µm 25

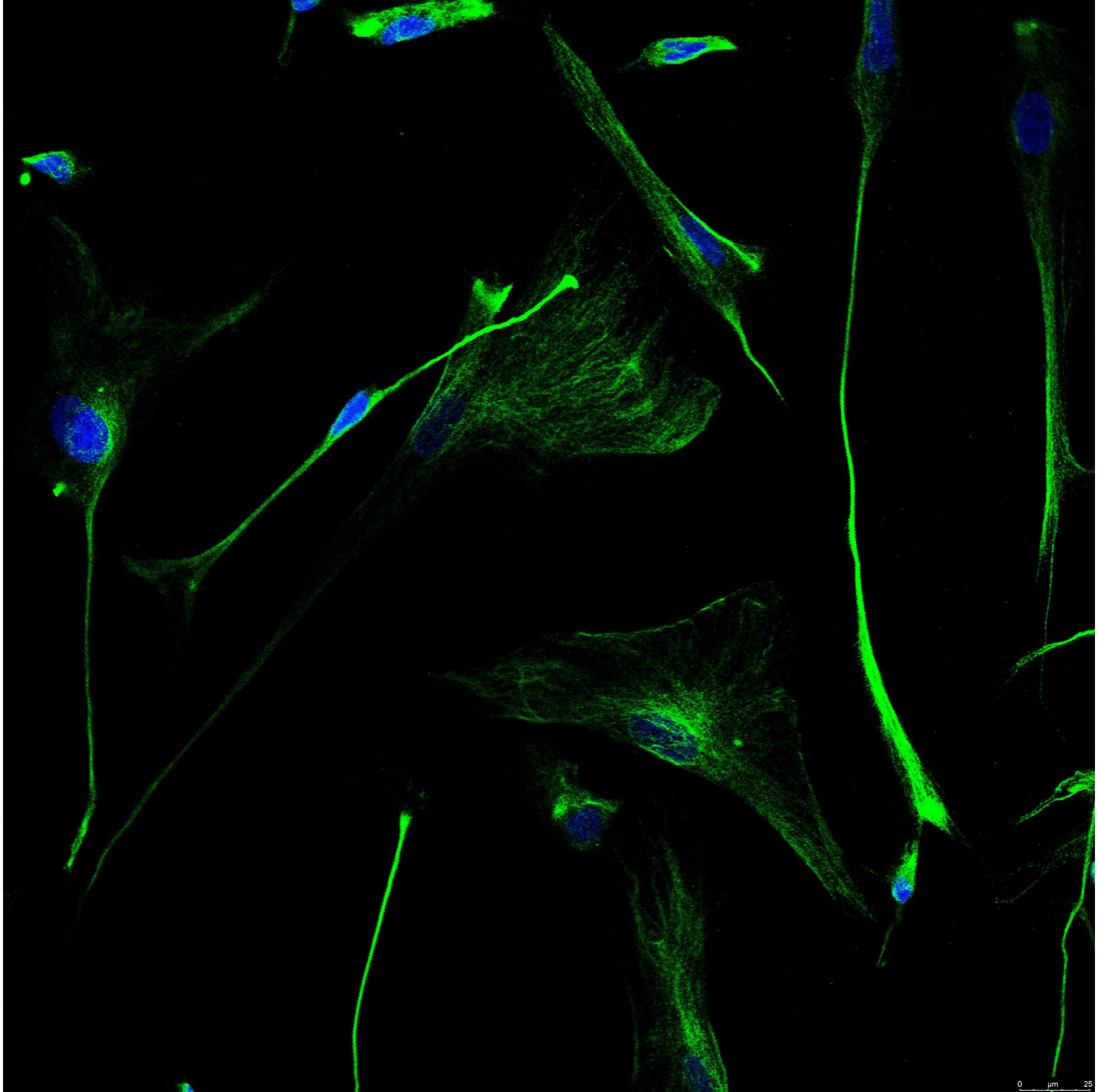
0 µm 25

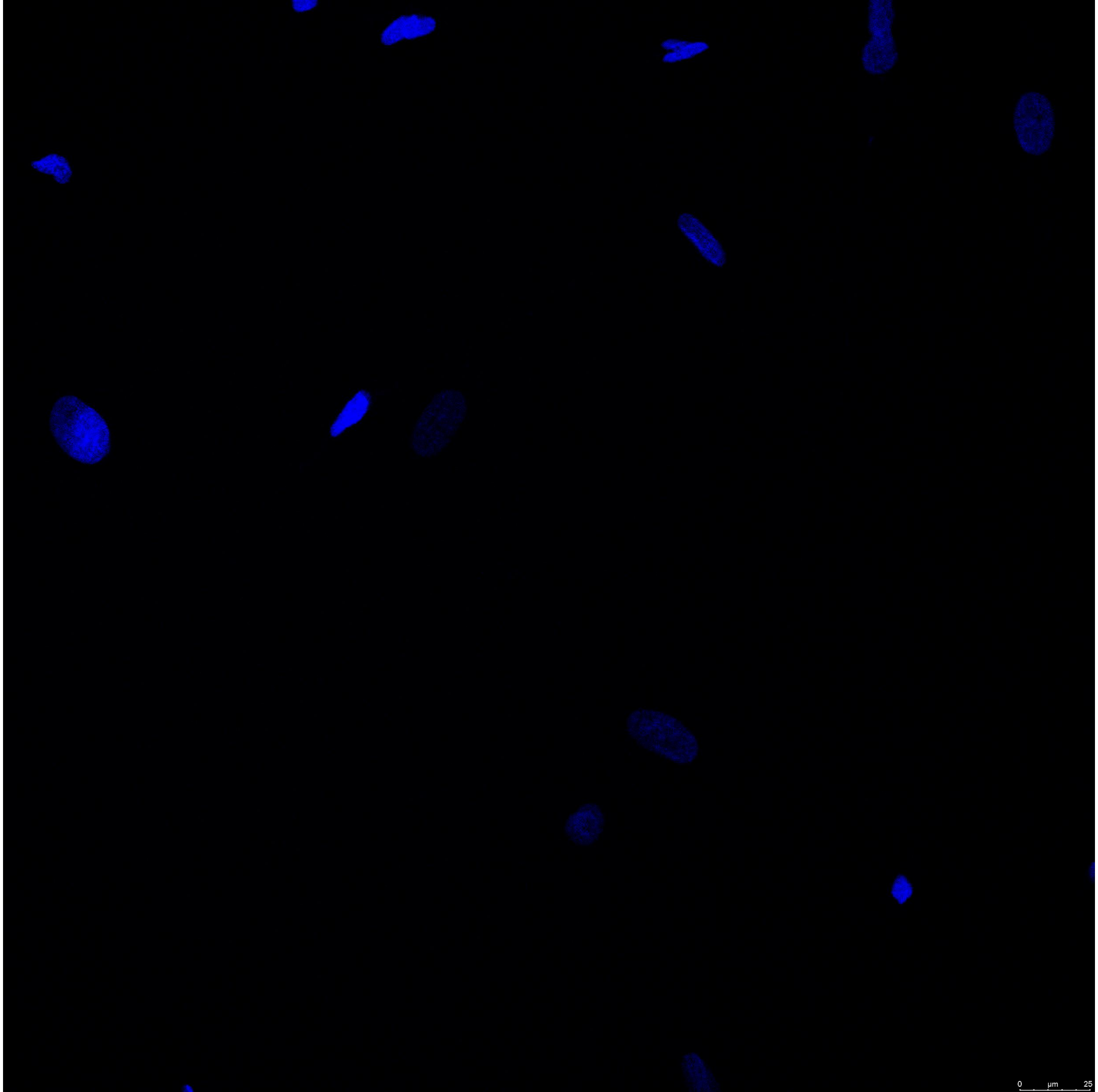
0 µm 25

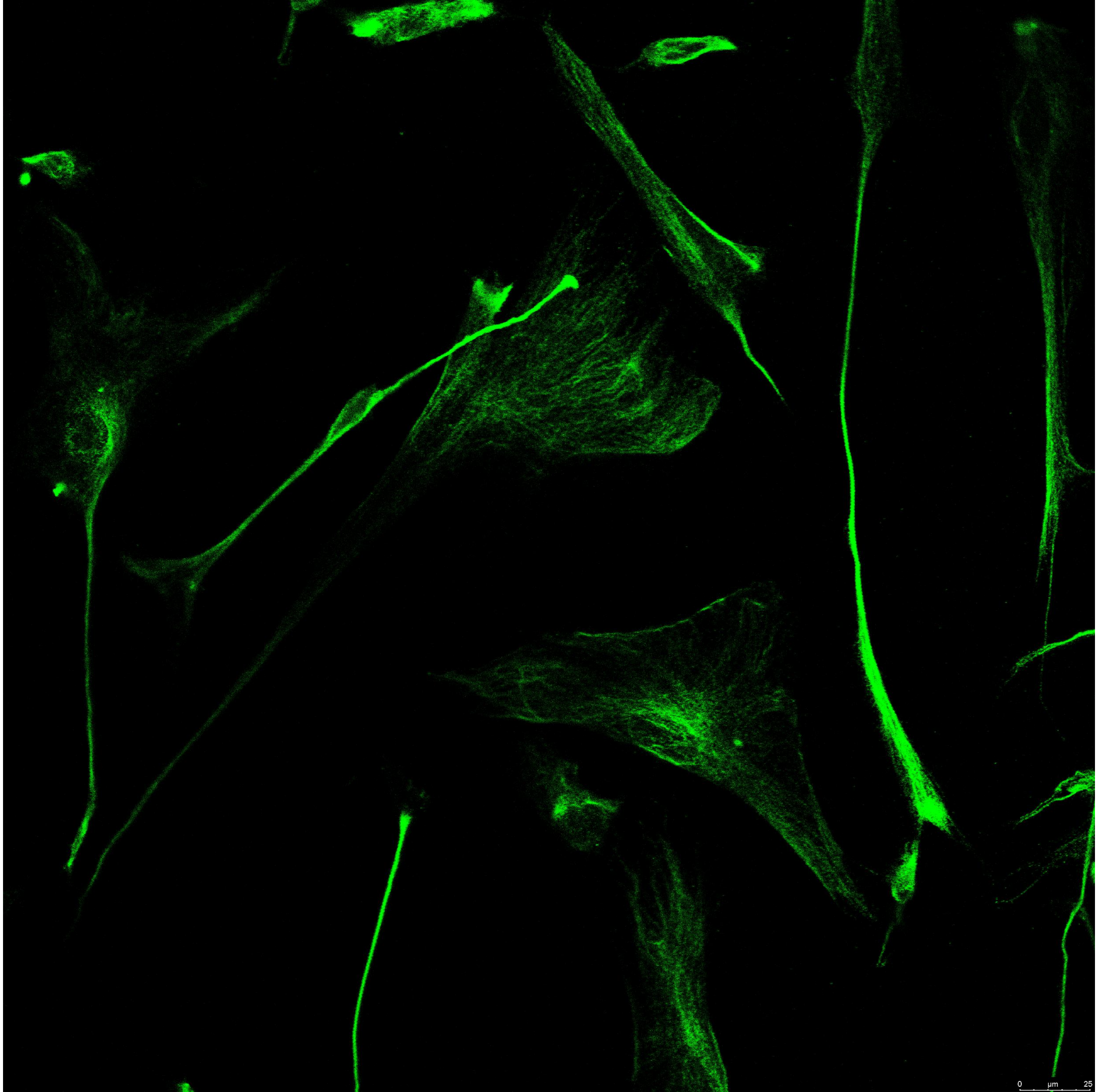
0 µm 25

Figure 1B+1C
CP2A

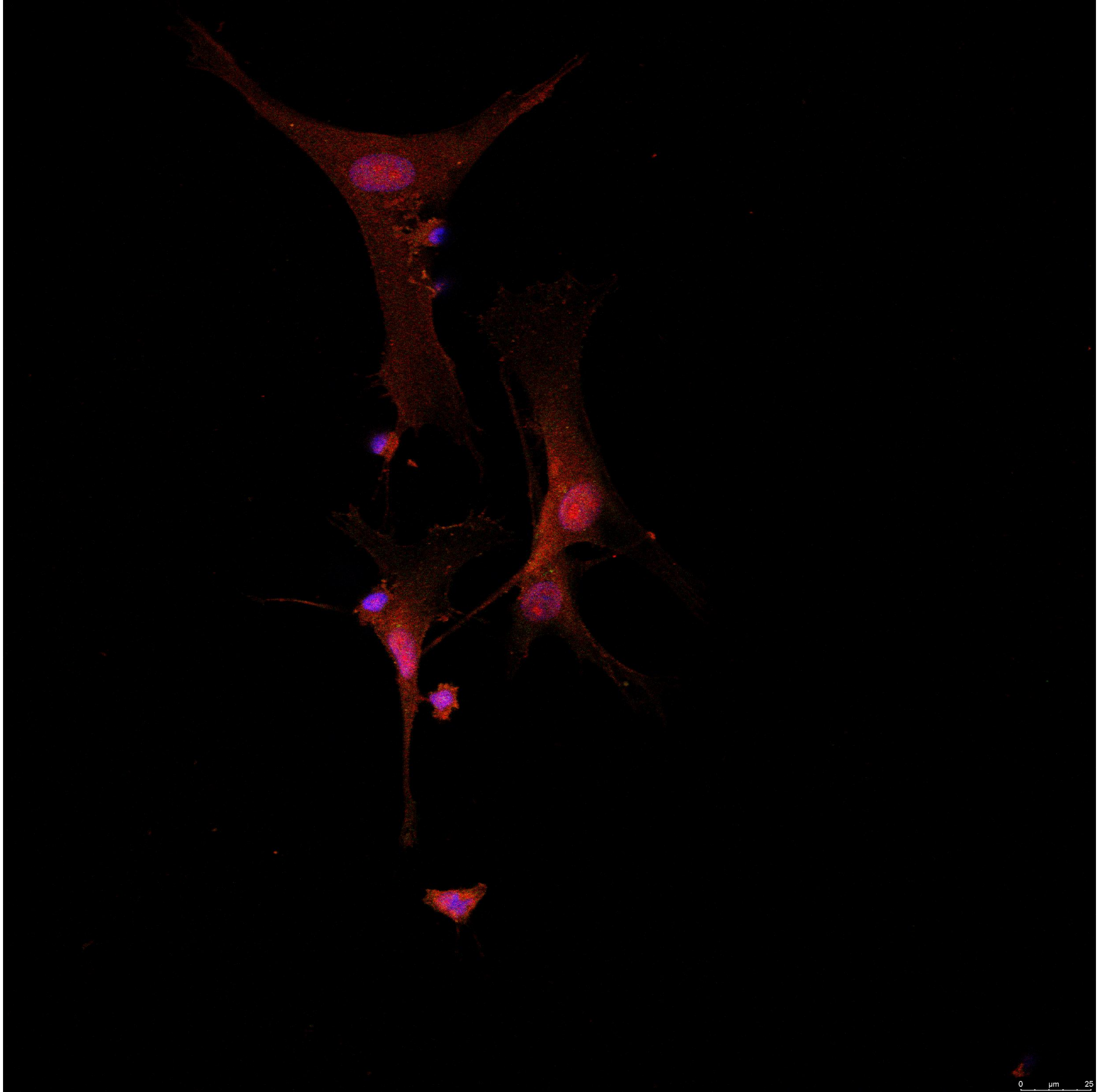

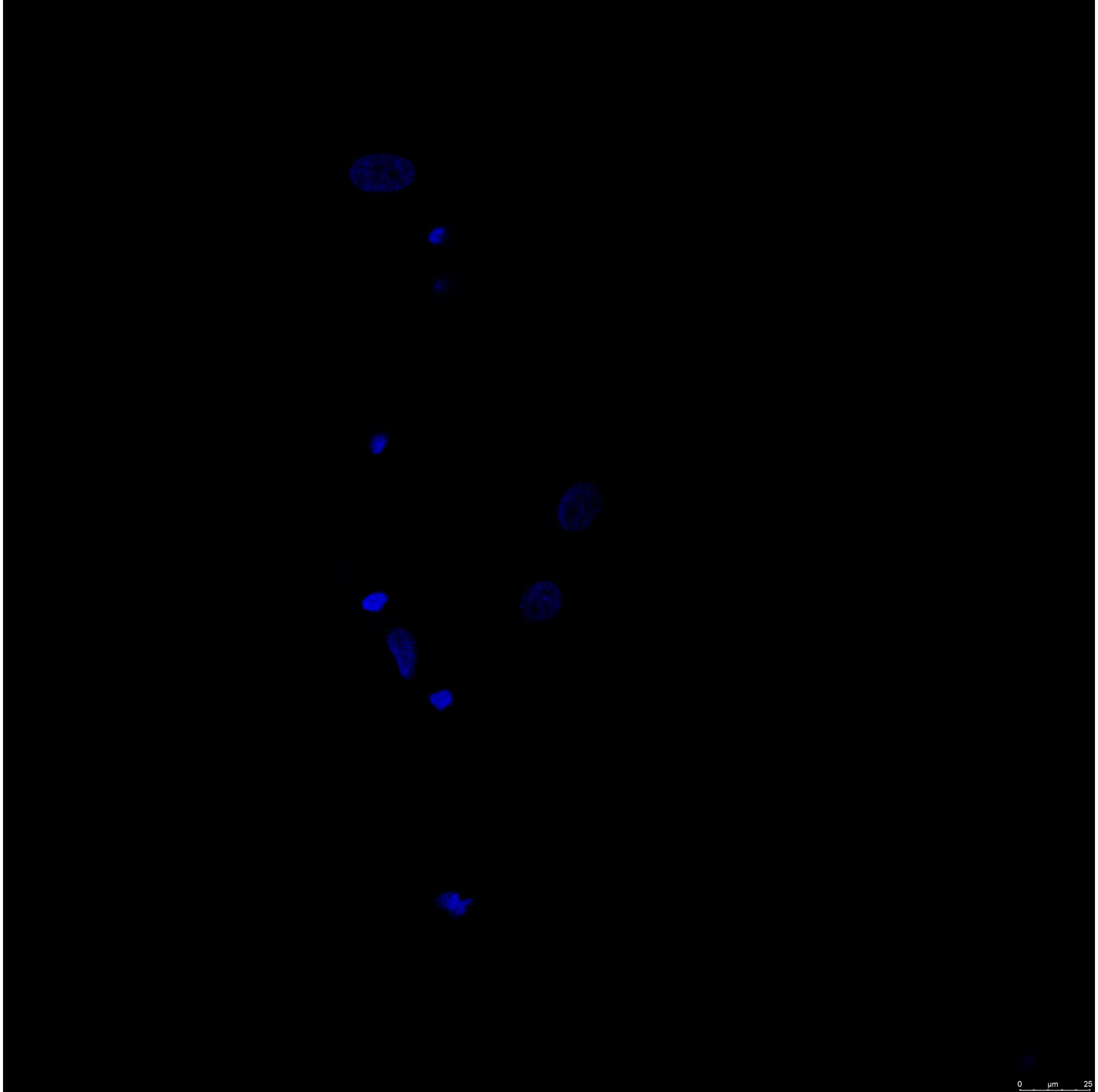
0 µm 25

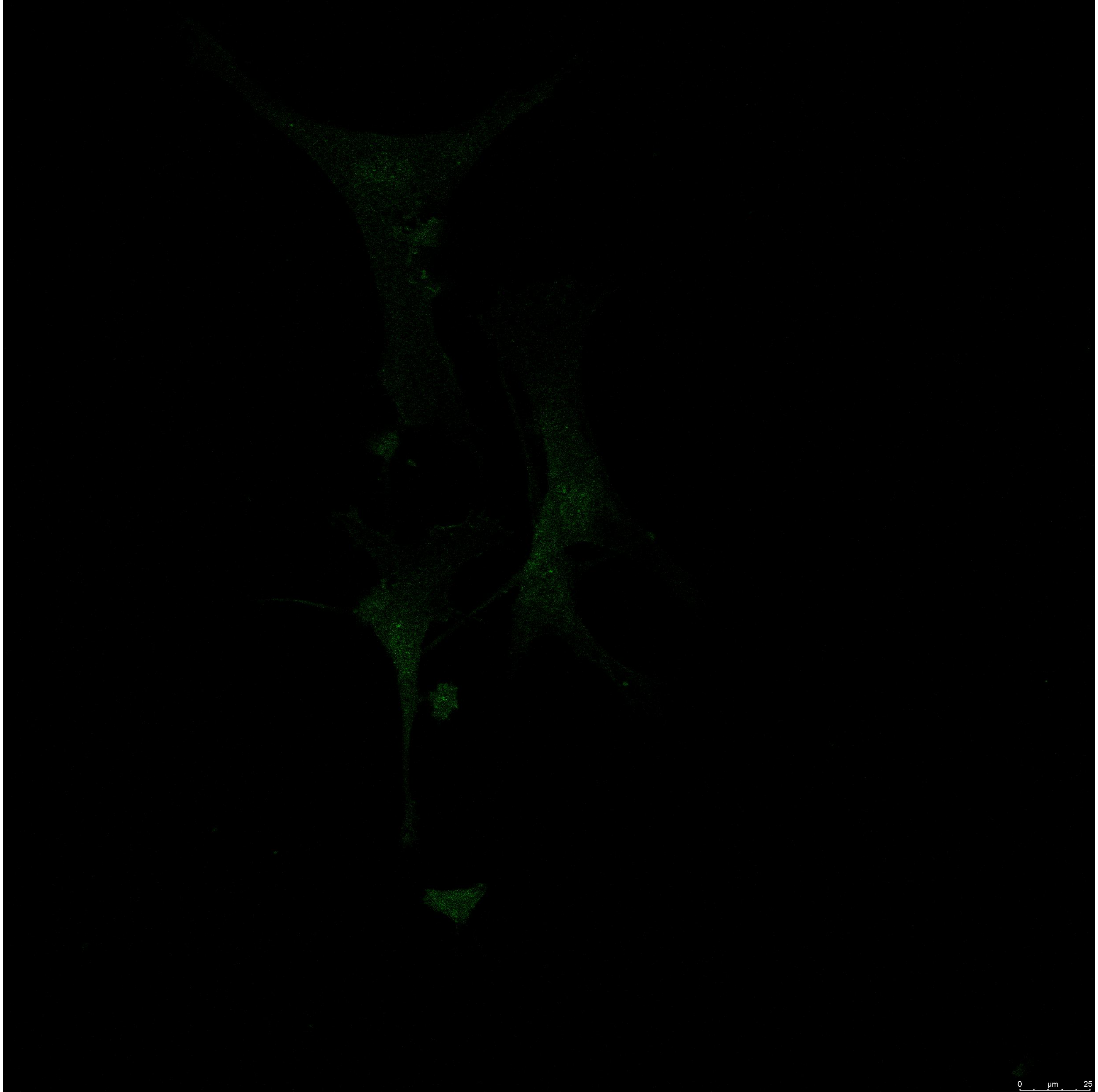
0 µm 25

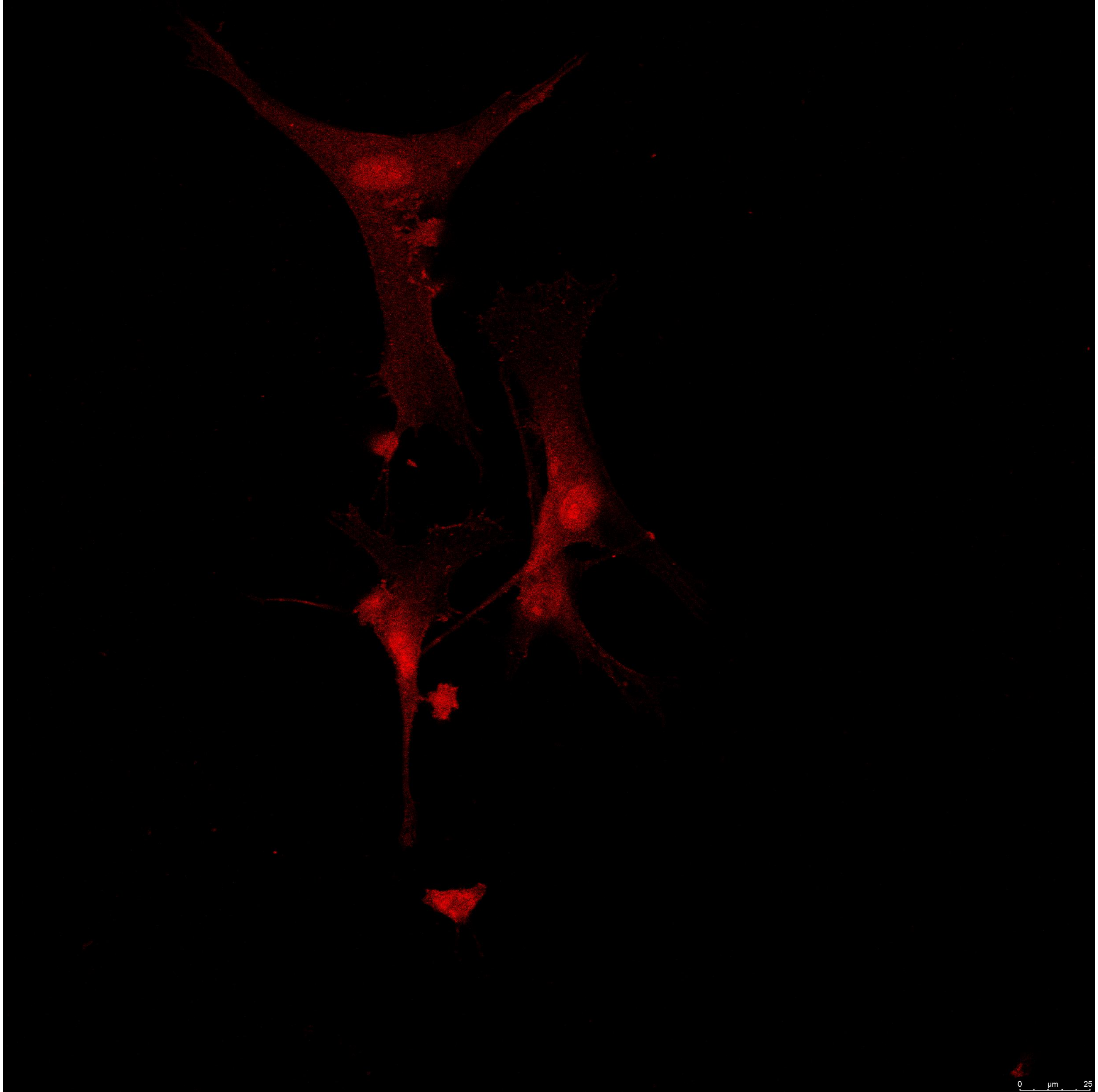
0 µm 25

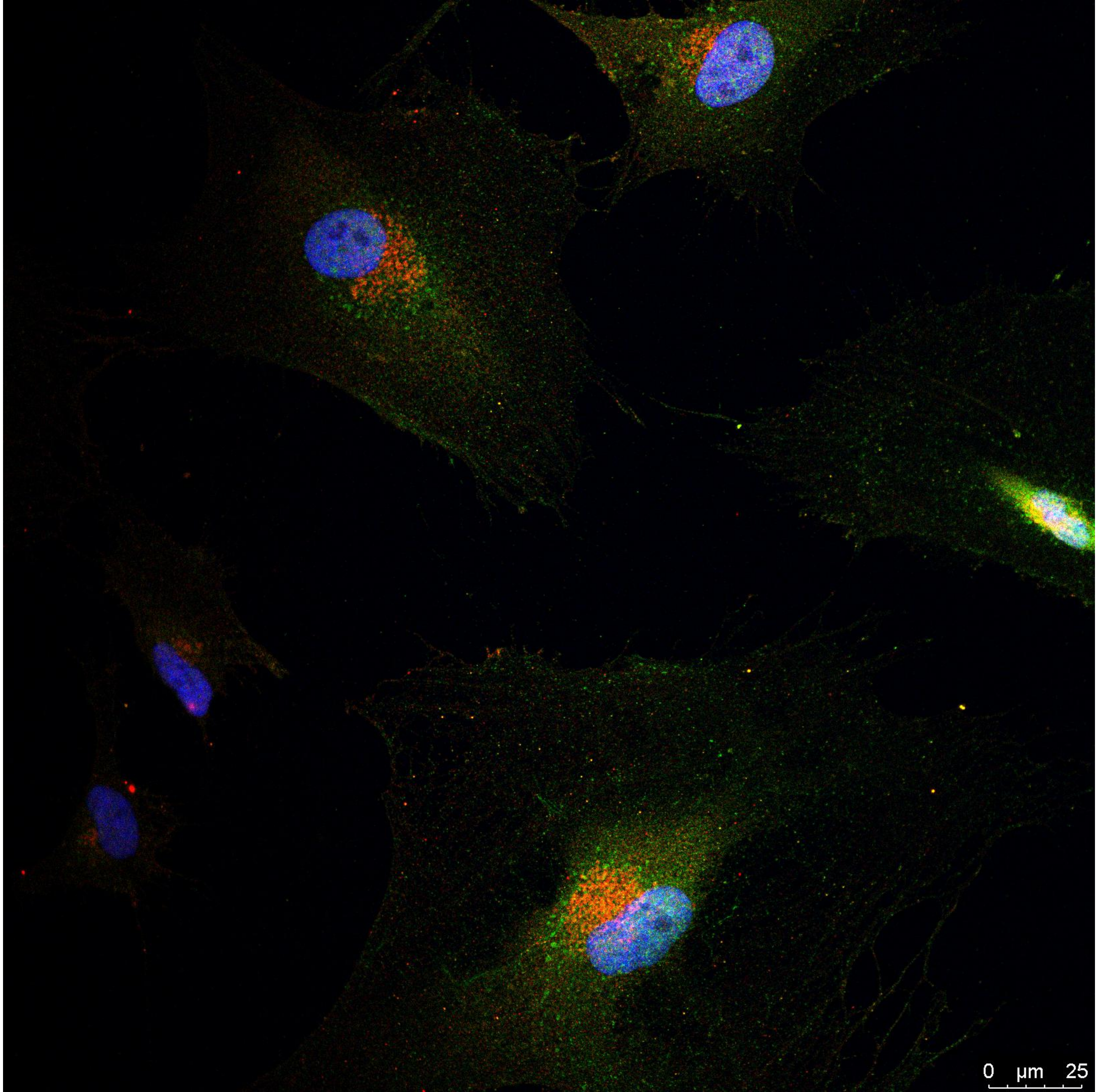
0 µm 25

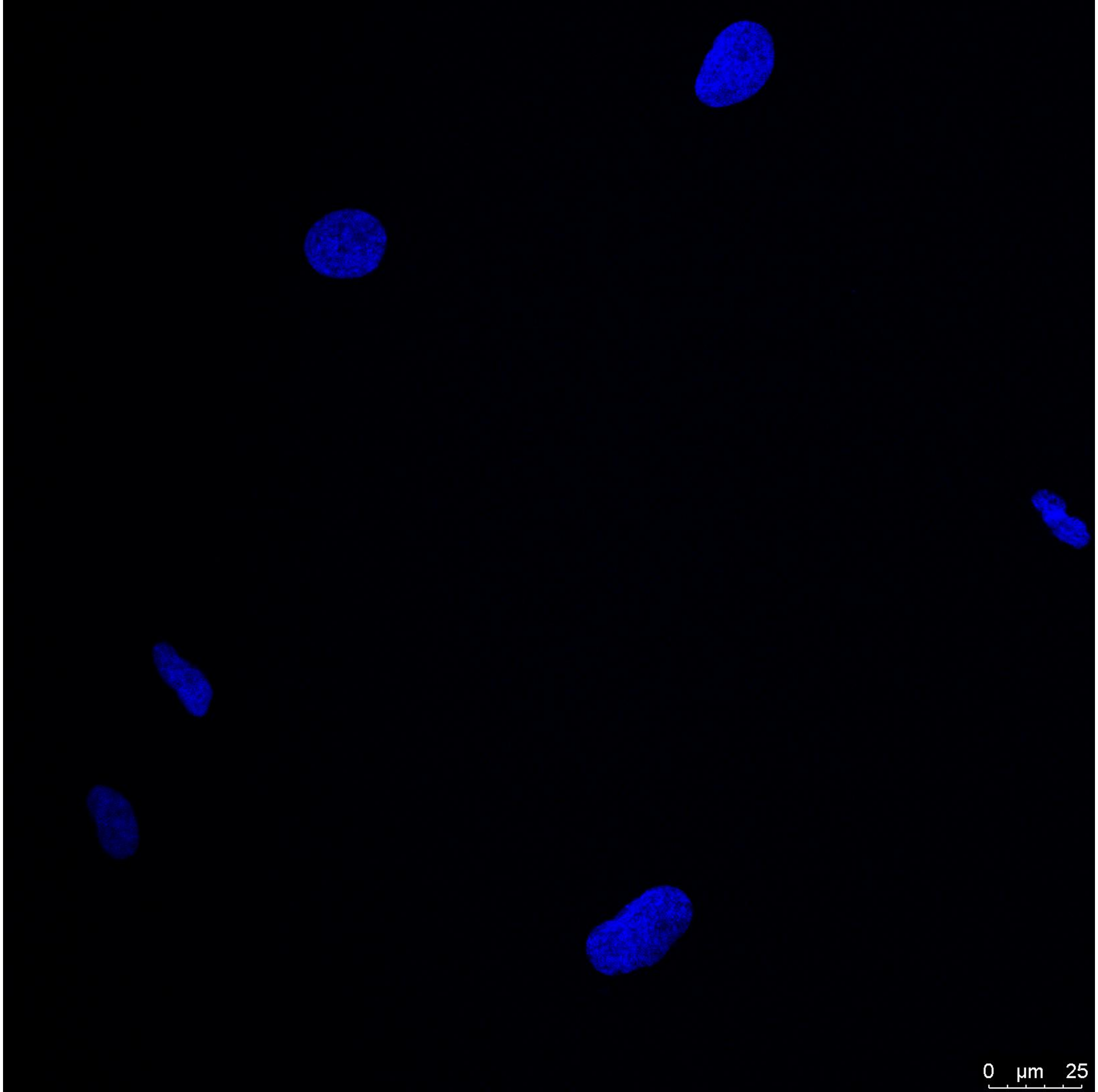
0 µm 25

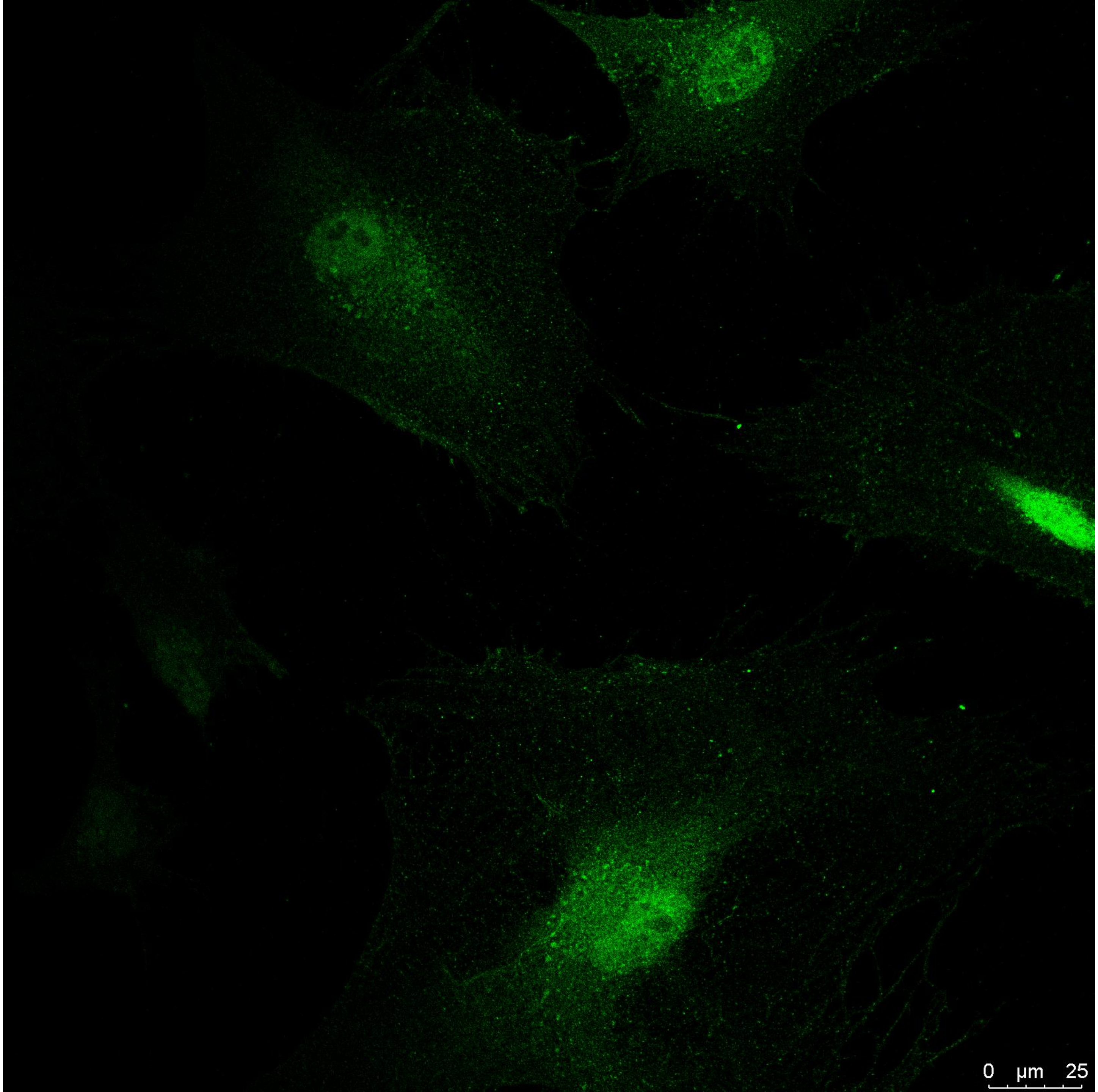
0 µm 25

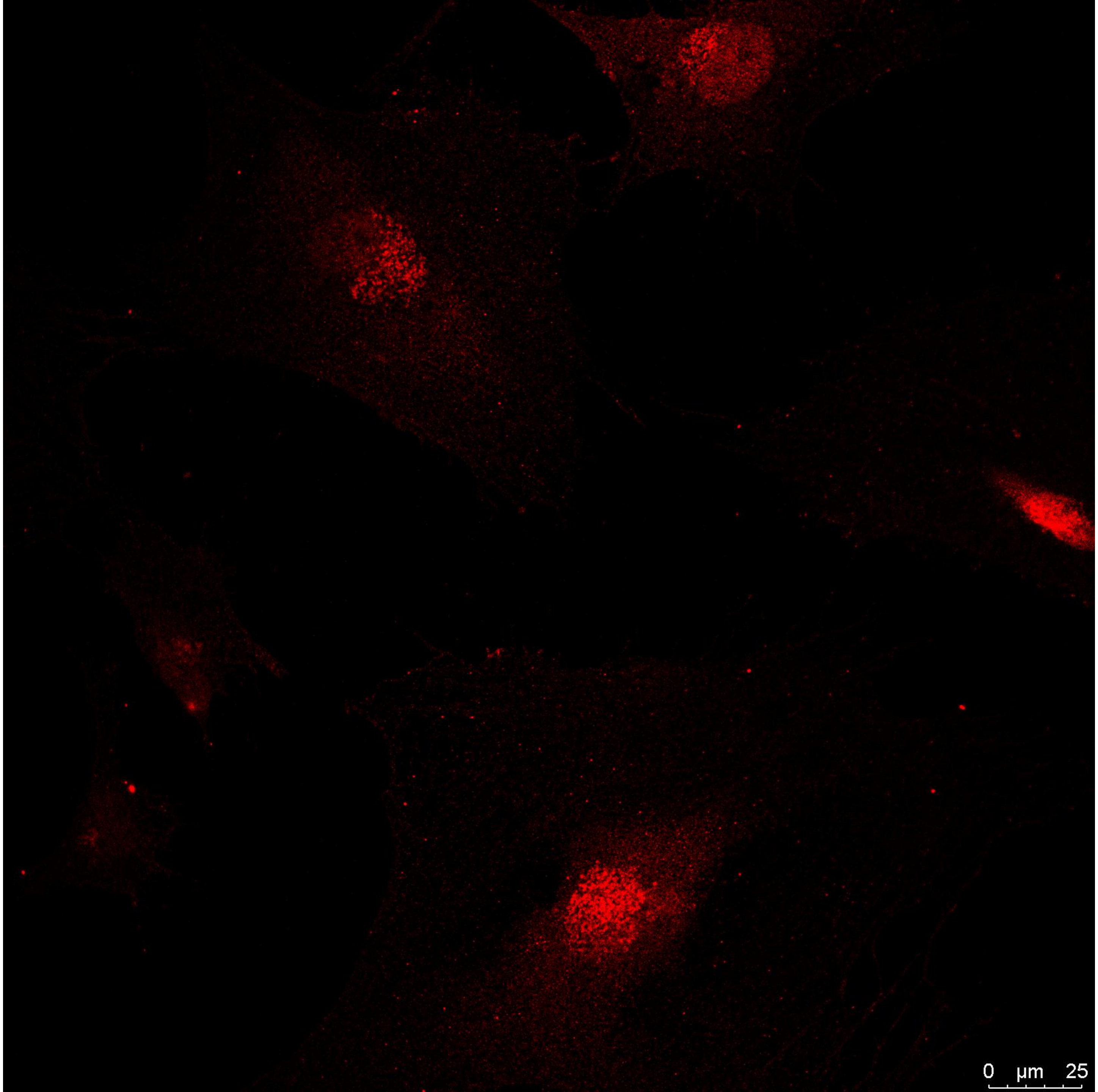
0 µm 25

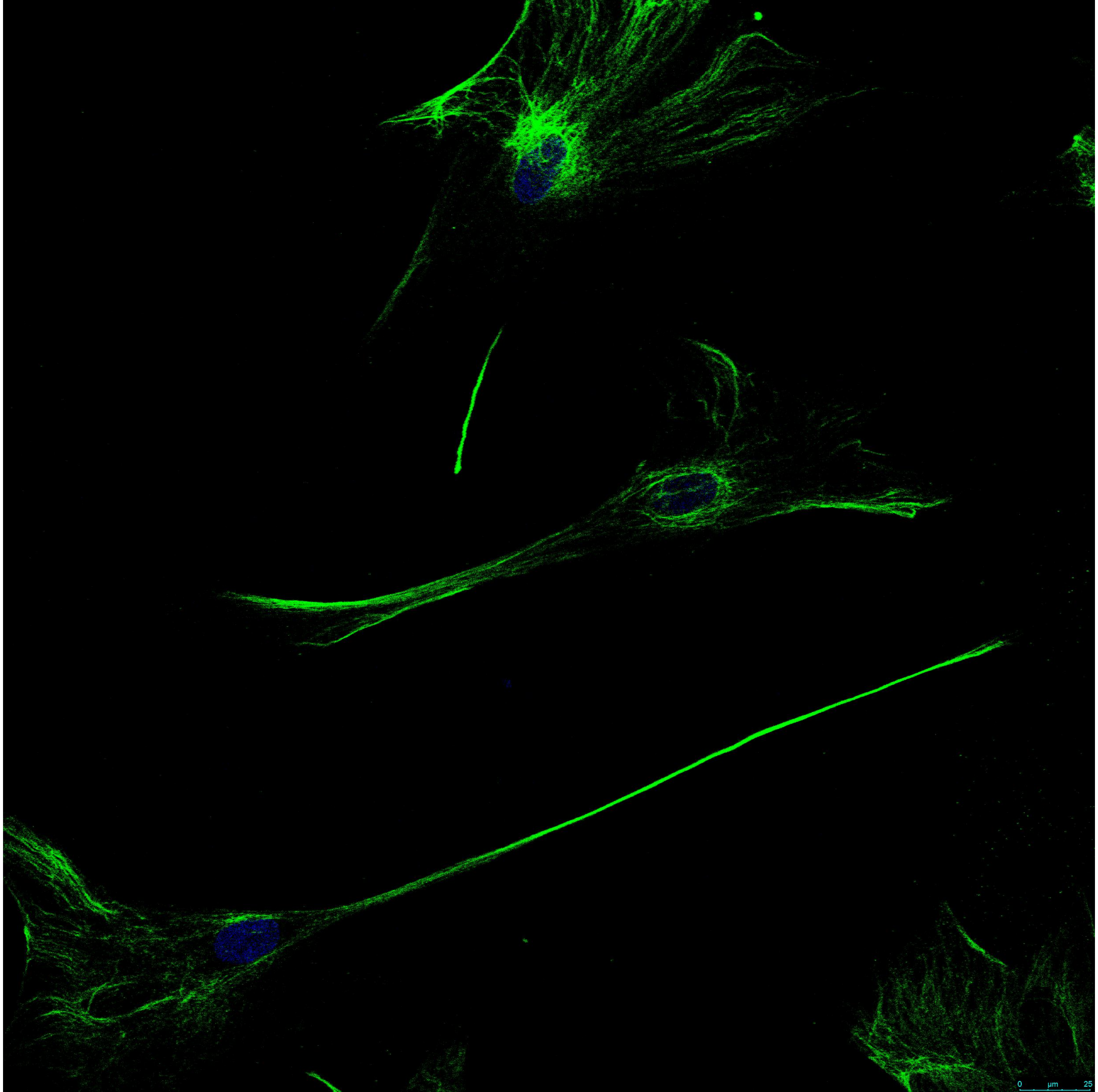
0 µm 25

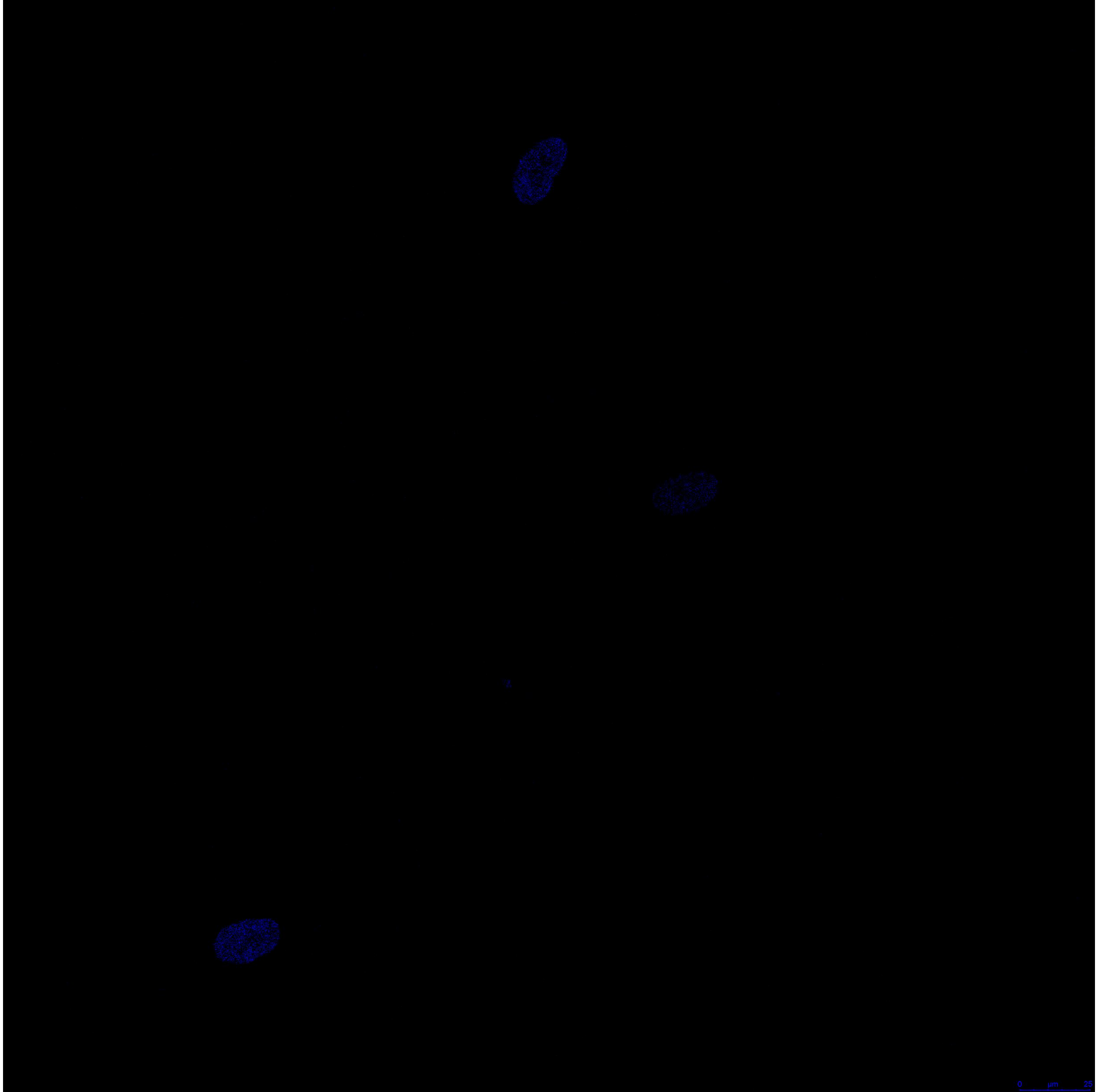
0 µm 25

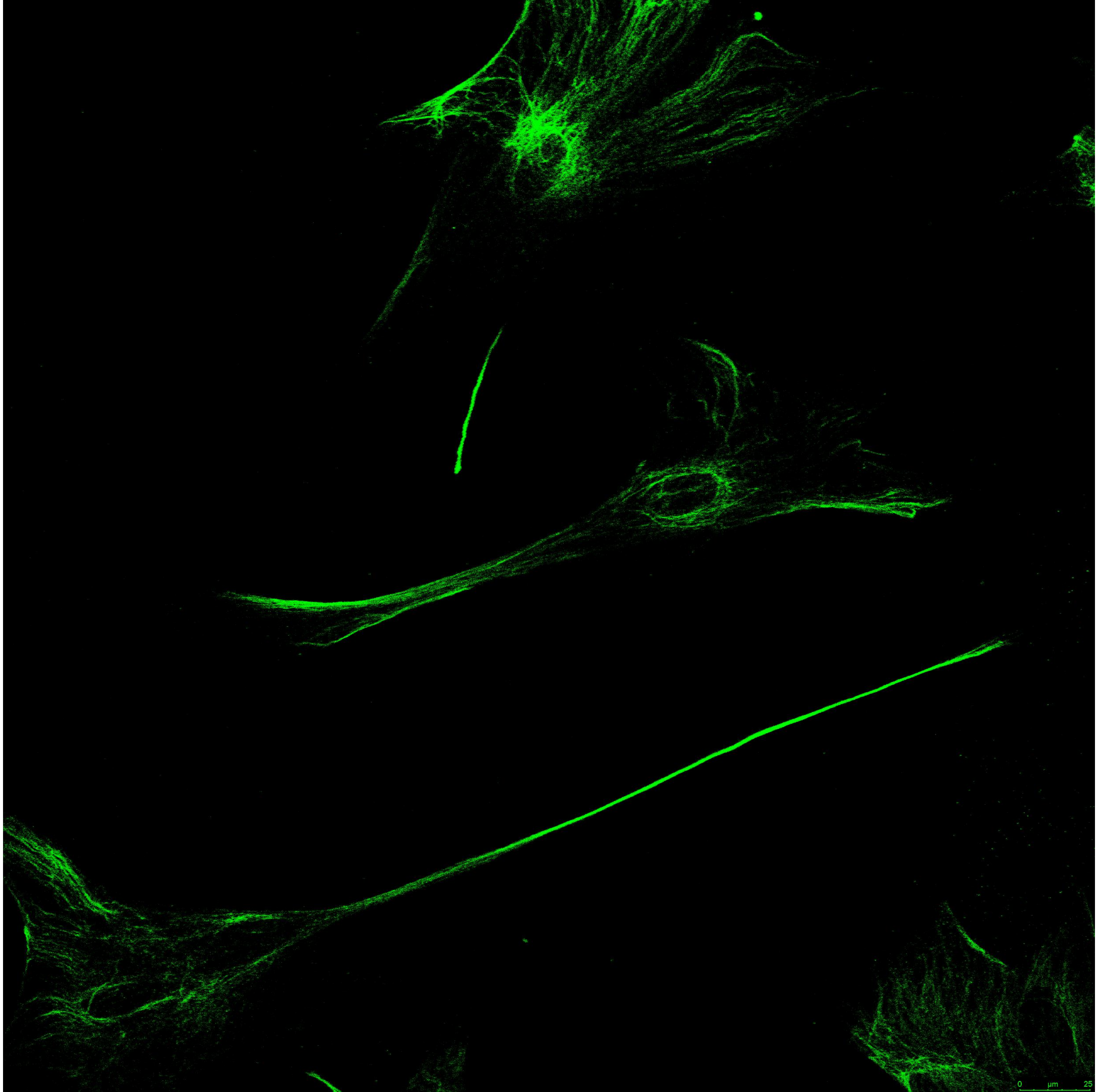
0
µm
25

Figure 3G

MFN1

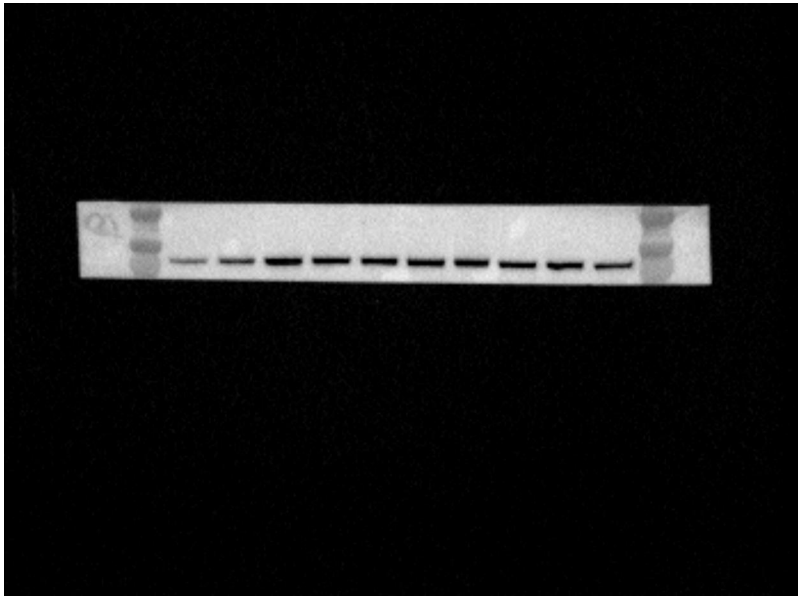

Figure 3G

OPA1

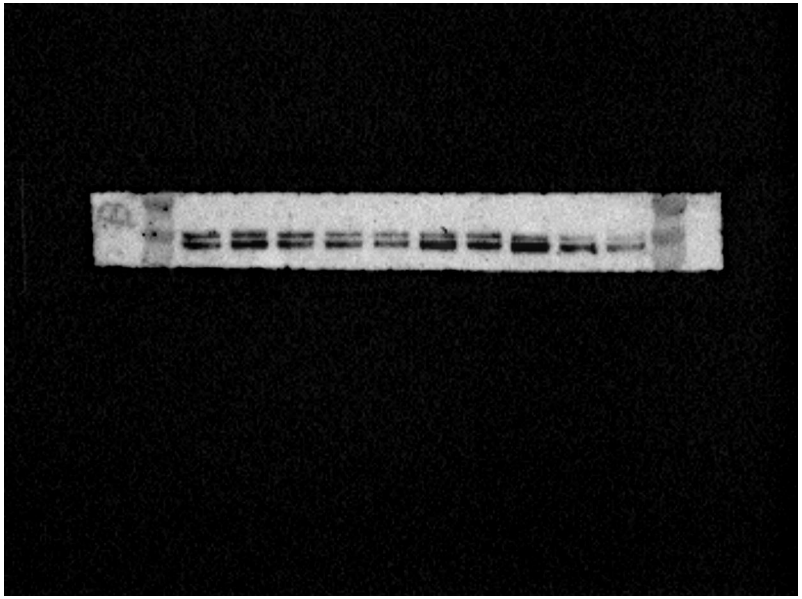

Figure 3G

MFF

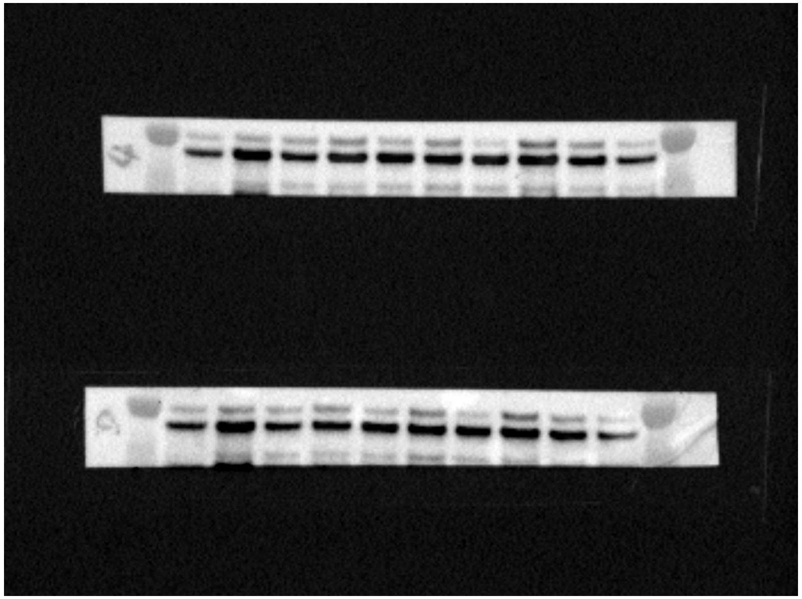

Figure 3G

P-DRP1 616

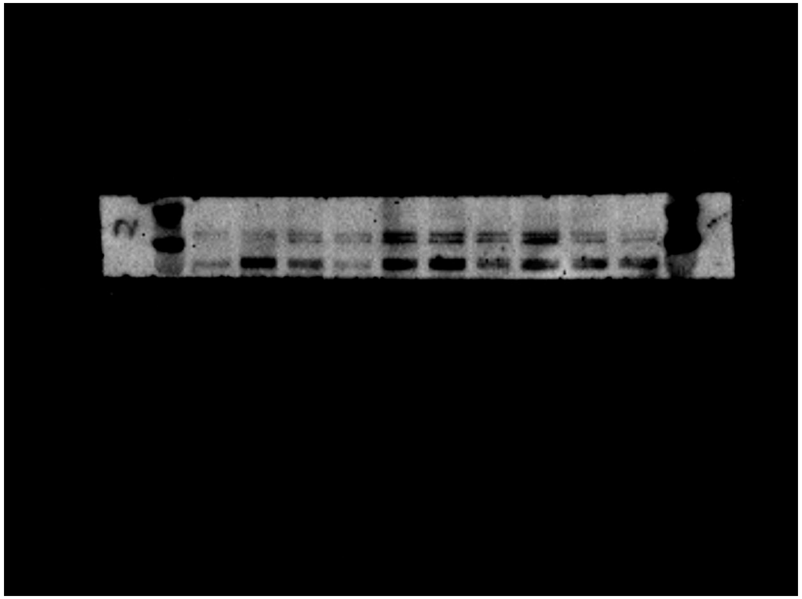

Figure 3G

DRP1

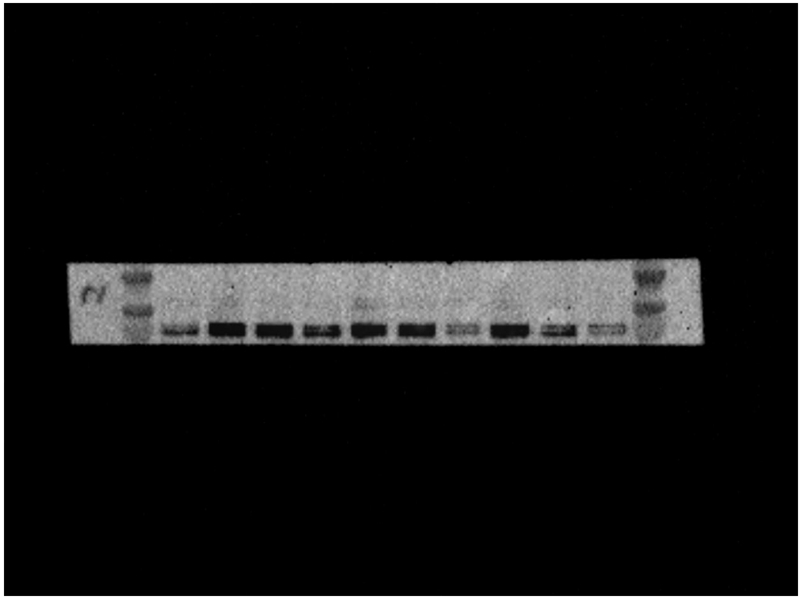

Figure 3G

GAPDH

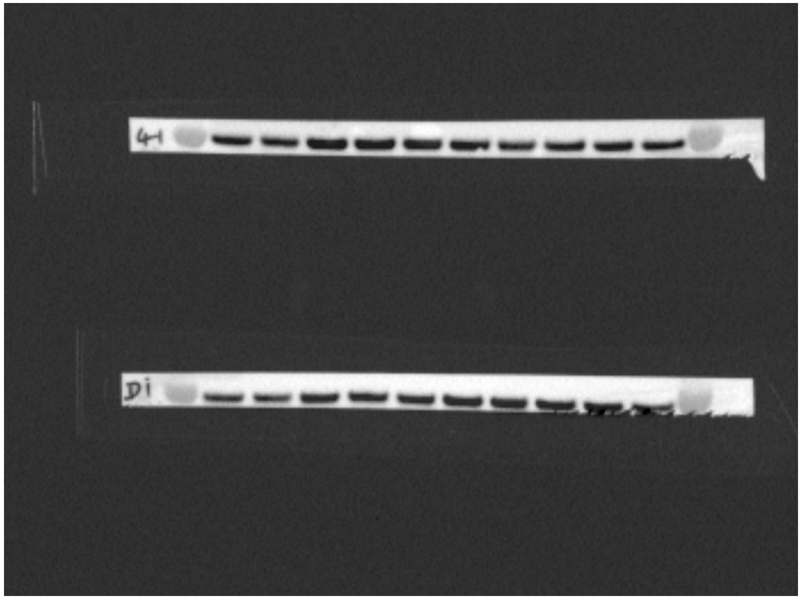

Figure 3G

ALL GEL-1

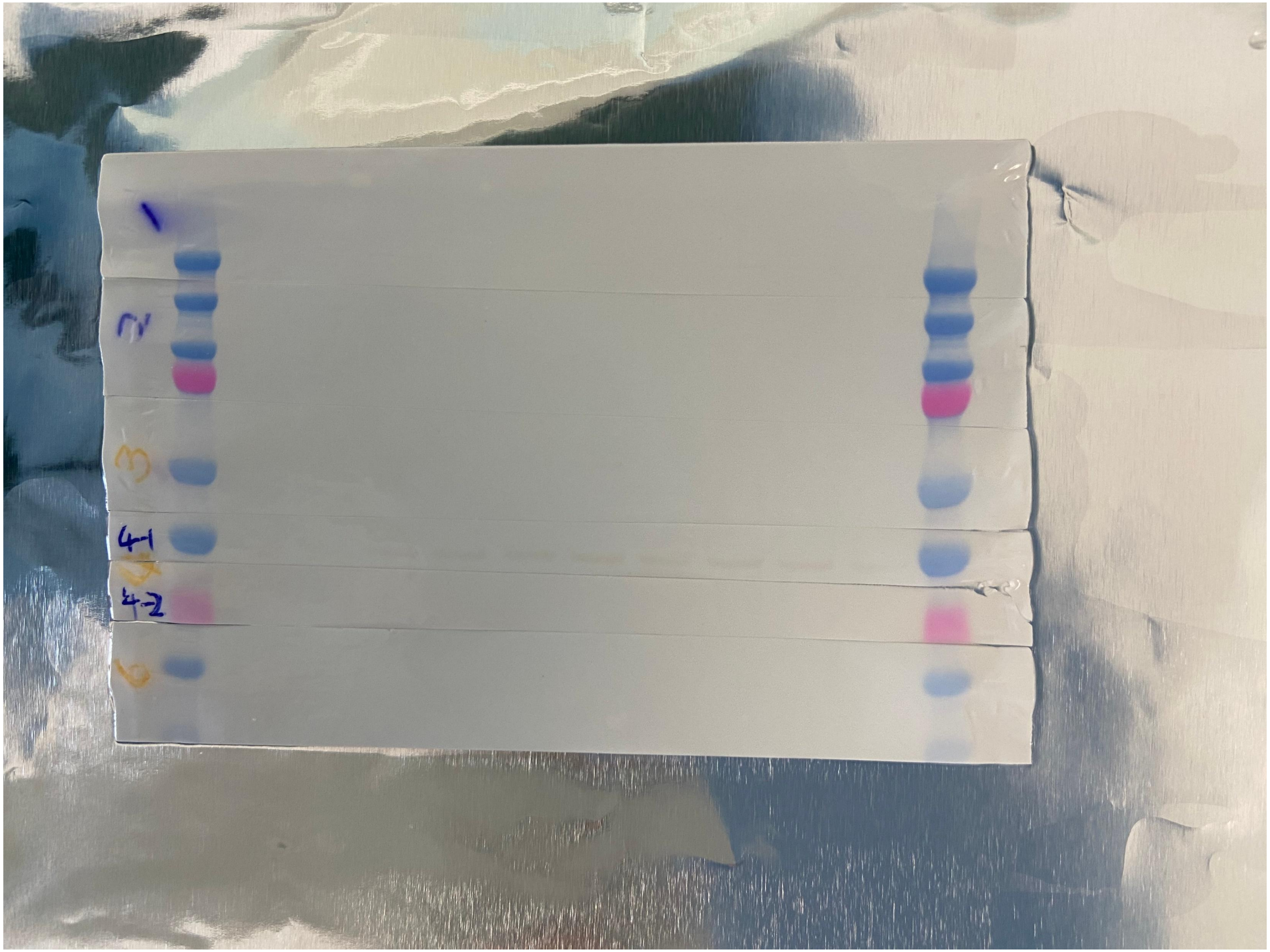

Figure 3G

ALL GEL-2

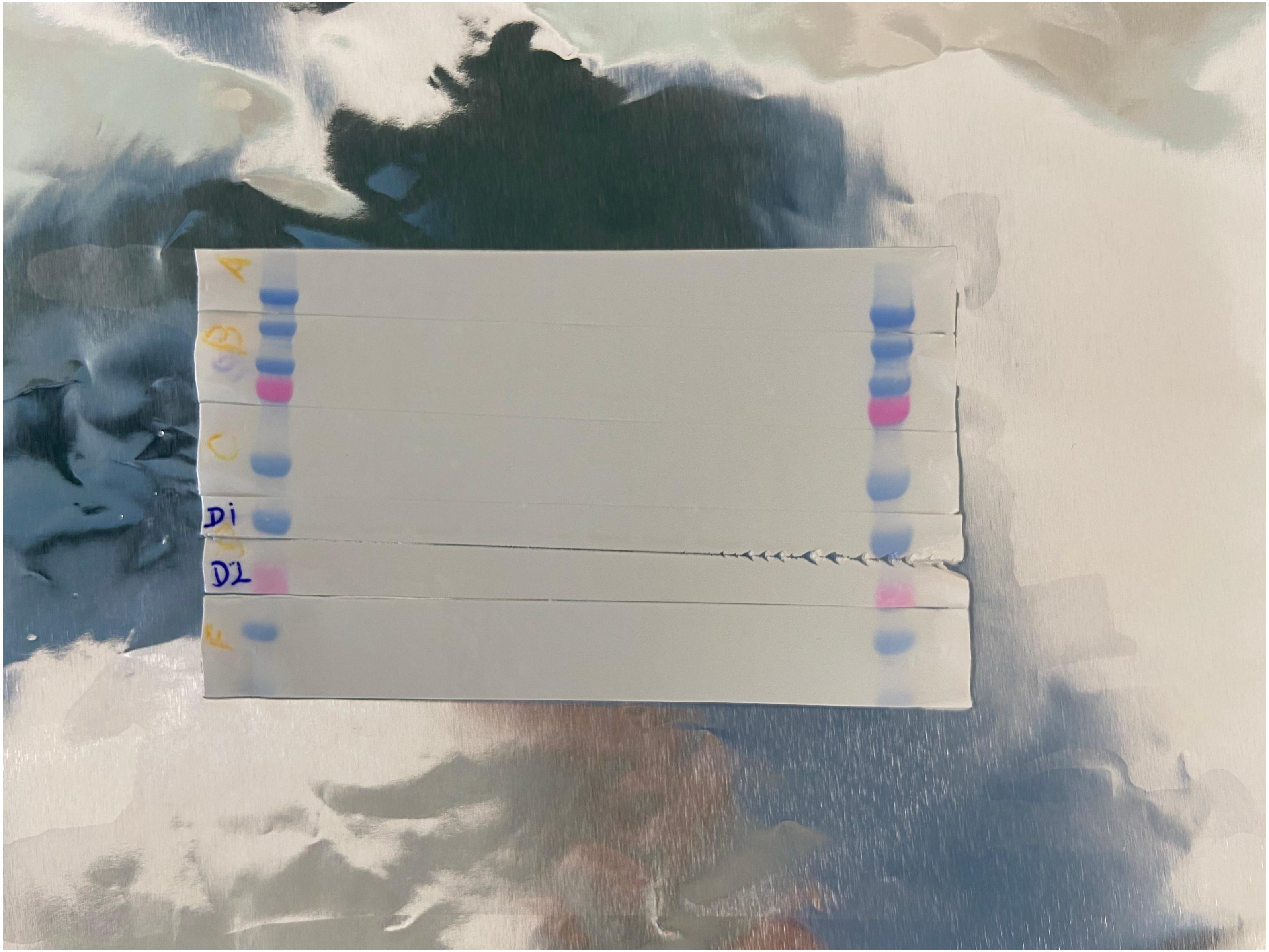

Figure 3H
CTRL

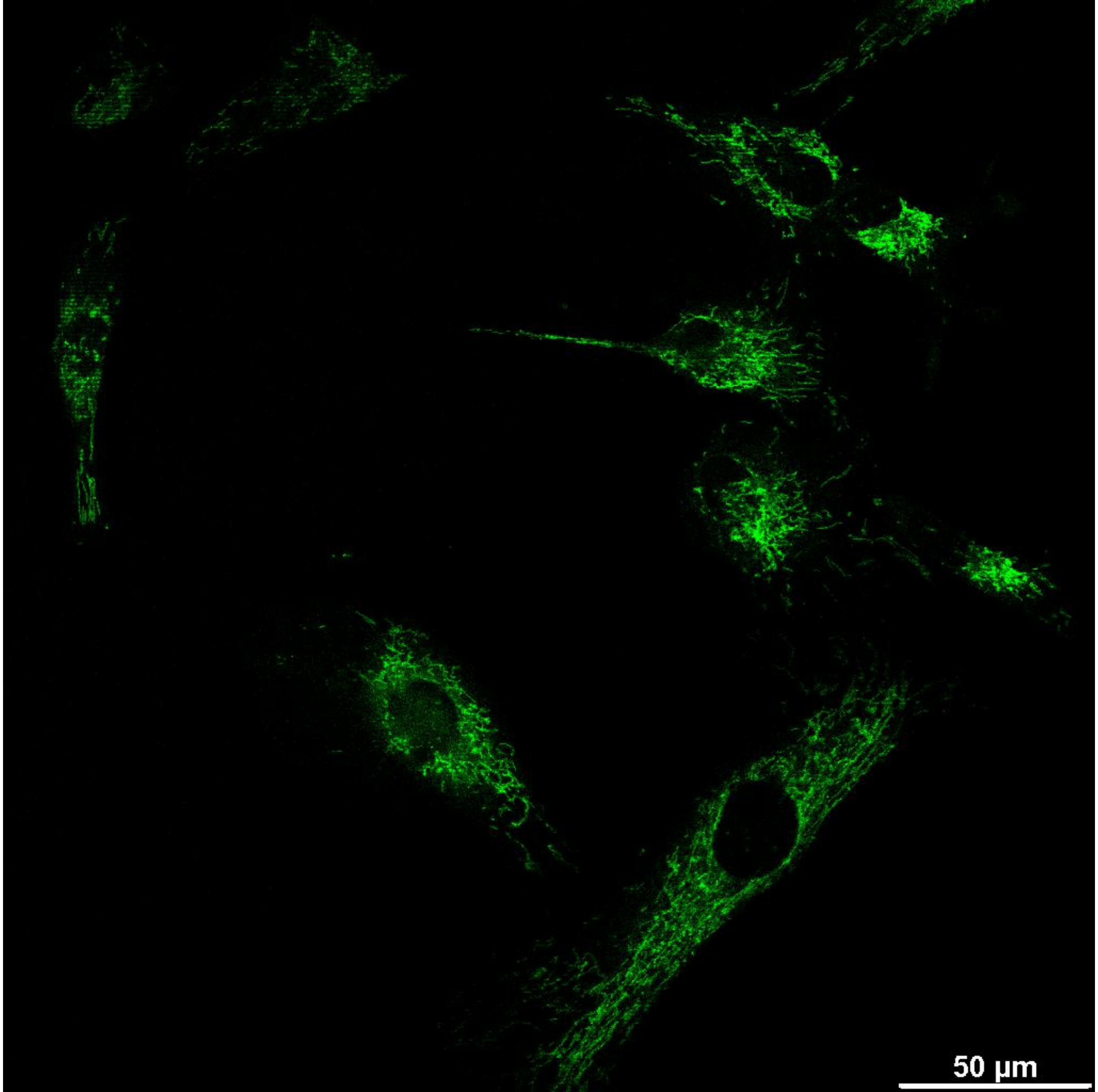

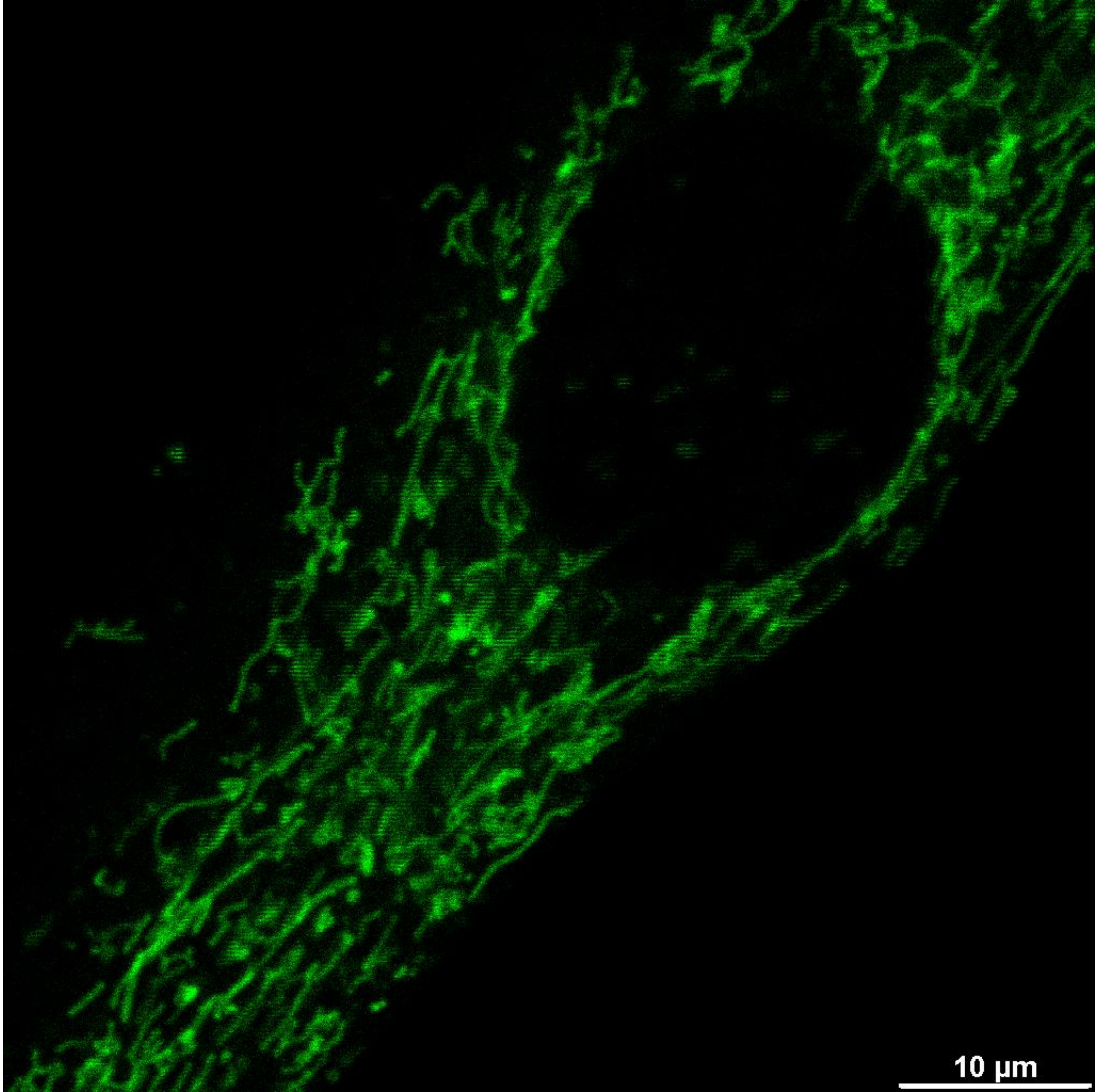
10 µm

Figure 3H
WS5A

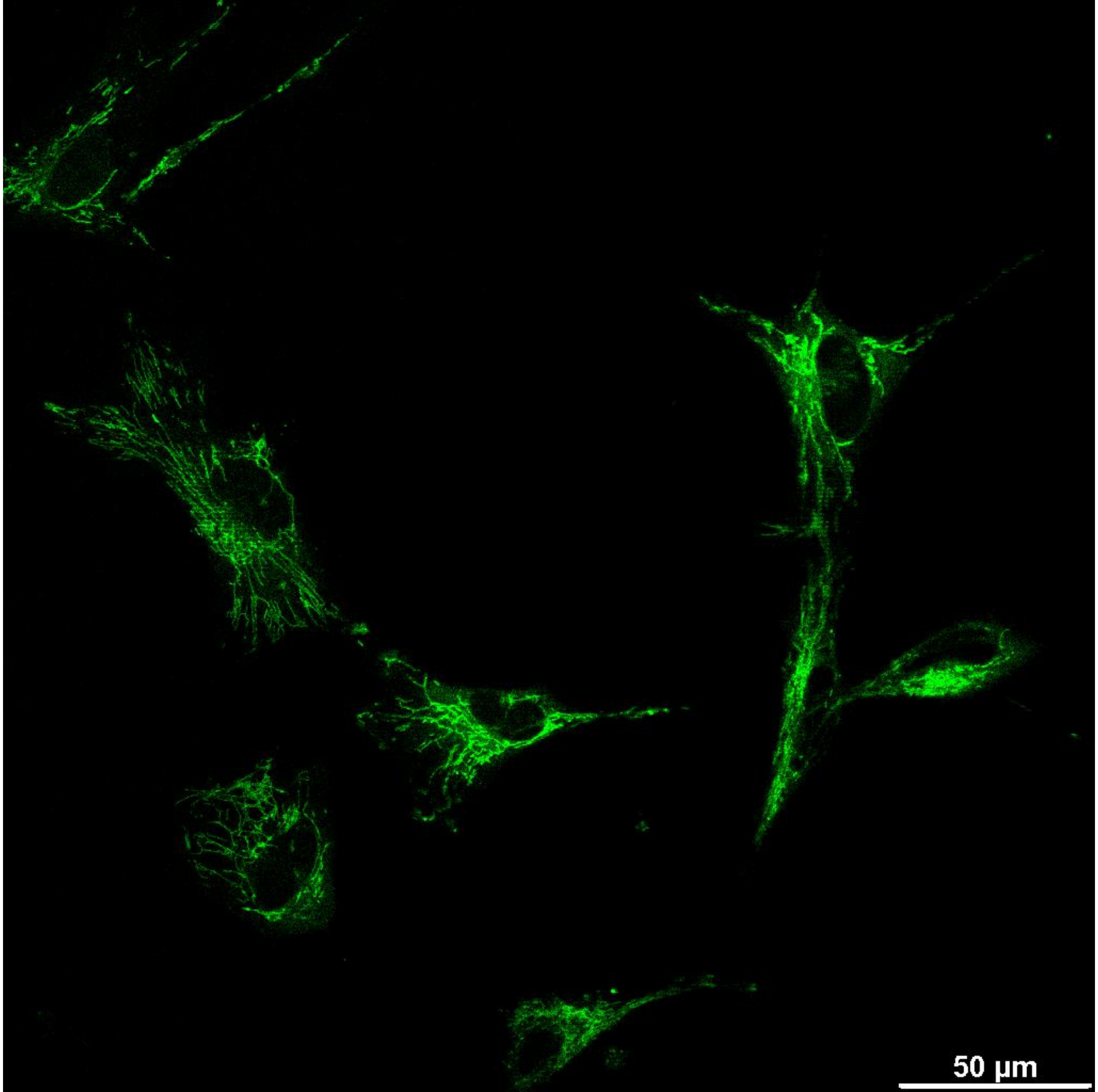

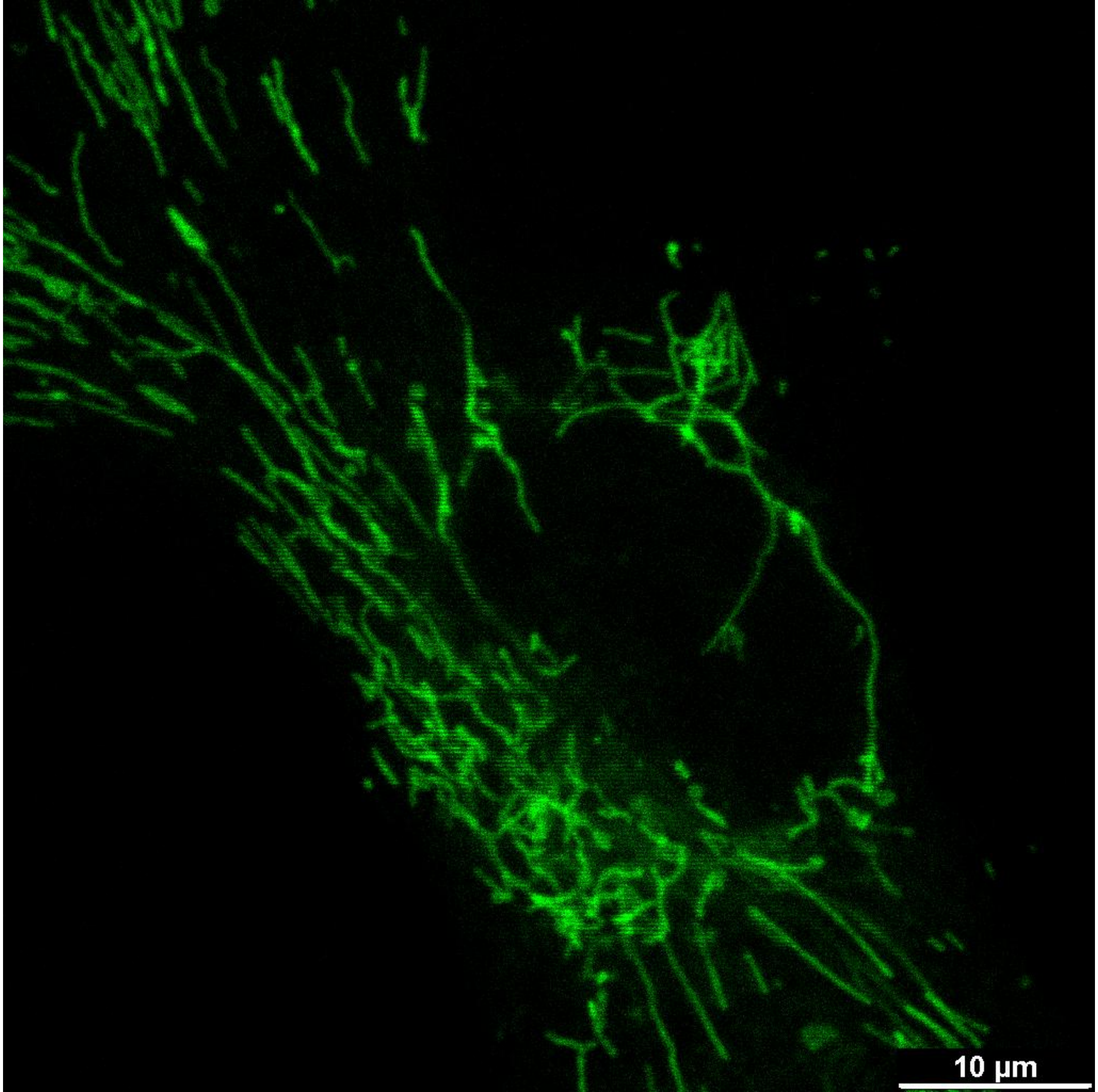
10 µm

Figure 3H
CP2A

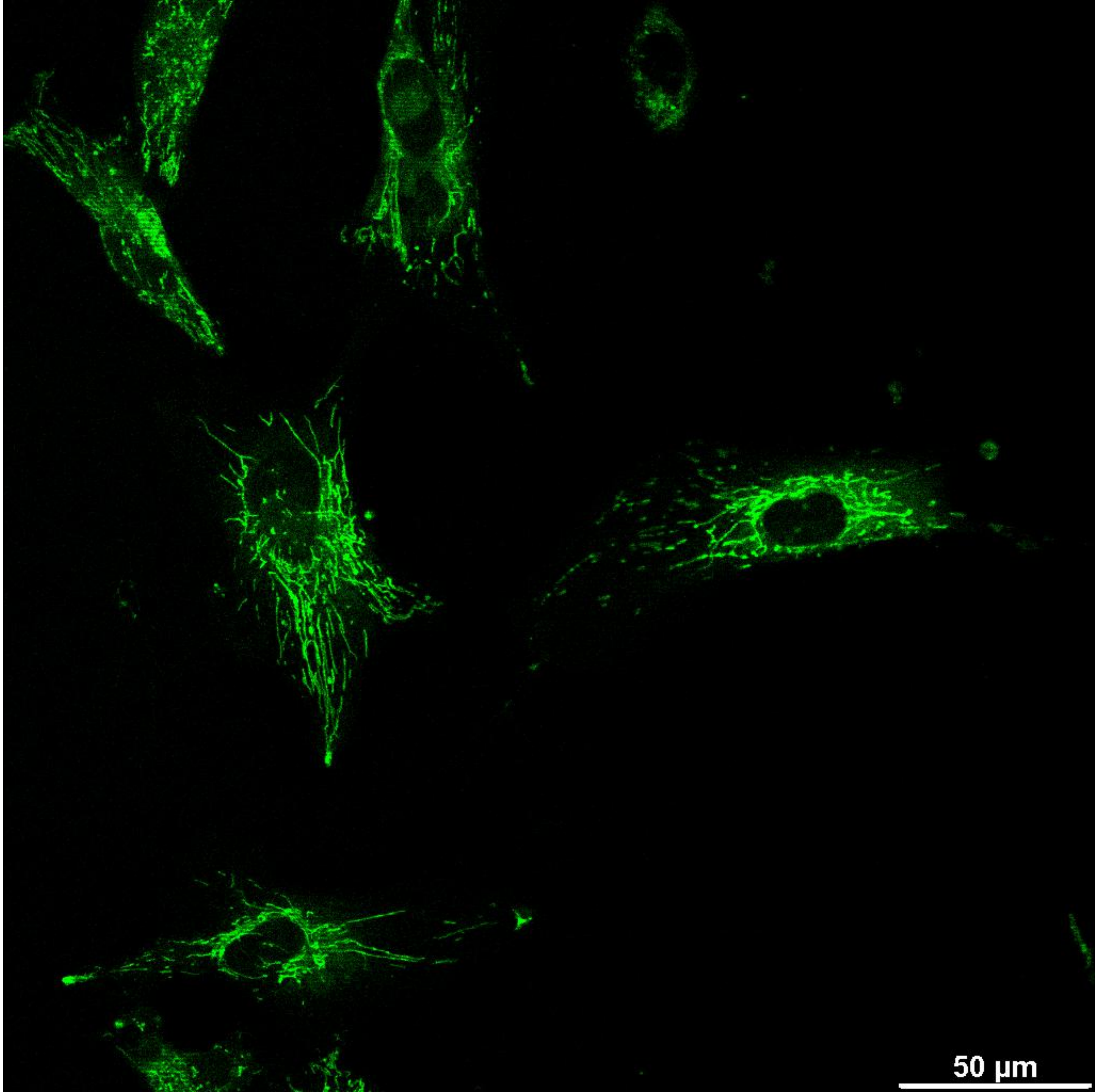

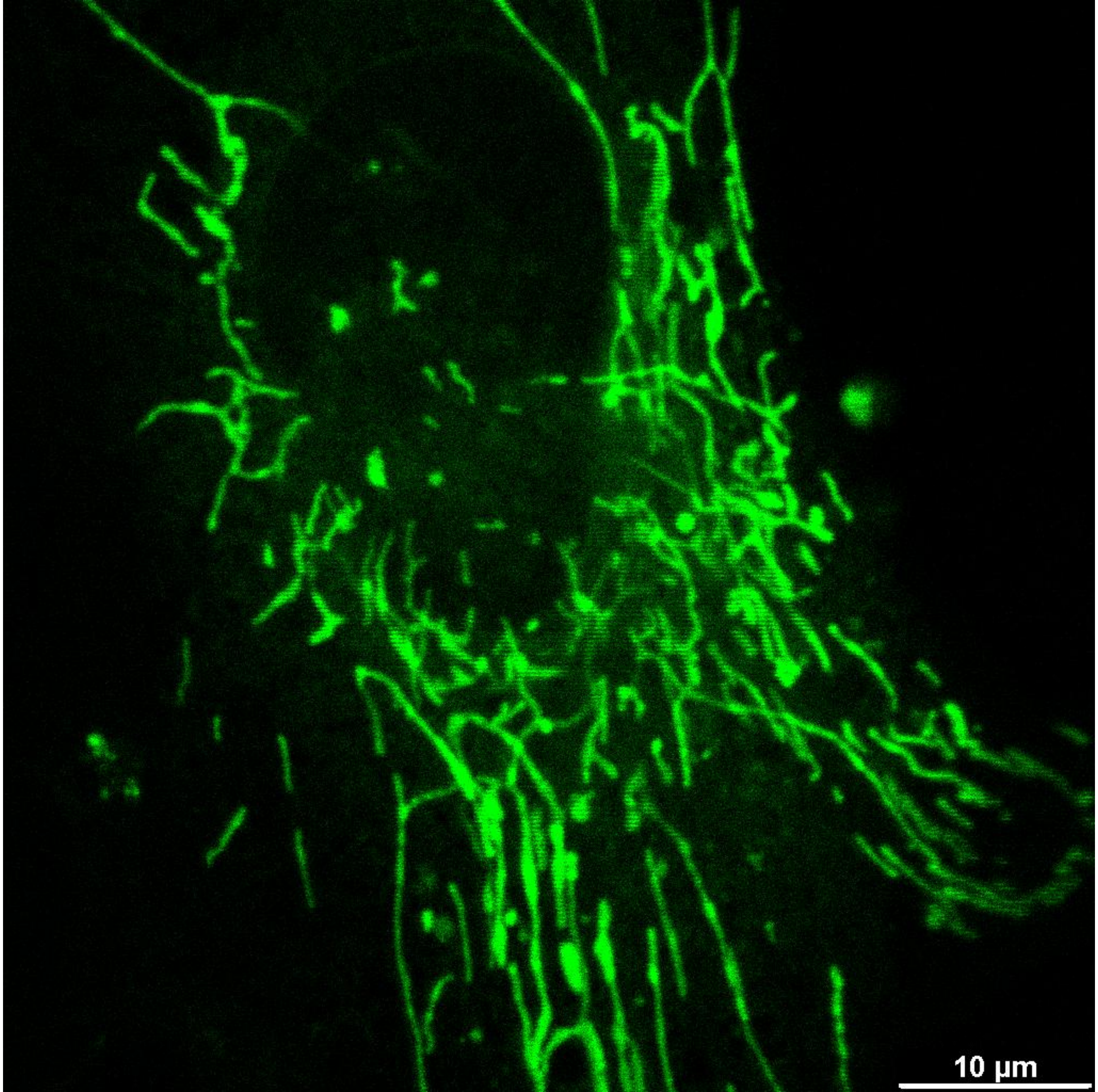
10 µm

Figure 3K

TOMM20

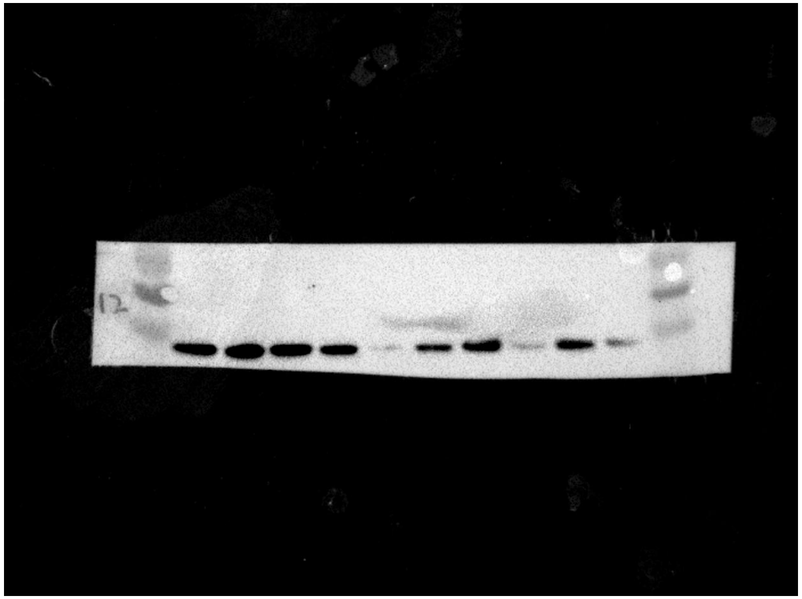

Figure 3K

PGC-1β

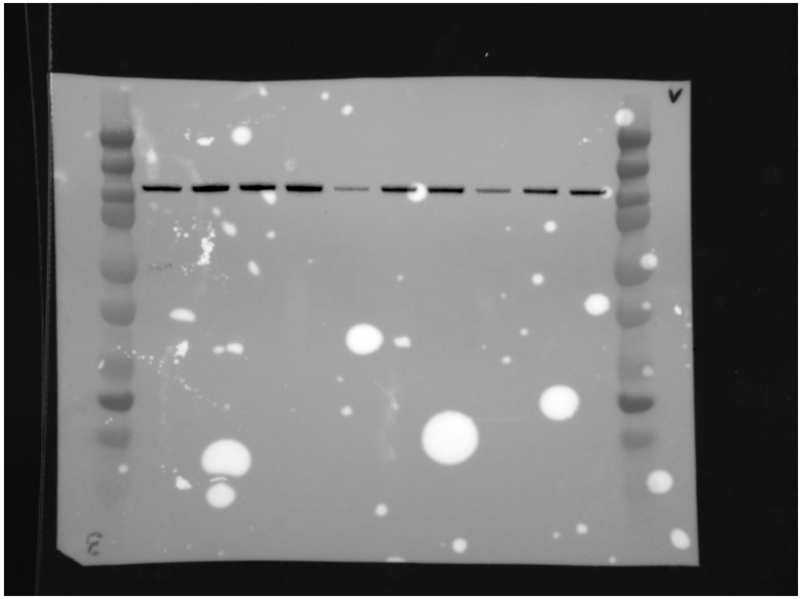

Figure 3K

GAPDH

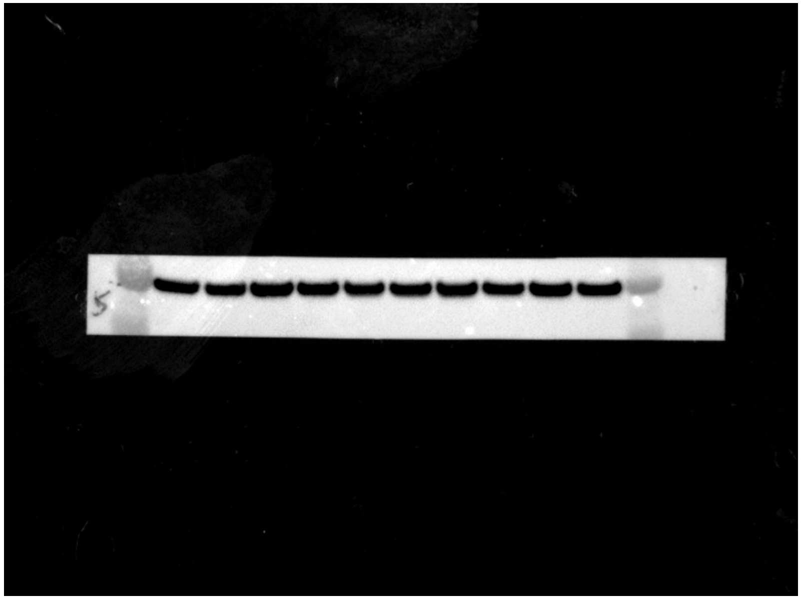

Figure 4A
CTRL

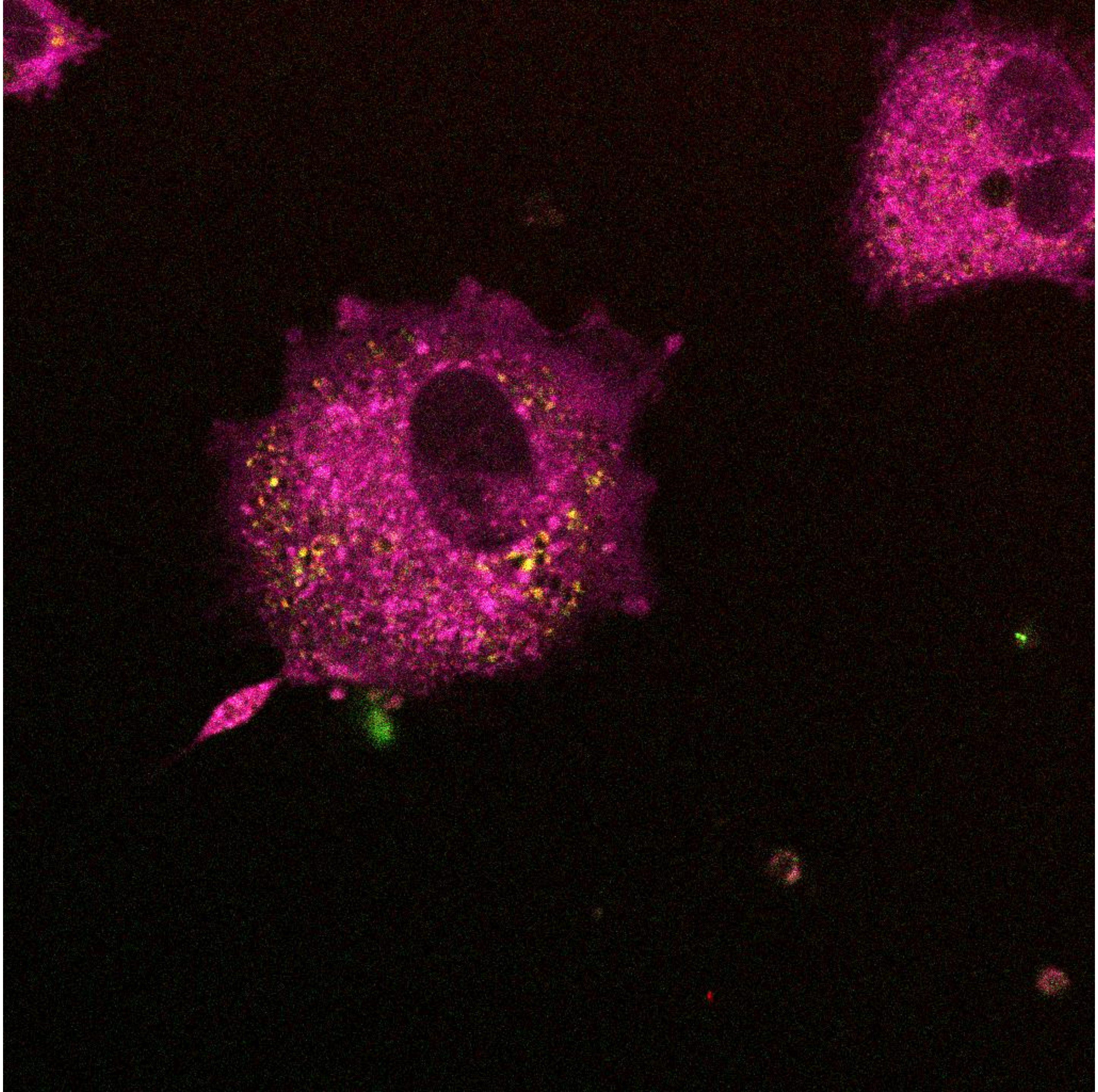

Figure 4A
CTRL

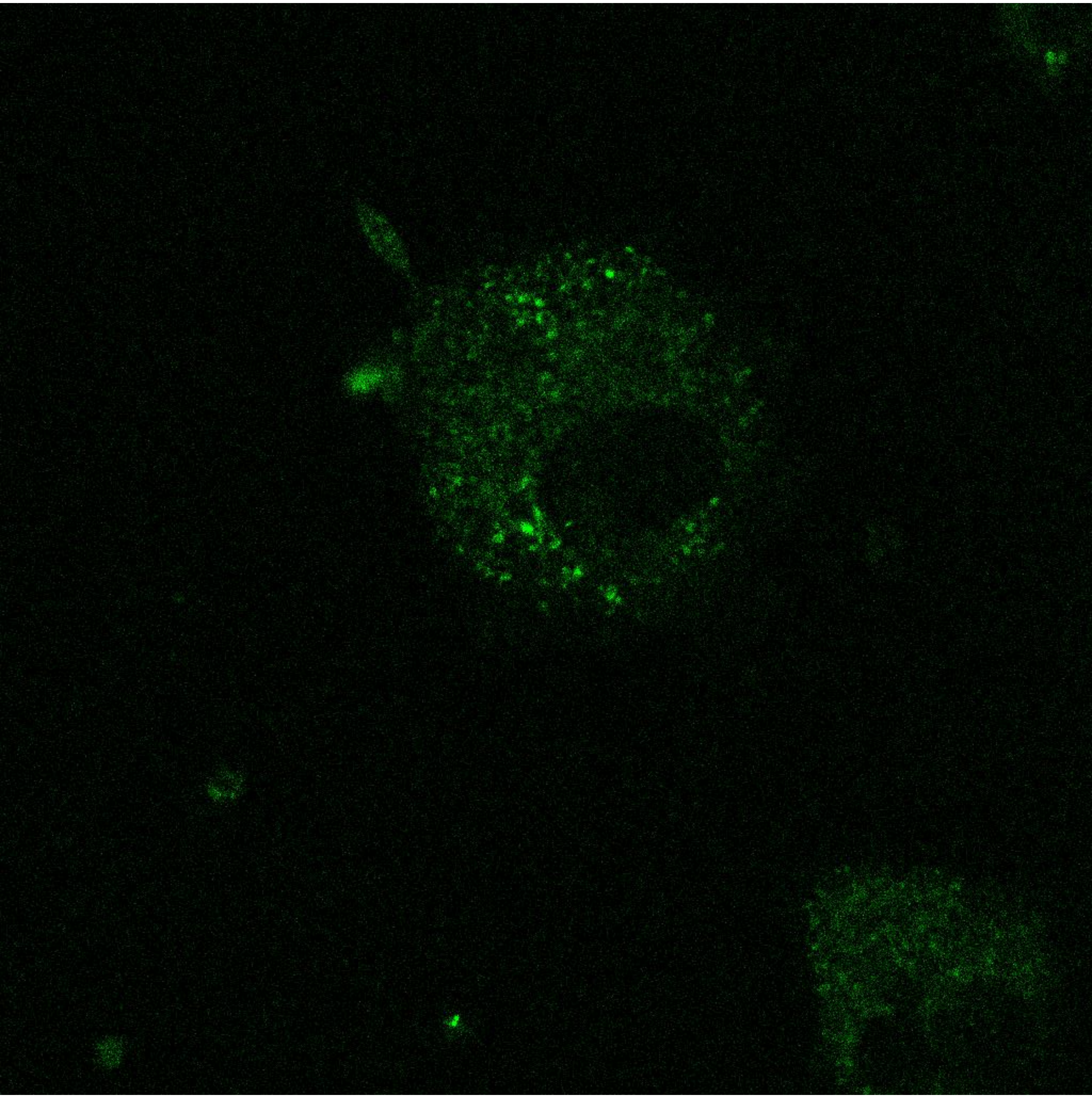

Figure 4A
CTRL

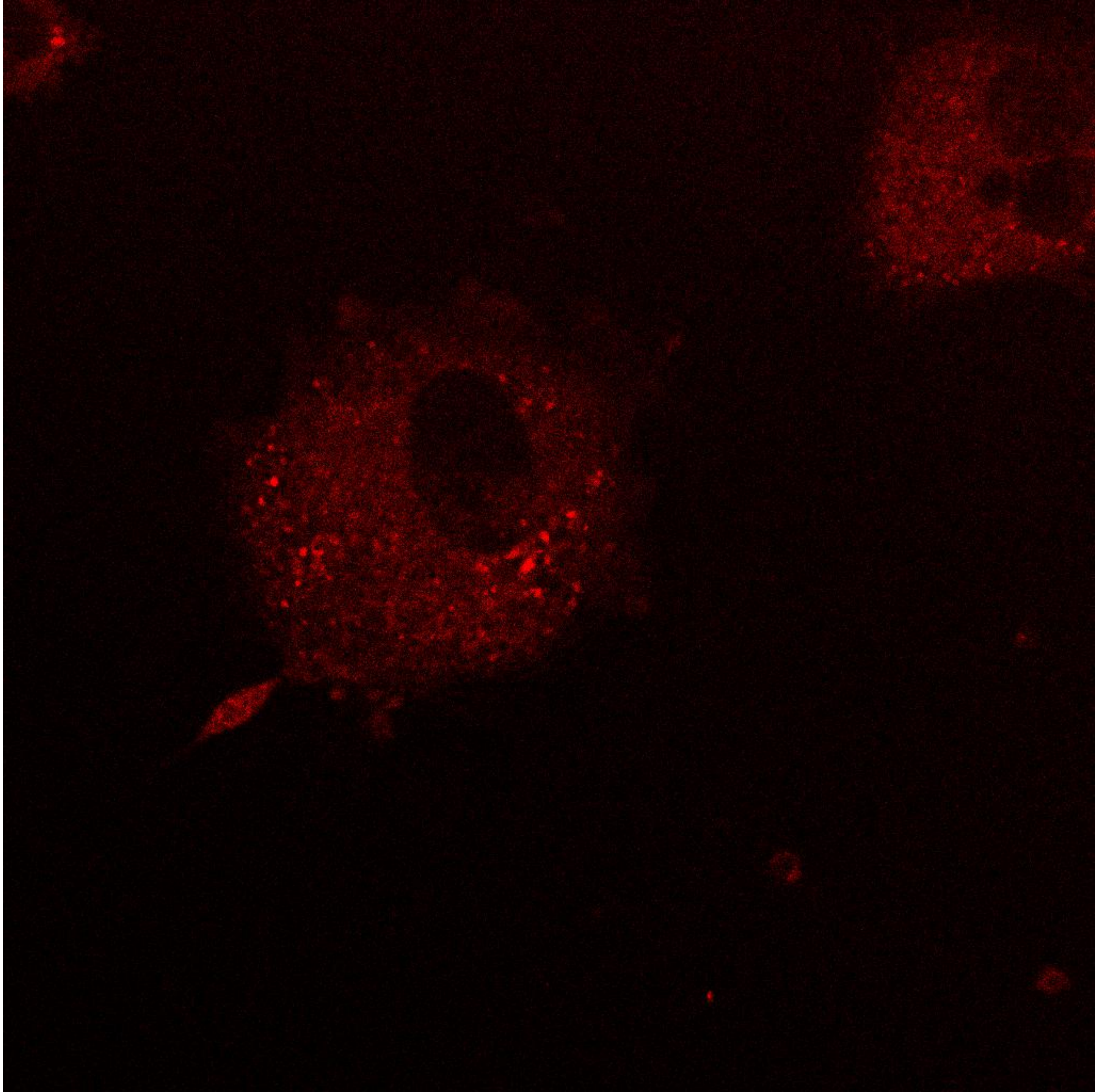

Figure 4A
CTRL

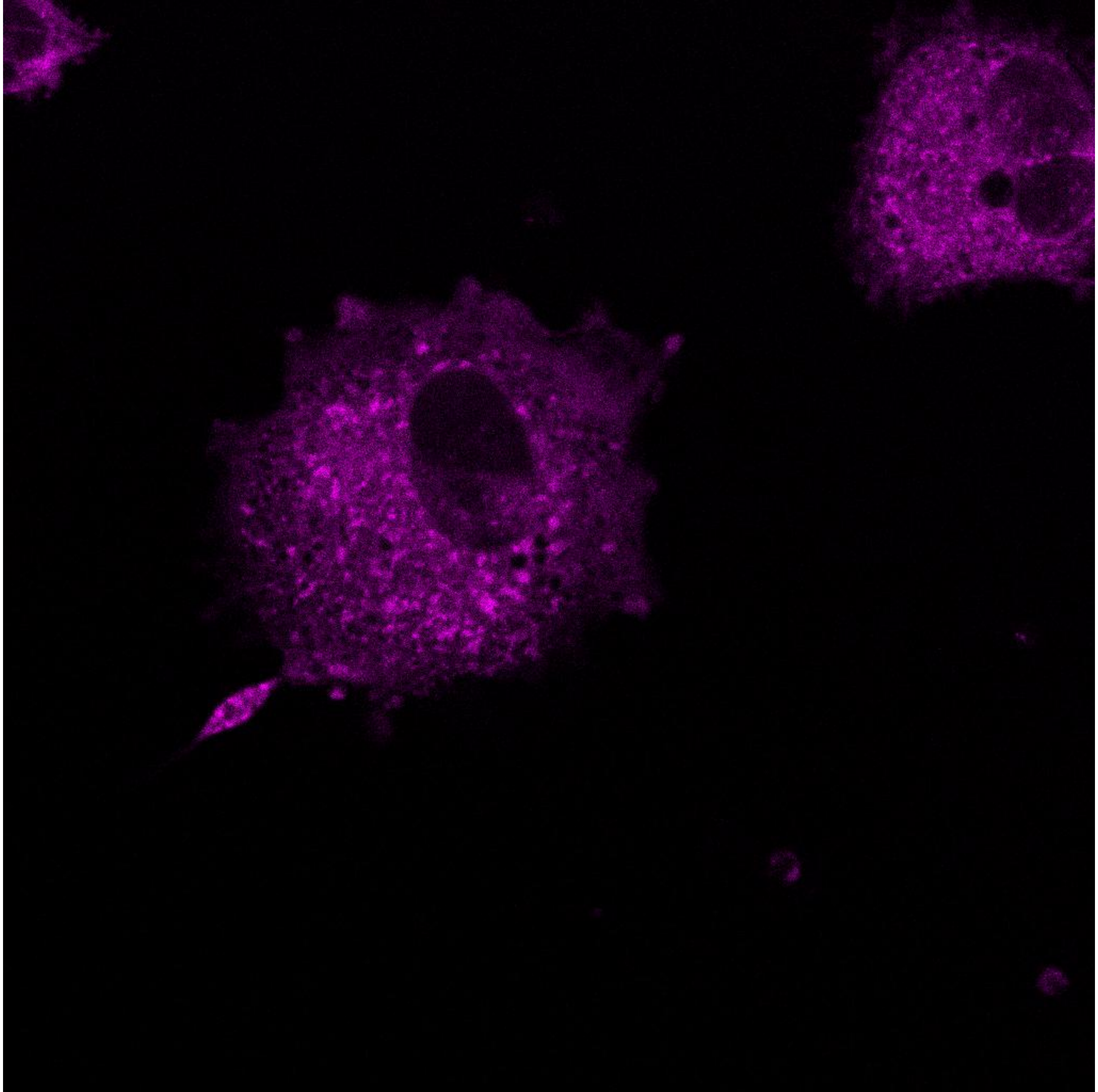

Figure 4A
CTRL

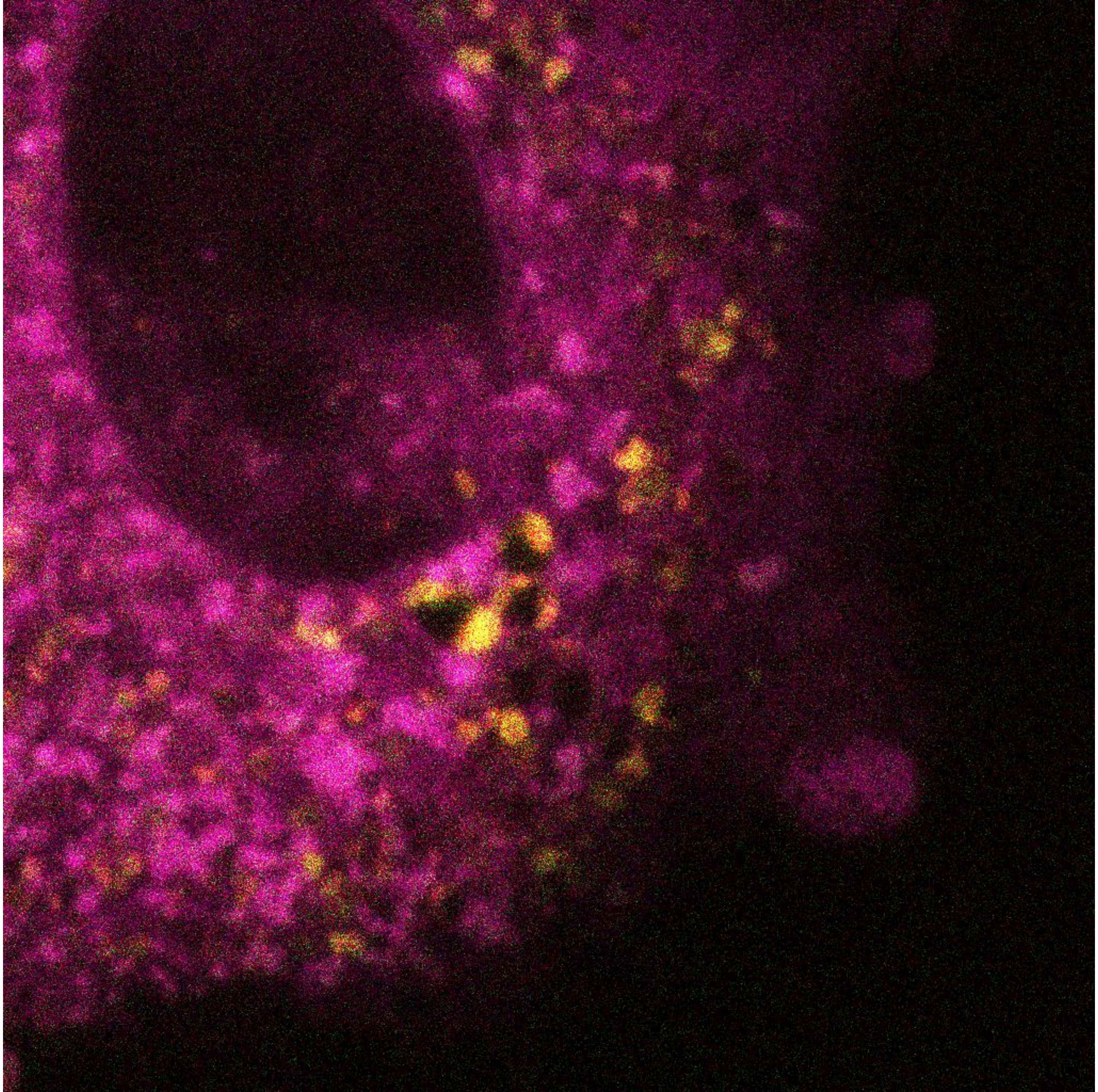

Figure 4A
CTRL

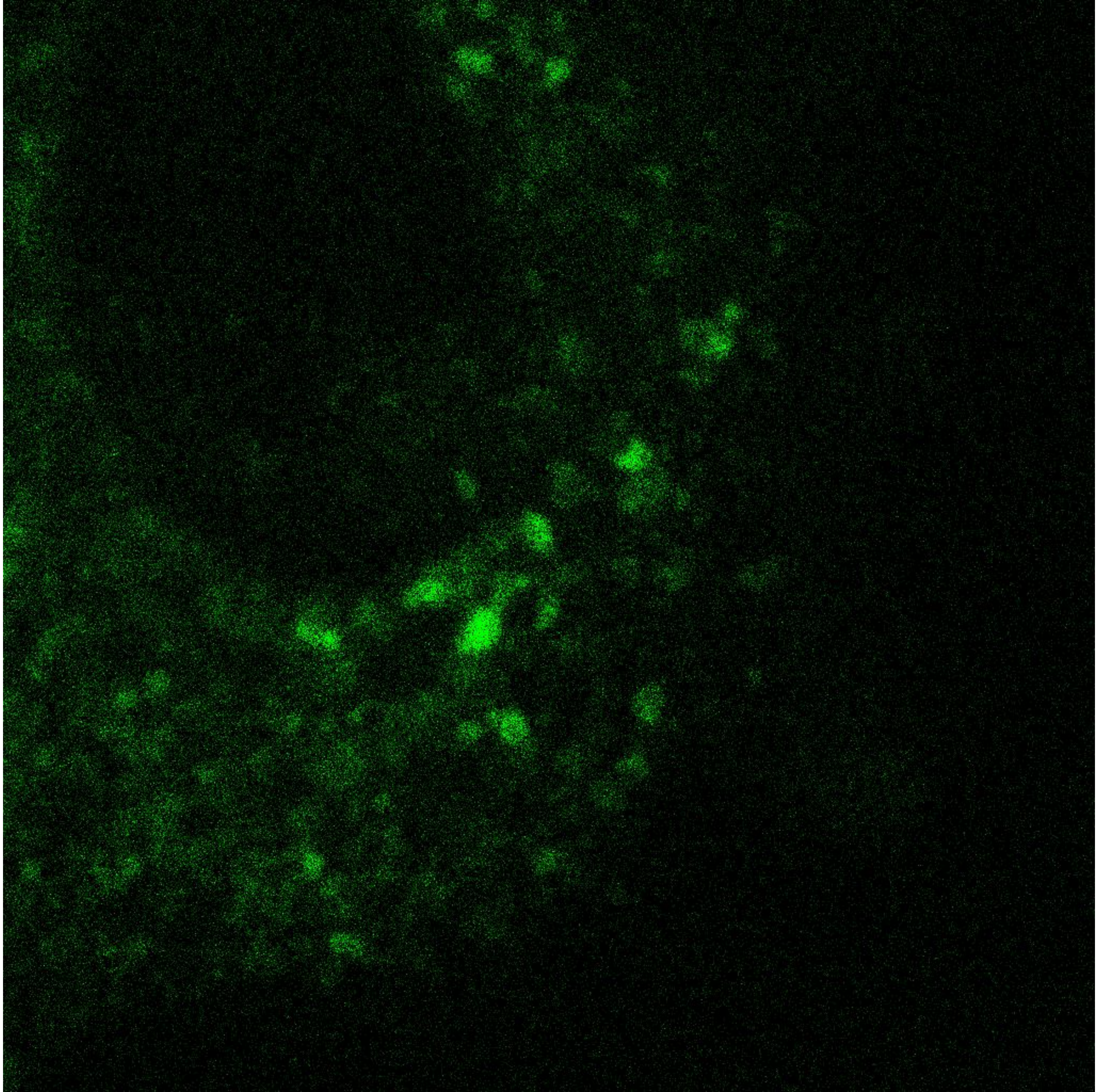

Figure 4A
CTRL

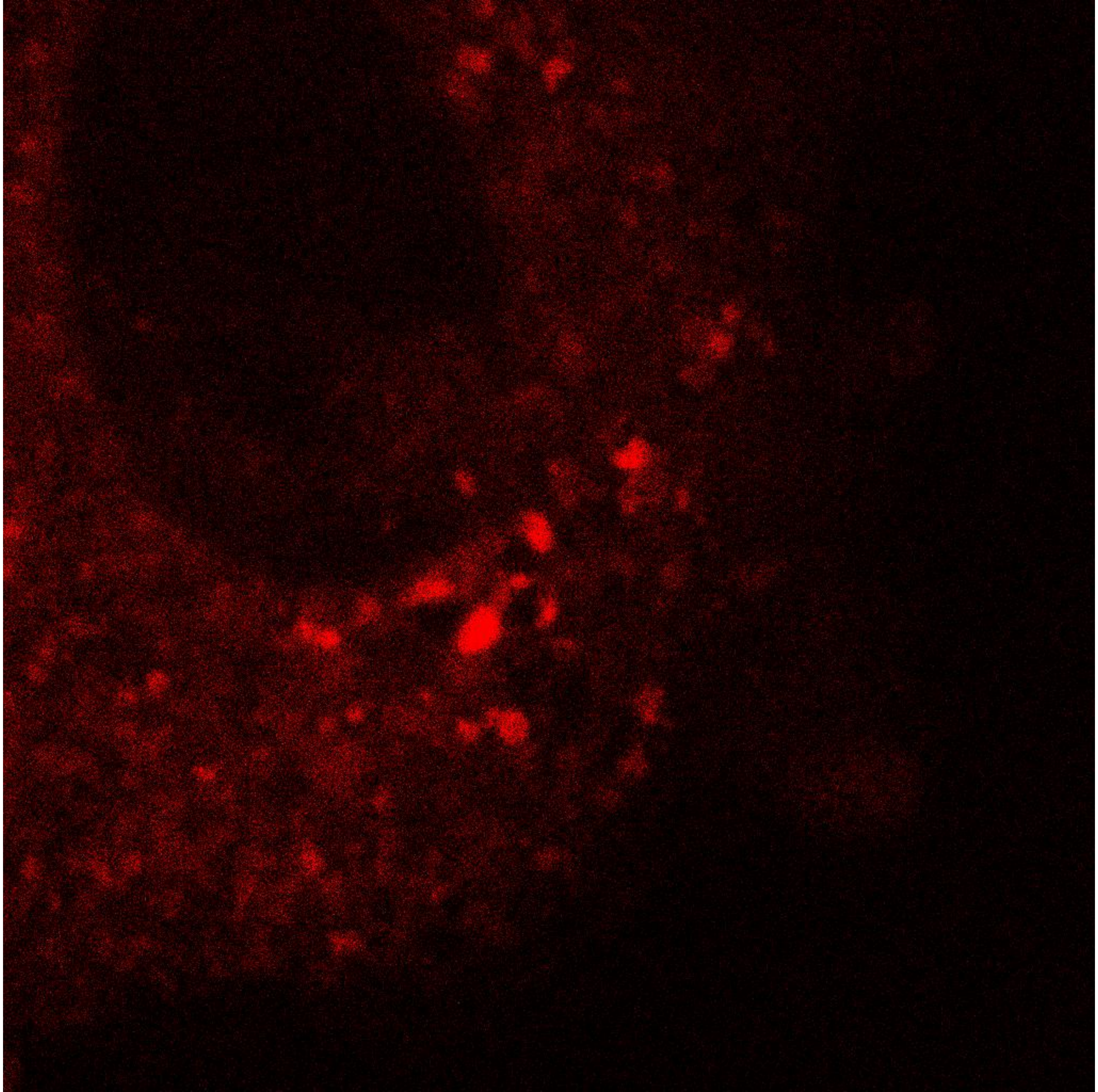

Figure 4A
WS5A

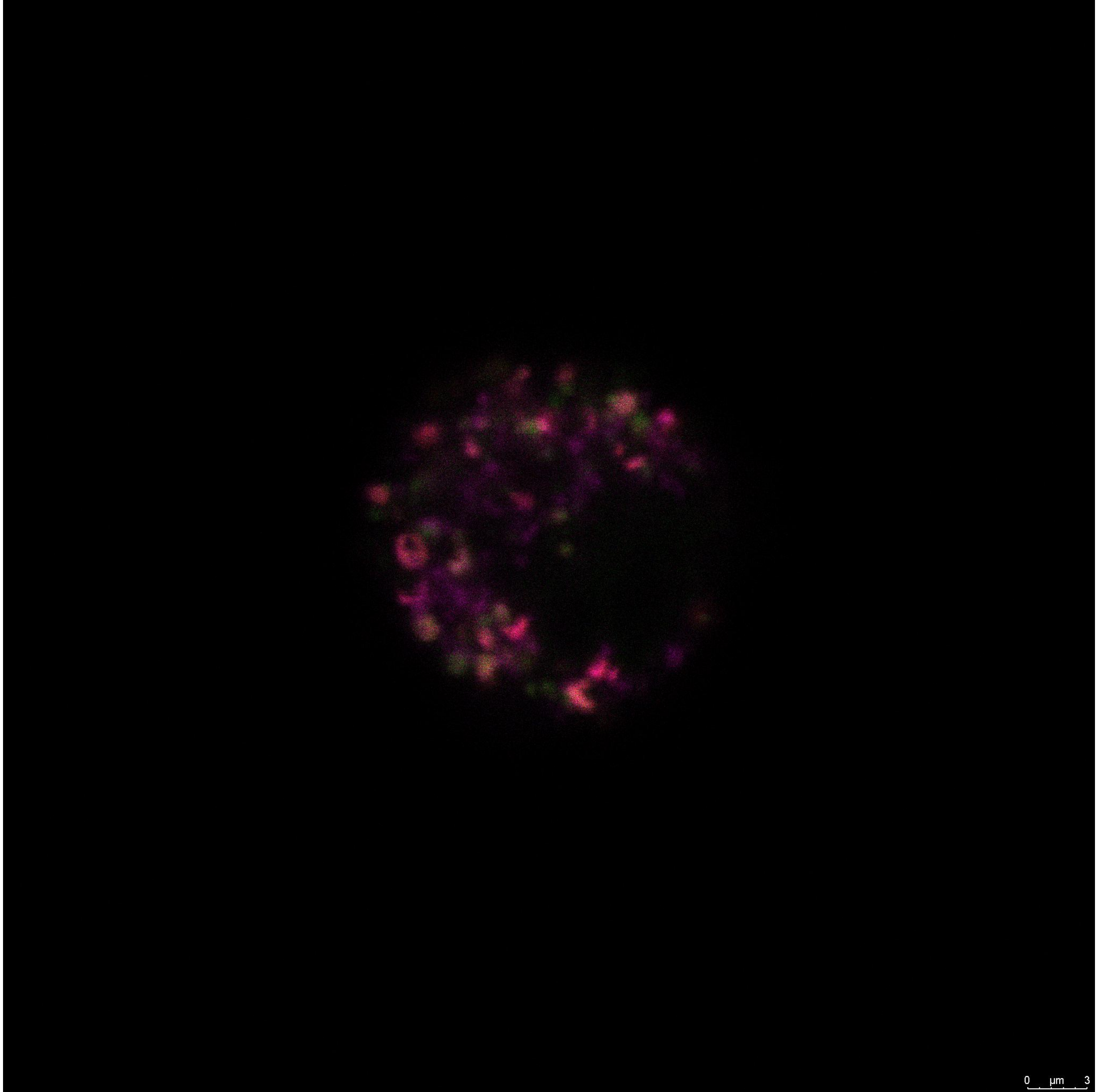

Figure 4A
WS5A

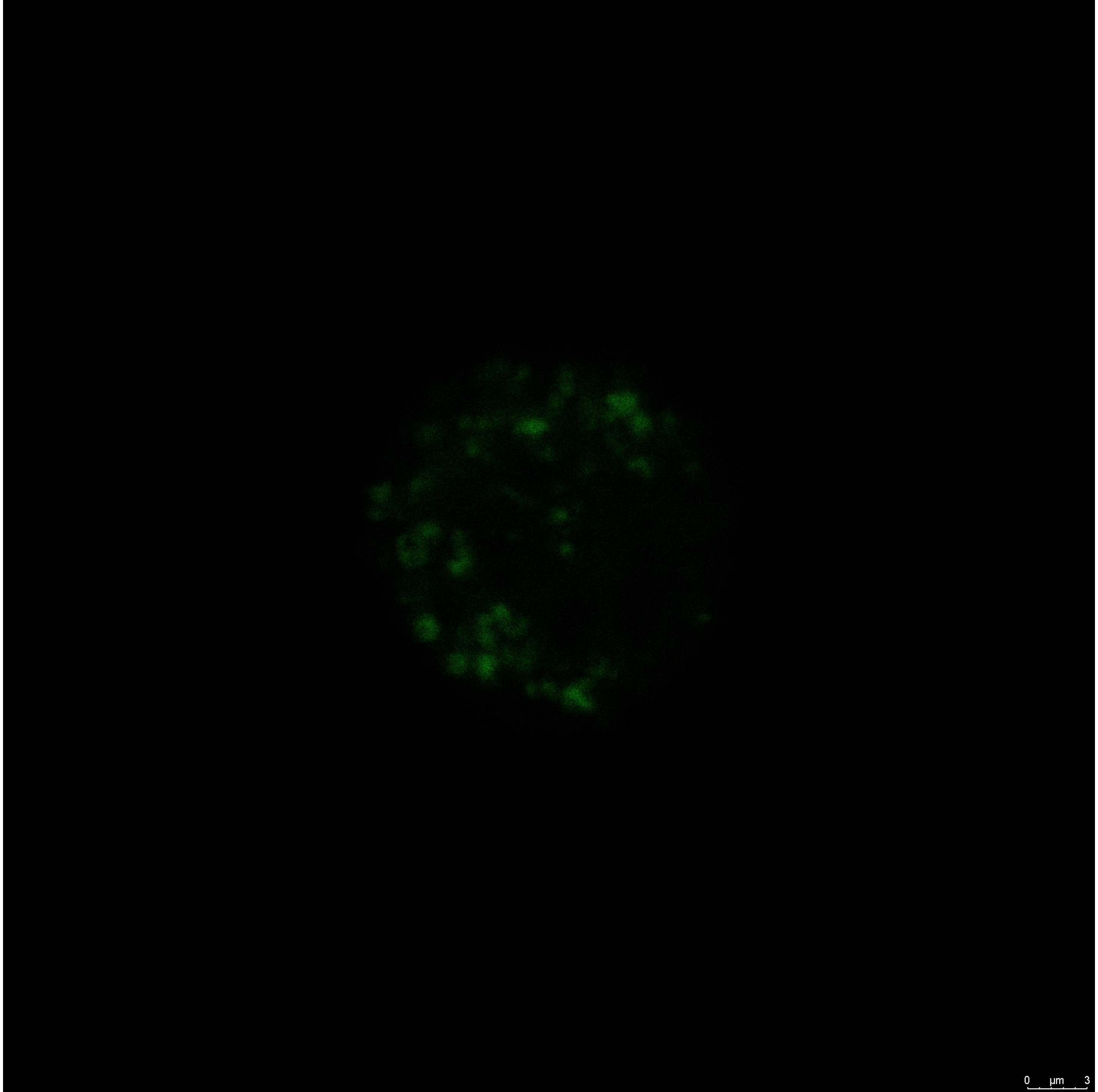

Figure 4A
WS5A

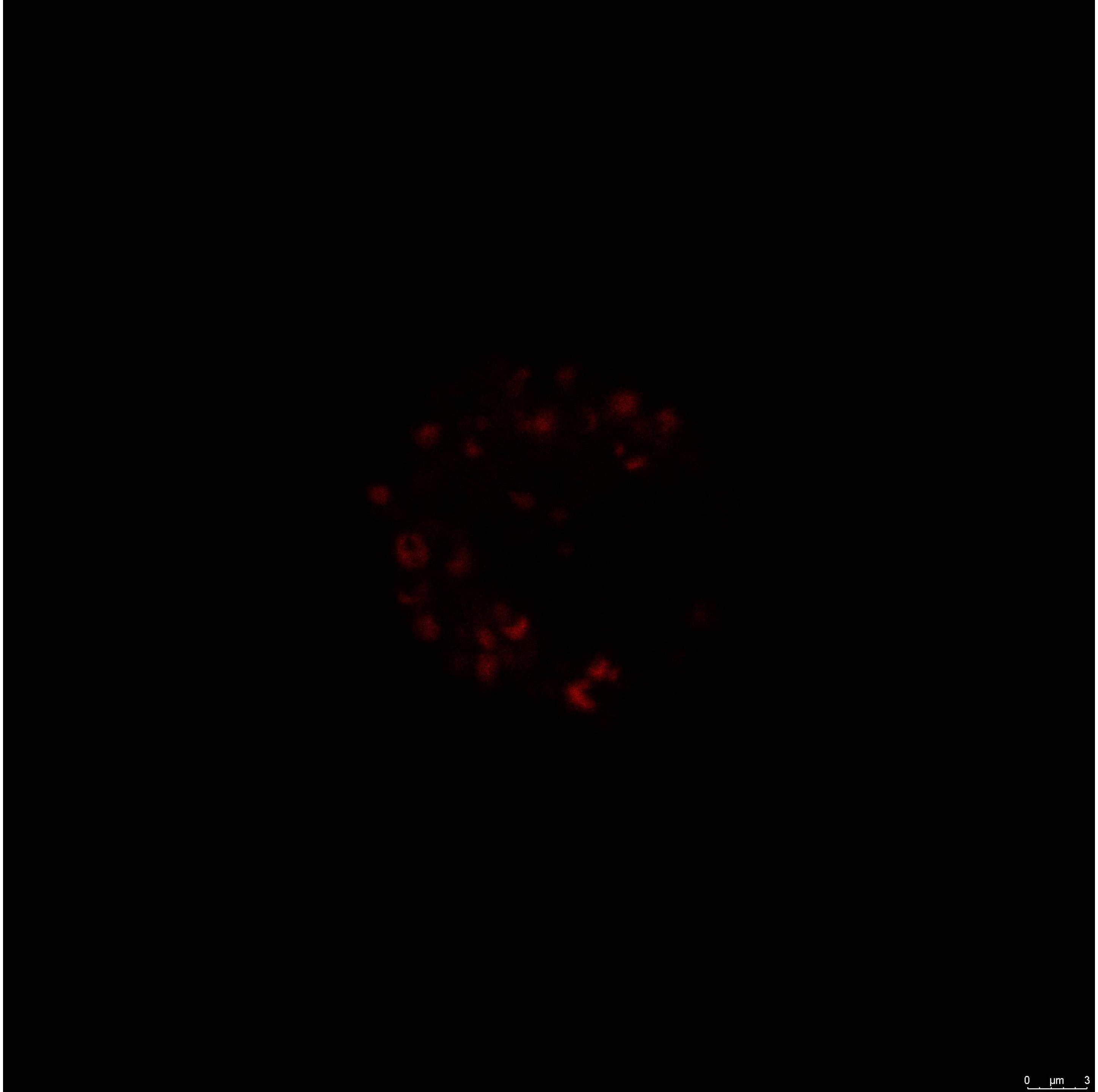

Figure 4A
CP2A

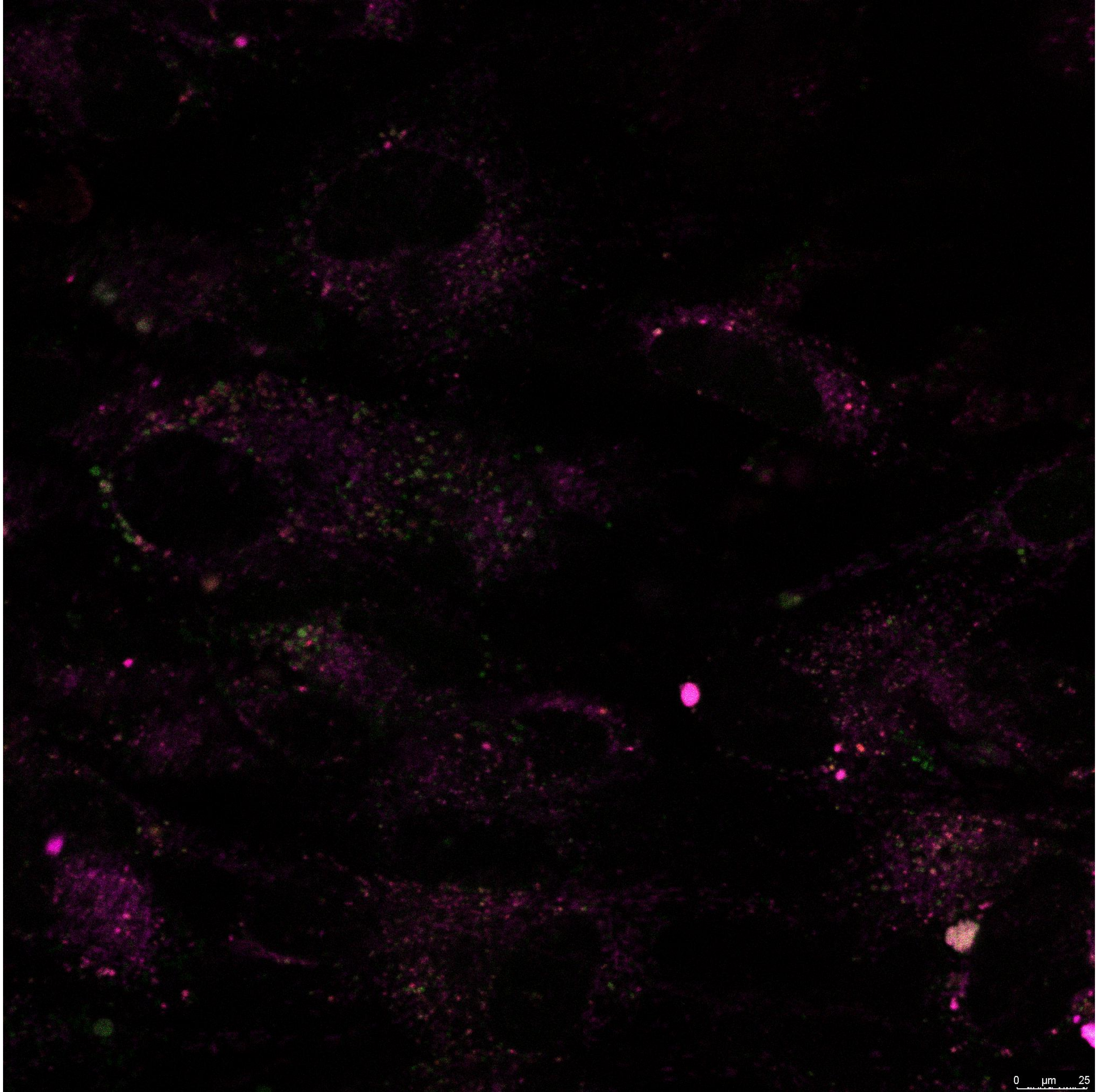

Figure 4A
CP2A

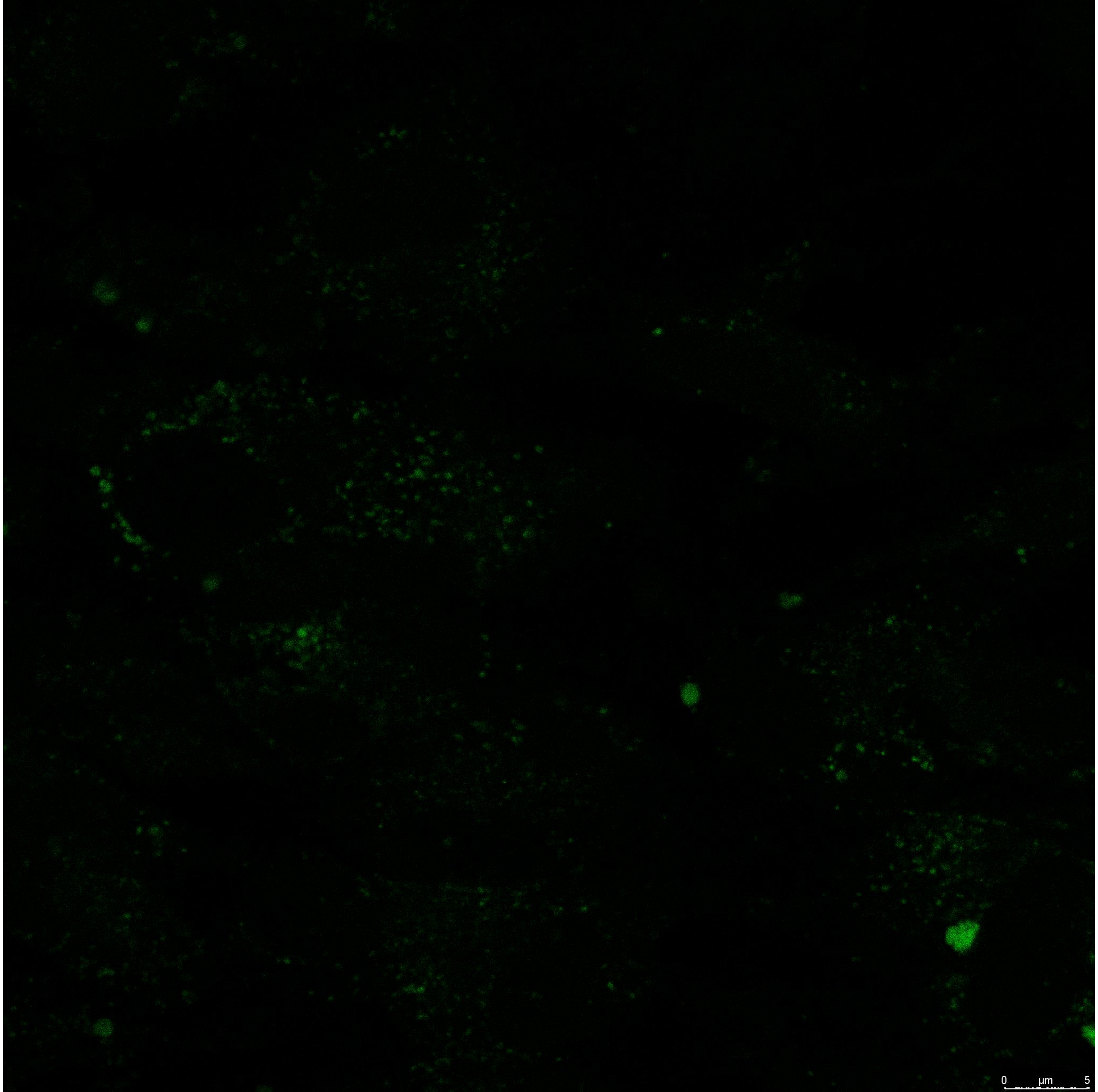

Figure 4A
CP2A

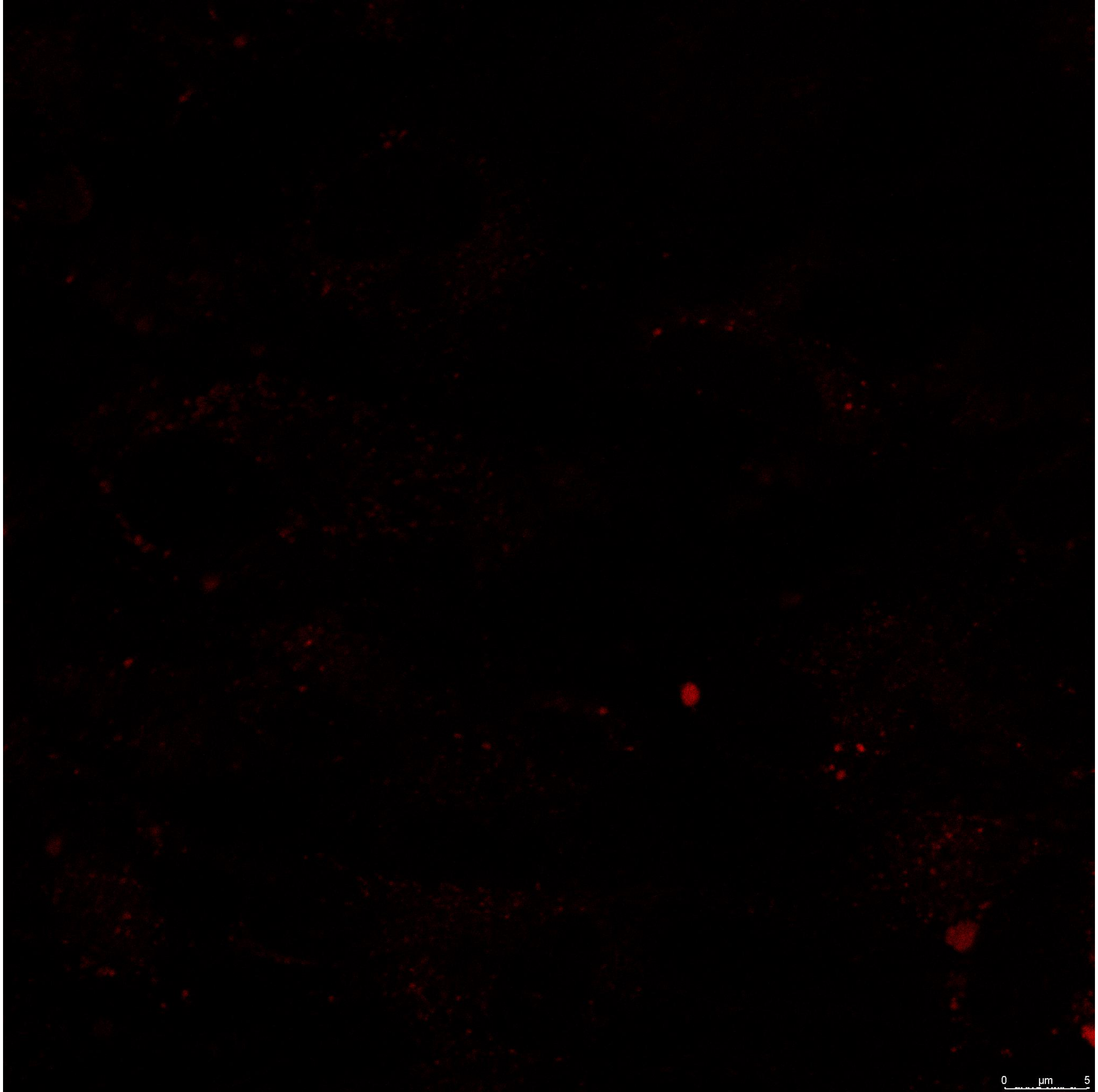

Figure 4A
CP2A

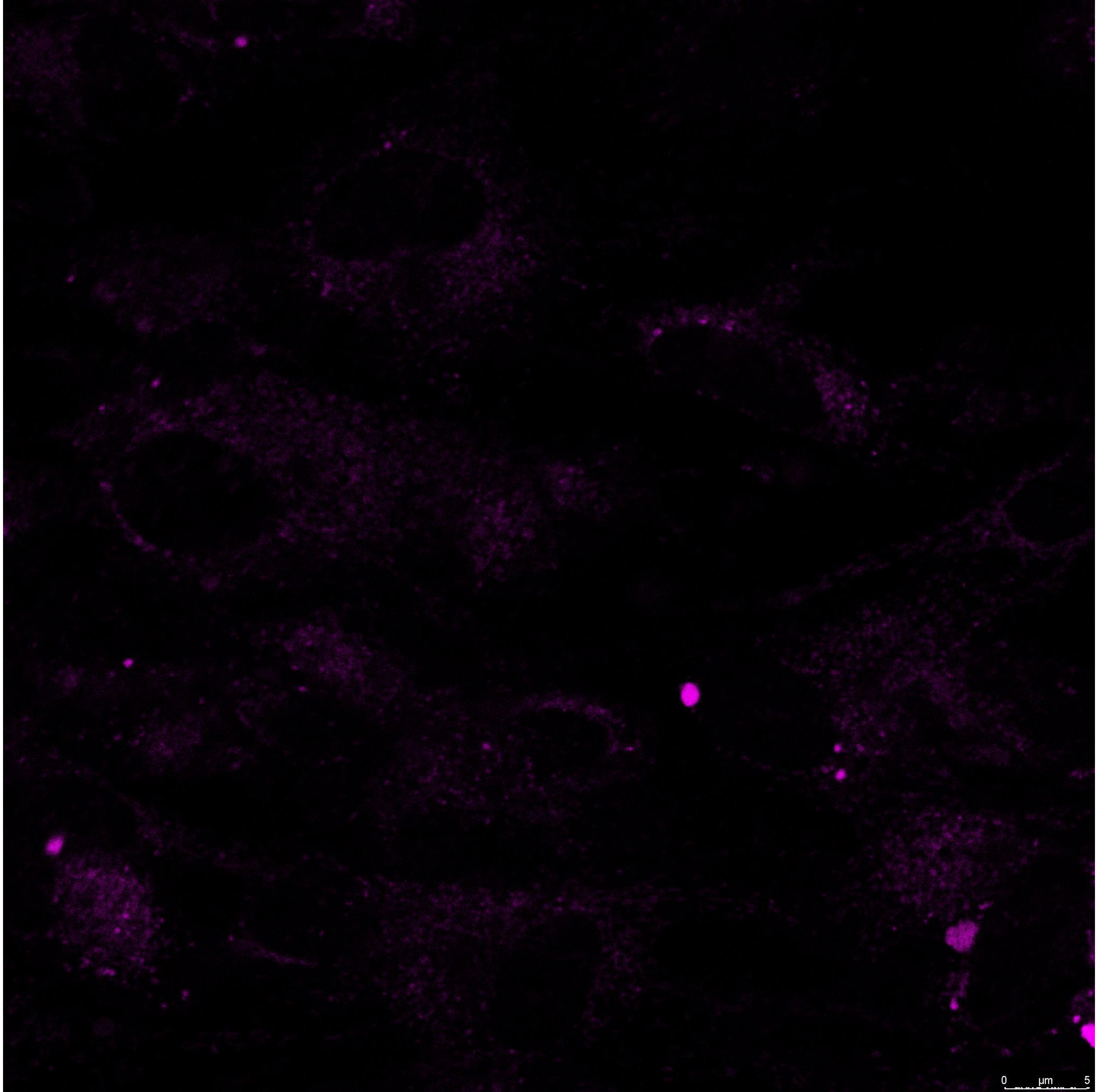

Figure 4C

LAMP2A

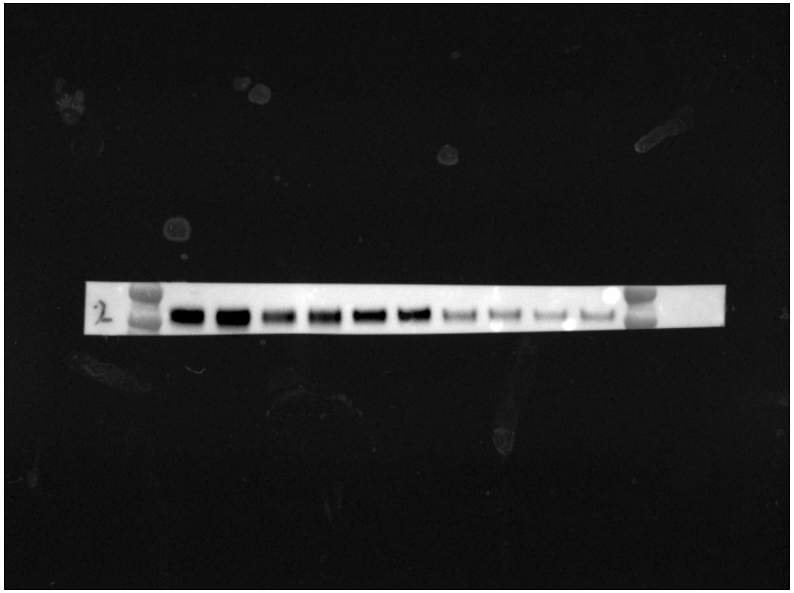

Figure 4C

LC3B

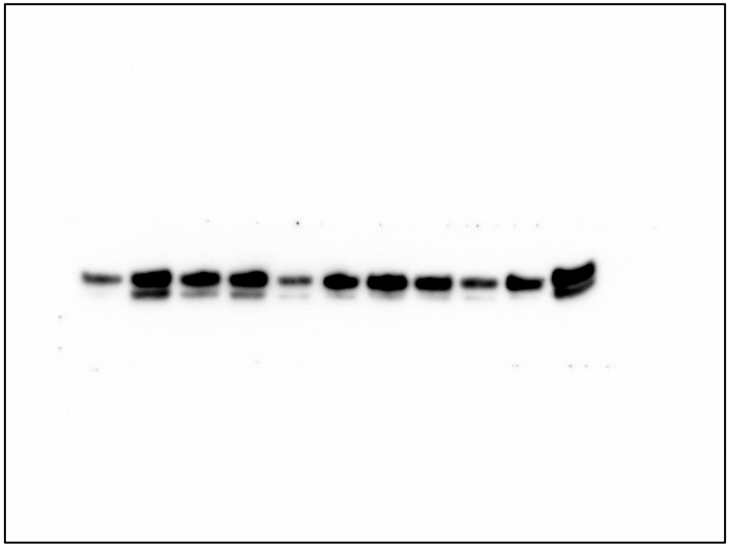

Figure 4C

p62

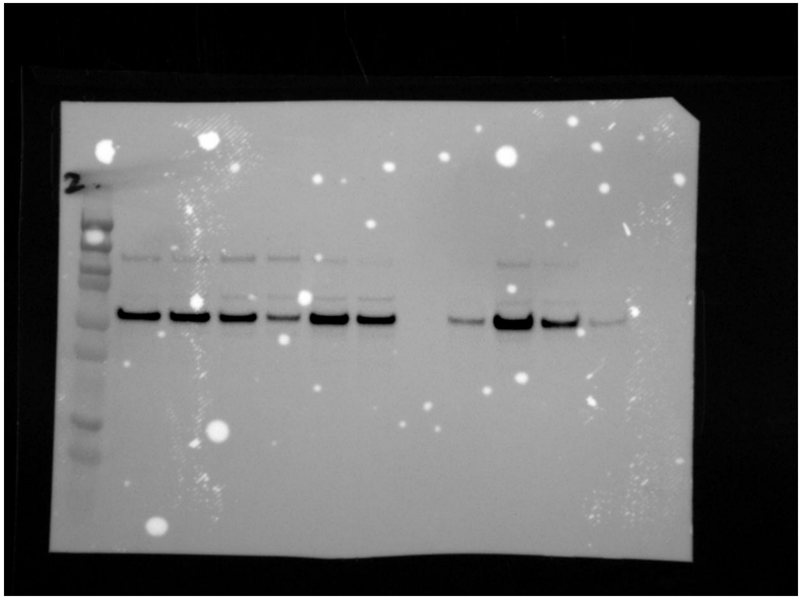

Figure 4C

GAPDH

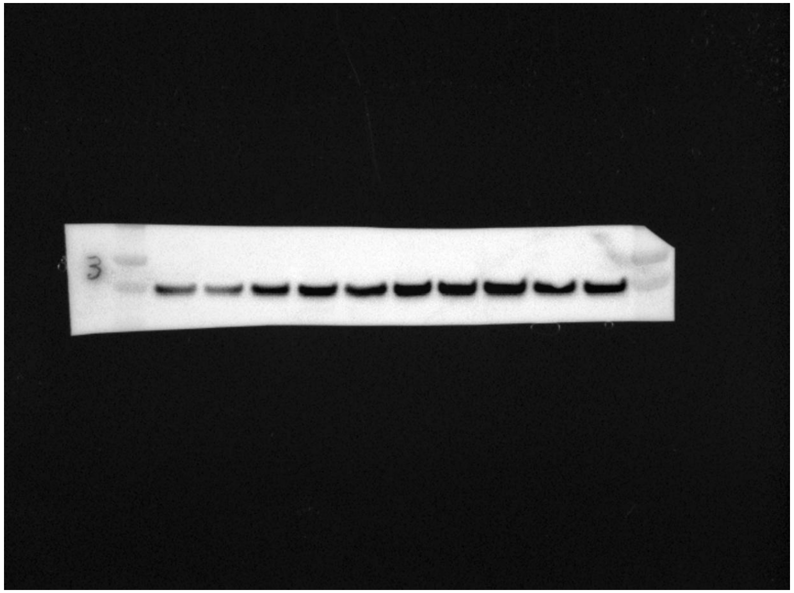

Figure 4D

PINK1

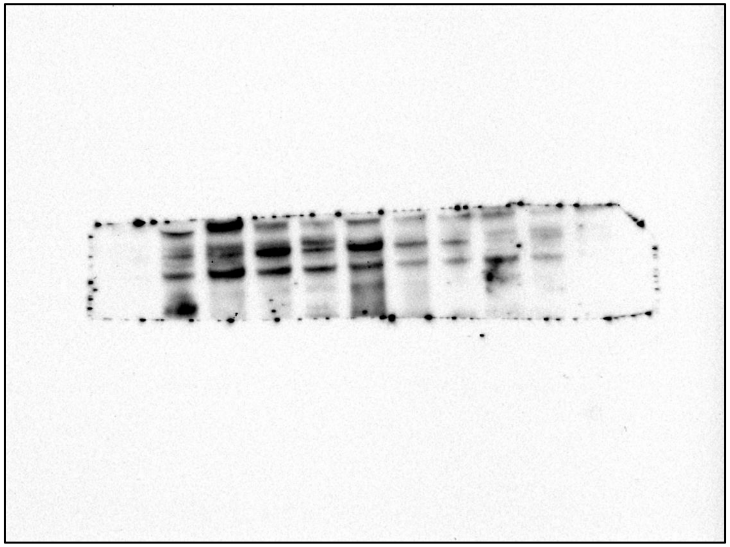

Figure 4D

PARKIN

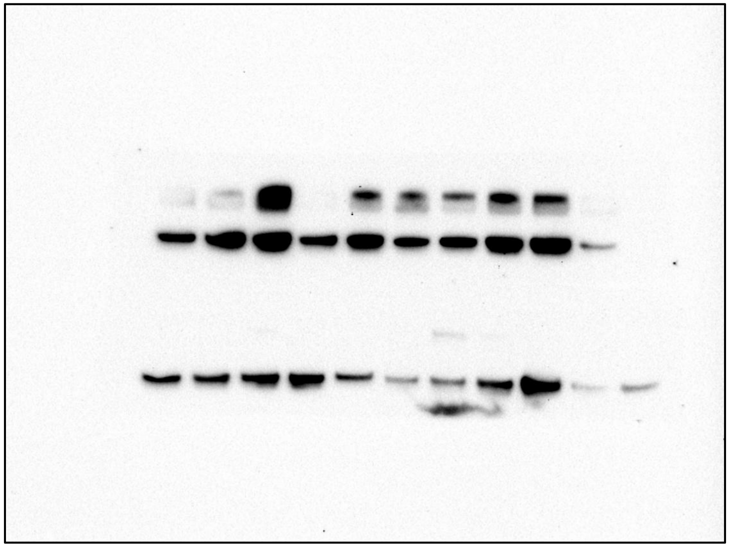

Figure 4D

GAPDH

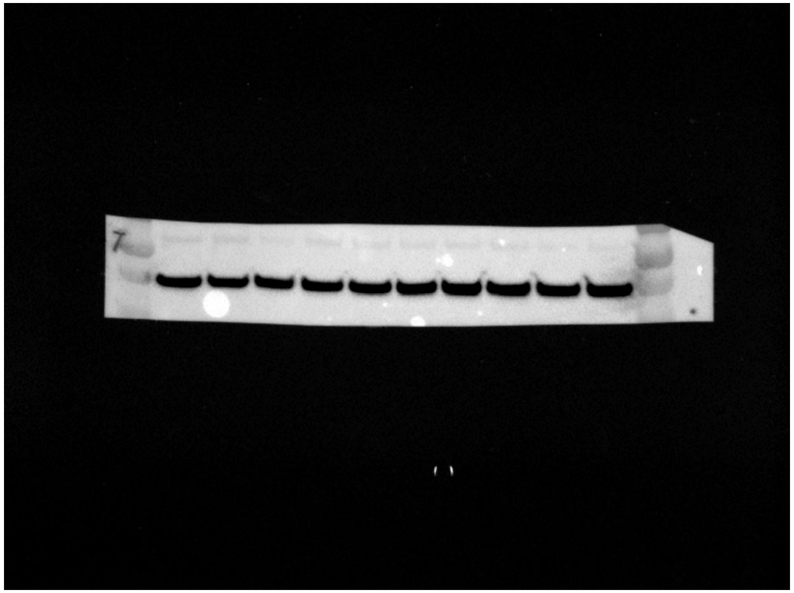

Figure 5A

p-mTOR

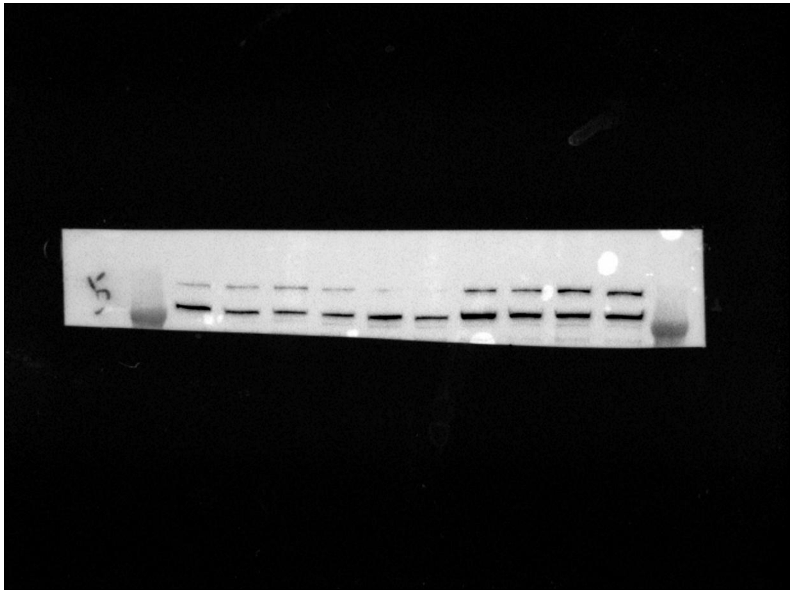

Figure 5A

mTOR

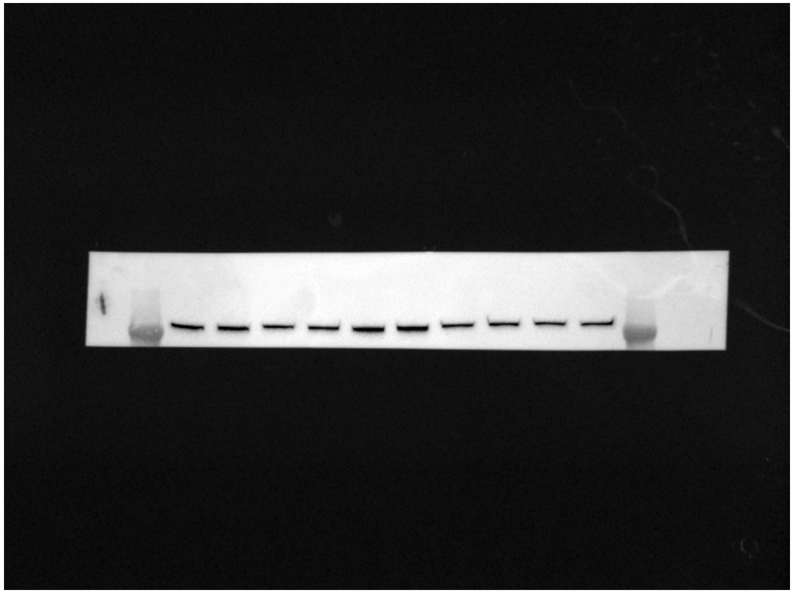

Figure 5A

GAPDH

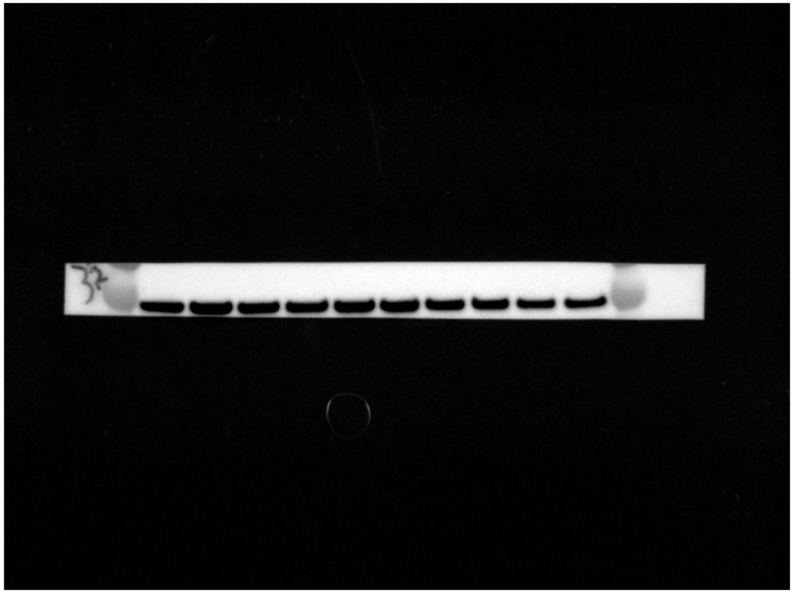

Figure 5B

p-ULK1

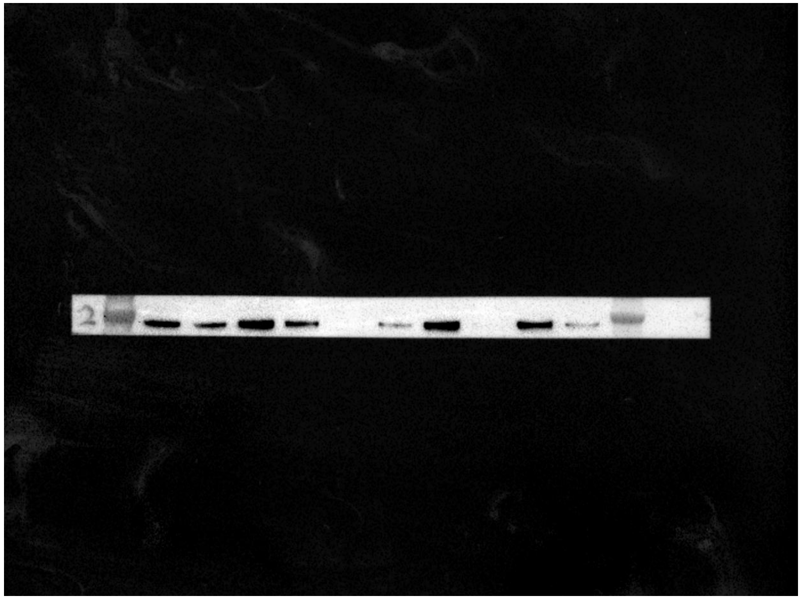

Figure 5B

ULK1

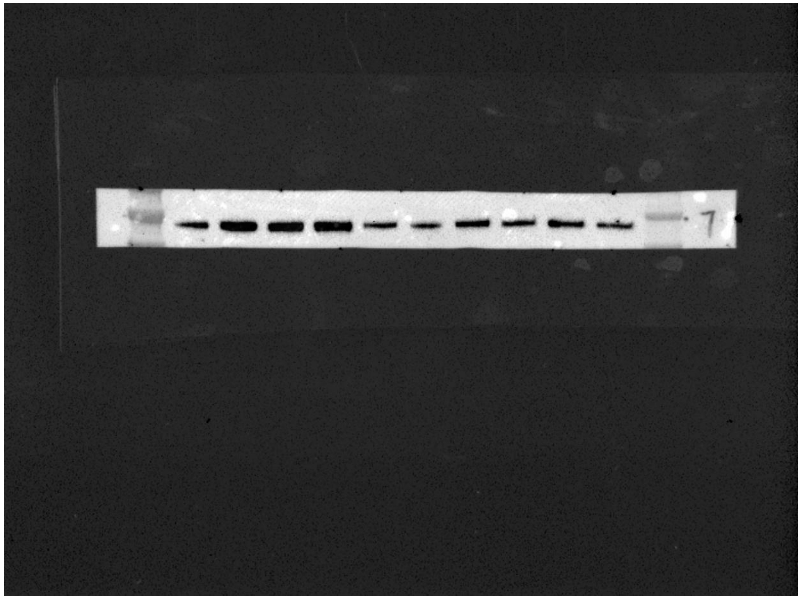

Figure 5B

GAPDH

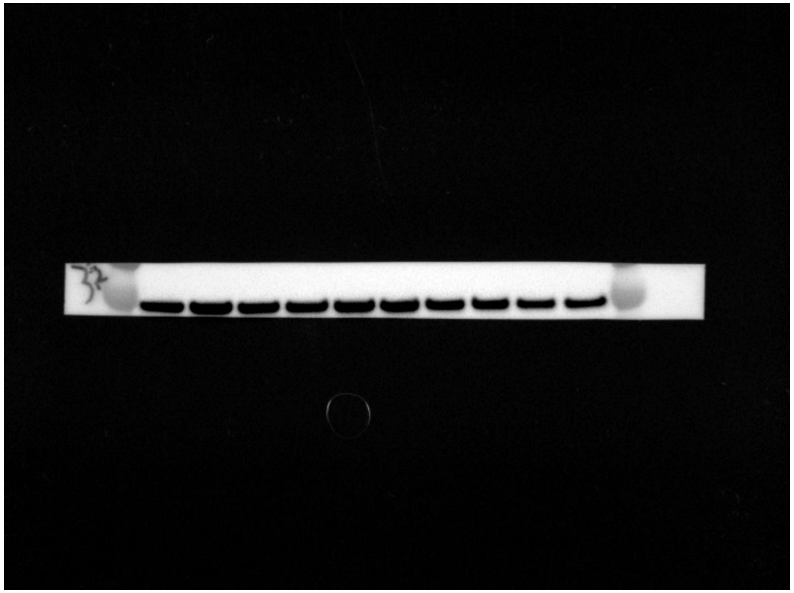

Figure 5C

p-AKT

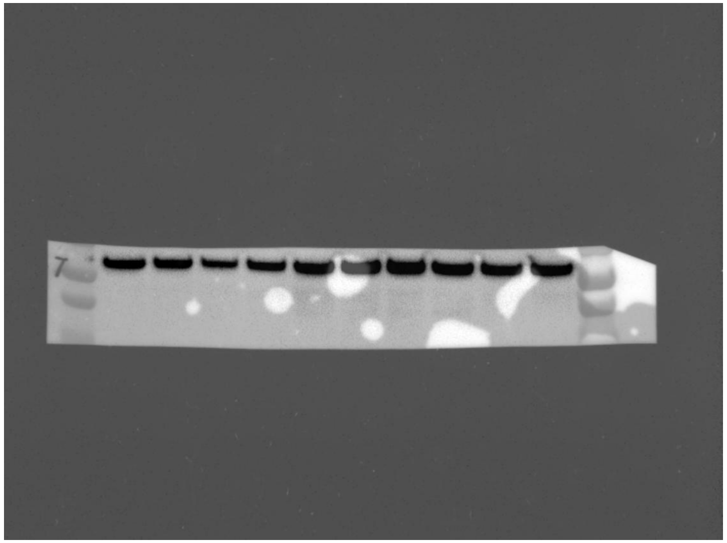

Figure 5C

AKT

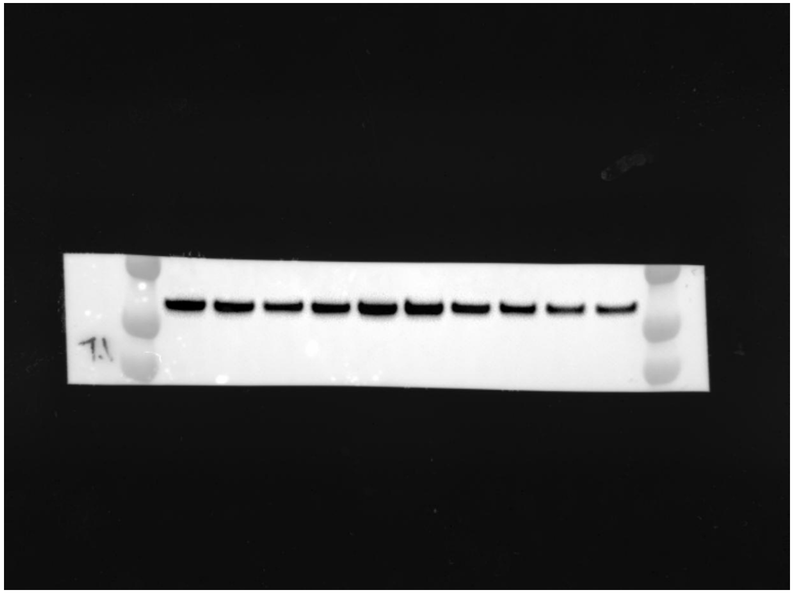

Figure 5C

GAPDH

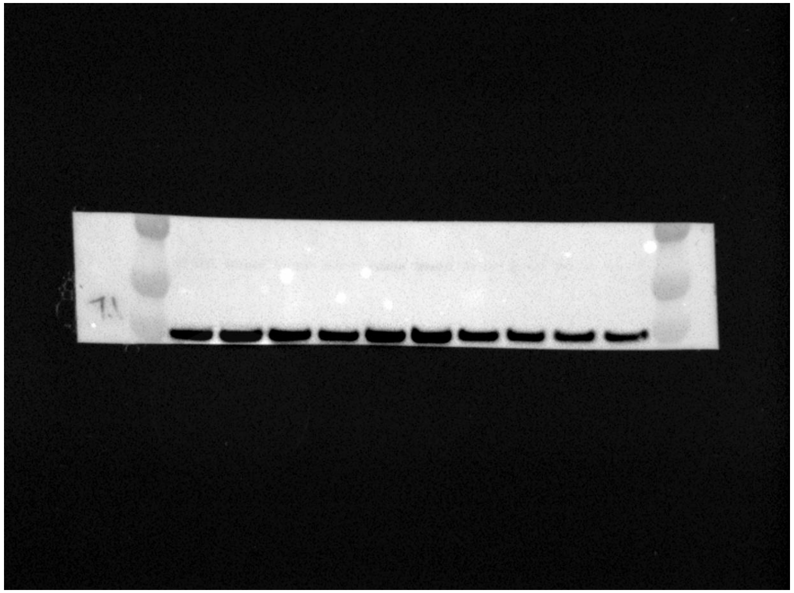

Figure 5D

p-AMPK (T183+T172)

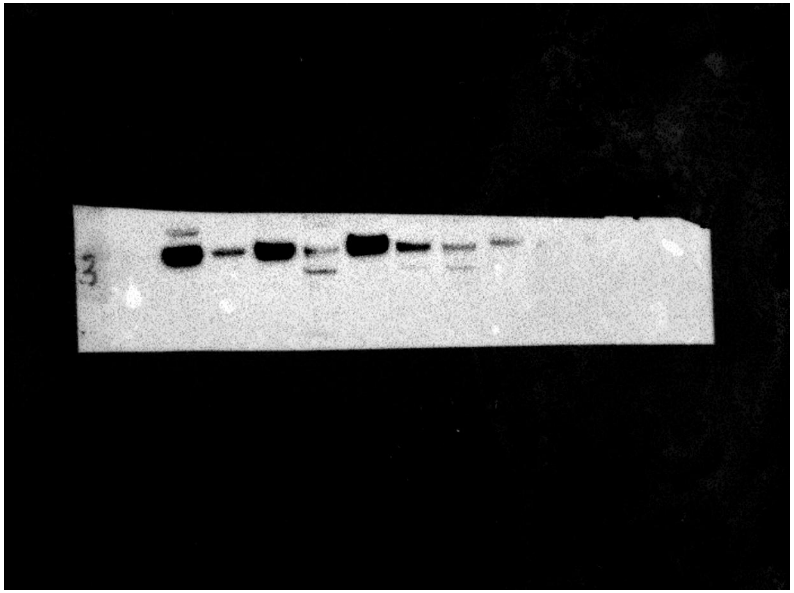

Figure 5D

AMPK

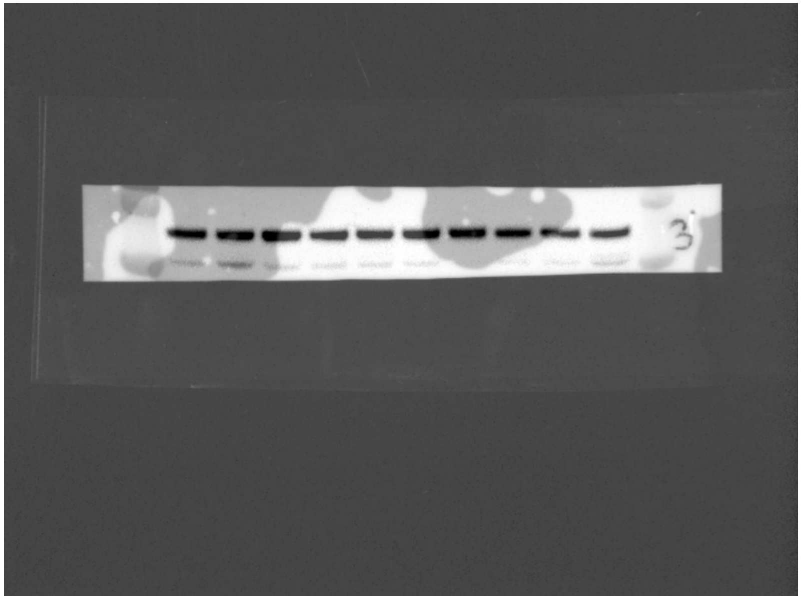

Figure 5D

GAPDH

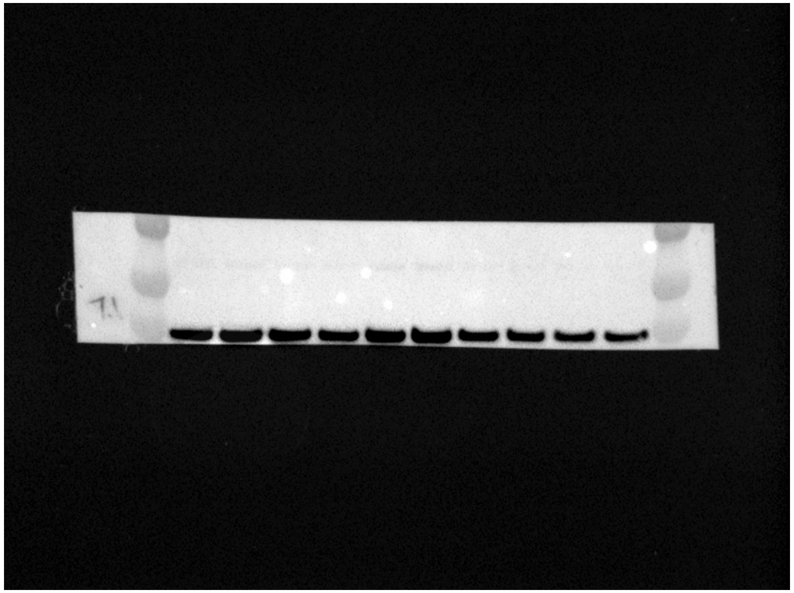

Figure 6D

p-SIRT1

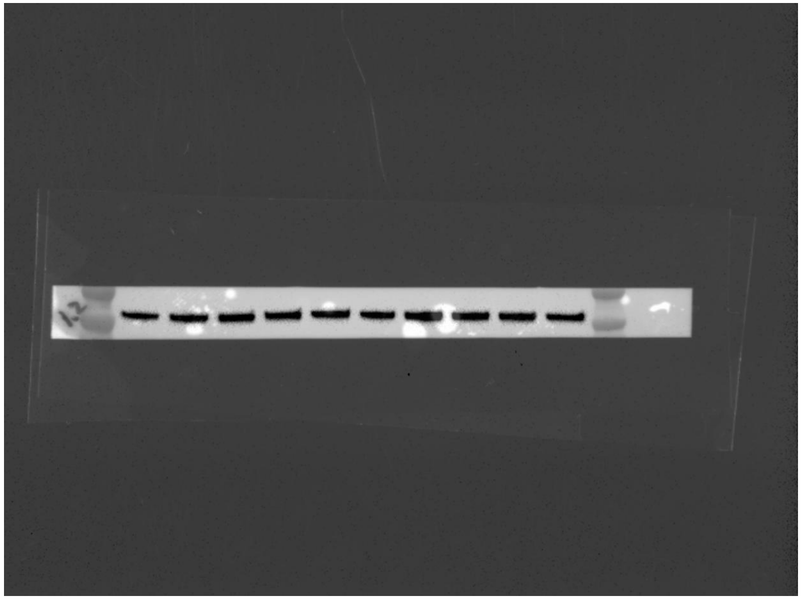

SIRT1

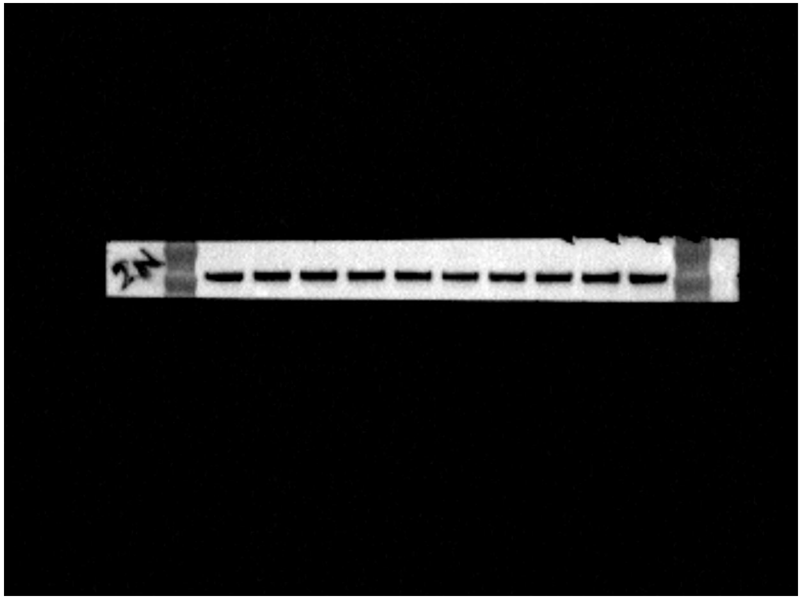

Figure 6D

p-AMPK (T183+T172)

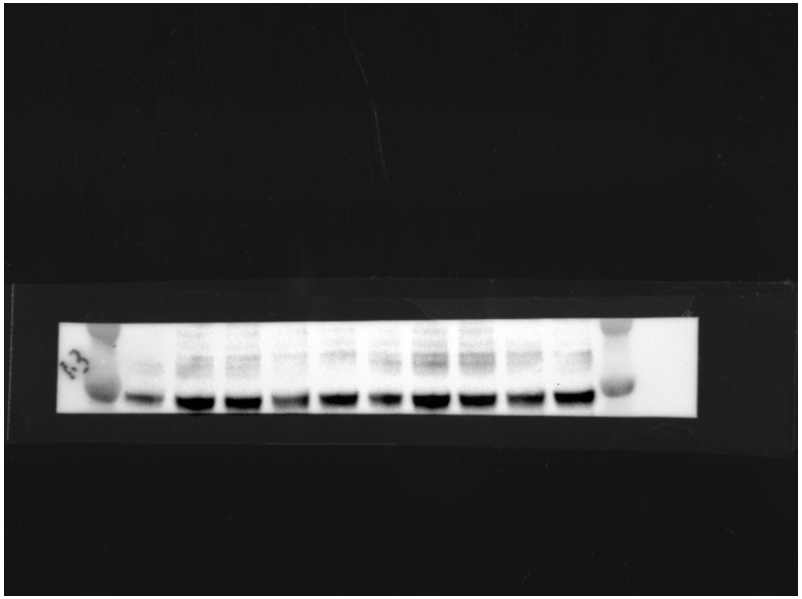

AMPK

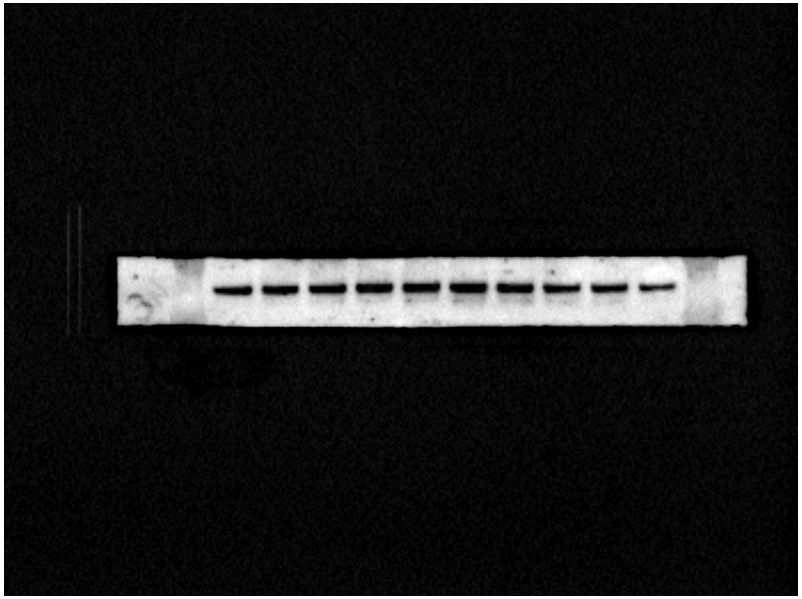

Figure 6D

p-mTOR

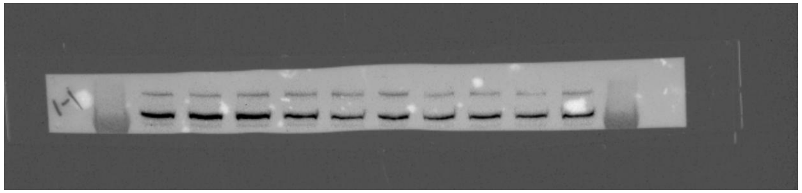

mTOR

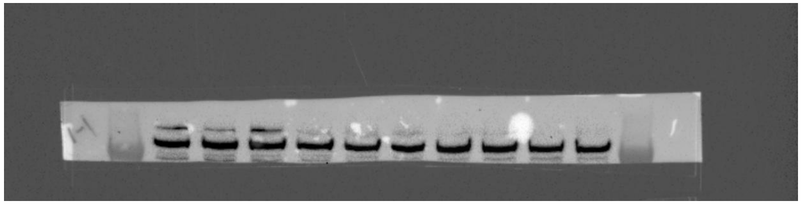

Figure 6D

GAPDH

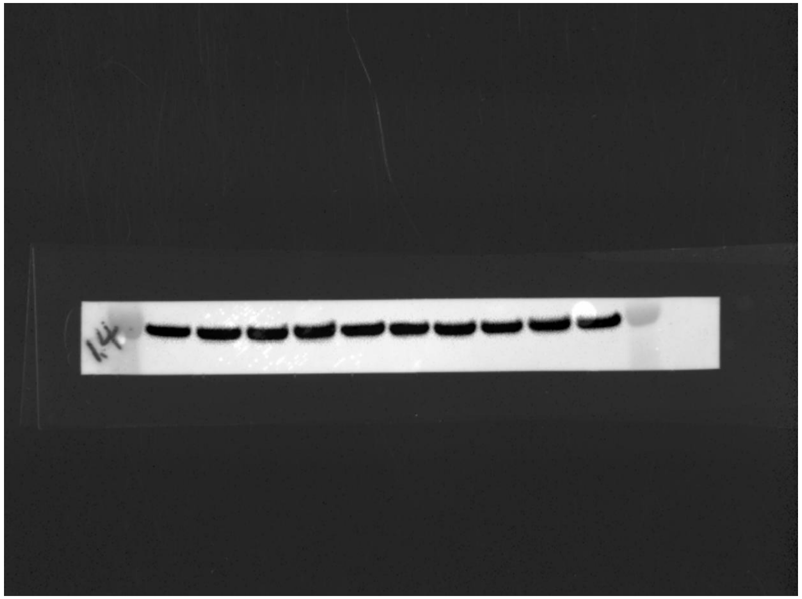

Figure 6E

LC3B

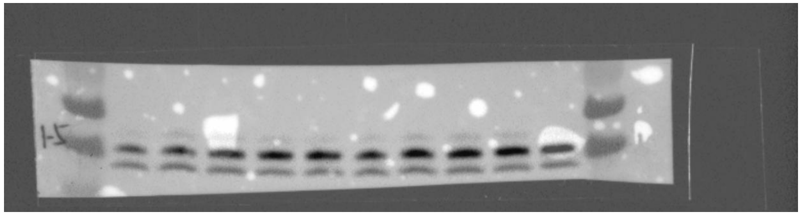

Figure 6E

p62

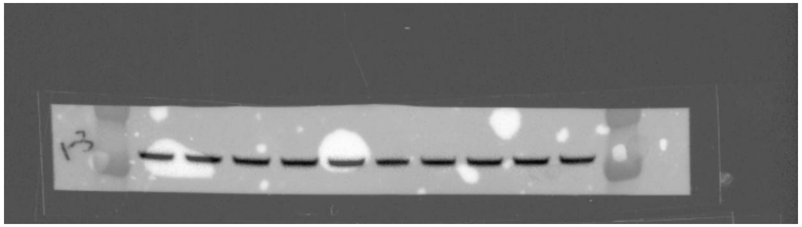

Figure 6E

GAPDH

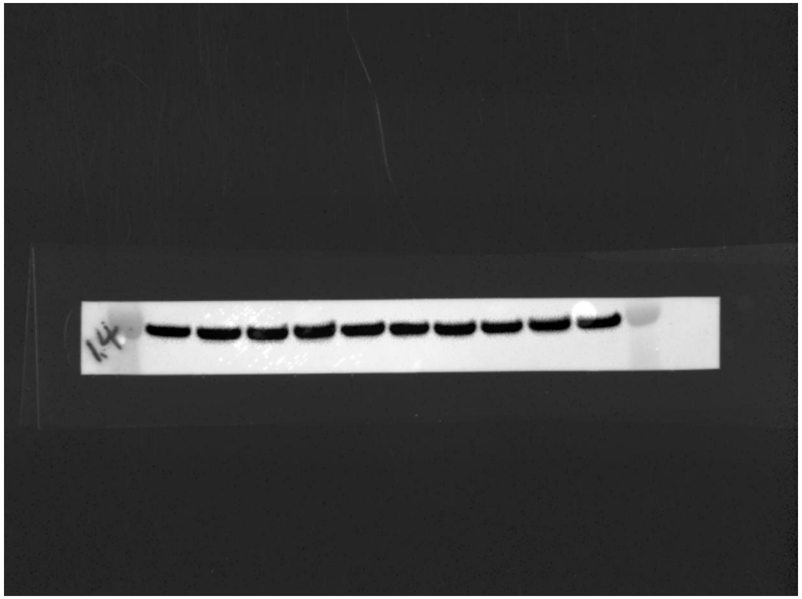

Figure 6F

p-SIRT1

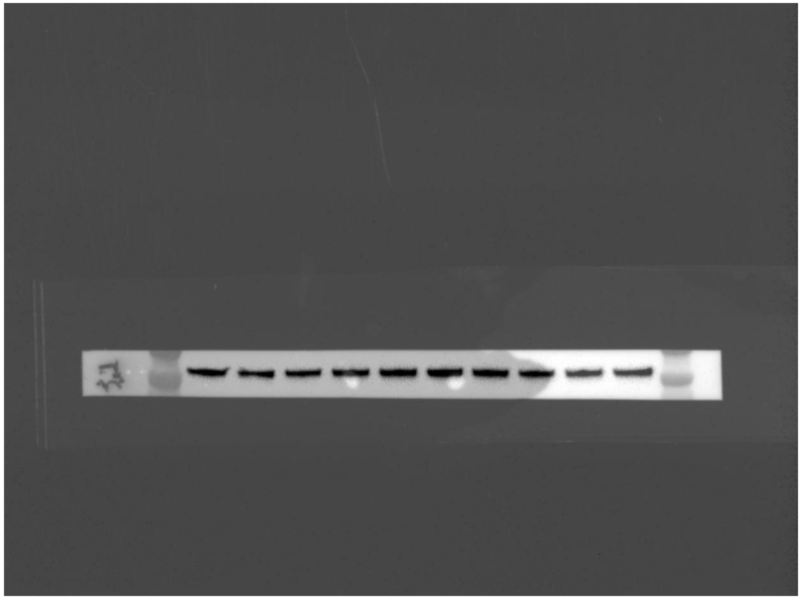

SIRT1

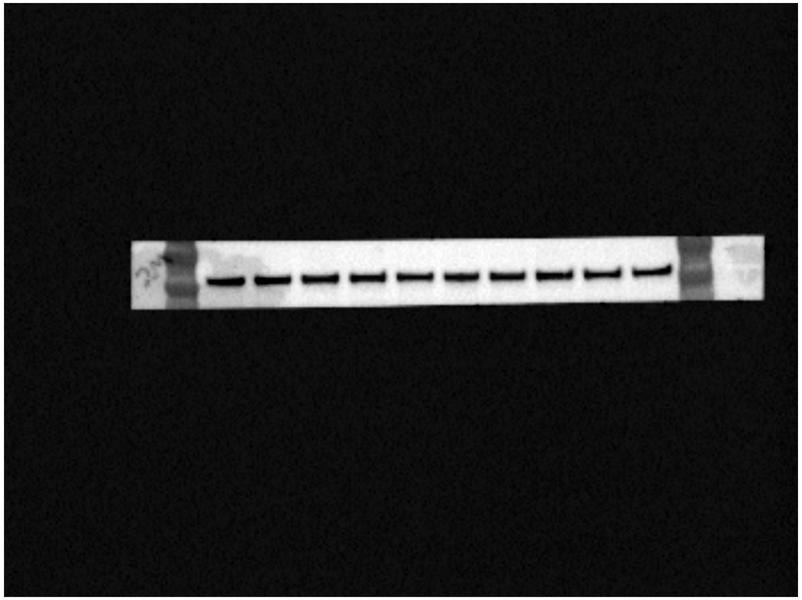

Figure 6F

p-AMPK (T183+T172)

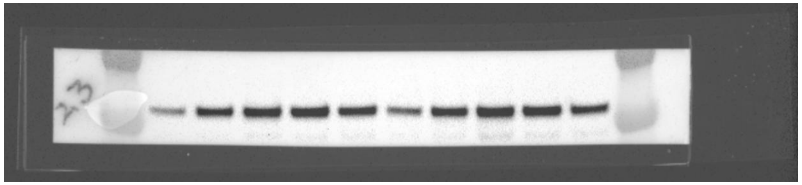

AMPK

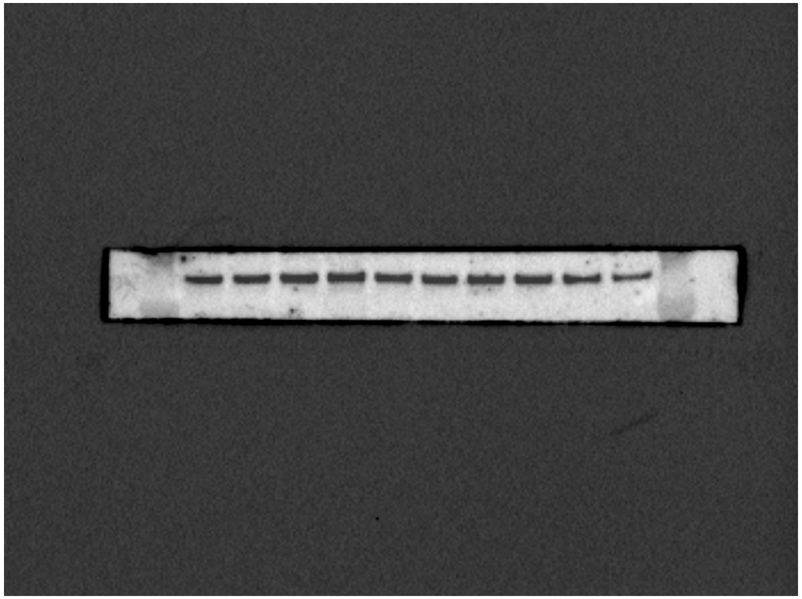

Figure 6F

p-mTOR

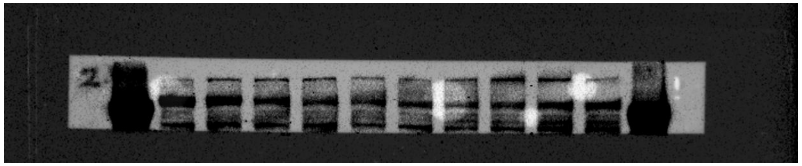

mTOR

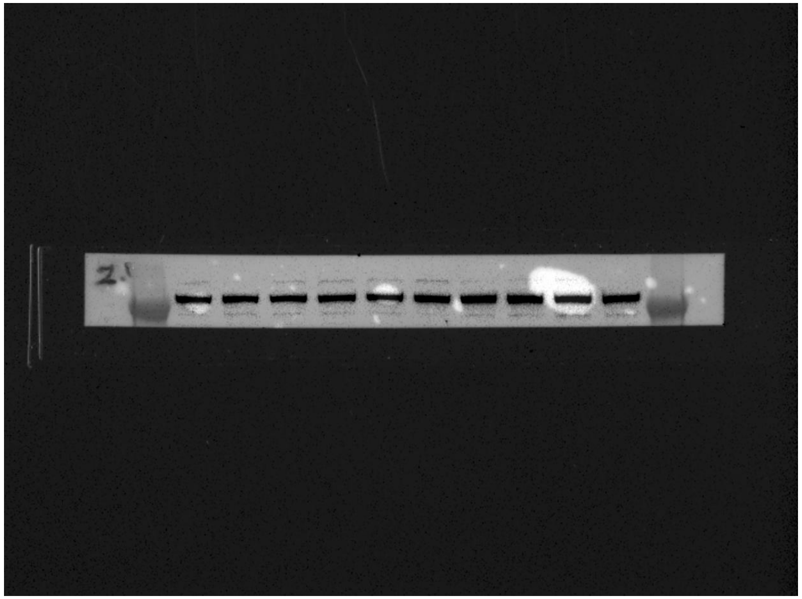

Figure 6F

GAPDH

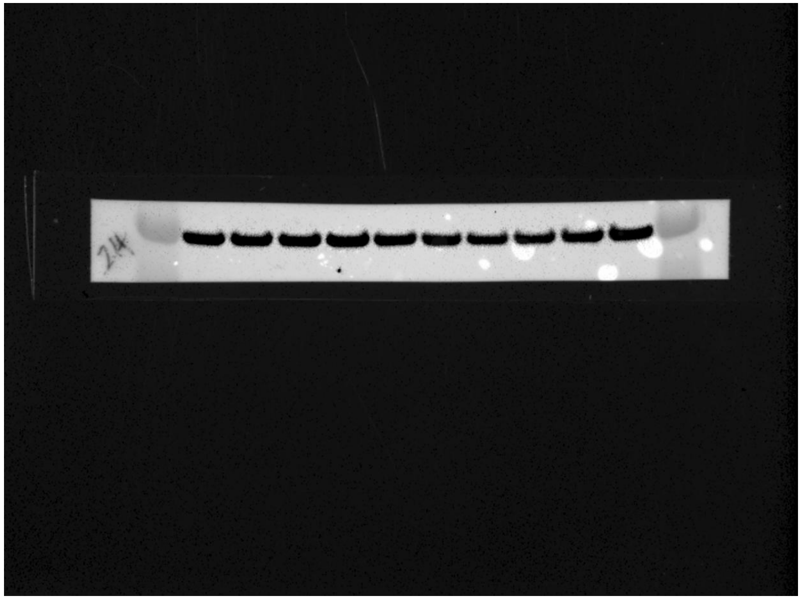

Figure 6G

LC3B

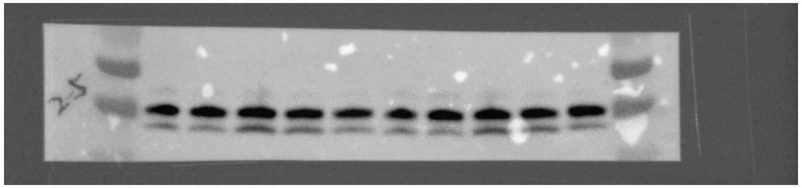

Figure 6G

p62

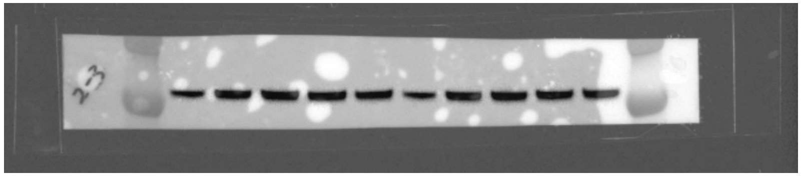

Figure 6G

GAPDH

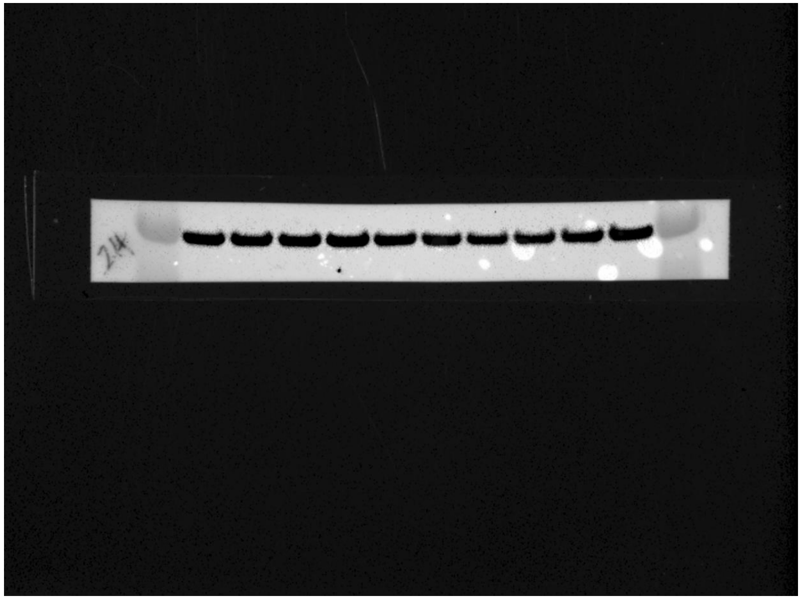

Figure 7A

p-SIRT1

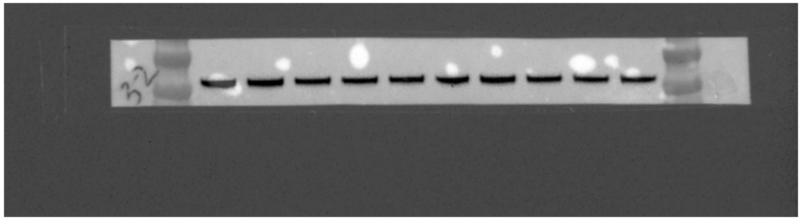

SIRT1

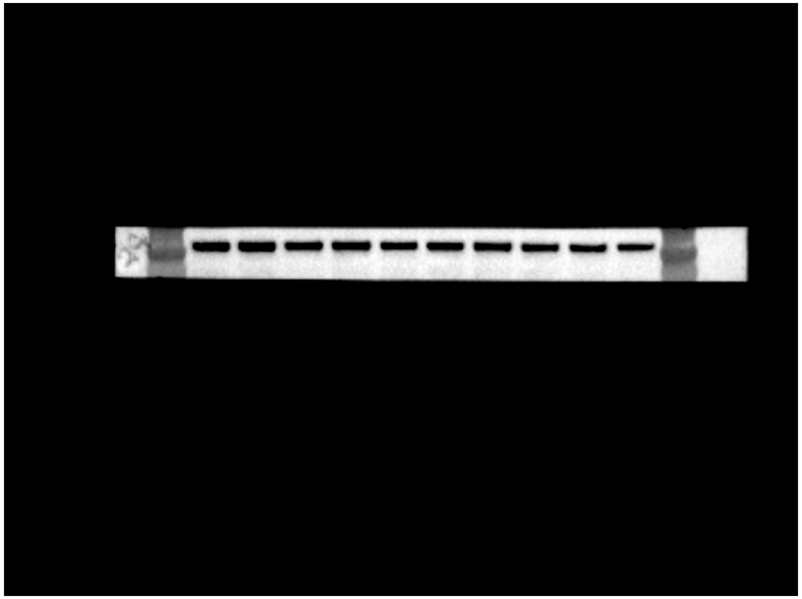

Figure 7A

p-AMPK (T183+T172)

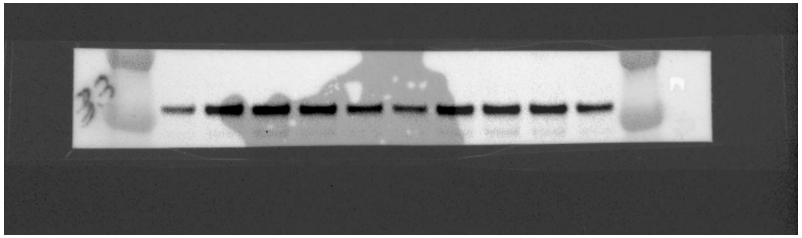

AMPK

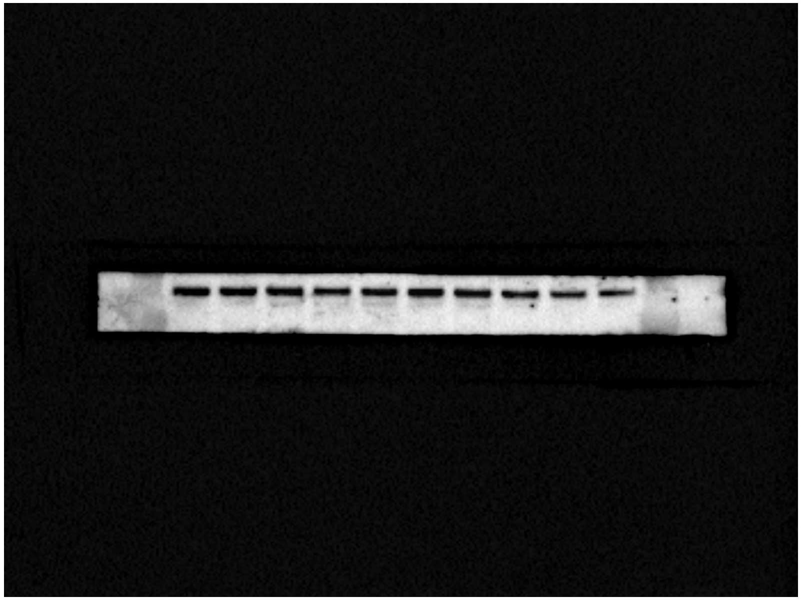

Figure 7A

p-mTOR

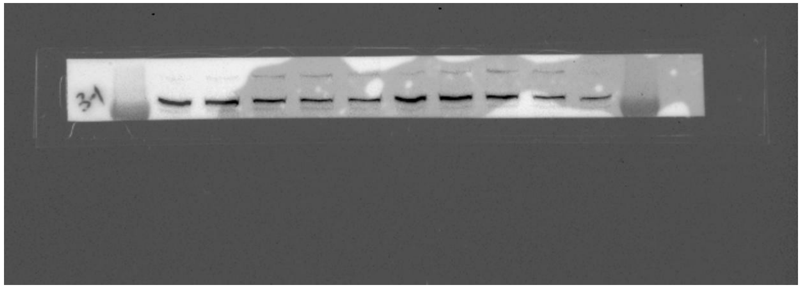

mTOR

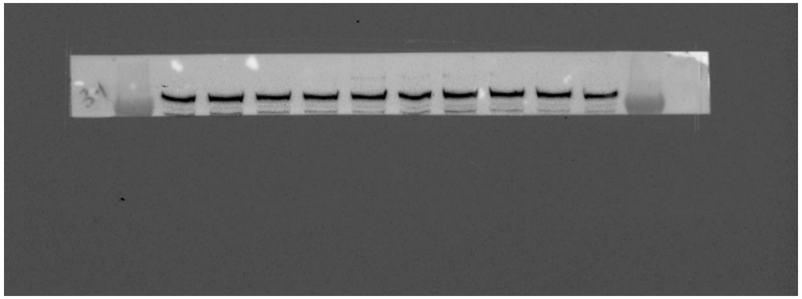

Figure 7A

GAPDH

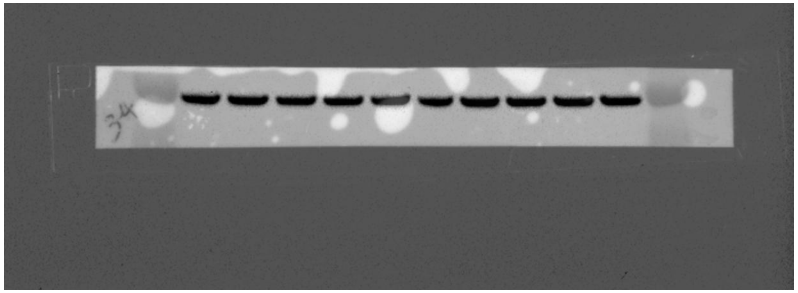

Figure 7B

LC3B

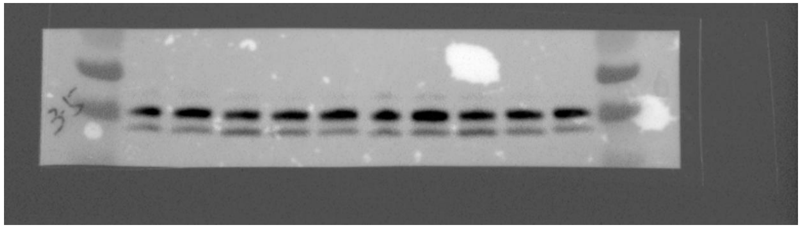

Figure 7B

p62

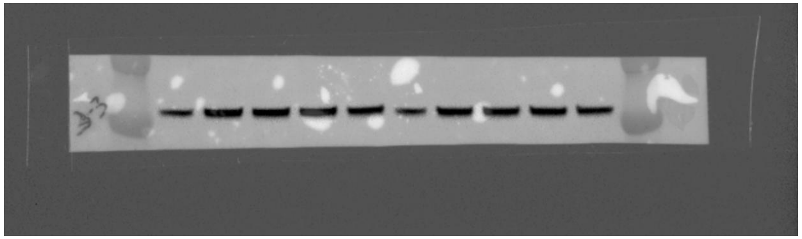

Figure 7B

GAPDH

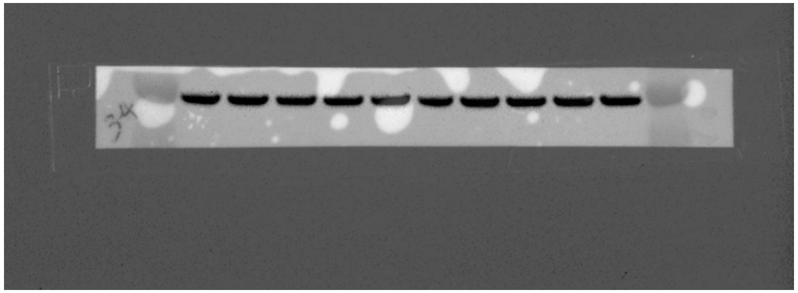

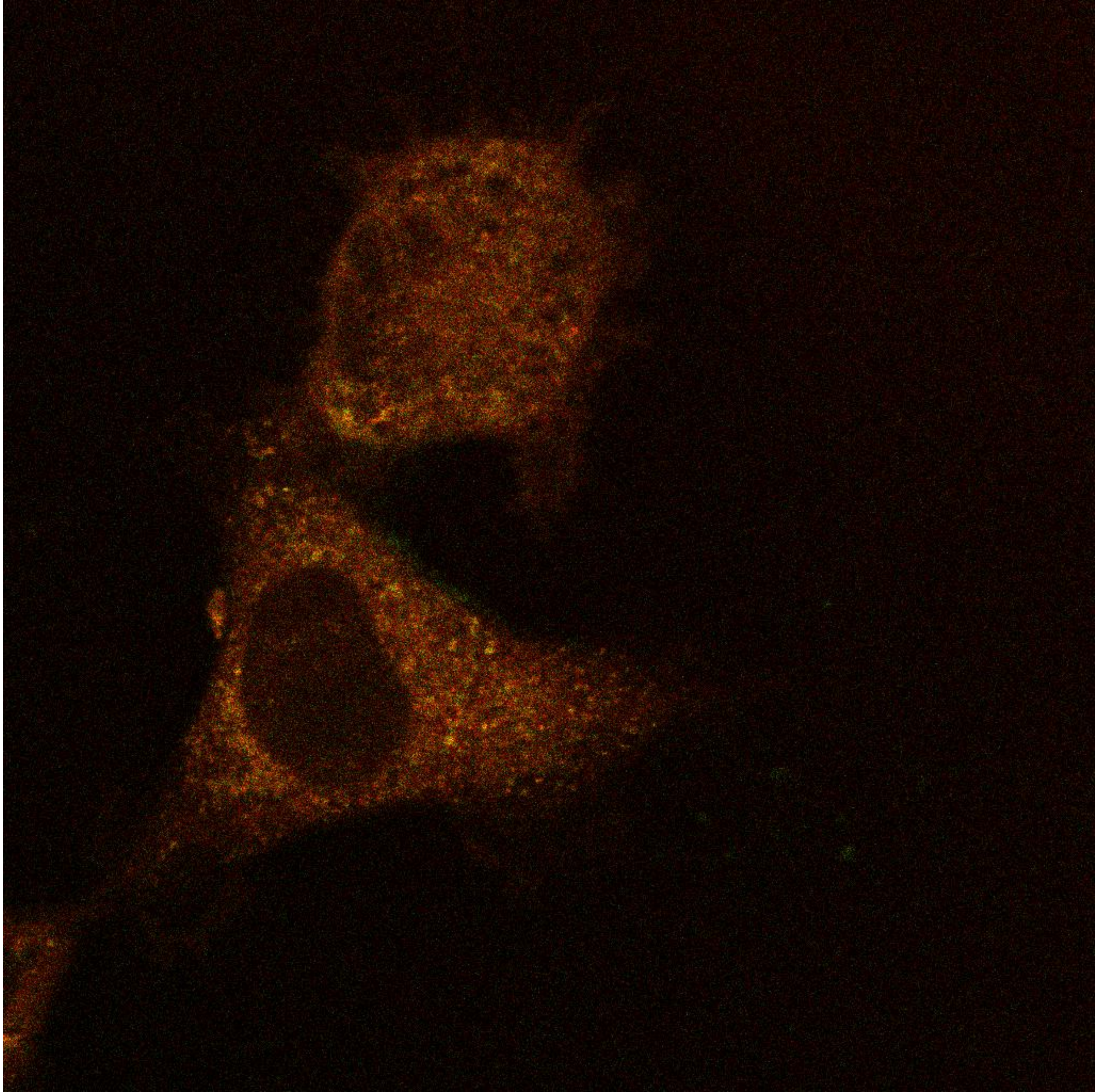

Figure 7C
WS5A Non-treated

Figure 7C
WS5A Non-treated

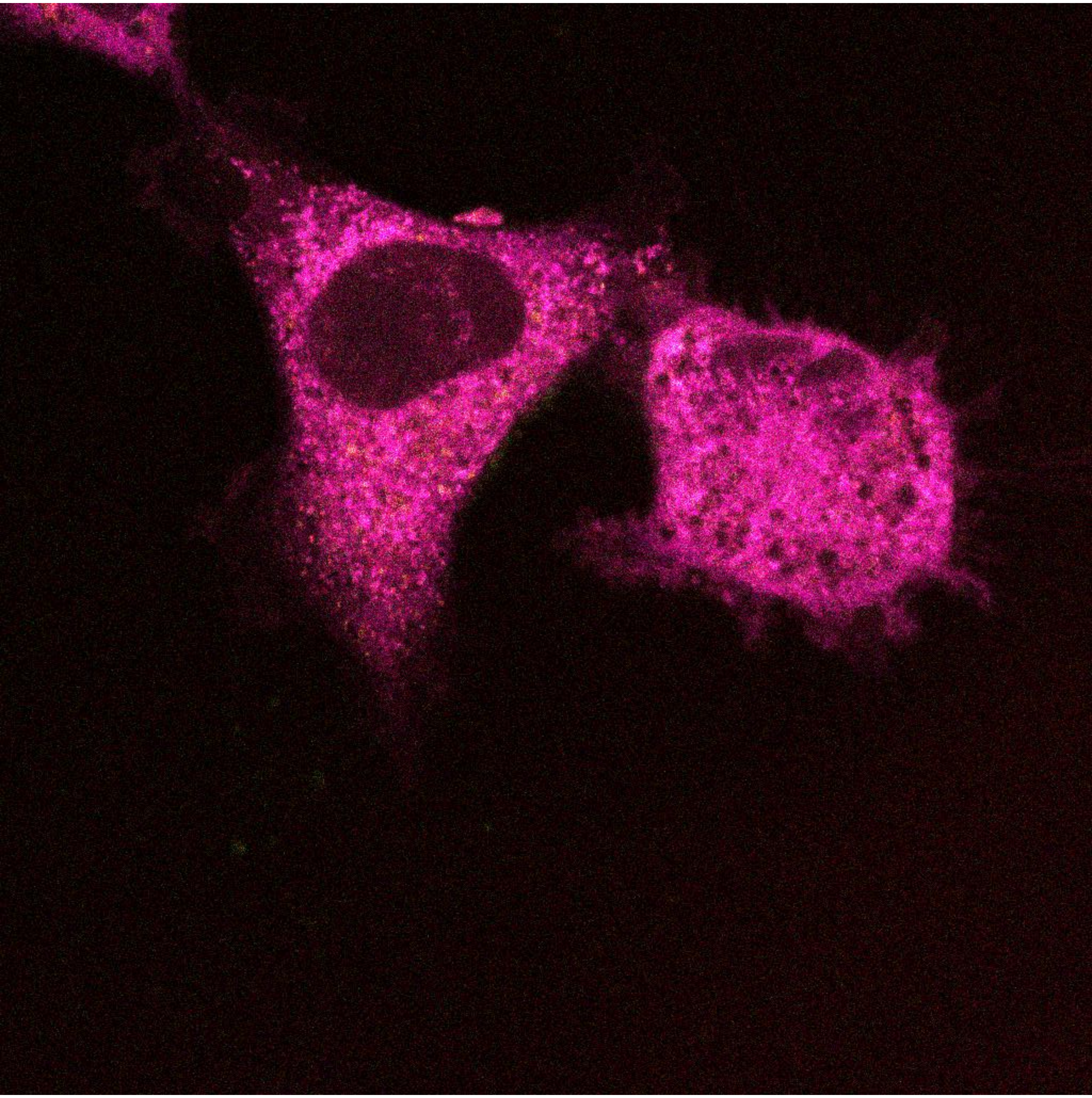

Figure 7C
WS5A Non-treated

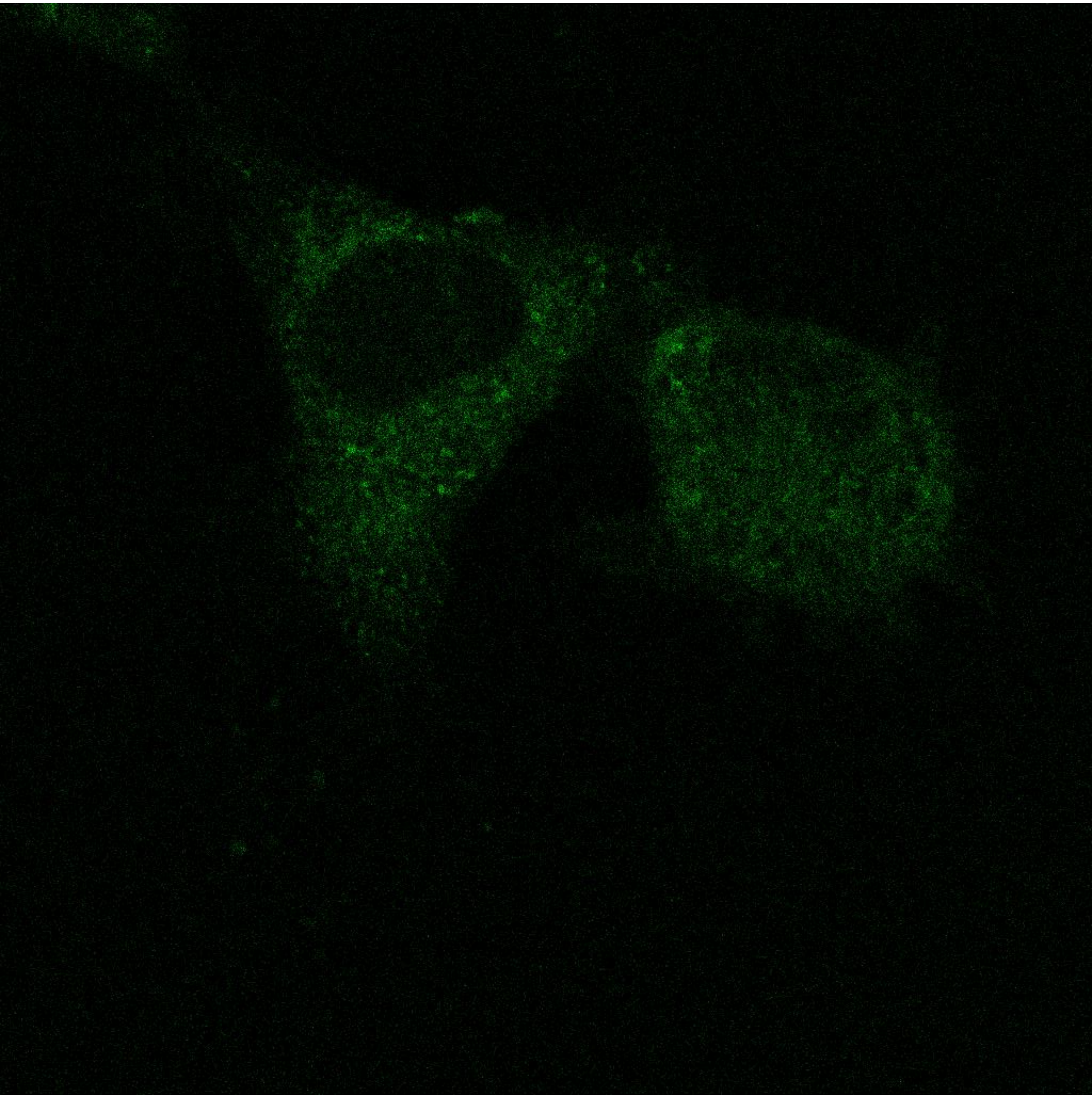

Figure 7C
WS5A Non-treated

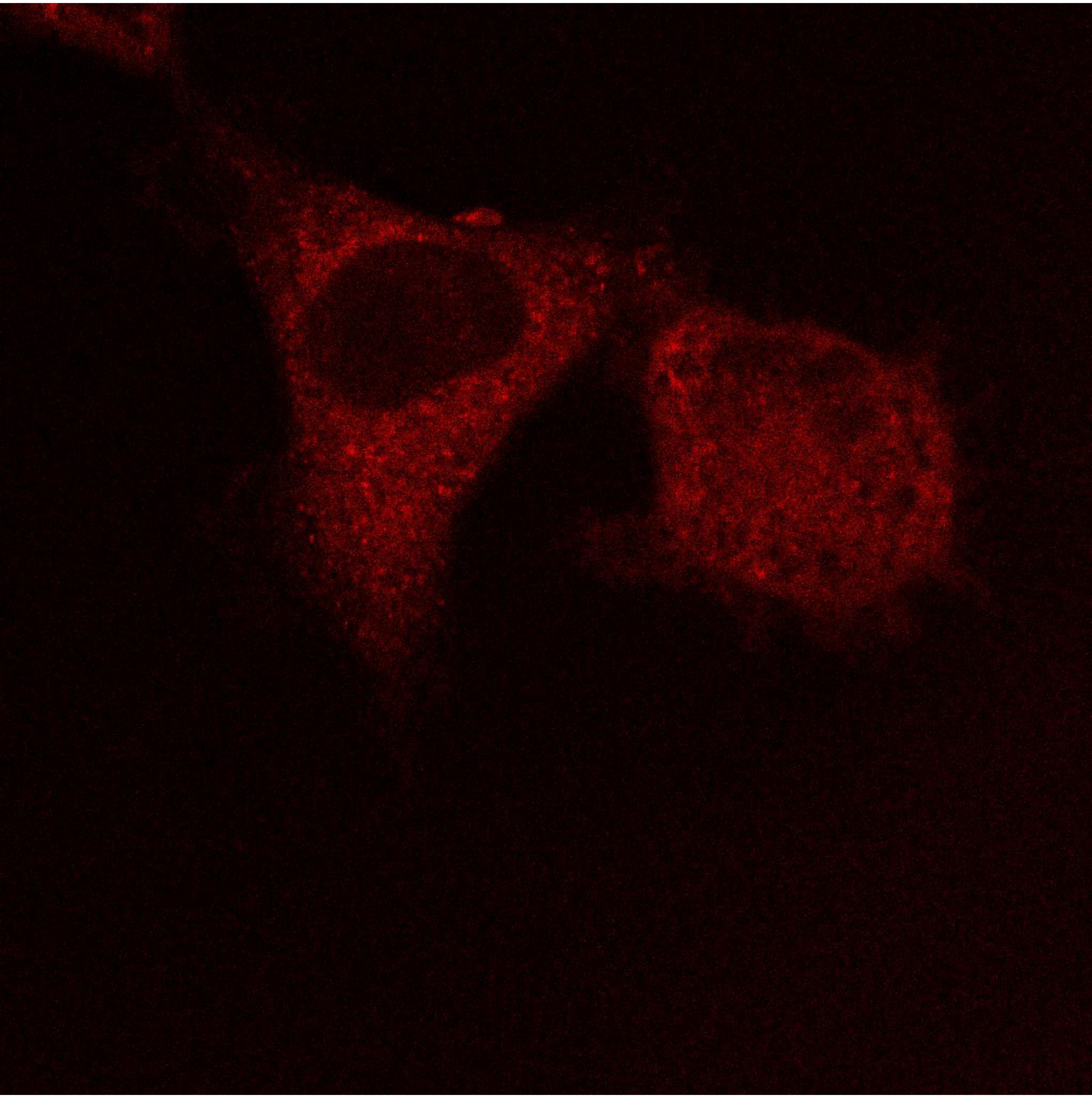

Figure 7C
WS5A Non-treated

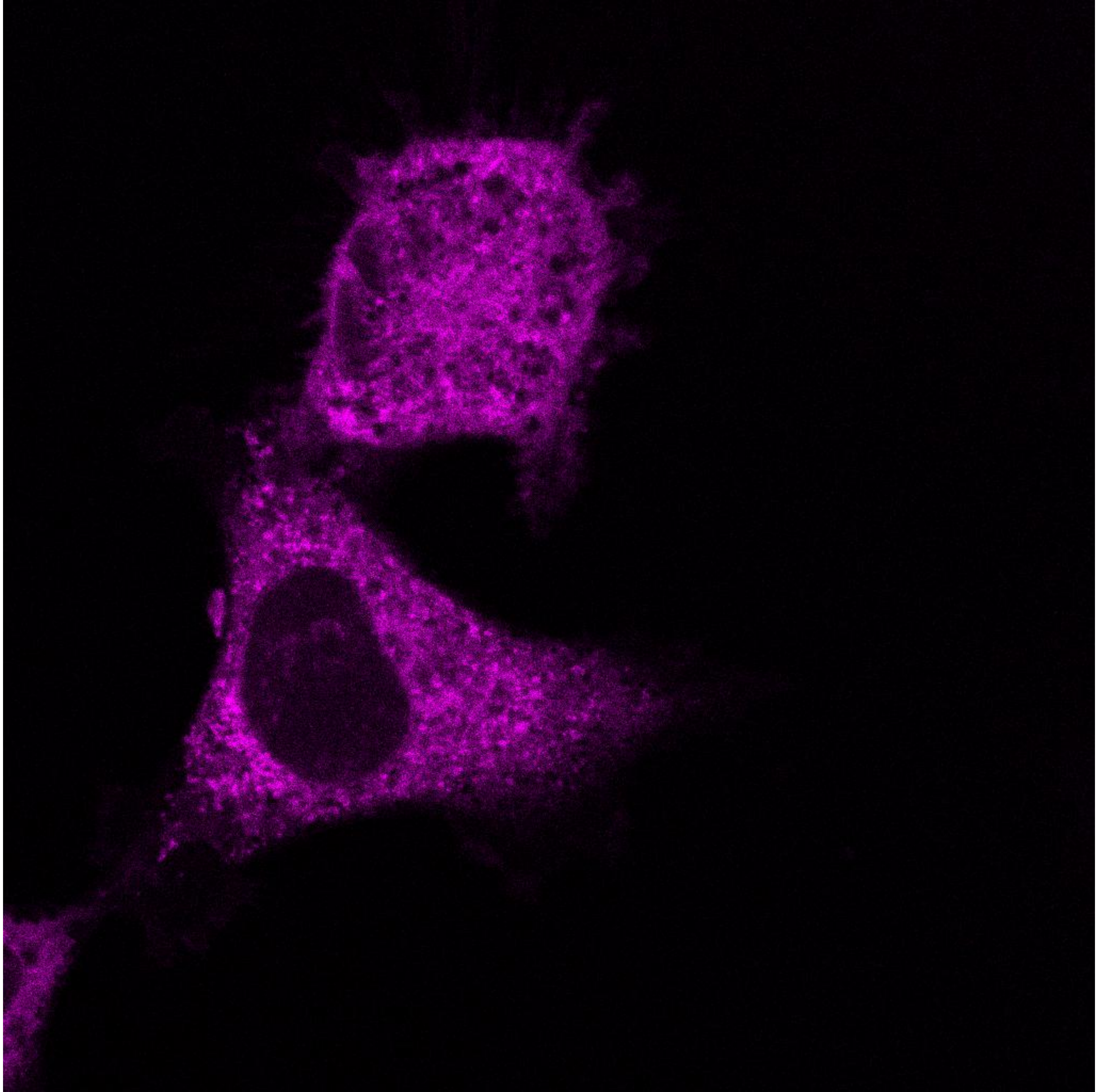

Figure 7C
WS5A NR 0.5mM+M2.5mM

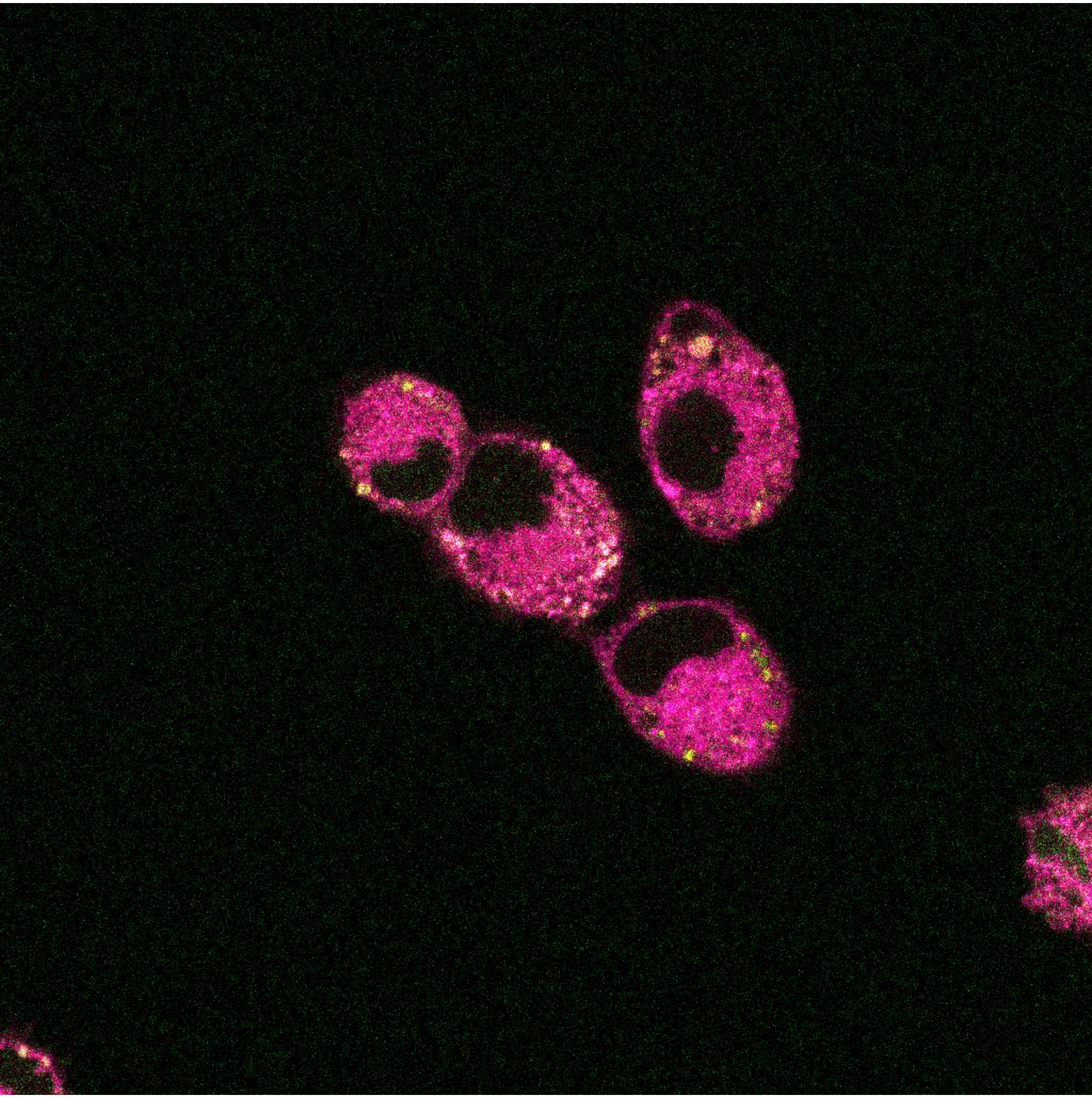

Figure 7C
WS5A NR 0.5mM+M2.5mM

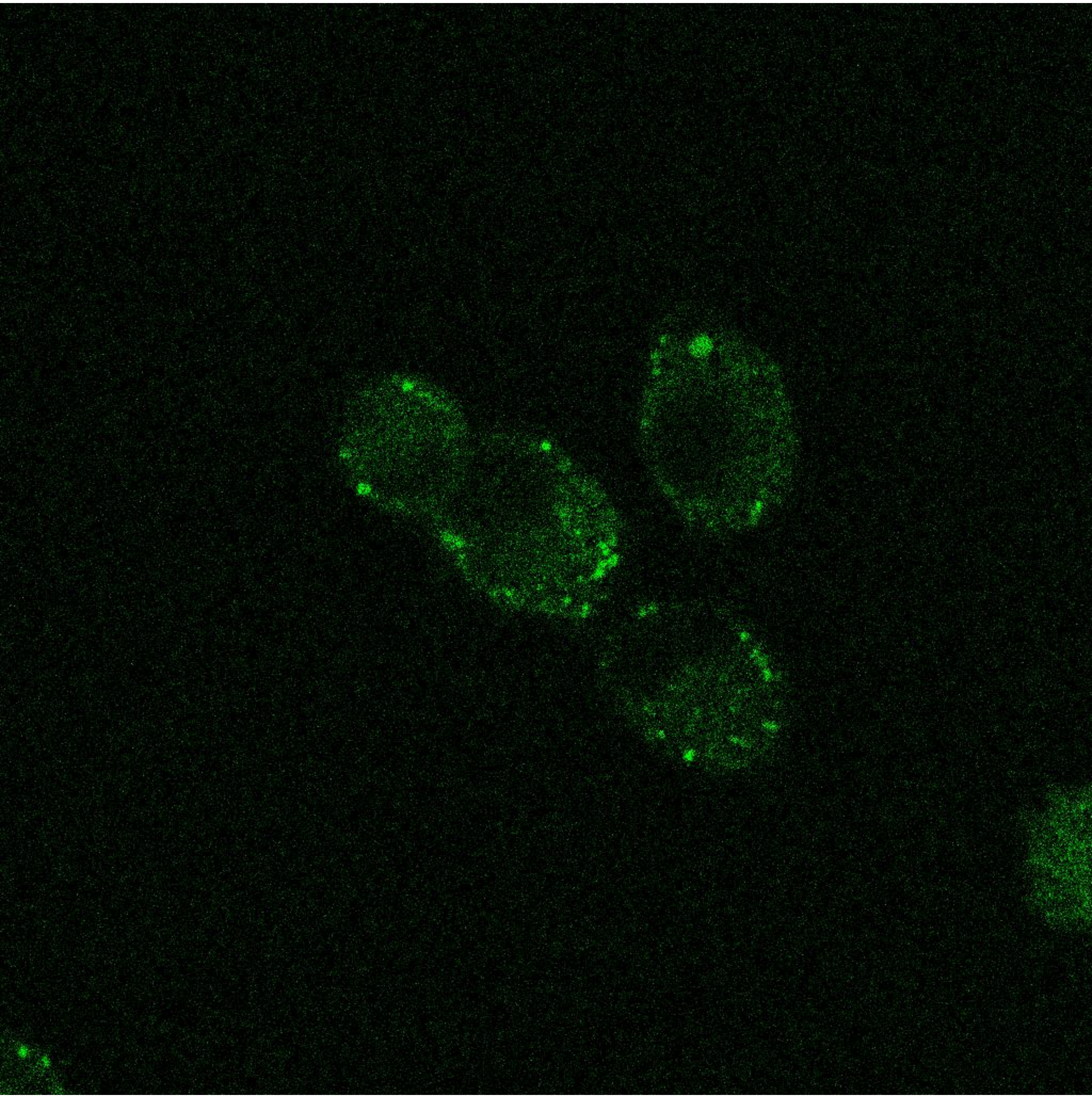

Figure 7C
WS5A NR 0.5mM+M2.5mM

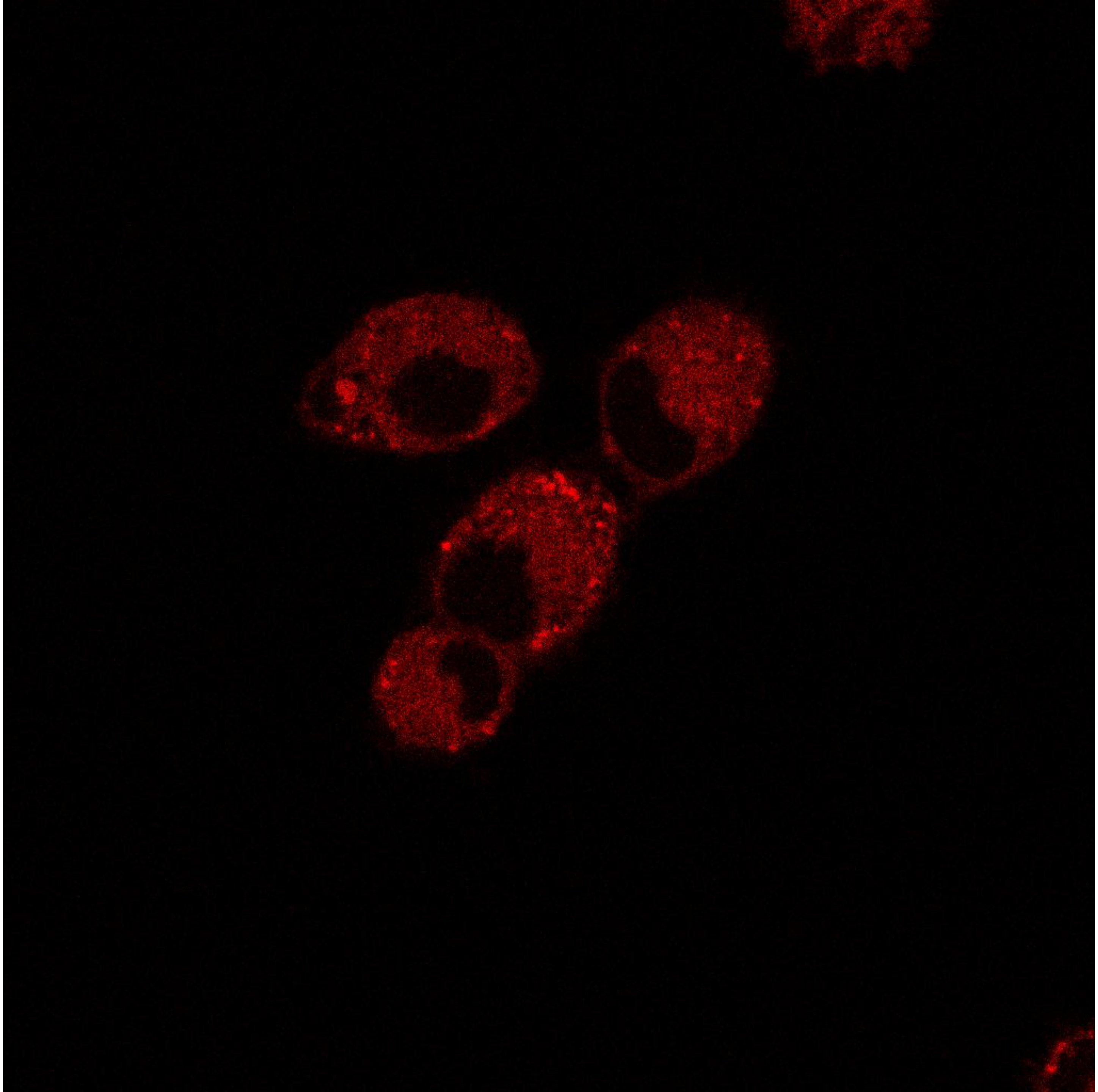

Figure 7C
WS5A NR 0.5mM+M2.5mM

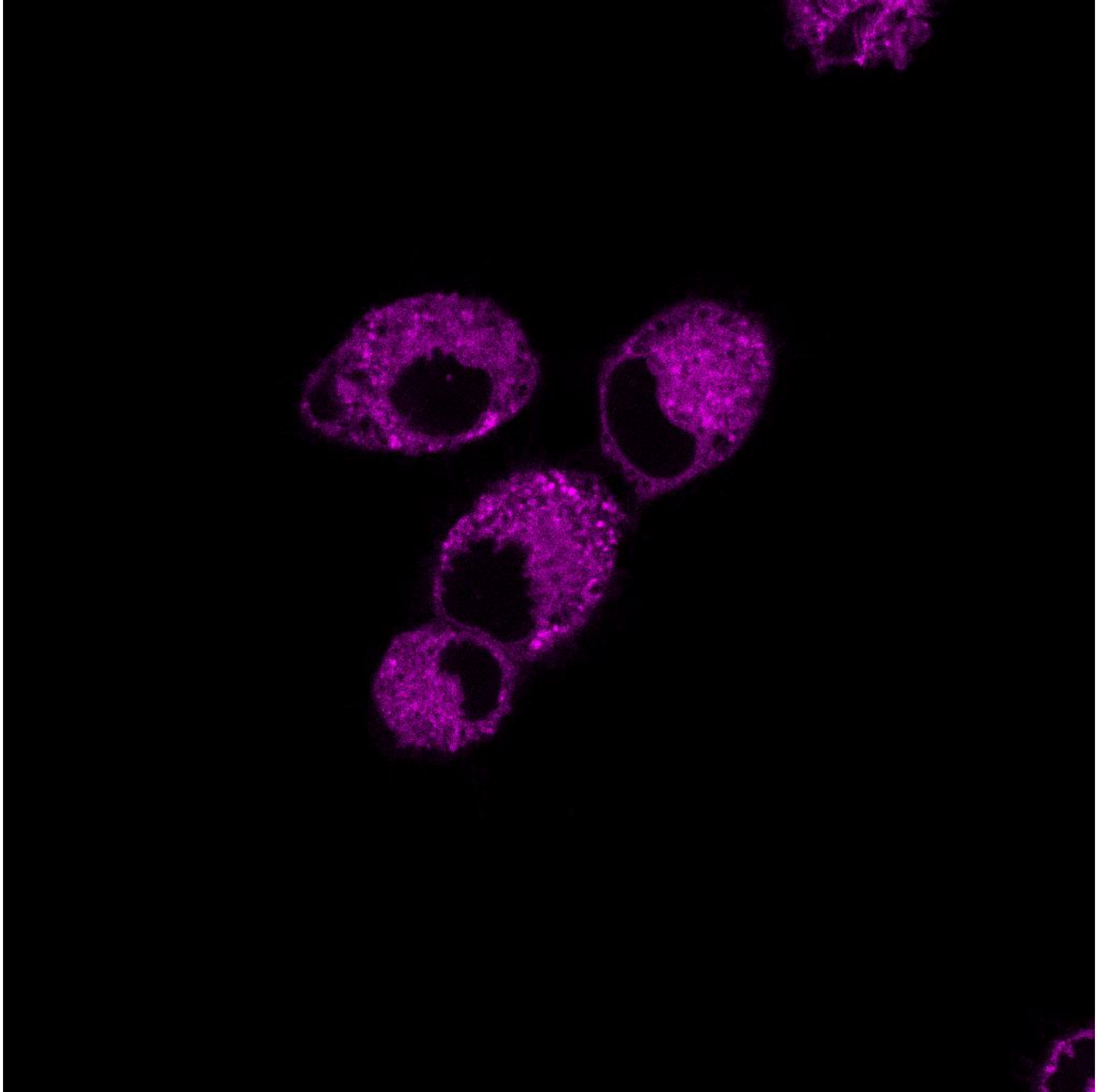

Figure 7C
WS5A NR 0.5mM+M2.5mM

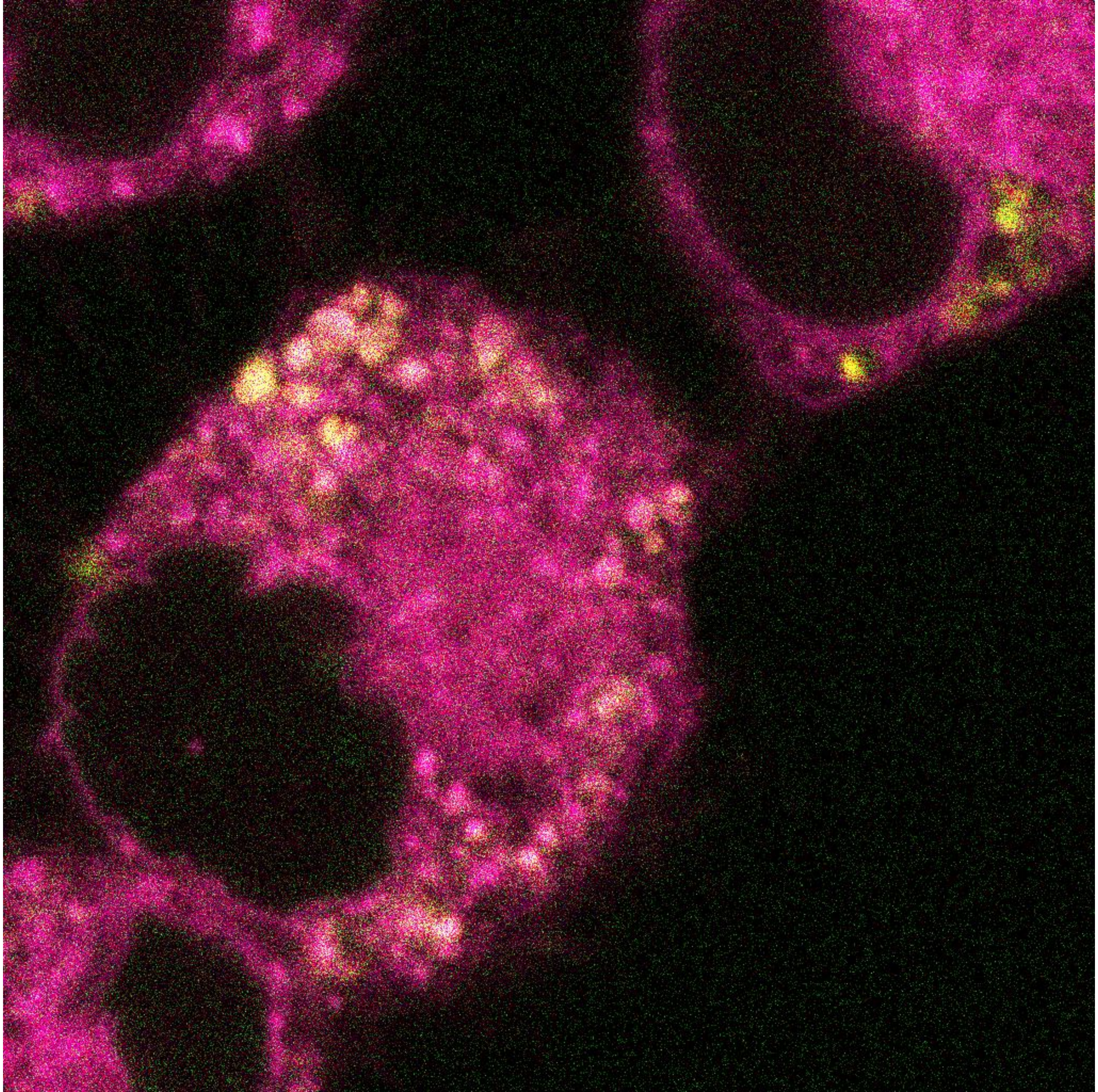

Figure 7C
WS5A NR 0.5mM+M2.5mM

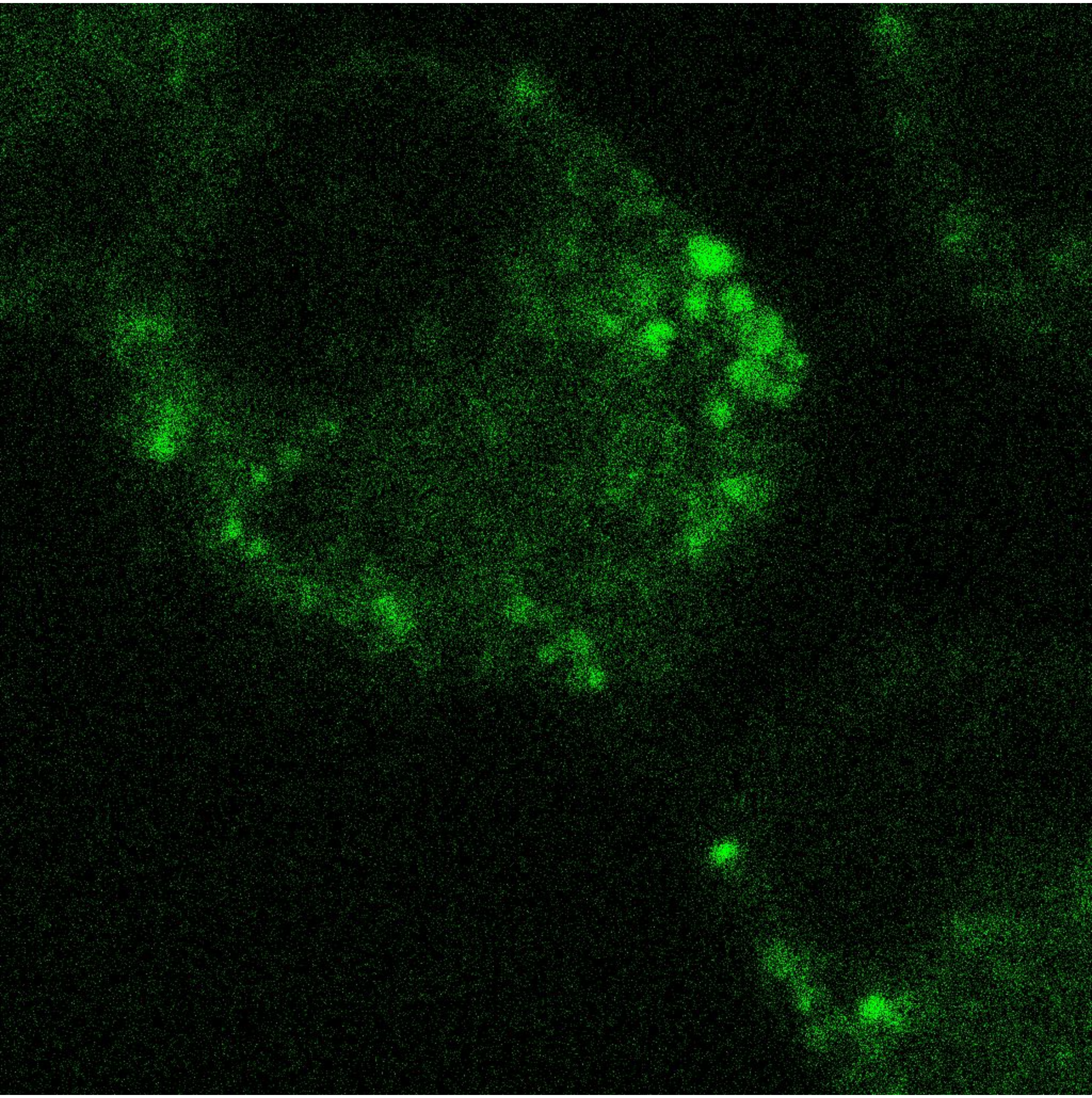

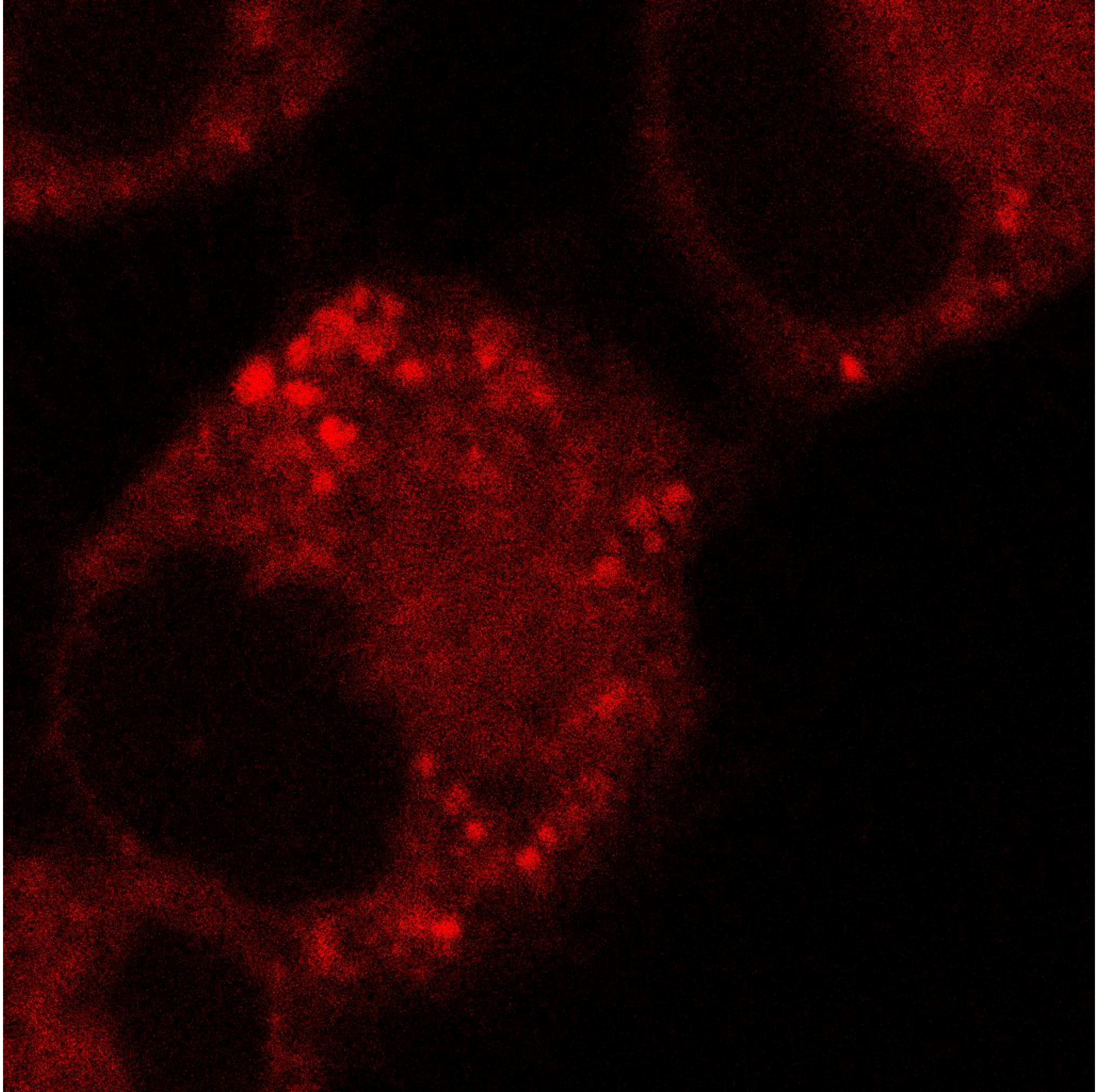

Figure 7C
WS5A NR 0.5mM+M2.5mM

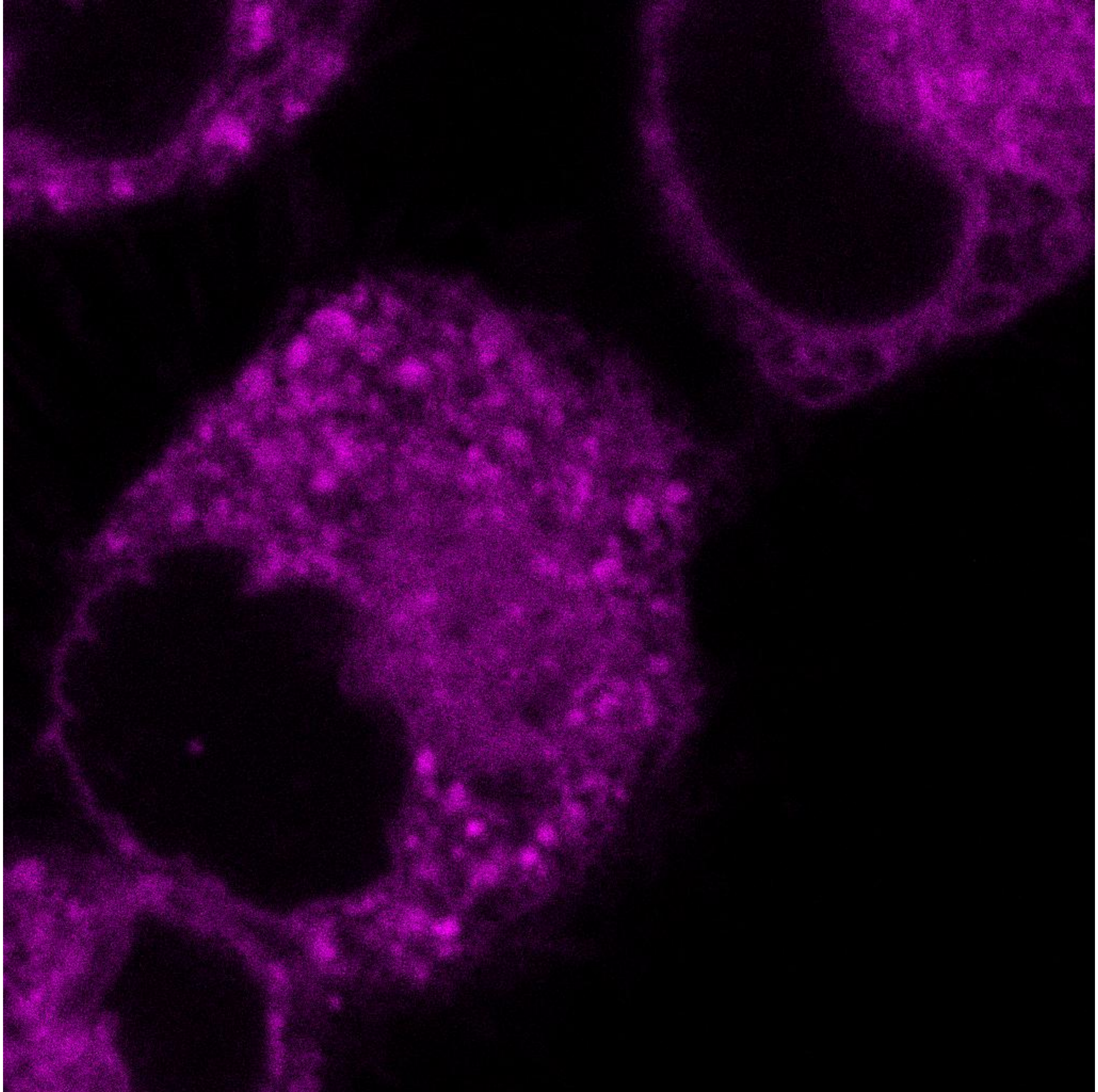

Figure 7C
WS5A NR 0.5mM+M2.5mM

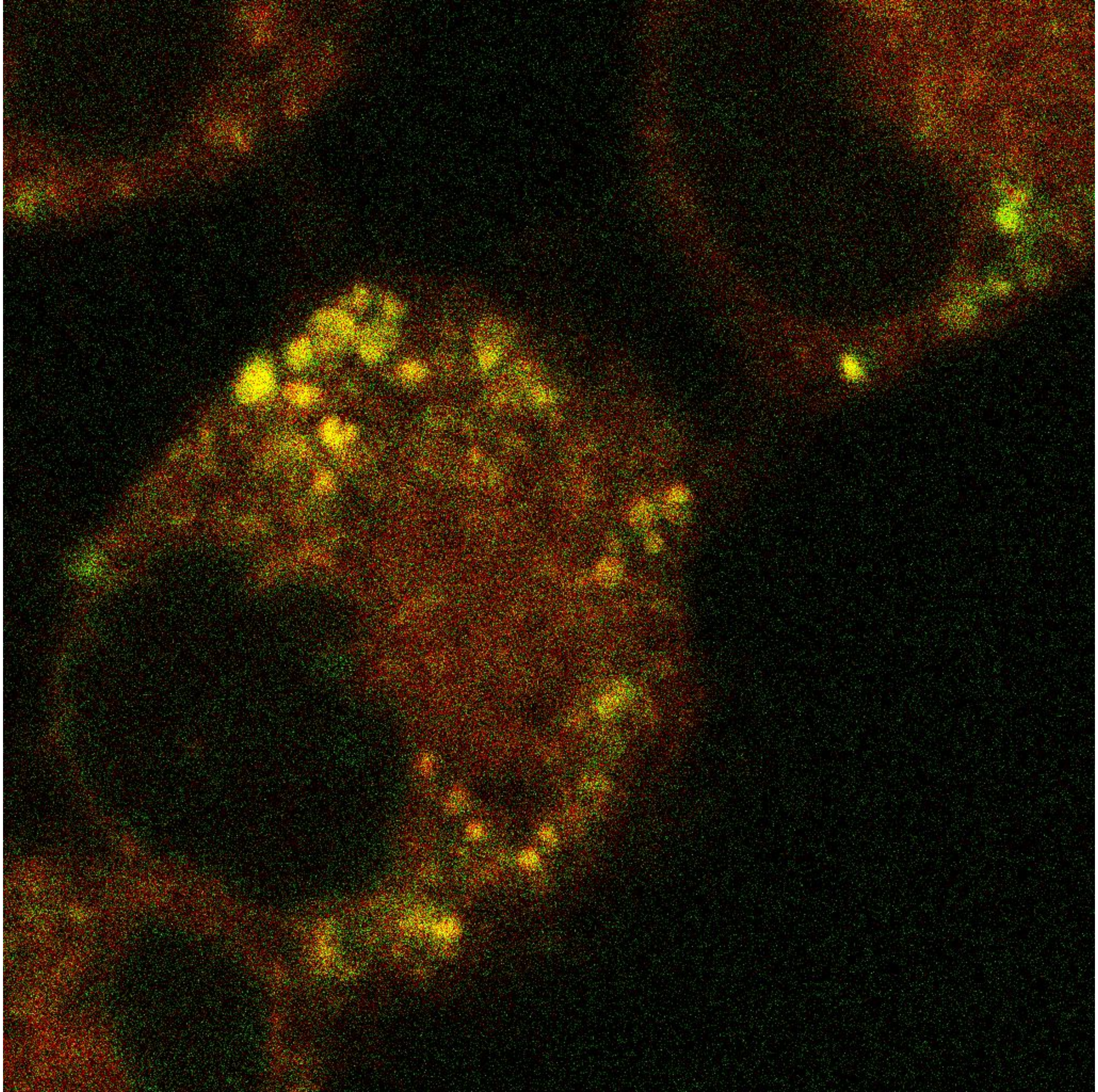

Figure 7C
WS5A NR 0.5mM+M2.5mM

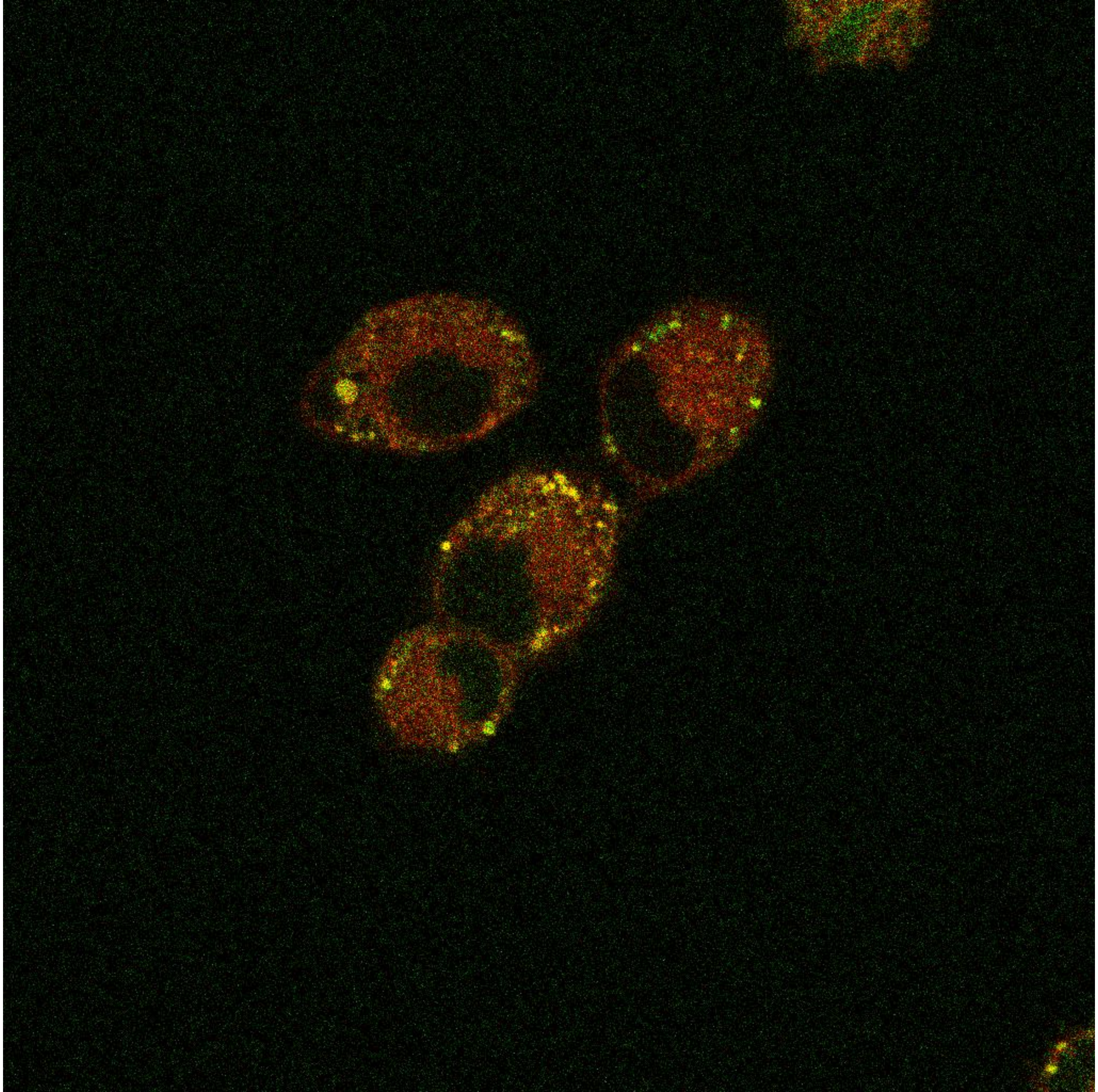

Figure 7C
WS5A NR 0.5mM+M2.5mM

Figure 7C
CP2A Non-treated

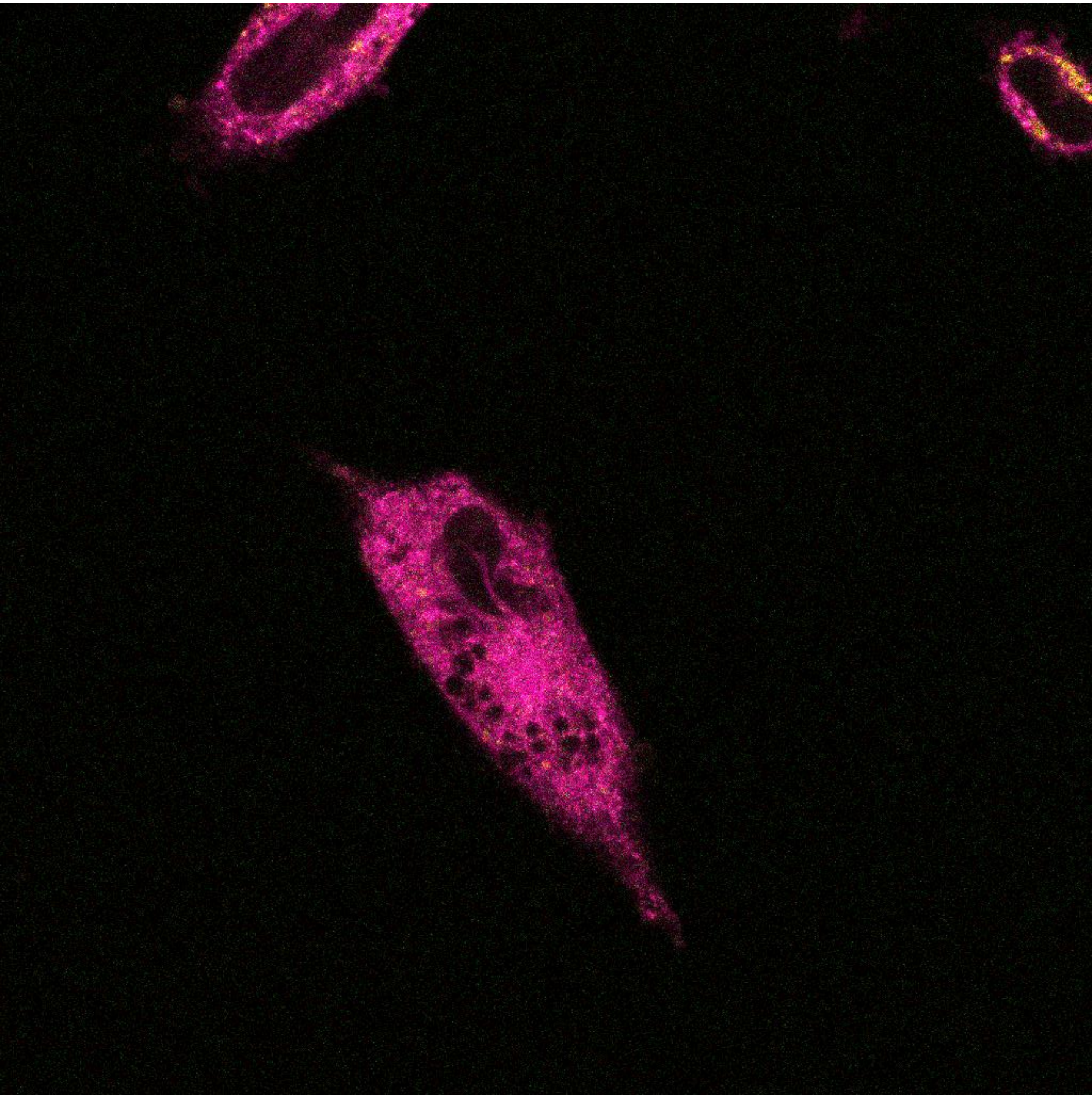

Figure 7C
CP2A Non-treated

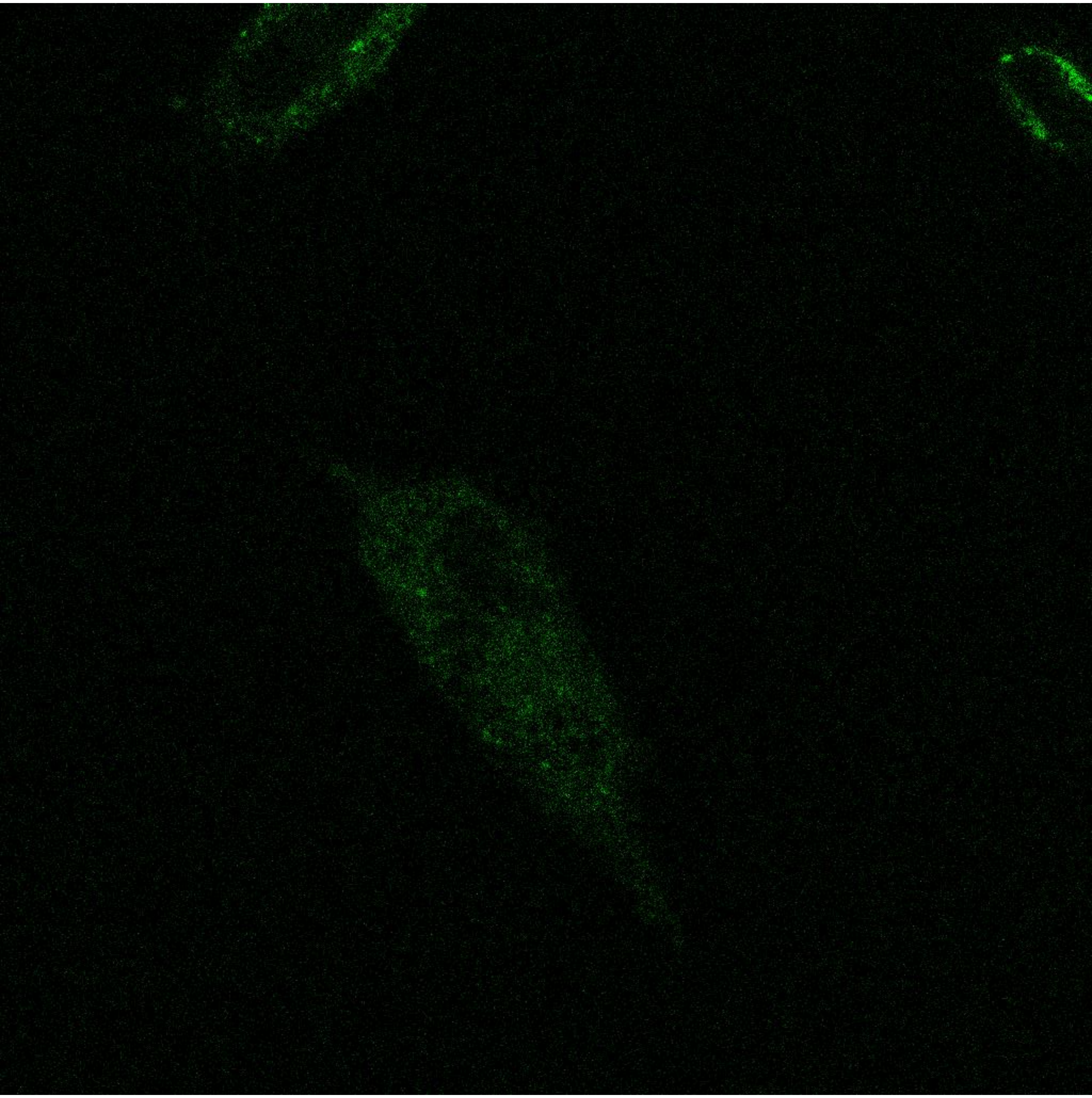

Figure 7C
CP2A Non-treated

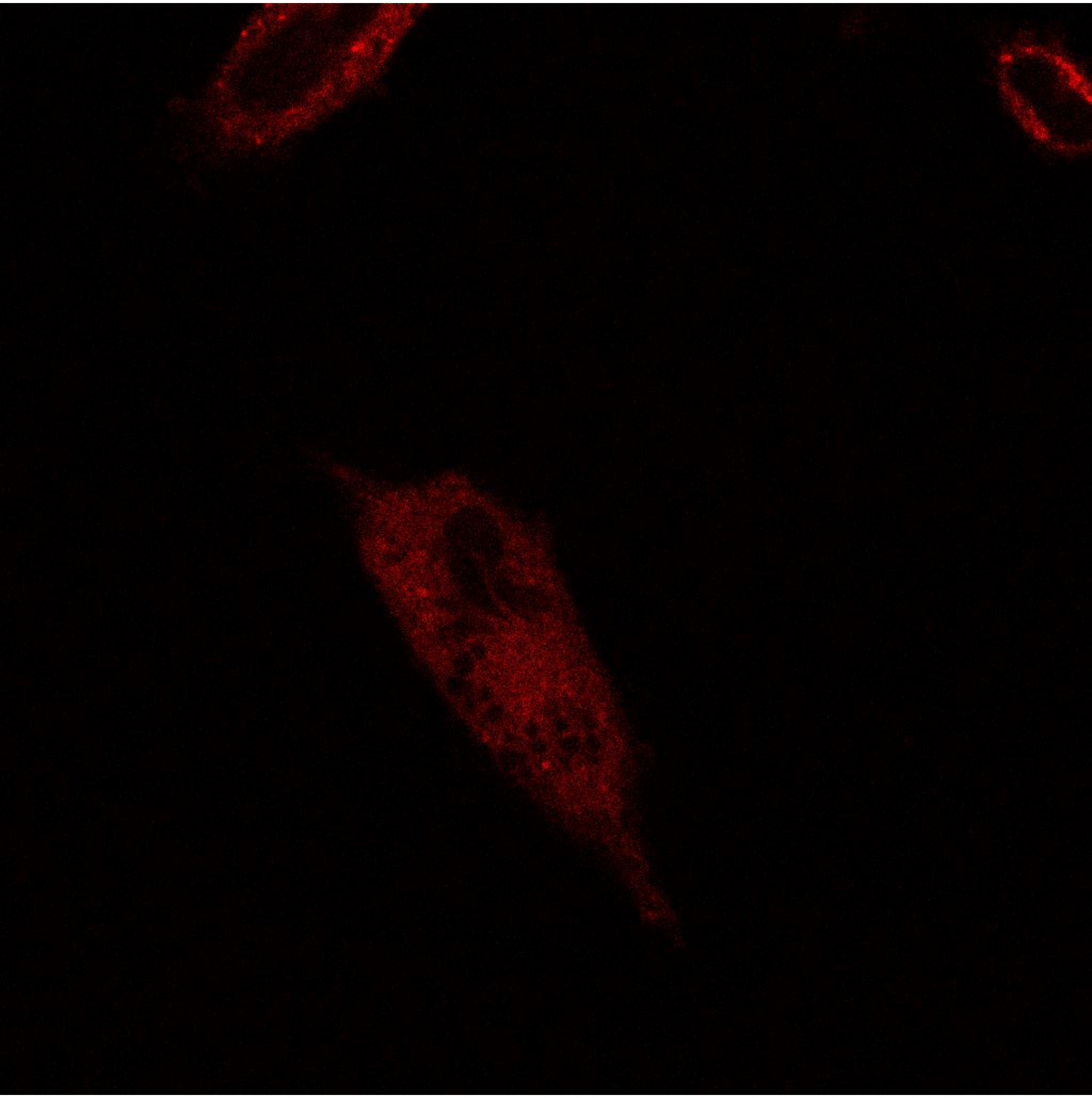

Figure 7C
CP2A Non-treated

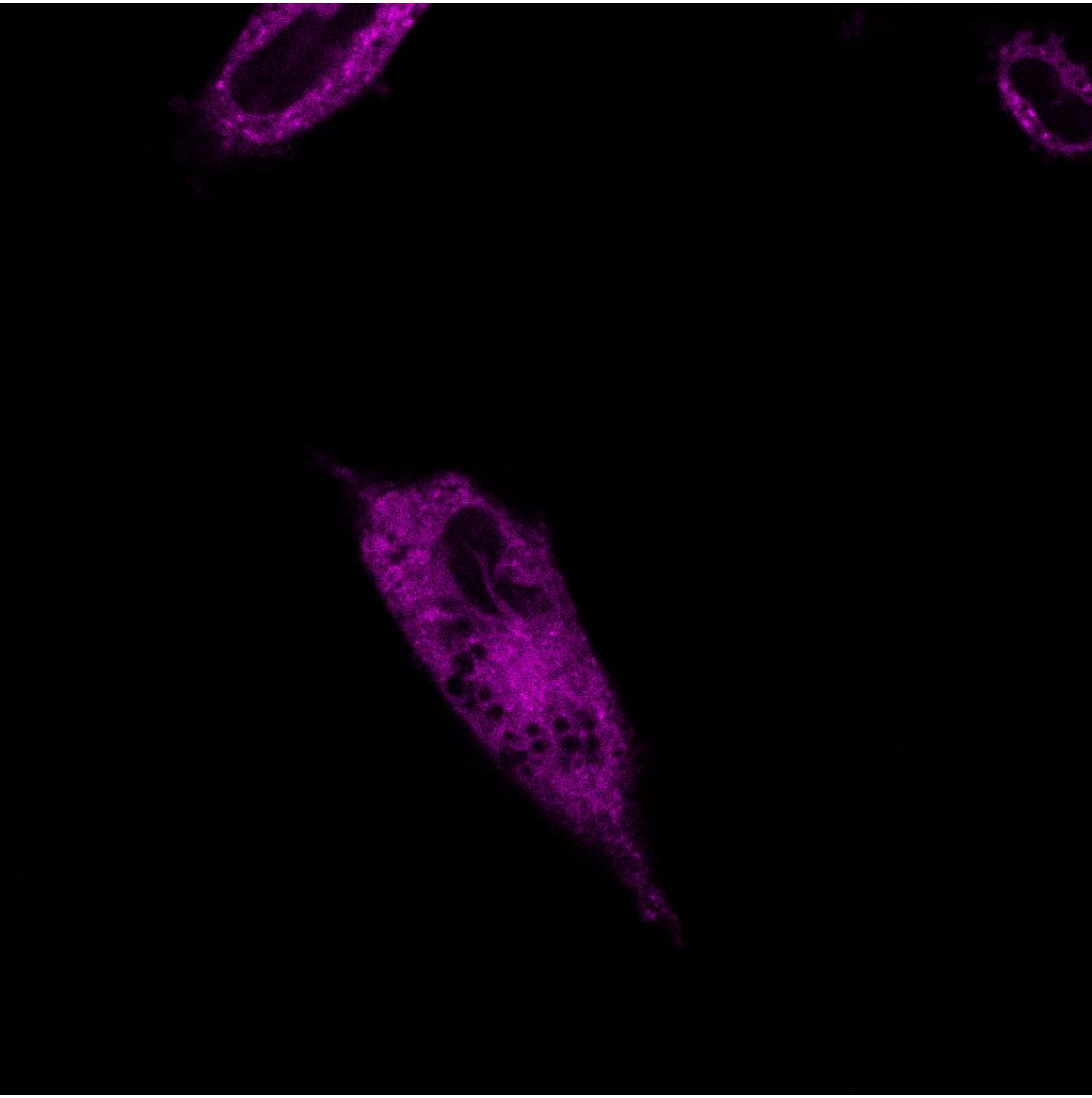

Figure 7C
CP2A Non-treated

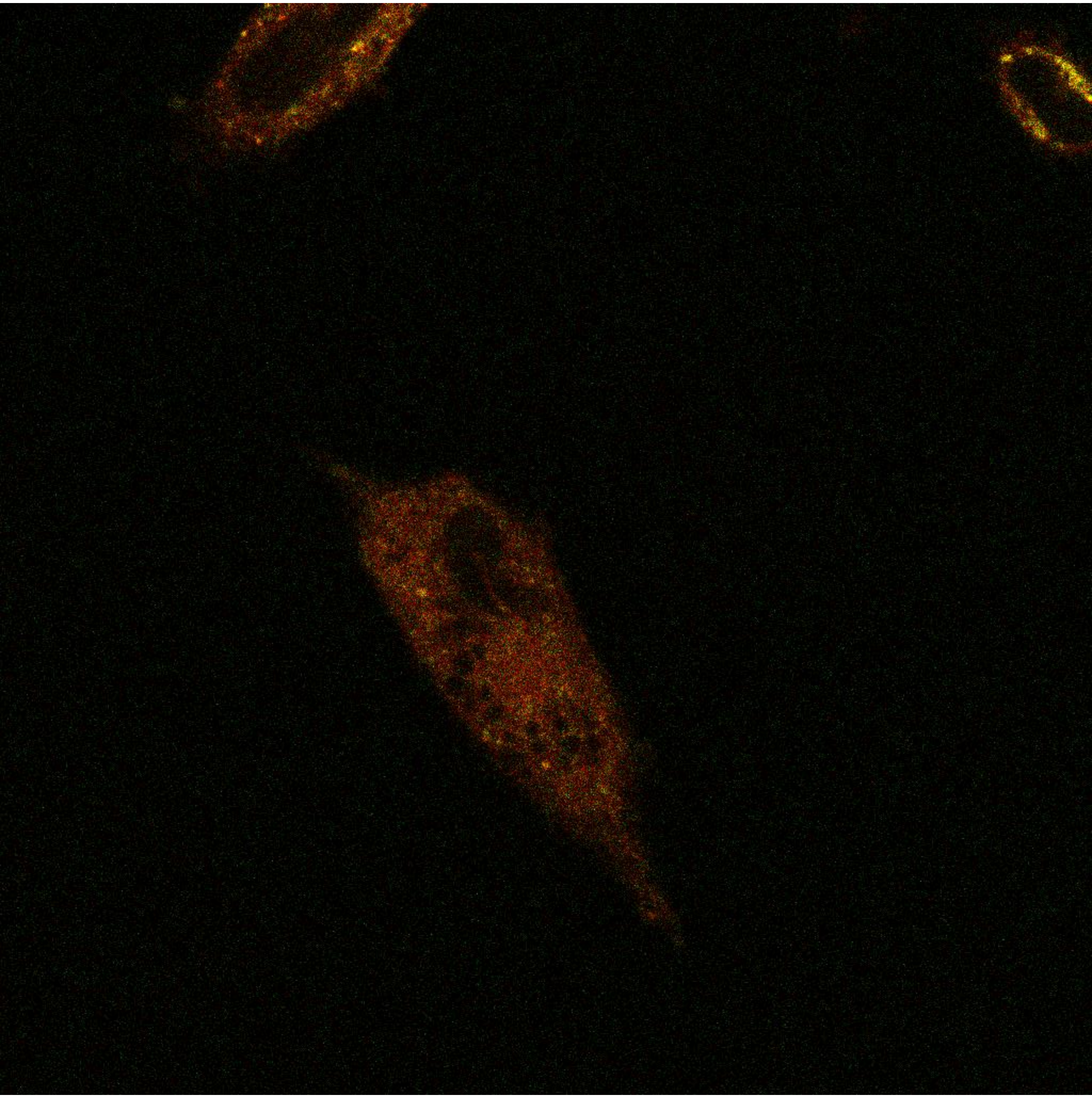

Figure 7C
CP2A NR 0.5mM+M2.5mM

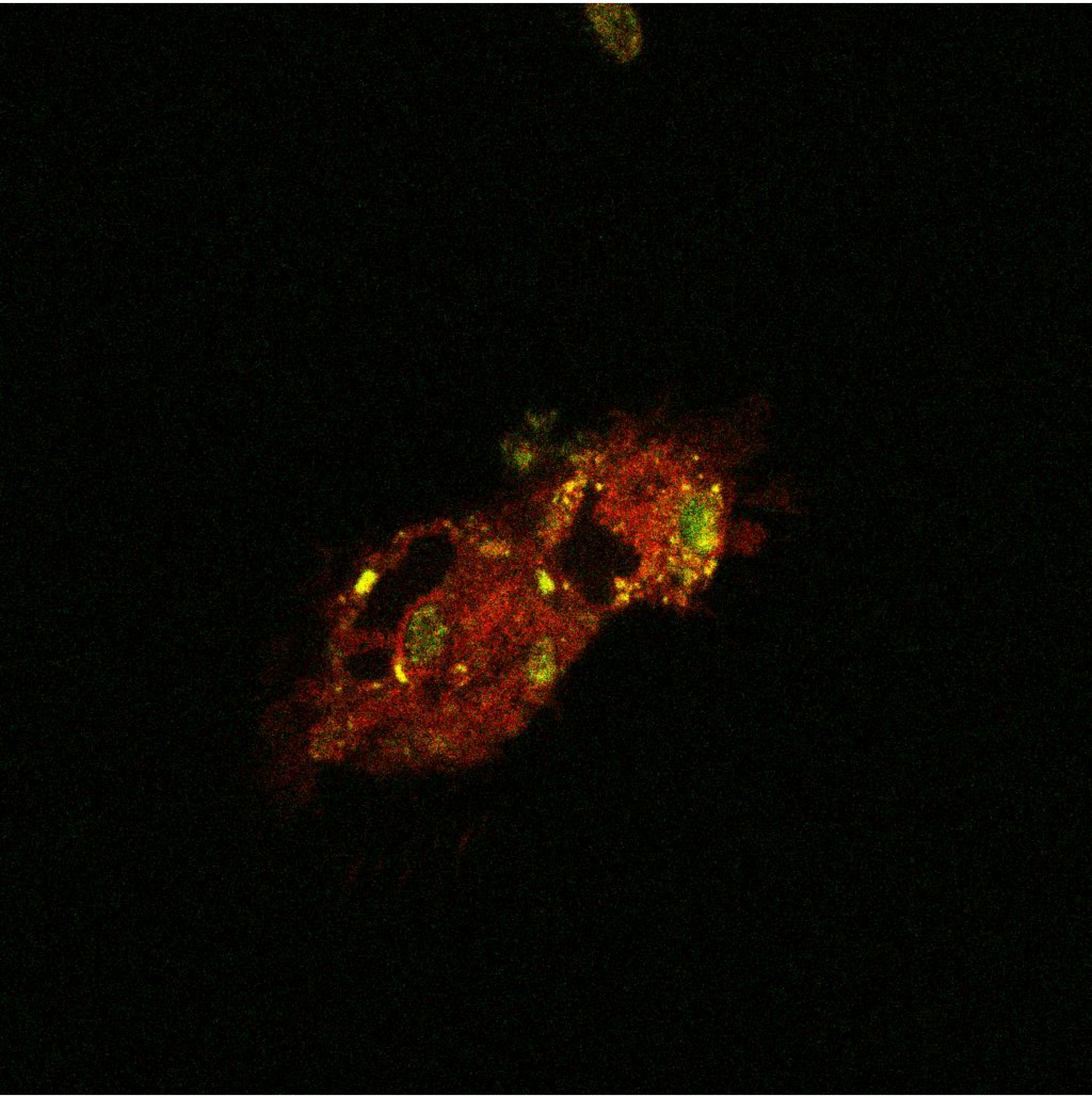

Figure 7C
CP2A NR 0.5mM+M2.5mM

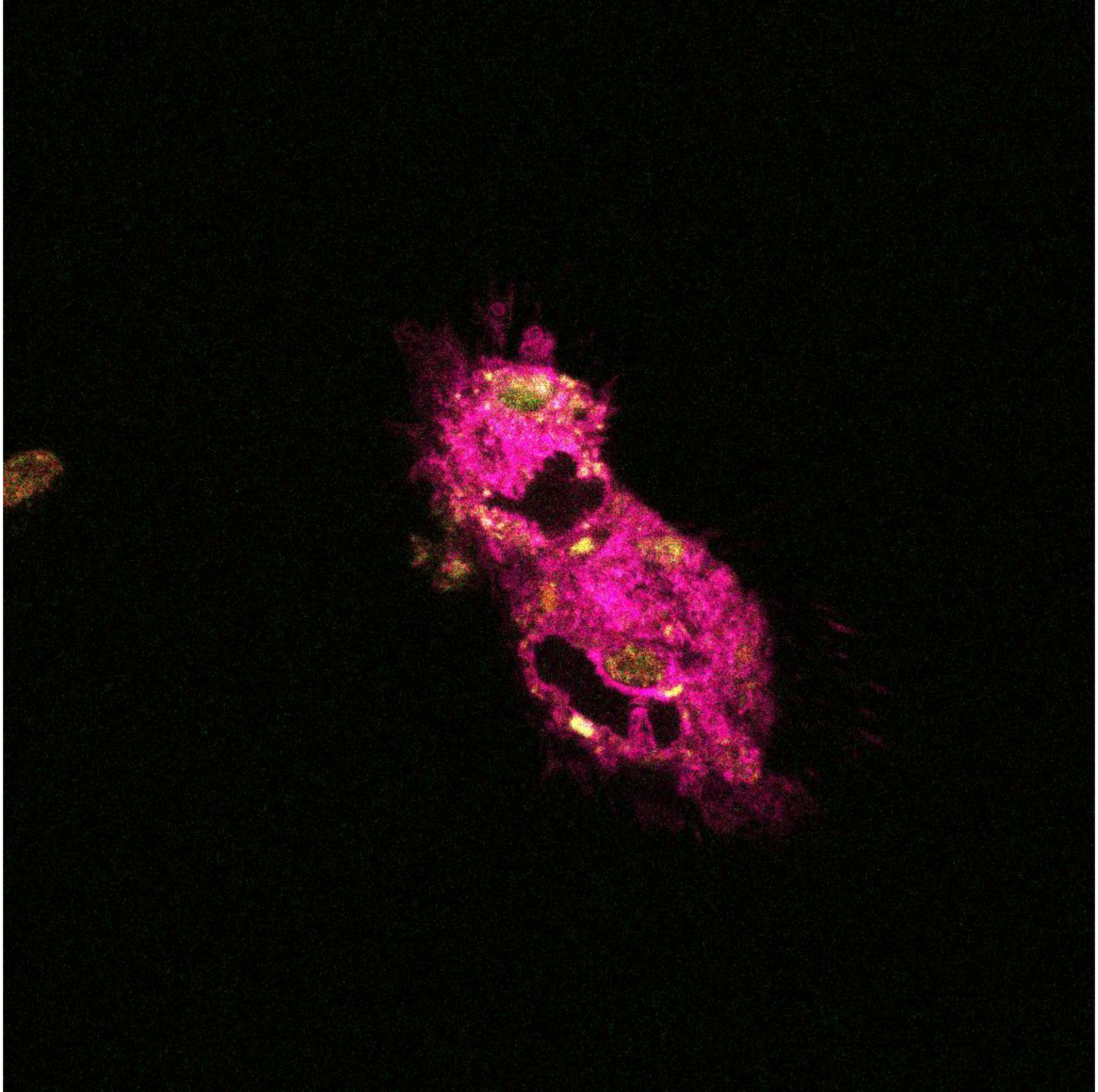

Figure 7C
CP2A NR 0.5mM+M2.5mM

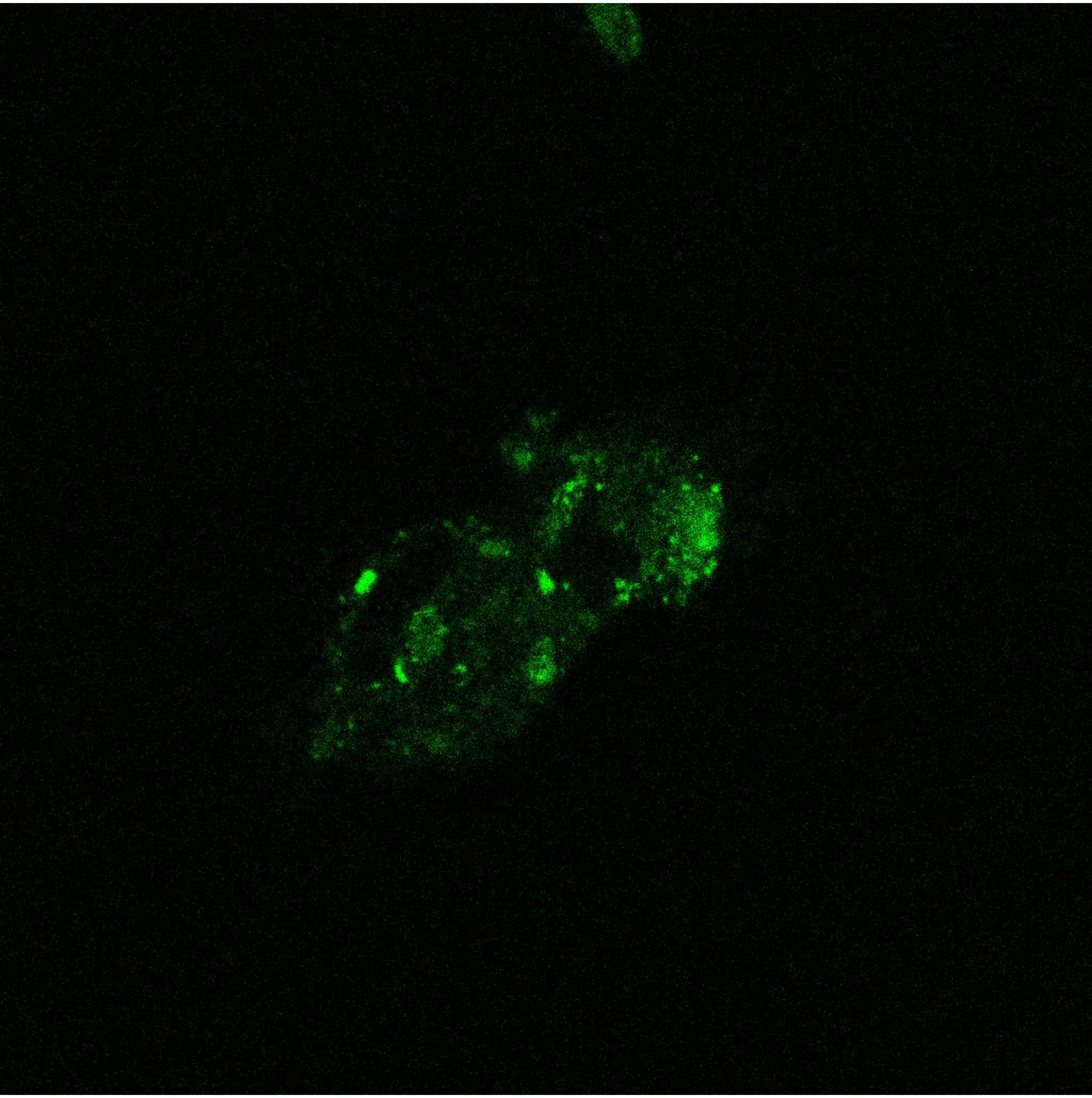

Figure 7C
CP2A NR 0.5mM+M2.5mM

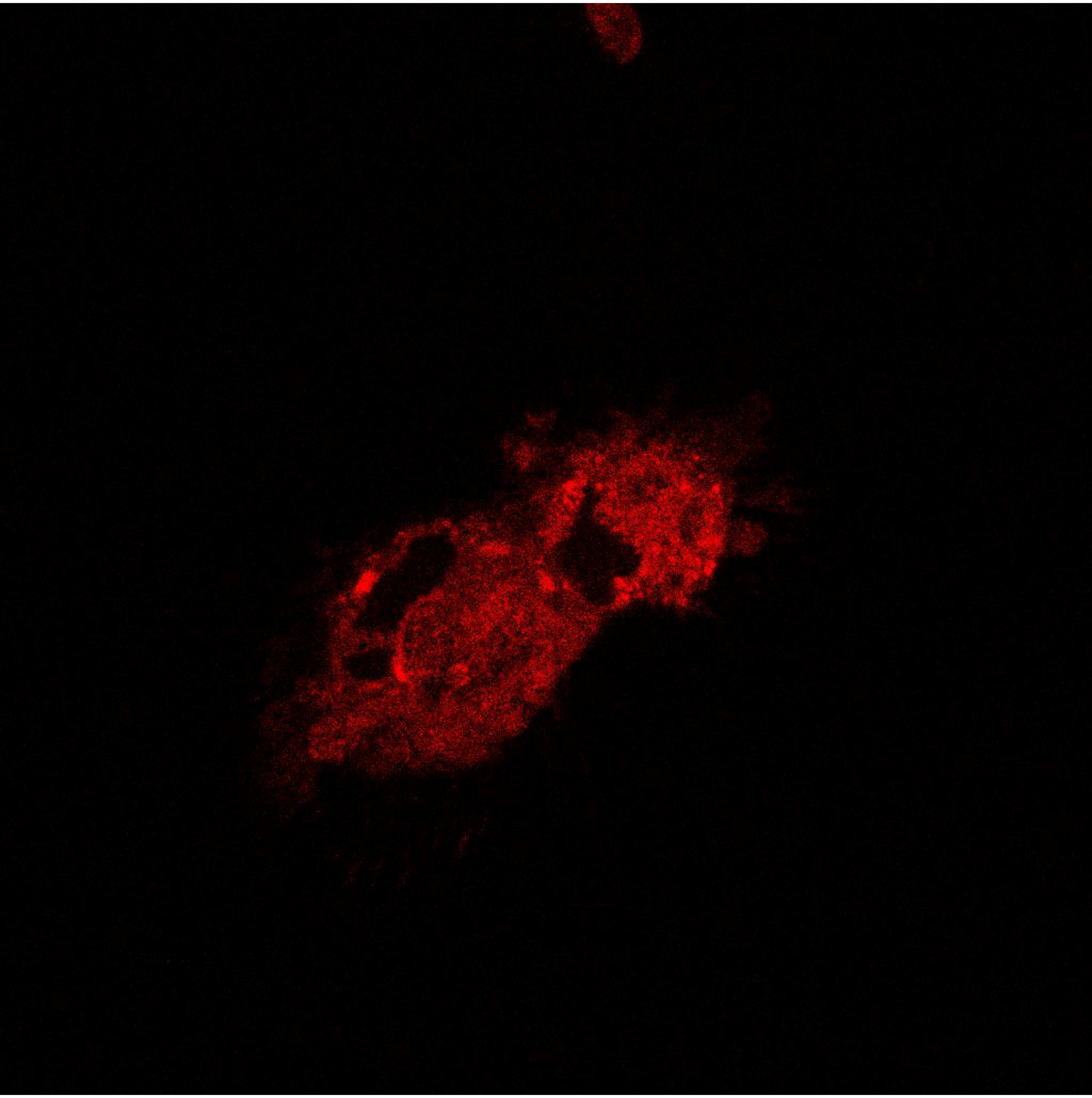

Figure 7C
CP2A NR 0.5mM+M2.5mM

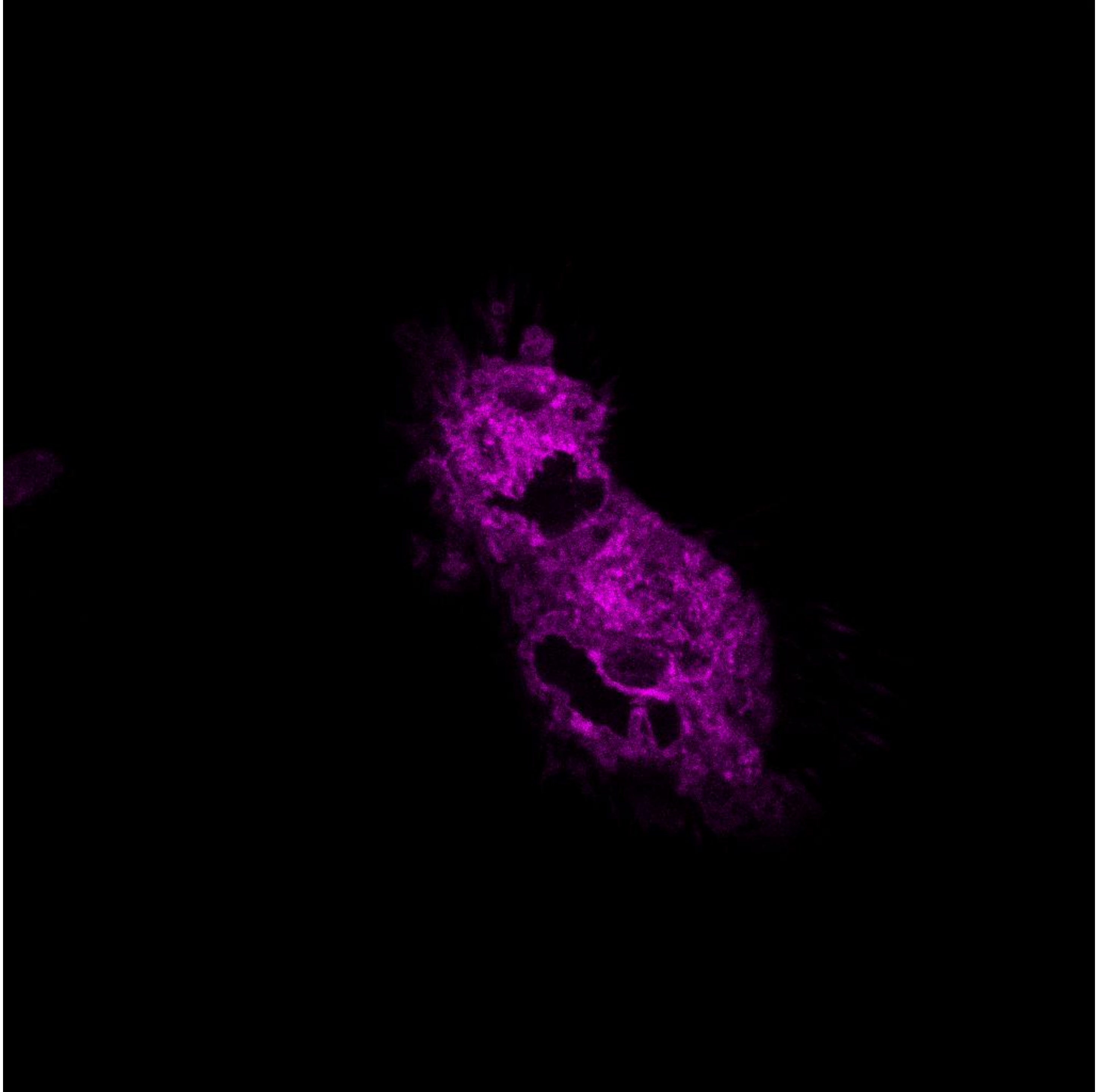

Figure 7C
CP2A NR 0.5mM+M2.5mM

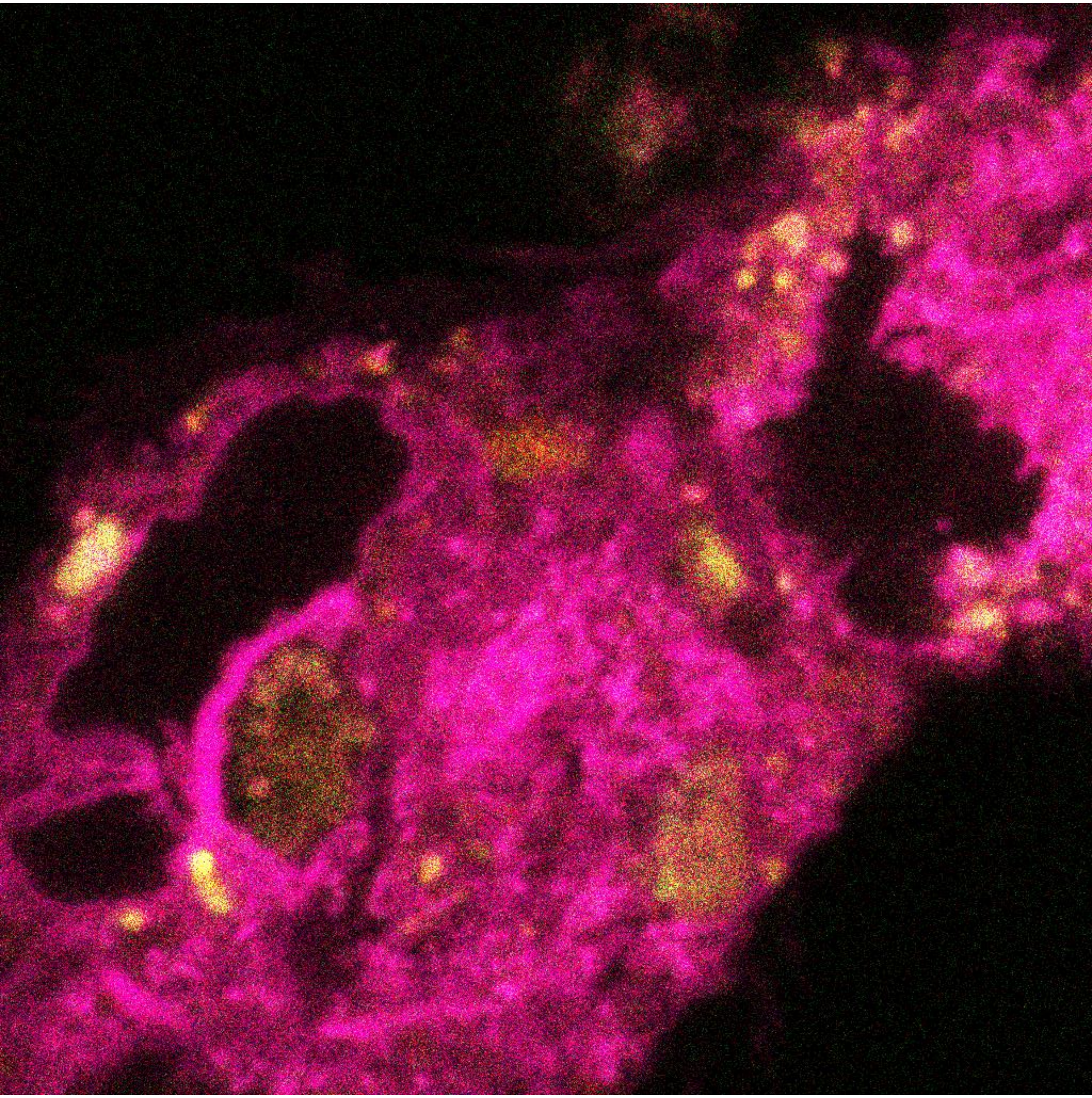

Figure 7C
CP2A NR 0.5mM+M2.5mM

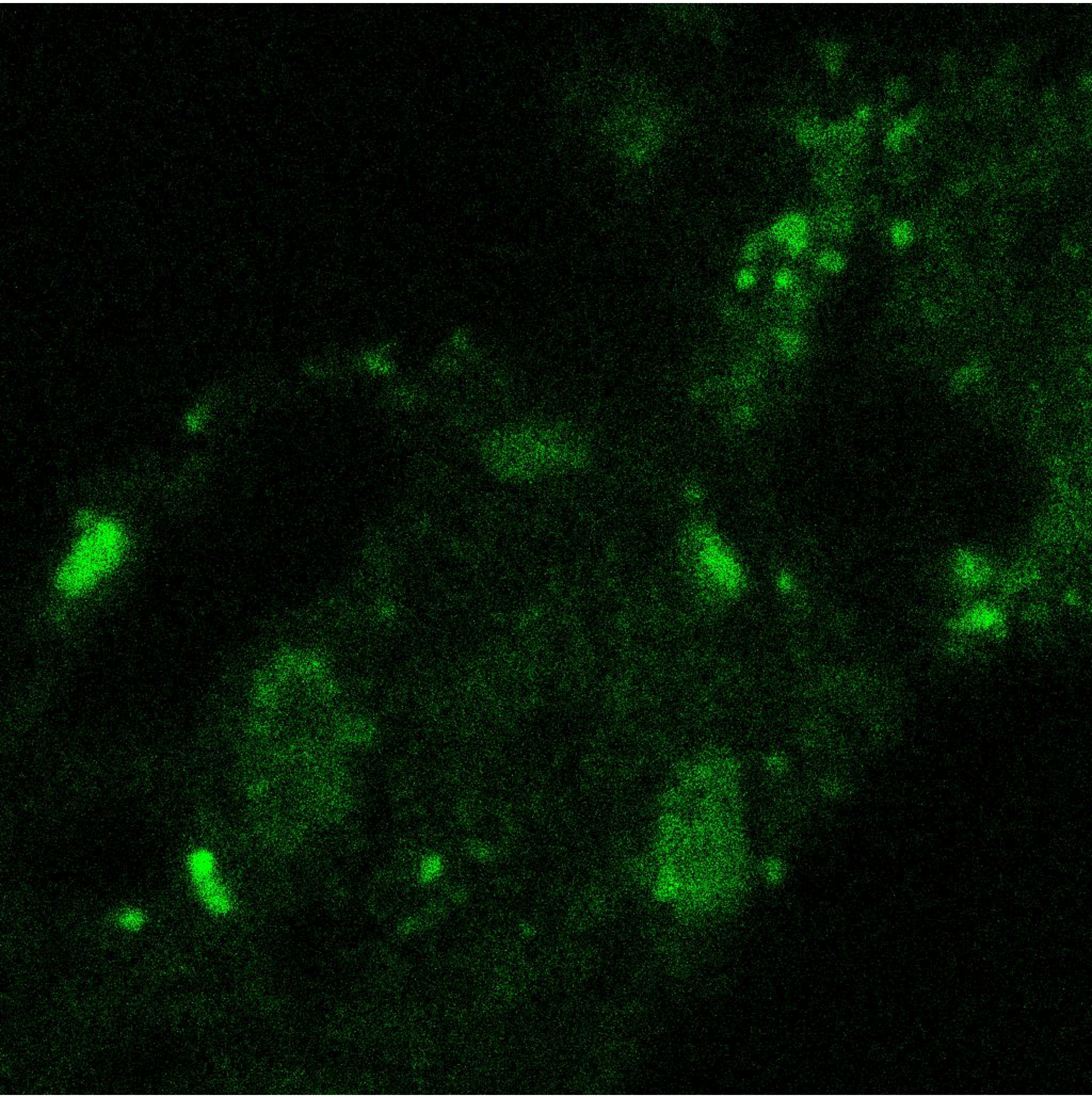

Figure 7C
CP2A NR 0.5mM+M2.5mM

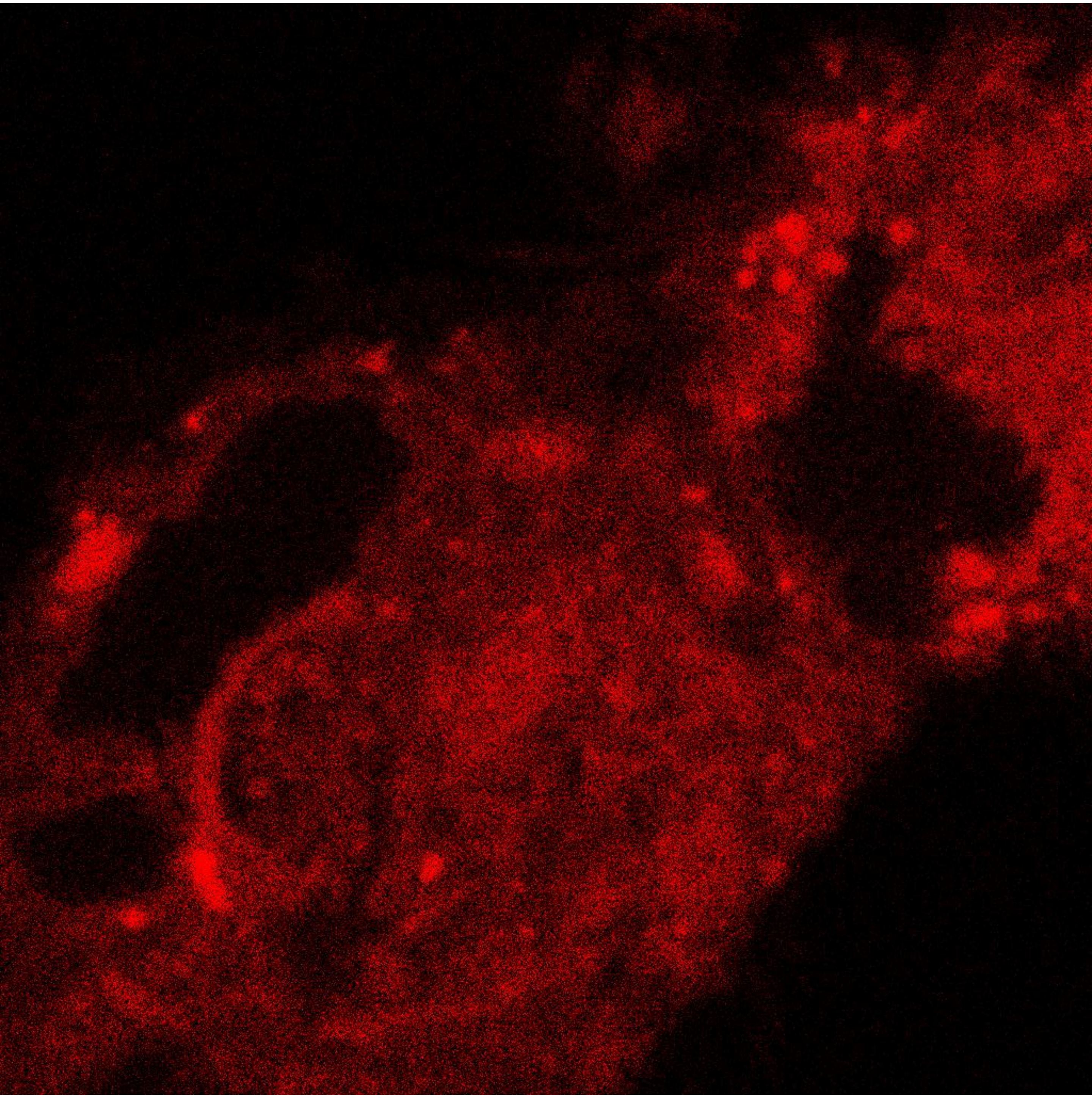

Figure 7C
CP2A NR 0.5mM+M2.5mM

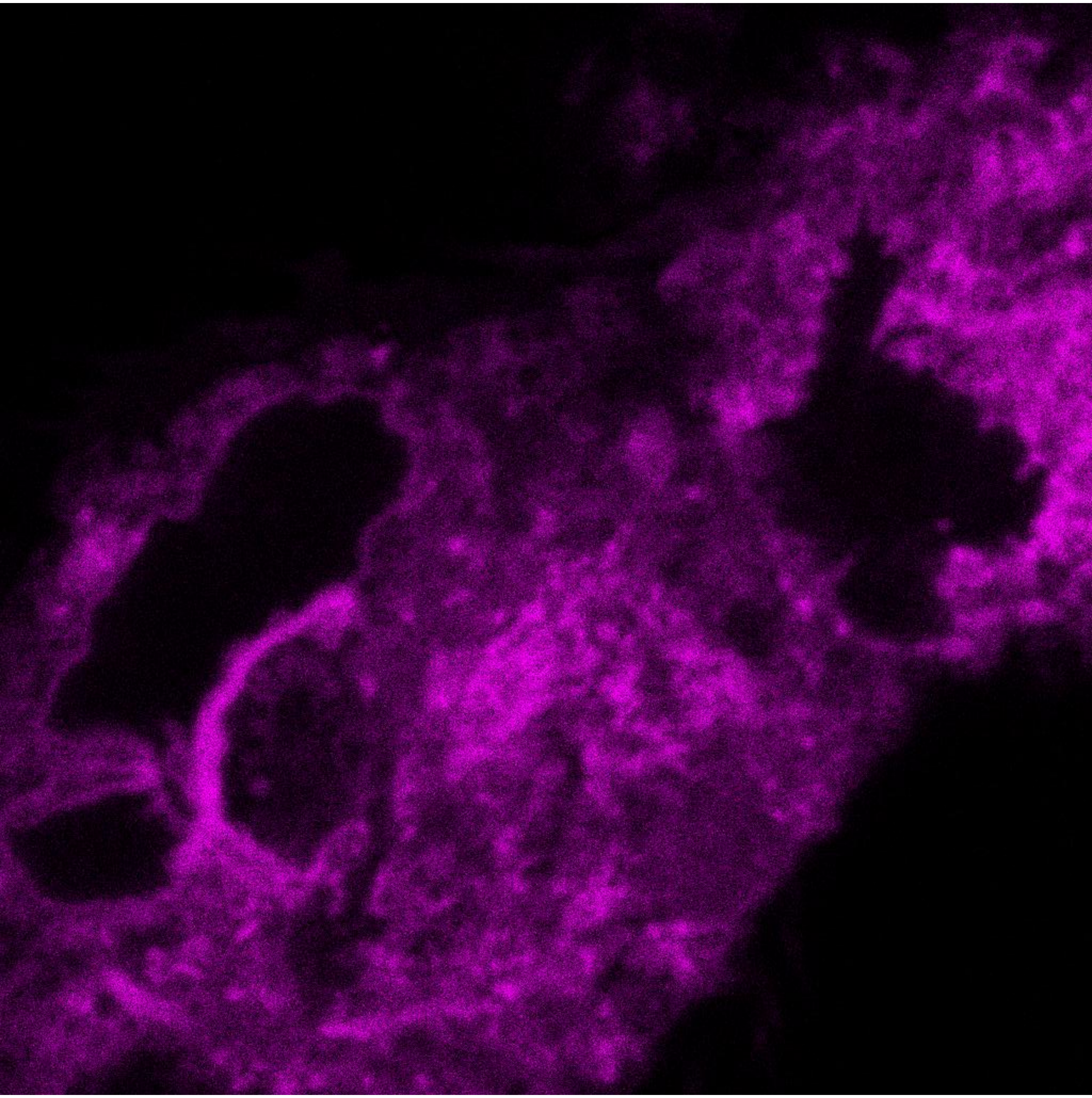

Figure 7C
CP2A NR 0.5mM+M2.5mM

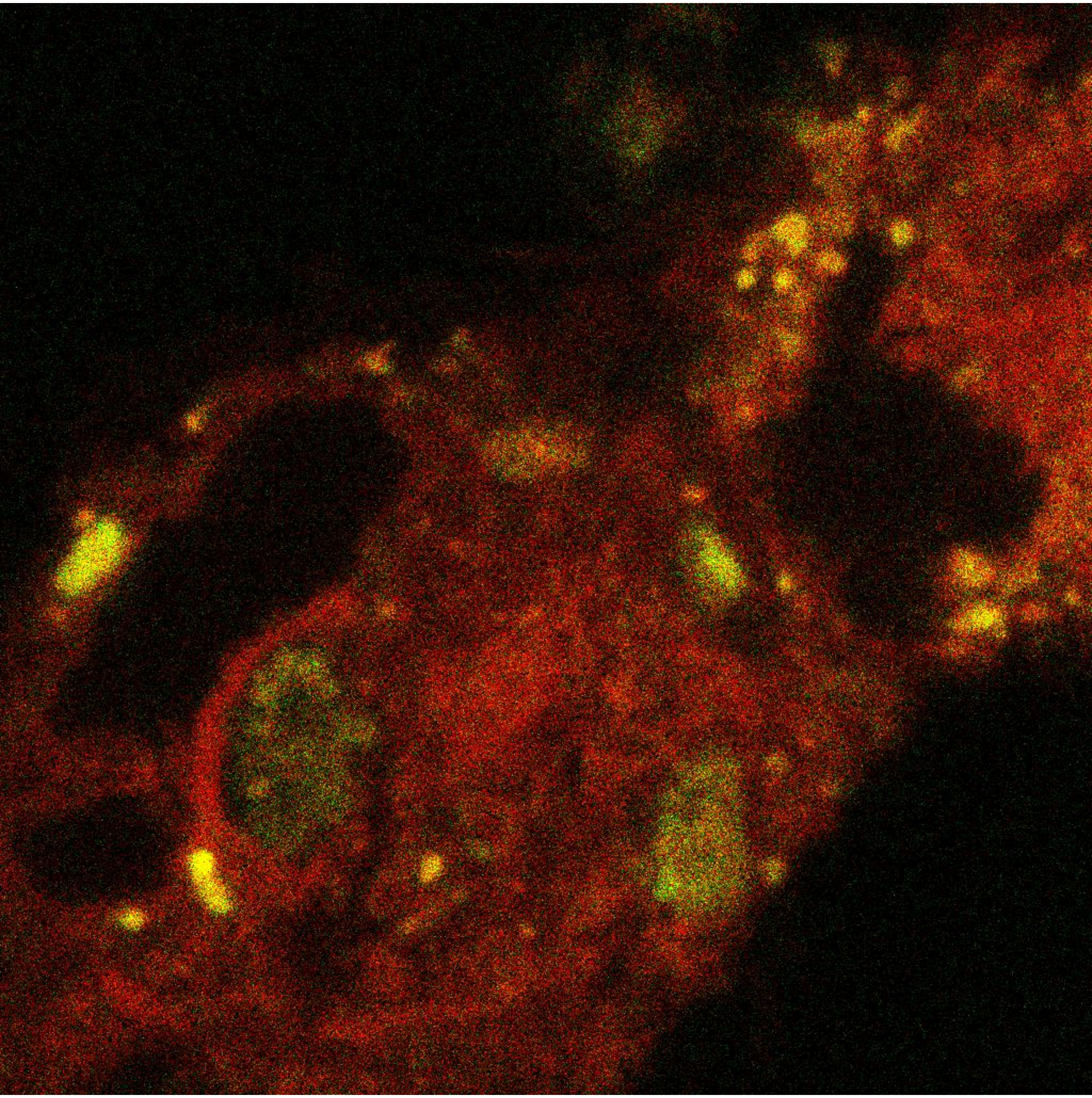

Figure 8A

NR

PGC-1α

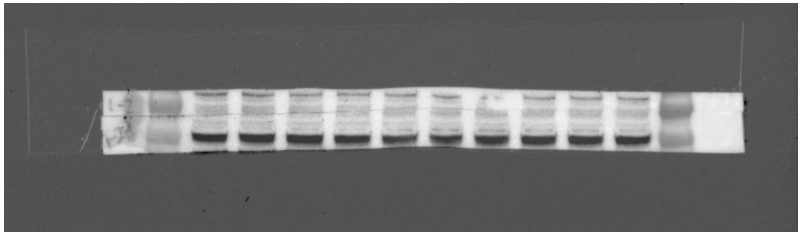

Figure 8A

NR

NDUFB10

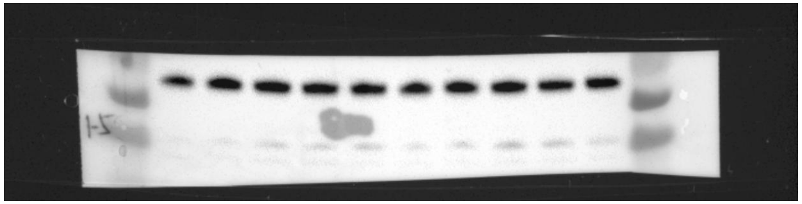

Figure 8A

NR

GAPDH

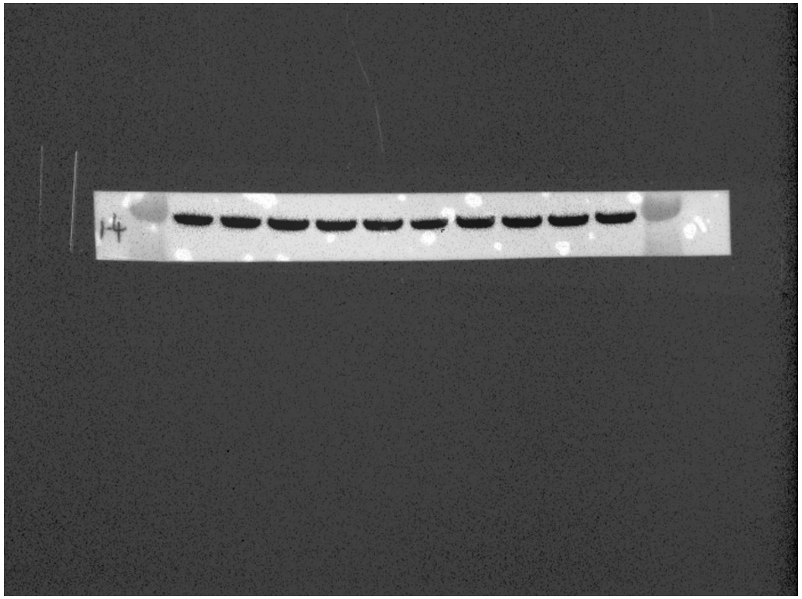

Figure 8A

Metformin

PGC-1α

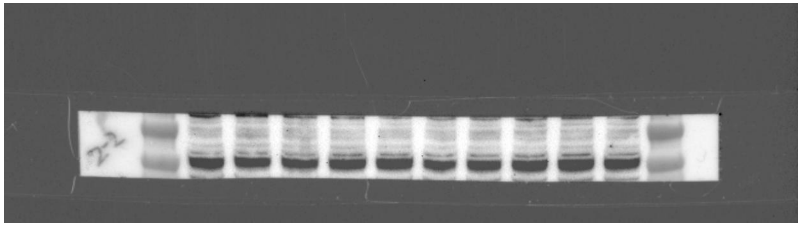

Figure 8A

Metformin

NDUFB10

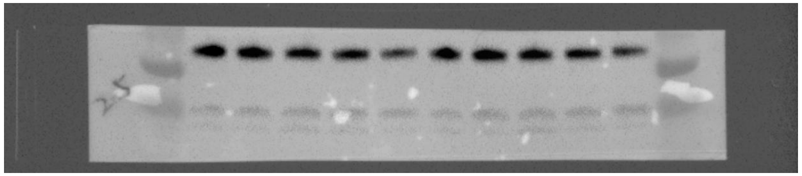

Figure 8A

Metformin

GAPDH

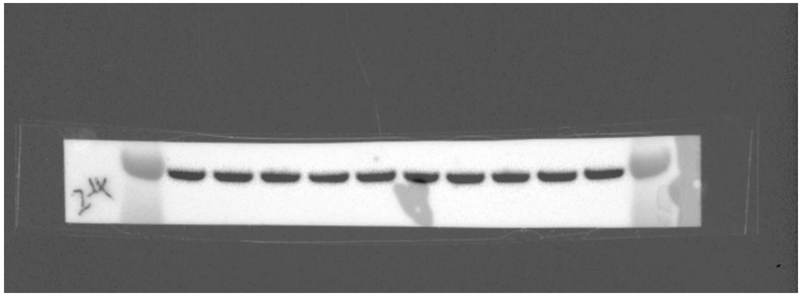

Figure 8A

NR+Metformin

PGC-1α

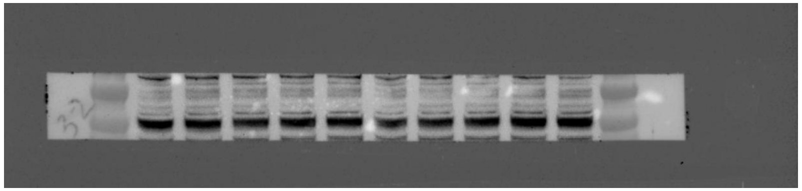

Figure 8A

NR+Metformin

NDUFB10

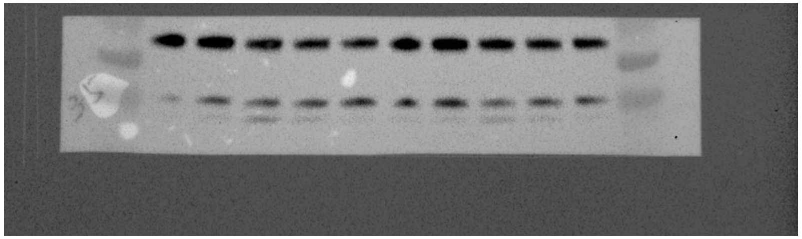

Figure 8A

NR+Metformin

GAPDH

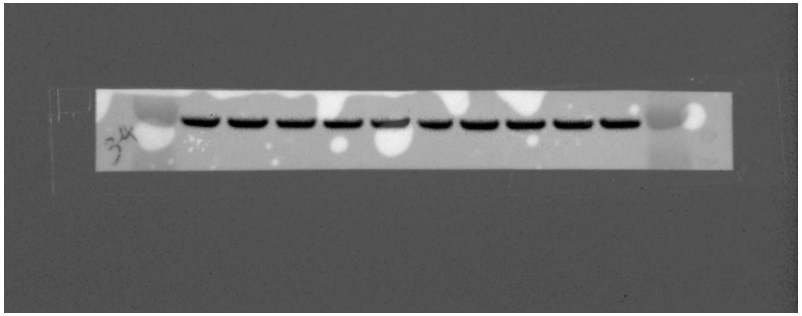

Figure 8B

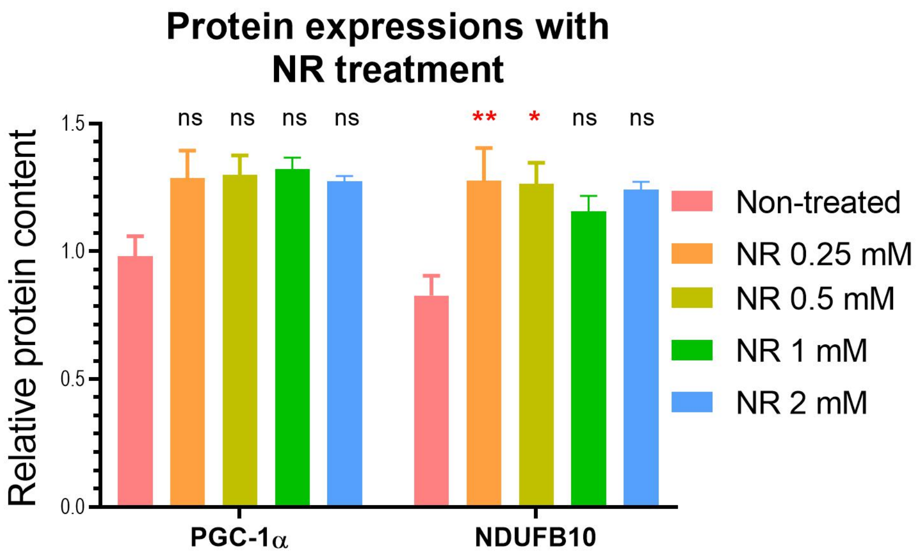

Protein expressions with NR treatment
Relative protein content
1.5
1.0
0.5
0.0
ns ns ns ns
** * ns ns
PGC-1α
NDUFB10
Non-treated
NR 0.25 mM
NR 0.5 mM
NR 1 mM
NR 2 mM

Figure 8C

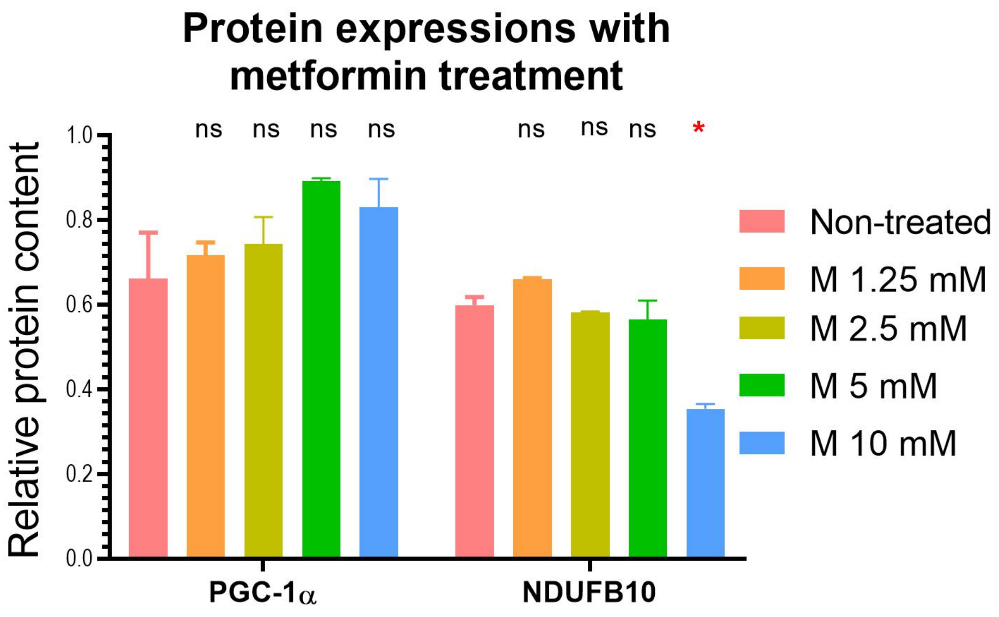
Protein expressions with metformin treatment
Relative protein content
1.0
0.8
0.6
0.4
0.2
0.0
ns
ns
ns
ns
ns
ns
ns
*
PGC-1α
NDUFB10
Non-treated
M 1.25 mM
M 2.5 mM
M 5 mM
M 10 mM

Figure 8D

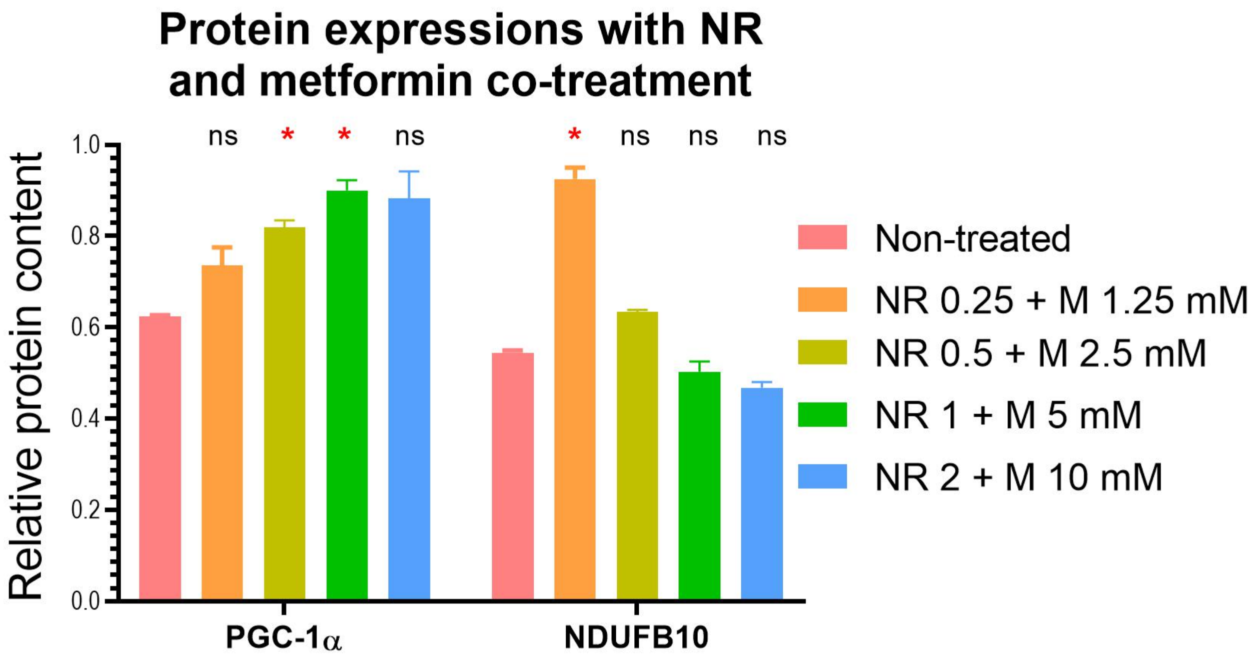

Protein expressions with NR and metformin co-treatment
Relative protein content
1.0
0.8
0.6
0.4
0.2
0.0
ns
*
*
ns
*
ns
ns
ns
PGC-1α
NDUFB10
Non-treated
NR 0.25 + M 1.25 mM
NR 0.5 + M 2.5 mM
NR 1 + M 5 mM
NR 2 + M 10 mM

Figure 8E

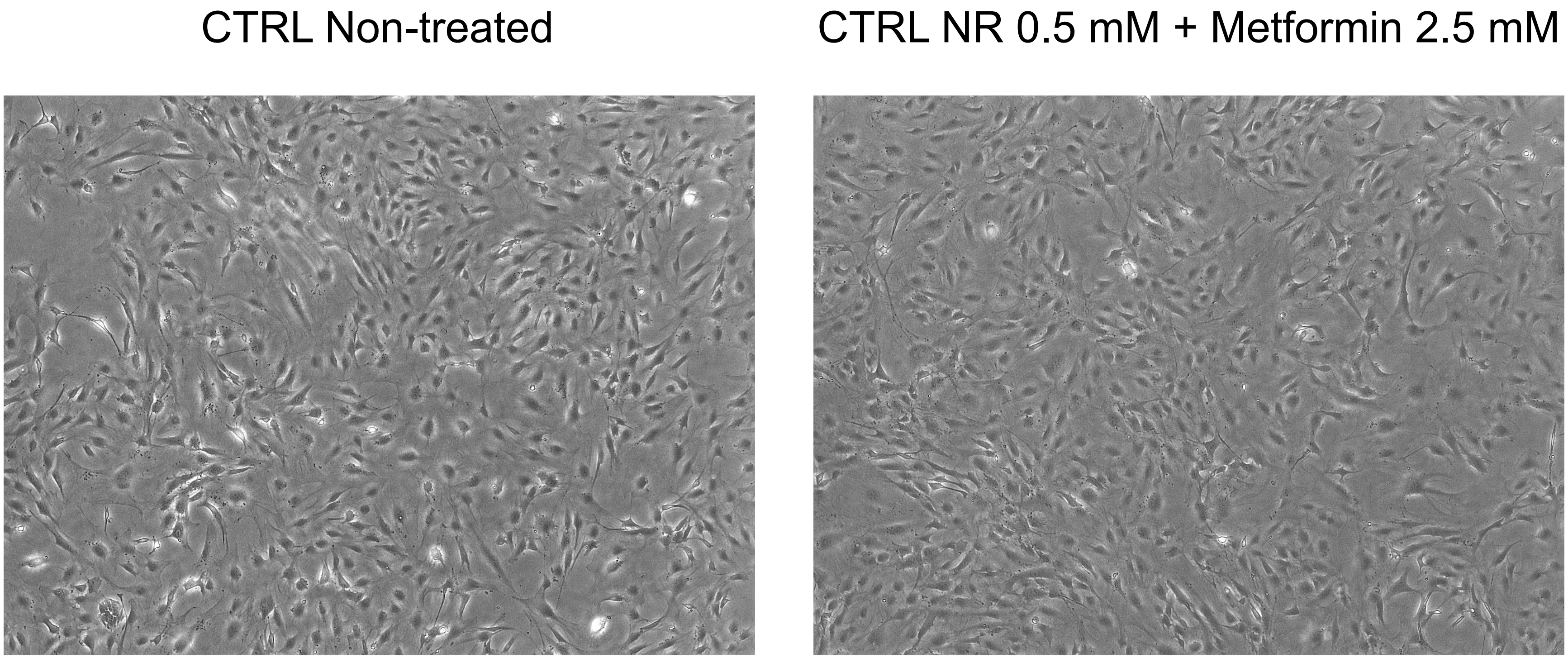

Figure 8E

WS5A Non-treated

WS5A NR 0.5 mM + Metformin 2.5 mM

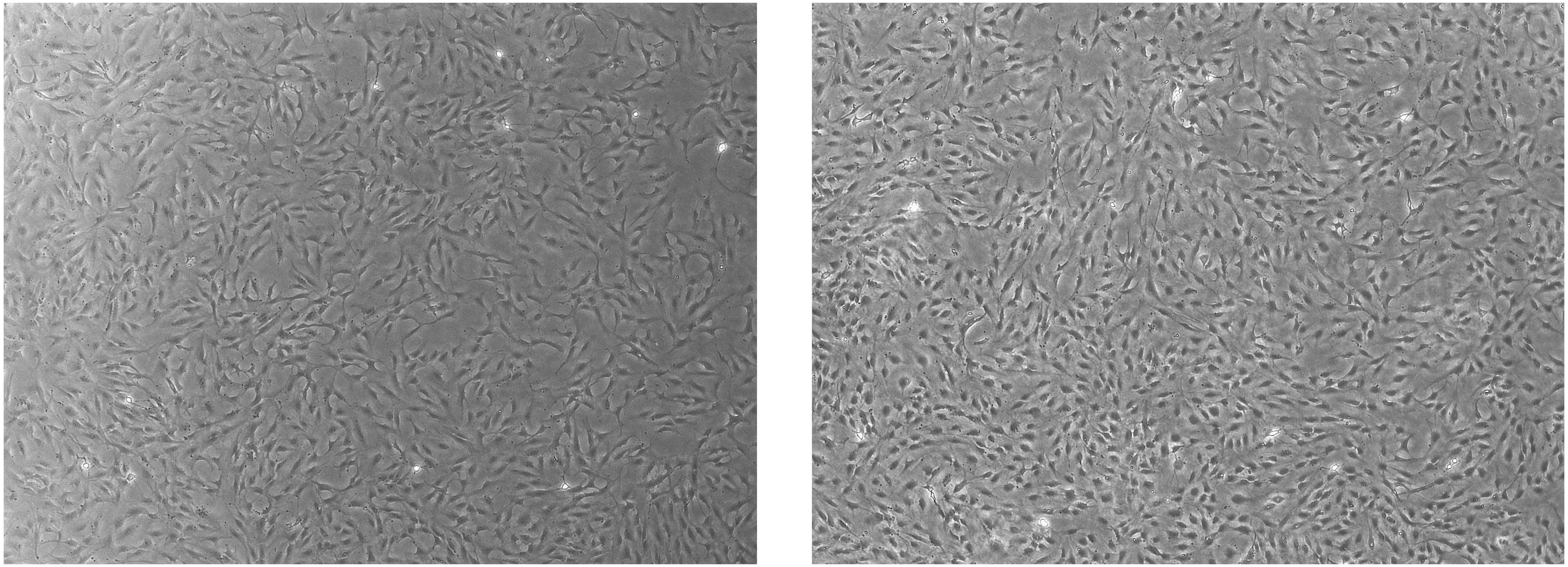

Figure 8E

CP2A Non-treated

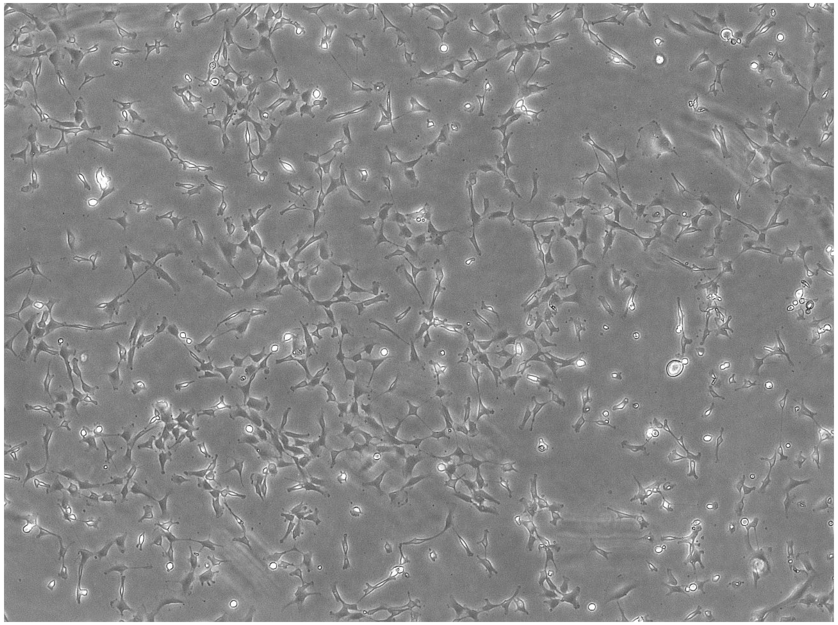

CP2A NR 0.5 mM + Metformin 2.5 mM

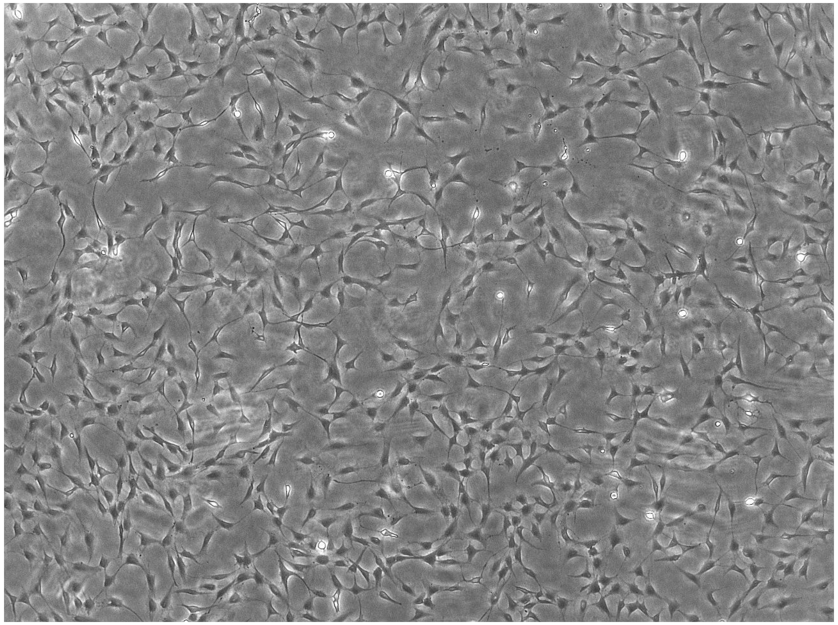

Figure 8F

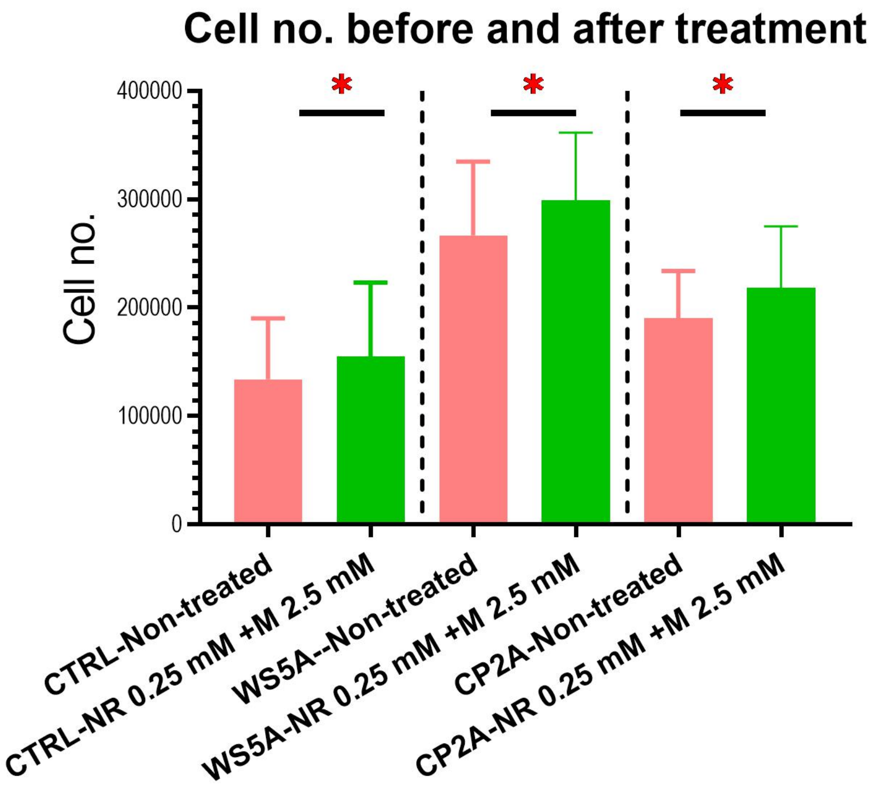

Cell no. before and after treatment
Cell no.
400000
300000
200000
100000
0
*
*
*
CTRL-Non-treated
CTRL-NR 0.25 mM +M 2.5 mM
WS5A--Non-treated
WS5A-NR 0.25 mM +M 2.5 mM
CP2A-Non-treated
CP2A-NR 0.25 mM +M 2.5 mM

Figure 8G

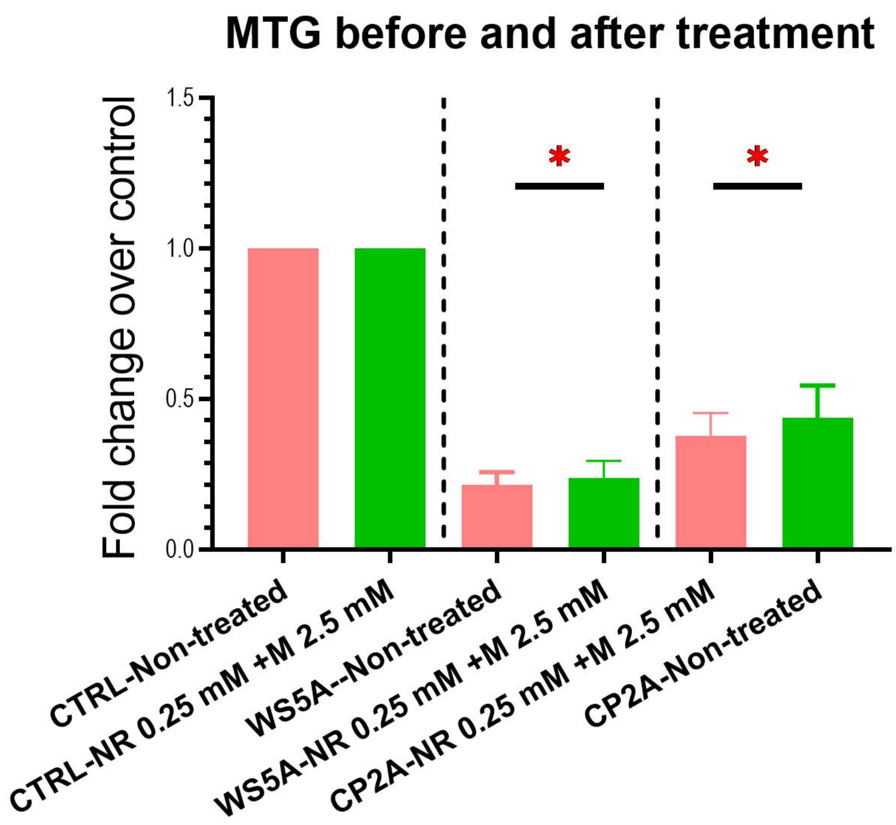

MTG before and after treatment
Fold change over control
1.5
1.0
0.5
0.0
*
*
CTRL-Non-treated
CTRL-NR 0.25 mM +M 2.5 mM
WS5A--Non-treated
WS5A-NR 0.25 mM +M 2.5 mM
CP2A-NR 0.25 mM +M 2.5 mM
CP2A-Non-treated

Figure 8H

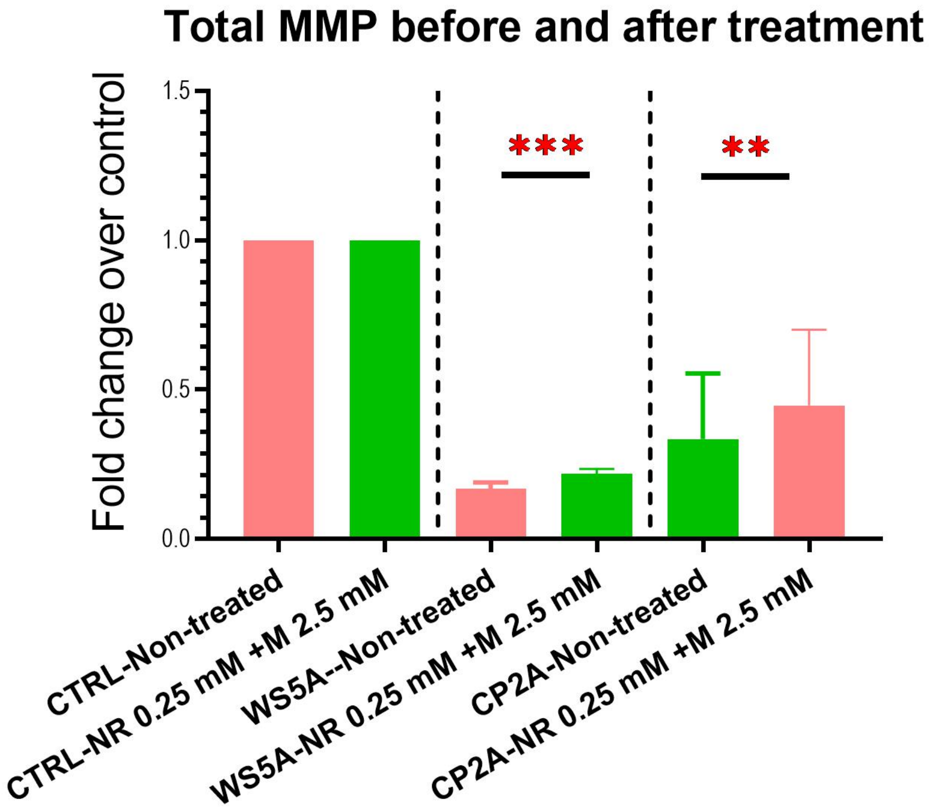

Total MMP before and after treatment
Fold change over control
1.5
1.0
0.5
0.0
***
**
CTRL-Non-treated
CTRL-NR 0.25 mM +M 2.5 mM
WS5A--Non-treated
WS5A-NR 0.25 mM +M 2.5 mM
CP2A-Non-treated
CP2A-NR 0.25 mM +M 2.5 mM

Figure 8I

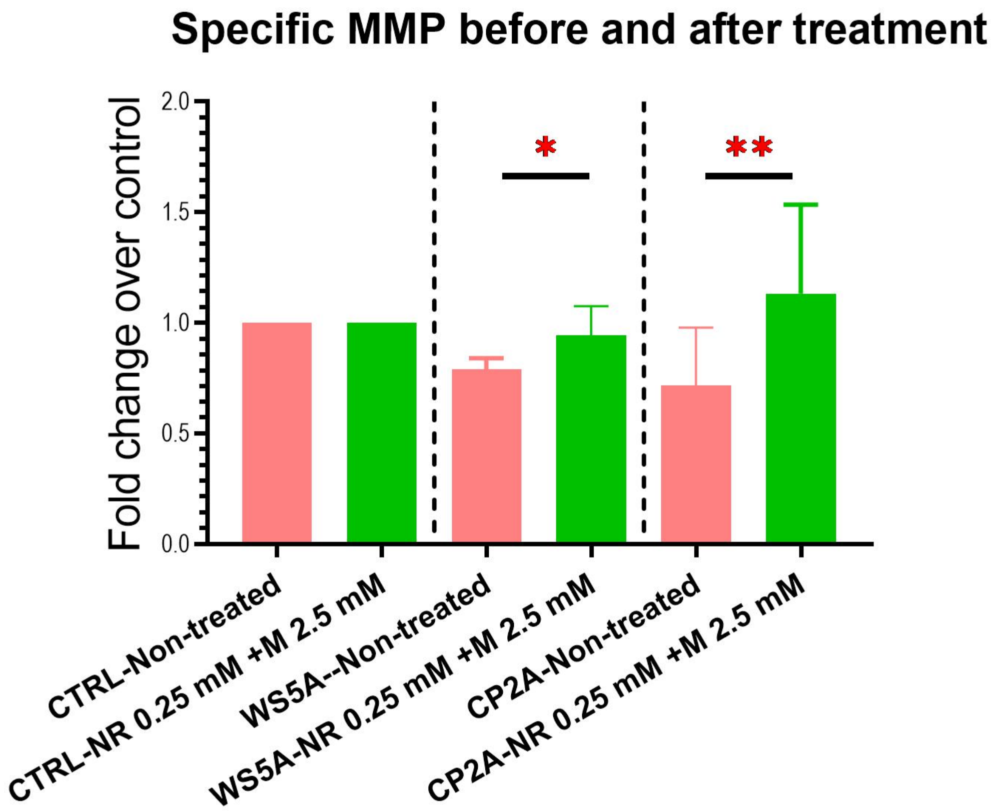
Specific MMP before and after treatment
Fold change over control
2.0
1.5
1.0
0.5
0.0
*
**
CTRL-Non-treated
CTRL-NR 0.25 mM +M 2.5 mM
WS5A--Non-treated
WS5A-NR 0.25 mM +M 2.5 mM
CP2A-Non-treated
CP2A-NR 0.25 mM +M 2.5 mM

Figure 8J

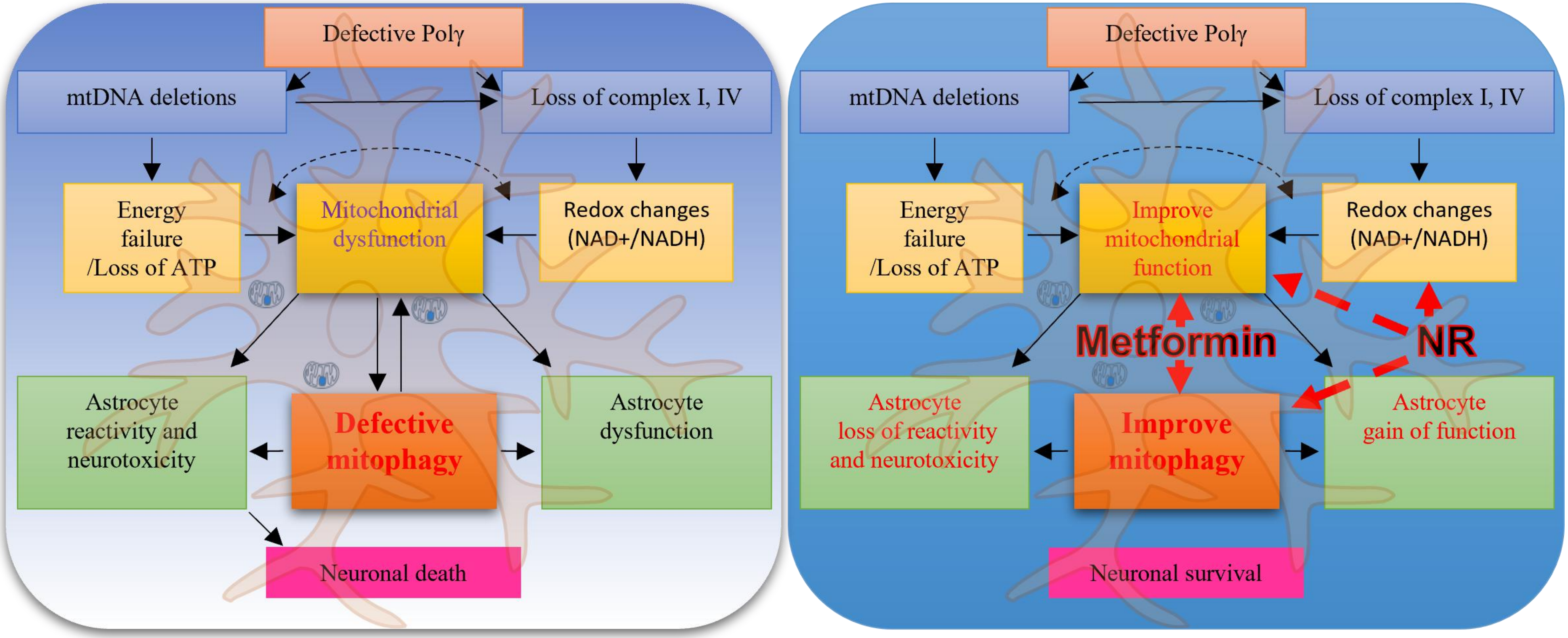

Defective Polγ
mtDNA deletions
Loss of complex I, IV
Energy failure /Loss of ATP
Mitochondrial dysfunction
Redox changes (NAD+/NADH)
Astrocyte reactivity and neurotoxicity
Defective mitophagy
Astrocyte dysfunction
Neuronal death
Defective Polγ
mtDNA deletions
Loss of complex I, IV
Energy failure /Loss of ATP
Improve mitochondrial function
Redox changes (NAD+/NADH)
Metformin
NR
Astrocyte loss of reactivity and neurotoxicity
Improve mitophagy
Astrocyte gain of function
Neuronal survival
