# Supplementary material for: Nicotinamide Riboside and Metformin Ameliorate Mitophagy Defect in Induced Pluripotent Stem Cell-Derived Astrocytes With POLG Mutations
Source: Front Cell Dev Biol. 2021 Sep 24;9:737304. doi: 10.3389/fcell.2021.737304 (PMC8497894; doi:10.3389/fcell.2021.737304)
Supplement: Supplementary file 3 [file Data_Sheet_3.doc]

**Supplementary Information on Data Availability Statements**

The raw data in this study was supplemented in supplemental files. The rest of the data that support the findings of this study are available from the corresponding authors upon reasonable request. The RNAseq analysis GEO accession number for the data reported in this paper is GSE168518.

All data generated or analyzed during this study are included in the link: https://www.jianguoyun.com/p/DZout-oQw67TCRjNhIEE
