# Supplementary material for: Nicotinamide Riboside and Metformin Ameliorate Mitophagy Defect in Induced Pluripotent Stem Cell-Derived Astrocytes With POLG Mutations
Source: Front Cell Dev Biol. 2021 Sep 24;9:737304. doi: 10.3389/fcell.2021.737304 (PMC8497894; doi:10.3389/fcell.2021.737304)
Supplement: Supplementary file 4 [file Table_1.docx]

***STAR★Methods***

***Key Resources Table***

| ***REAGENT or RESOURCE*** | **SOURCE** | **IDENTIFIER** |
| --- | --- | --- |
| ***Antibodies*** | | |
| anti-SOX2 | Abcam | Cat# ab97959, RRID:AB_2341193 |
| anti-NESTIN | Abcam | Cat# ab22035, RRID:AB_446723 |
| anti-NANOG | Abcam | Cat# ab80892, RRID:AB_2150114 |
| anti-PAX6 | Abcam | Cat# ab5790, RRID:AB_305110 |
| anti-OCT4 | Abcam | Cat# ab19857, RRID:AB_445175 |
| anti-GFAP | Abcam | Cat# ab4674, RRID:AB_304558 |
| anti-S100β | Abcam | Cat# ab196442, RRID:AB_2722596 |
| anti-CD44 | Abcam | Cat# ab189524, RRID:AB_2885107 |
| anti-EAAT-1 | Abcam | Cat# ab416, RRID:AB_304334 |
| anti-p62 | Abcam | Cat# ab109012, RRID:AB_2810880 |
| anti-Glutamine Synthetase (GS） | Abcam | Cat# ab64613, RRID:AB_1140869 |
| anti-p-AMPK (T183+T172) | Abcam | Cat# abab23875, RRID:AB_447741 |
| anti-AMPK | Cell Signaling Technology | Cat# 2532, RRID:AB_330331 |
| anti-MFN1 | Cell Signaling Technology | Cat# 14739, RRID:AB_2744531 |
| anti-OPA1 | Cell Signaling Technology | Cat# 80471 |
| anti-p-DRP1 (Ser616) | Cell Signaling Technology | Cat# 4494, RRID:AB_11178659 |
| anti-DRP1 | Cell Signaling Technology | Cat# 8570, RRID:AB_10950498 |
| anti-MFF | Cell Signaling Technology | Cat# 84580, RRID:AB_2728769 |
| anti-LC3B | Abcam | Cat# ab51520, RRID:AB_881429 |
| anti-LAMP2A | Abcam | Cat# ab125068, RRID:AB_10971511 |
| anti-mTOR | Cell Signaling Technology | Cat# 2983, RRID:AB_2105622 |
| anti-p-mTOR | Cell Signaling Technology | Cat# 2971, RRID:AB_330970 |
| anti-p-ULK1 | Cell Signaling Technology | Cat# 6888, RRID:AB_10829226 |
| anti-ULK1 | Cell Signaling Technology | Cat# 8054, RRID:AB_11178668 |
| anti-PGC-1β | Abcam | Cat# ab98285, RRID:AB_10673596 |
| anti-PGC-1α | Abcam | Cat# ab77210, RRID:AB_1603314 |
| anti-NDUFB10 | Abcam | Cat# ab196019 |
| anti-p-AKT | Cell Signaling Technology | Cat# 4060, RRID:AB_2315049 |
| anti-AKT | Cell Signaling Technology | Cat# 9272, RRID:AB_329827 |
| anti-PINK1 | Cell Signaling Technology | Cat# 6946, RRID:AB_11179069 |
| anti-Parkin | Cell Signaling Technology | Cat# 4211, RRID:AB_2159920 |
| anti-TOMM20 | Abcam | Cat# ab56783, RRID:AB_945896 |
| anti-p-SIRT1 | Cell Signalling Technology | Cat# 2314, RRID:AB_561516 |
| anti-GAPDH | Abcam | Cat# ab8245, RRID:AB_2107448 |
| Goat anti-Rabbit IgG - Alexa Flour^®^ 488 | Molecular Probes | Cat# A-11008, RRID:AB_143165 |
| Goat anti-mouse IgG - Alexa Flour^®^ 594 | Molecular Probes | Cat# A-11005, RRID:AB_141372 |
| Goat anti-chicken IgG - Alexa Flour^®^ 594 | Molecular Probes | Cat# A-11042, RRID:AB_2534099 |
| Swine anti-Rabbit HRP | Dako | Cat# P0217, RRID:AB_2728719 |
| Goat anti-Mouse HRP | Thermo Fisher Scientific | Cat# 62-6520, RRID:AB_2533947 |
| ***Chemicals, Peptides, and Recombinant Proteins*** | | |
| DAPI | Thermo Fisher Scientific | P36962 |
| MTG | Invitrogen | M7514 |
| FCCP | Abcam | ab120081 |
| MTDR | Invitrogen | M22426 |
| ***Critical Commercial Assays*** | | |
| Mitophagy Detection Kit | Dojindo | MD01-10 |
| ***Software and Algorithms*** | | |
| GraphPad Prism version 8 for Windows | GraphPad Software, Inc | https://www.graphpad.com/ |
| C6 Plus Workstation Computer and Software | BD Biosciences | https://www.bdbiosciences.com/us/instruments/research/cell-analyzers/bd-accuri/bd-accuri-c6-plus-options/c6-plus-workstation-computer-and-software/p/661391 |
| Image J software | NIH | https://imagej.nih.gov/ij/index.html |
| Image Lab Software | BioRad | https://www.bio-rad.com/en-us/product/image-lab-software?ID=KRE6P5E8Z |
